# Structure elucidation of nigricanoside A through enantioselective total synthesis†
†Electronic supplementary information (ESI) available: Complete experimental details and characterization data. See DOI: 10.1039/c5sc00281h



**DOI:** 10.1039/c5sc00281h

**Published:** 2015-03-12

**Authors:** Jie Chen, Panduka Koswatta, J. Robb DeBergh, Peng Fu, Ende Pan, John B. MacMillan, Joseph M. Ready

**Affiliations:** a Department of Biochemistry , UT Southwestern Medical Center , 5323 Harry Hines Blvd , Dallas , 75390-9038 , TX , USA . Email: john.macmillan@utsouthwestern.edu ; Email: joseph.ready@utsouthwestern.edu

## Abstract

Nigricanoside A was isolated from green alga, and its dimethyl ester was found to display potent cytotoxicity. Its scarcity prevented a full structure elucidation, leaving total synthesis as the only means to determine its relative and absolute stereochemistry and to explore its biological activity. Here we assign the stereochemistry of the natural product through enantioselective total synthesis and provide initial studies of its cytotoxicity.

## Abstract 2

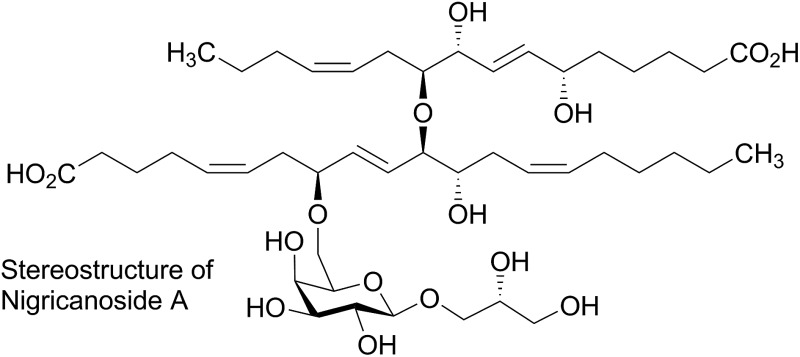
Total synthesis enabled the assignment of relative and absolute stereochemistry of nigricanoside A, which was reported to show potent cytotoxicity.

## Introduction

Nigricanoside A (**1**) was isolated in Prince Rupert Bay, Dominica from the green alga *Avrainvillea nigricans* by the Roberge and Andersen groups.^1^ To facilitate purification, the dicarboxylic acid was converted to the dimethyl ester (**2**). Nigricanoside A dimethyl ester was reported to inhibit the proliferation of human breast (MCF7) and colon (HCT-116) cancer cell lines with IC_50_'s of 3 nM. Additionally, MCF7 cells were arrested during mitosis, and were characterized by a disorganized microtubule spindle. Diester **2** modestly accelerated the polymerization of tubulin *in vitro*, but at concentrations >1000-fold above its IC_50_ values. Thus, it remains unclear if tubulin and/or microtubules are the direct targets of the nigricanosides.

^1^H and ^13^C NMR experiments revealed the subunits of nigricanoside A and their connectivity. Four domains comprise the natural product: a 16 carbon fatty acid, a 20 carbon fatty acid, galactose and glycerol. These substructures are also present in monogalactosyldiacylglycerols, which can account for up to 20% of the dry weight of algae.^2^ In the case of nigricanoside A, however, the fatty acids and galactose are connected with unprecedented ether bonds, not the ester bonds found in diacylglycerols. The initial heroic efforts of the Roberge and Andersen groups only provided sub-milligram quantities of **2**, which proved insufficient to completely establish the relative or absolute stereochemistry of the natural product. Efforts to obtain more material met with failure owing to an inability to locate additional *Avrainvillea nigricans* alga on subsequent collecting expeditions.^3^

The geometry of the five olefins and the identity of the sugar moiety were assigned based on coupling constants, but the other seven oxygenated stereocenters remain ambiguous. Total synthesis provides the only means to procure additional nigricanoside A for detailed biological investigation and complete structural elucidation.^4^ Several groups have reported studies towards this objective, but no structural assignment or total synthesis has been disclosed.^5^ The principle synthetic challenges presented by nigricanoside A include the 17 stereochemical elements, the two unprecedented ether bonds, and the high polarity of the natural product arising from extensive oxygenation.

## Results and discussion

In designing a synthesis, our primary objective was to design a flexible route that could access all 256 diastereomers (7 isolated stereocenters + d/l galactose). We planned to rely on asymmetric catalysis and chiral auxiliaries to provide multiple stereochemical configurations with equal facility. The initial selection of a target molecule was informed by the structure of trioxilin A3 (**4**), which features a *trans* diol at C11/C12 and likely arises from the hydrolysis of the corresponding epoxide, hepoxilin A3.^6^ Likewise, all monogalactosyldiacylglycerols isolated from green algae to date feature d-galactose. Finally, a model study suggested an anti relationship between the C6 and C9 allylic alcohols.^7^

The 20-C fatty acid was synthesized as shown in Scheme 2, and started with the addition of a terminal alkyne (**5**) to epoxide (*R*)-**6**.^8^ Semi-reduction provided the *cis*-olefin, and routine manipulations yielded the aldehyde **8**, which was alkynylated with the Bestmann–Ohira reagent.^9^ Use of sodium methoxide as the base for this reaction rather than the more common K_2_CO_3_ was critical to avoid epimerization of the C8′ stereocenter (nigricanoside A numbering).^10,11^ Separately, the acetylide derived from 1-heptyne opened glycidol (*S*)-**6** to install the C12′ stereocenter, and partial hydrogenation, followed by oxidative cyclization yielded the acetal **11** as an inconsequential mixture of diastereomers. Next, regioselective opening of the acetal and oxidation with the Dess–Martin periodinane gave the α-hydroxy aldehyde **12**. To join the right and left fragments of the 20-C fatty acid, alkyne **10** was subjected to hydrozirconation with Schwartz reagent and subsequent transmetalation with dimethylzinc.^12^ Addition to aldehyde **12** showed poor stereocontrol, even in the presence of optically active ligands.^13^ For that reason, the C11′ stereocenter was established through oxidation and chelate-controlled reduction^14^ to yield a protected version of trioxilin A3 (**13**) with at least 10 : 1 diastereoselectivity.^11,15^

**Scheme 1:**
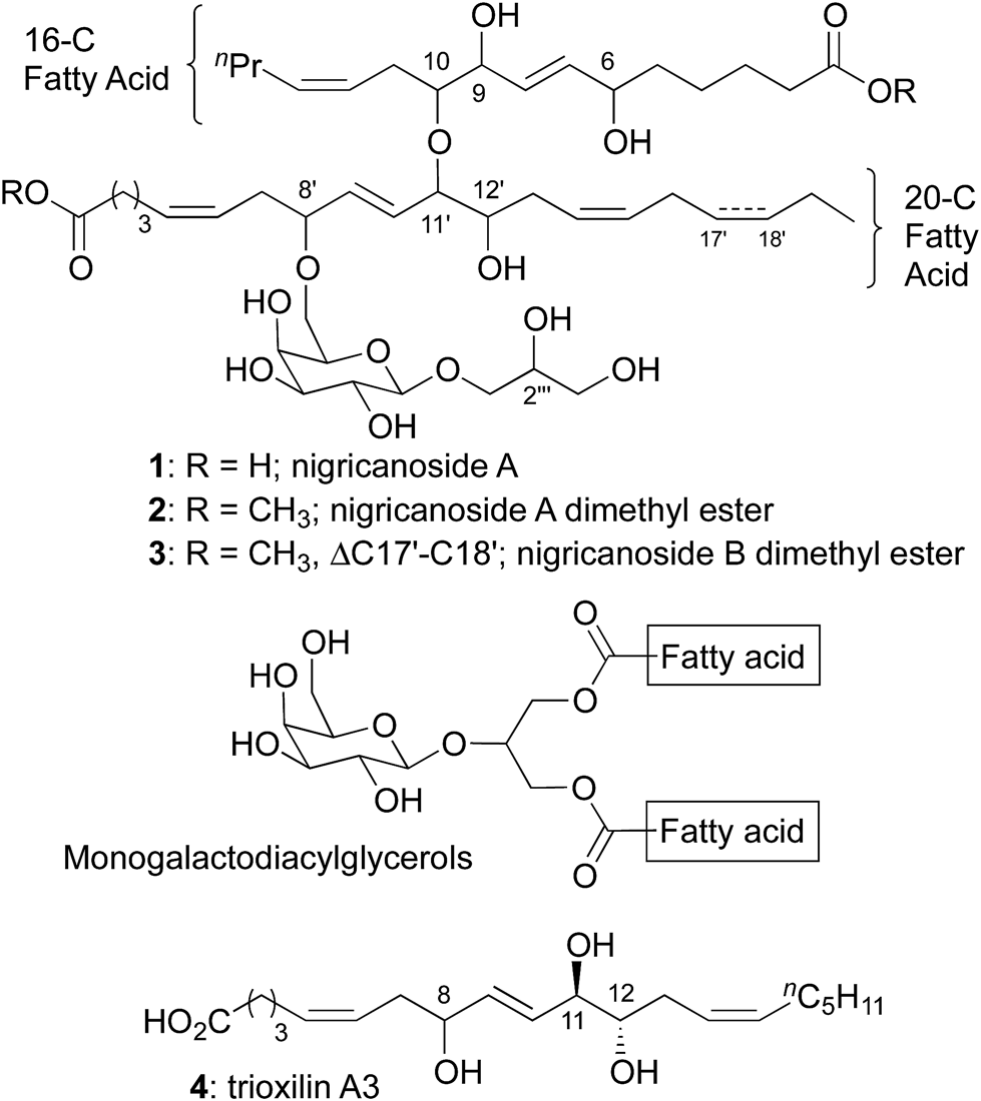
Structure and composition of nigricanoside A and related natural products.

**Scheme 2:**
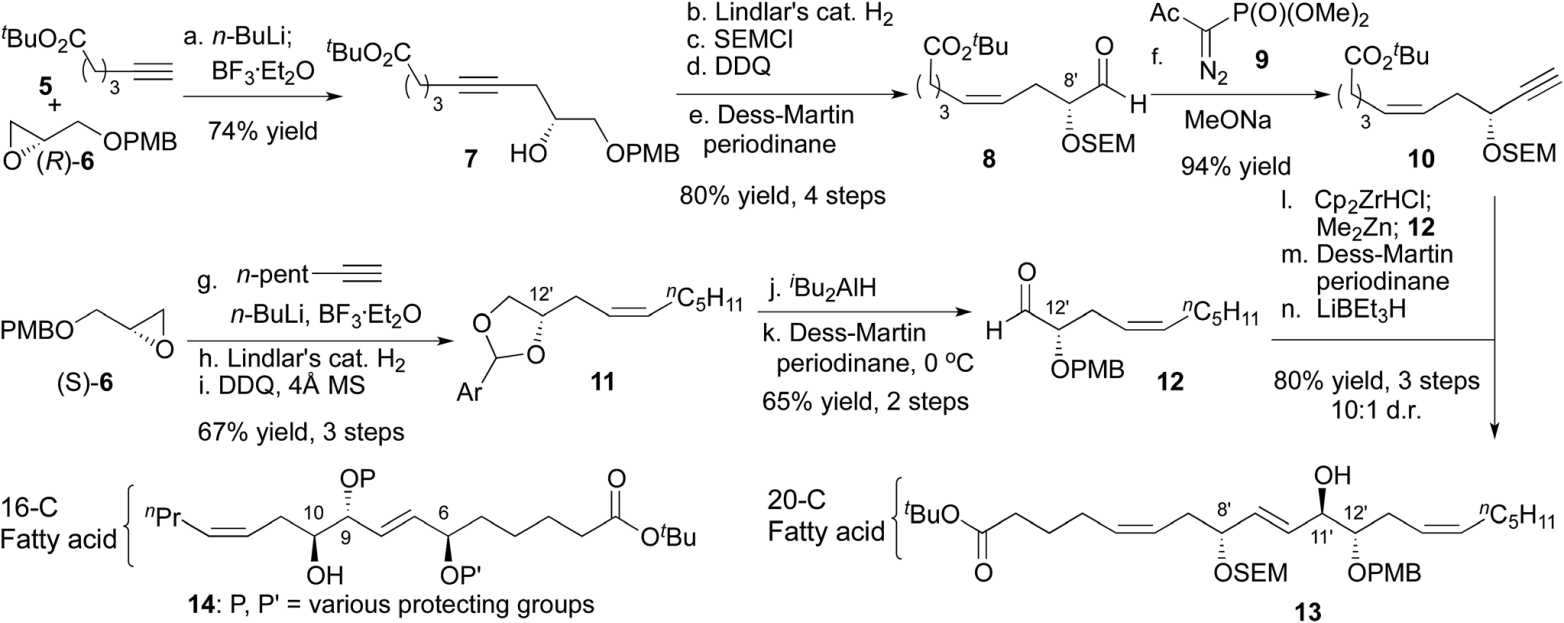
Synthesis of the 20-C fatty acid. (a) (*R*)-**6**, *n*-BuLi, BF_3_·Et_2_O, THF, –78 °C–rt, 74%. (b) Lindlar's cat. H_2_, EtOAc, 96%. (c) SEMCL, ^i^Pr_2_NEt, CH_2_Cl_2_, 0 °C–rt, 96%. (d) DDQ, pH = 7 buffer/CH_2_Cl_2_, 0 °C–rt, 97%. (e) Dess–Martin periodinane, NaHCO_3_, CH_2_Cl_2_, 0 °C, 90%. (f) Bestmann–Ohira reagent, NaOMe, THF, –78 °C, 94%. (g) (*S*)-**6**, *n*-BuLi, BF_3_·Et_2_O, THF, –78 °C–rt, (h) Lindlar's cat. H_2_, EtOAc. (i) DDQ, 4 Å MS, CH_2_Cl_2_, 67%, 3 steps. (j) ^i^Bu_2_AIH, CH_2_Cl_2_, –78 °C, 79%. (k) Dess–Martin periodinane, pyridine, CH_2_Cl_2_, 0 °C, 82%. (l) **10**, Cp_2_ZrHCL, CH_2_Cl_2_/toluene; Me_2_Zn, **12**, –78–0 °C, *ca.* 2 : 1 d.r. (m) Dess–Martin periodinane, NaHCO_3_, CH_2_Cl_2_, 0 °C, 83%, 2 steps. (n) LiBEt_3_H, CH_2_Cl_2_, –78 °C, 97%, >10 : 1 d.r.

With access to the 20-C fatty acid, we next sought to join it to the 16-C fatty acid. In this context, the scaffold of nigricanoside A might plausibly arise from addition of a C10 alcohol of the 16-C fatty acid to a C11′–C12′ epoxide. Accordingly, we prepared the 16-C fragment **14** using similar chemistry as described for the 20-C fatty acid (see ESI†). Unfortunately, all attempts to fuse the 20-C and 16-C subunits proved unsuccessful, including additions to either *cis*- or *trans*-epoxides or S_N_2 displacements of a variety of C11′ leaving groups of either stereochemistry. These results suggest that if this fragment union is biosynthetically relevant, it is likely enzyme catalyzed.

**Scheme 3:**
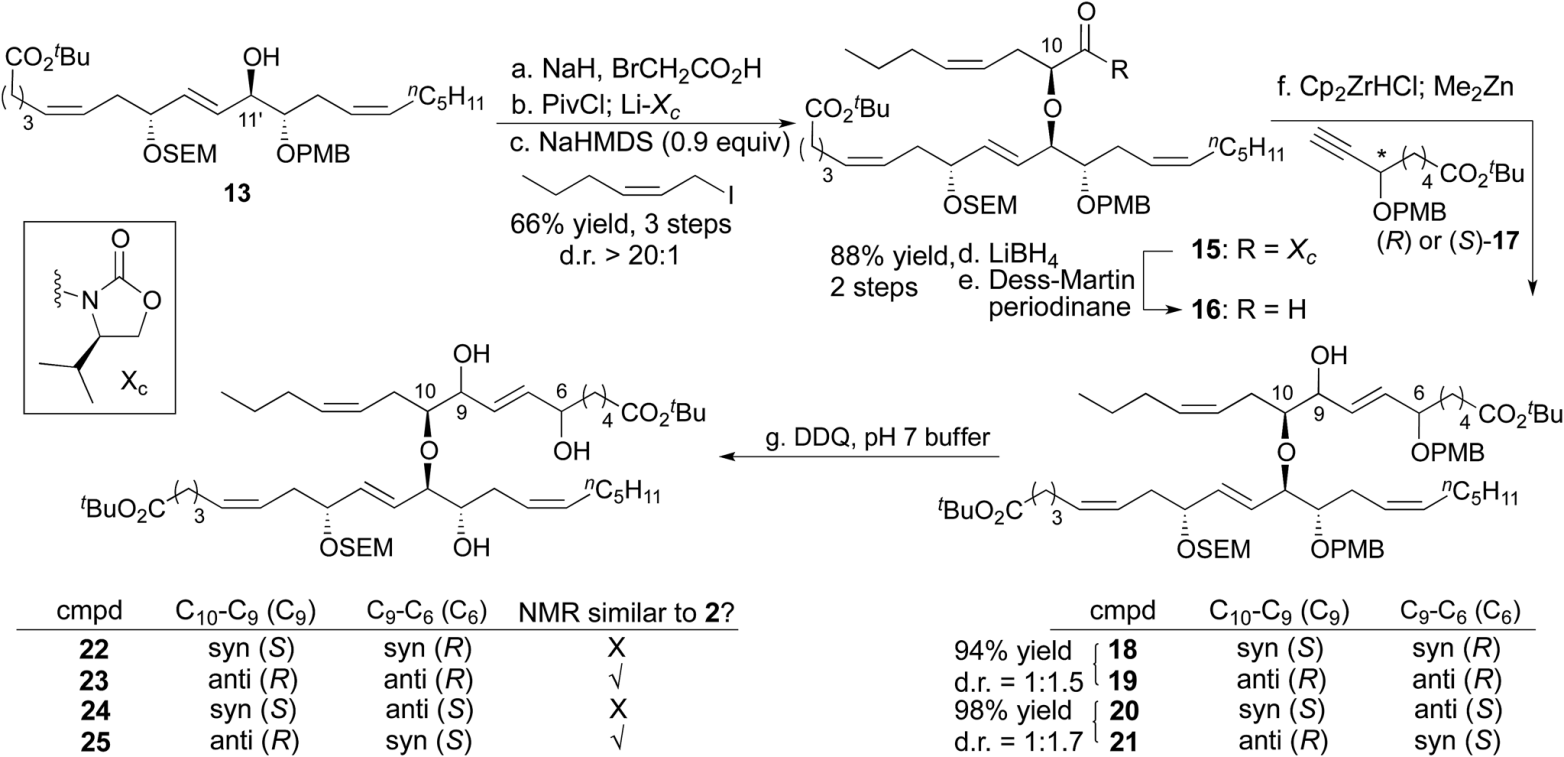
Synthesis of fatty acid portion of nigricanoside A. (a) NaH, BrCH_2_CO_2_H, THF/DMF = 2/1, 0 °C–rt, 91%. (b) PivCl, Et_3_N, Et_2_O, 0 °C; Li–X_c_, THF, rt, 91%. (c) (*Z*)-1-iodohex-2-ene, NaHMDS (0.9 equiv.), THF, –78 °C, 79%, d.r > 20 : 1, +9% recovered starting material. (d) LiBH_4_, MeOH, THF, 0 °C, 94%. (e) Dess–Martin periodinane, NaHCO_3_, CH_2_Cl_2_, 0 °C, 94%. (f) (+)-**17** or (–)-**17**, Cp_2_ZrHCl; Me_2_Zn; **16**, CH_2_Cl_2_/toluene, –78–0 °C. (g) DDQ, pH7 buffer/CH_2_Cl_2_, 0 °C–rt.

The full fatty acid skeleton of nigricanoside was successfully constructed in a stepwise manner (Scheme 3). Specifically, the C11′ secondary alcohol was alkylated with bromoacetate, and then the Evans chiral auxiliary was appended.^16^ Allylation with (*Z*)-1-iodohex-2-ene introduced the remaining aliphatic tail and set the C10 stereocenter with d.r = >20 : 1.^17^ This alkylation required the use of sub-stoichiometric quantities of base (0.9 equiv.). Use of a full equivalent led to substantial decomposition through a process that involved base-promoted elimination of the 20-C fatty acid (*i.e.* **13**) from the alkylated product, **15**. Nonetheless, with less than a full equivalent of base, 79% of the alkylated product (**15**) and 9% starting material could be isolated cleanly. Next, both enantiomers of alkyne **17** were synthesized separately using Noyori hydrogenation to control the C6 stereocenter.^18,19^ The vinyl zinc reagents derived from hydrozirconation of (*R*)-**17** and (*S*)-**17** were then added to aldehyde **16** to complete the carbon skeleton of the fatty acid portion of nigricanoside A. This addition generated a mixture of C9 epimers such that we had access to all four possible C6/C9 diastereomers (**18–21**). The PMB groups were removed with DDQ, providing 4 diasteromeric triols (**22–25**) that contained the complete fatty acid portion of nigricanoside, and facilitated comparison to the reported data for the natural product. The C9/C10 anti diastereomers more closely resembled the natural product than their C9/C10 syn congeners according to ^1^H NMR. The chemical shift of the C7 and C9 protons appeared to favor the anti/anti diastereomer **23**, so we elected to advance this stereochemical series in the synthesis, although the anti/syn diastereomer was also similar to the natural product.

**Scheme 4:**
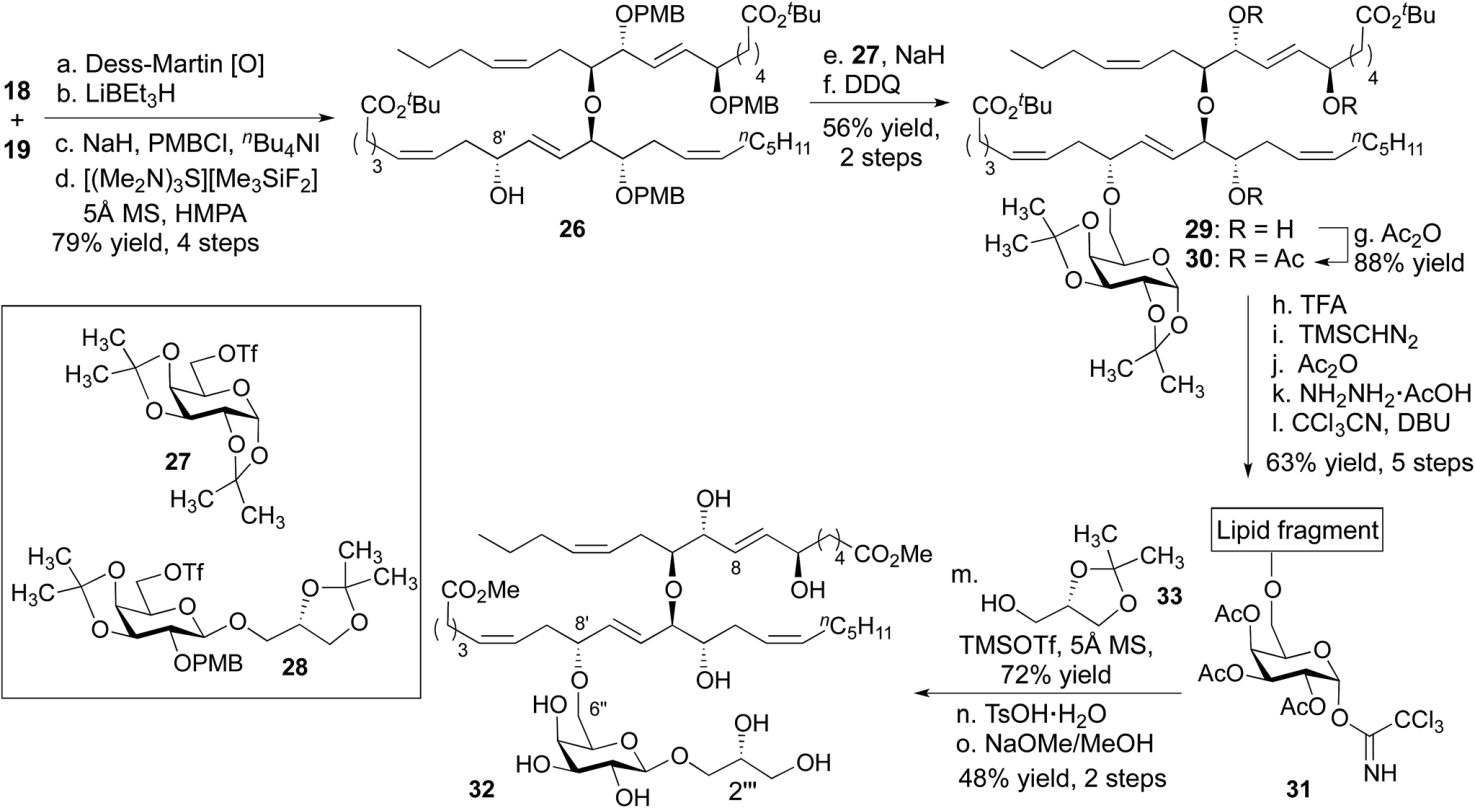
Synthesis of a nigricanoside A epimer. (a) Dess–Martin periodinane, NaHCO_3_, CH_2_Cl_2_, 0 °C, 96%. (b) LiBEt_3_H, CH_2_Cl_2_, –78 °C, 94%. (c) NaH, PMBCL, ^*n*^Bu_4_Nl, DMF, 0 °C–rt, 99%. (d) [(Me_2_N)_3_S][Me_3_SiF_2_], 4 Å MS, HMPA, 80 °C, 89% + 7% recovered starting material. (e) NaH, **27**, THF/DMF = 2/1, 0 °C–rt, 69%. (f) DDQ, pH = 7 buffer/CH_2_Cl_2_, 0 °C–rt 81%. (g) Ac_2_O, pyridine, cat. DMAP, CH_2_Cl_2_, 0 °C–rt, 88%. (h) TFA, CH_2_Cl_2_/H_2_O, rt. (i) TMSCHN_2_, MeOH/toluene, 0 °C. (j) Ac_2_O, pyridine, cat. DMAP, CH_2_Cl_2_, 0 °C–rt, 84%, 3 steps. (k) NH_2_NH_2_·AcOH, DMF, rt, 75%. (l) CCl_3_CN, cat. DBU, CH_2_Cl_2_, 0 °C–rt, quant. (m) **33**, TMSOTf, 5 Å MS, CH_2_Cl_2_, –78 °C, 72%. (n) cat. TsOH·H_2_O, MeOH, rt. (o) NaOMe, MeOH, 48% yield, 2 steps.

The C9 stereocenter was homogenized through chelate-controlled reduction of the corresponding ketone (Scheme 4).^12,15^ Two high-yielding protecting group manipulations generated a substrate suitable for introduction of the galactose ring (**26**). Specifically, the C8′ secondary alcohol was alkylated with the primary triflate **27**.^20^ This alkylation was only successful with an α-galactose. For example, with β-galactose **28**, elimination of the triflate dominated, affording the exocyclic olefin derived from **28** as the major product. After appending the α-galactose–diacetonide, the PMB groups were exchanged for acetates (**30**) so we could effect global deprotection under mild conditions as the last step of the synthesis. Next, the anomeric position of the galactose was activated through a sequence that involved removal of the acetonides and per-acylation of the galactose alcohols. The anomeric acetate was hydrolyzed, and addition of trichloroacetonitrile formed the trichloroacetimidate **31**.^21^ Reaction with (*S*)-solketal (**33**) in the presence of TMSOTf provided the full skeleton of nigricanoside A. In this glycosylation, 5 Å MS were uniquely effective. Remarkably, 4 Å MS inhibited formation of the desired product, a difference we attribute to the decreased basicity of 5 Å MS compared to 4 Å MS.^22^

On the cusp of completion, we were eager to remove the acetonide and acetate groups, which was accomplished with *p*-toluene sulfonic acid and NaOMe, respectively. We were concerned, however, by the ominous observation that the final diester (**32**) could not be dissolved in the DMSO-*d*_6_/C_6_D_6_ (2 : 25) mixture used in the original isolation. Ultimately, we were able to coerce the synthetic compound into solution by concentrating it from a solution in *d*_4_-methanol to form a thin film in a vial (concentration from water/CH_3_CN provided a solid). The ^1^H NMR of our synthetic material did not match that reported for the natural product. Moreover, the synthetic compound was inactive against HCT-116 and MCF-7 cells, whereas nigricanoside A dimethyl ester was reported to show low nM toxicity against these cell lines.

**Scheme 5:**
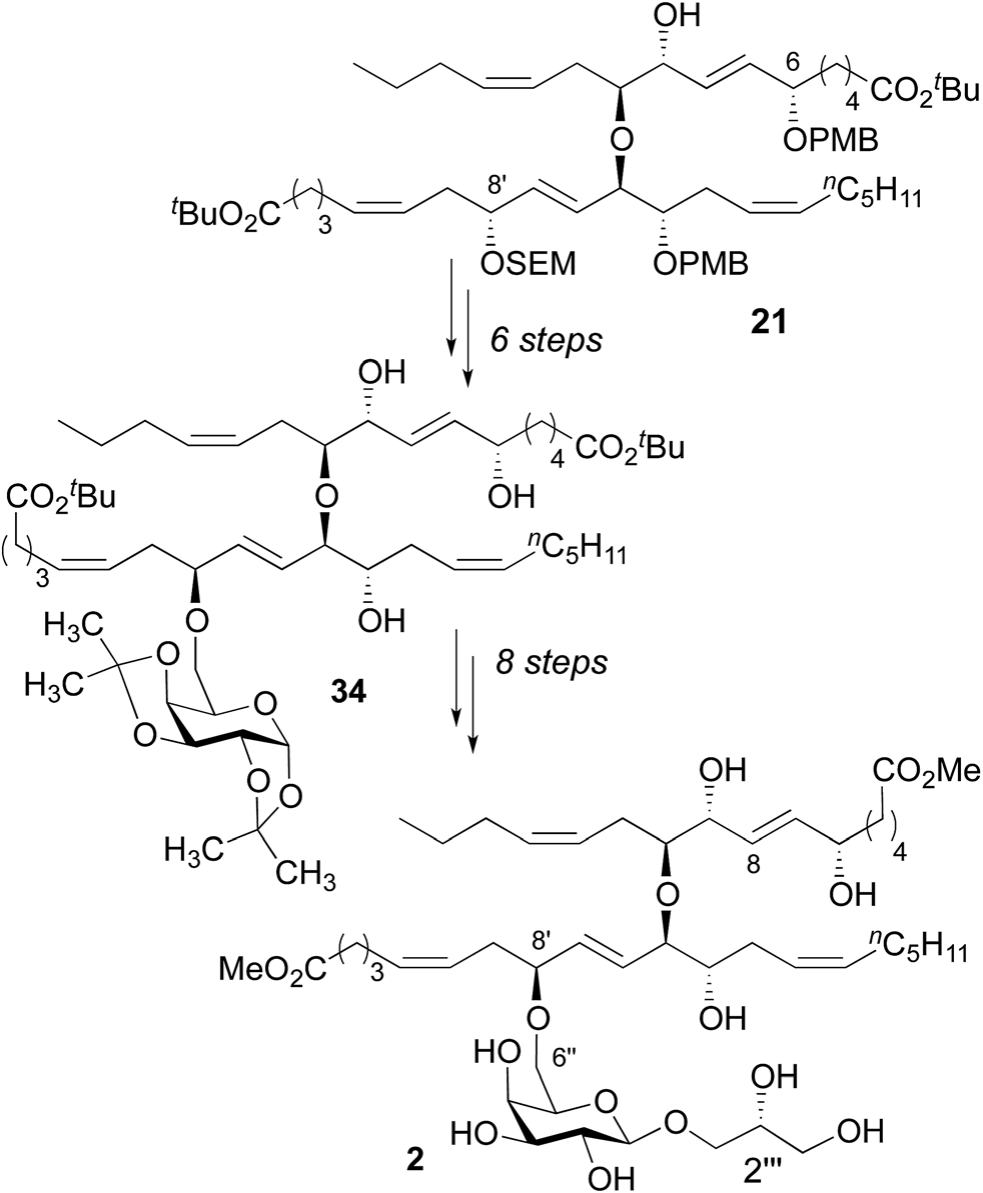
Synthesis of nigricanoside A dimethyl ester (**2**). See ESI† for synthetic details.

While we were disappointed to have prepared an isomer of the natural product, our reliance on asymmetric catalysis and chiral auxiliaries to dictate stereochemistry in our synthesis provided substantial flexibility. By design, we could prepare nearly any other diastereomer using the same overall strategy. In this context, the most obvious differences between the ^1^H NMR spectra for diester **32** and nigricanoside A dimethyl ester were associated with the C7–C8 *trans* olefin. This observation suggested that the natural product might feature a C6/C9 syn relationship. To test this hypothesis, the fatty acid fragment **21** was converted to the triol **34** using substantially the same chemistry as described above, with the addition of a Mitsunobu reaction to invert C8′ (Scheme 5, see ESI†).^23^ This latter inversion was based on the conjecture, which ultimately proved correct, that the two fatty acid fragments likely possessed the same relative stereochemistry within the 1,2,5-triol moieties. Finally, we proceeded to install the glycerol subunit analogously to the path developed for **32**. The 1D (^1^H, ^13^C) and 2D NMR data (COSY, HSQC, HMBC) of the dimethyl ester (**2**) exactly matched that reported for nigricanoside dimethyl ester. Optical rotation values indicated that we had prepared the natural enantiomer ([*α*]^20^ = –22, *c* = 0.1 CH_2_Cl_2_; lit [*α*]^25^ = –42, *c* = 0.24 CH_2_Cl_2_). By contrast, the C2′′′ epimer of **2** was clearly distinct from the natural product. In particular, the five resolvable C–H resonances of the glycerol subunit were shifted by 0.03–0.16 ppm relative to reported data for nigricanoside dimethyl ester.

We were surprised to find that neither **2** nor its C2′′′ epimer showed any toxicity towards HCT116 or MCF7 cells up to 10 μM. The isolation group additionally observed no toxicity from our synthetic material nor did they detect any mitotic arrest arising from treatment with synthetic **2**. None of the original sample is available for side-by-side comparison in biological assays, but it appears that nigricanoside A is not cytotoxic.

## Conclusions

**Fig. 1:**
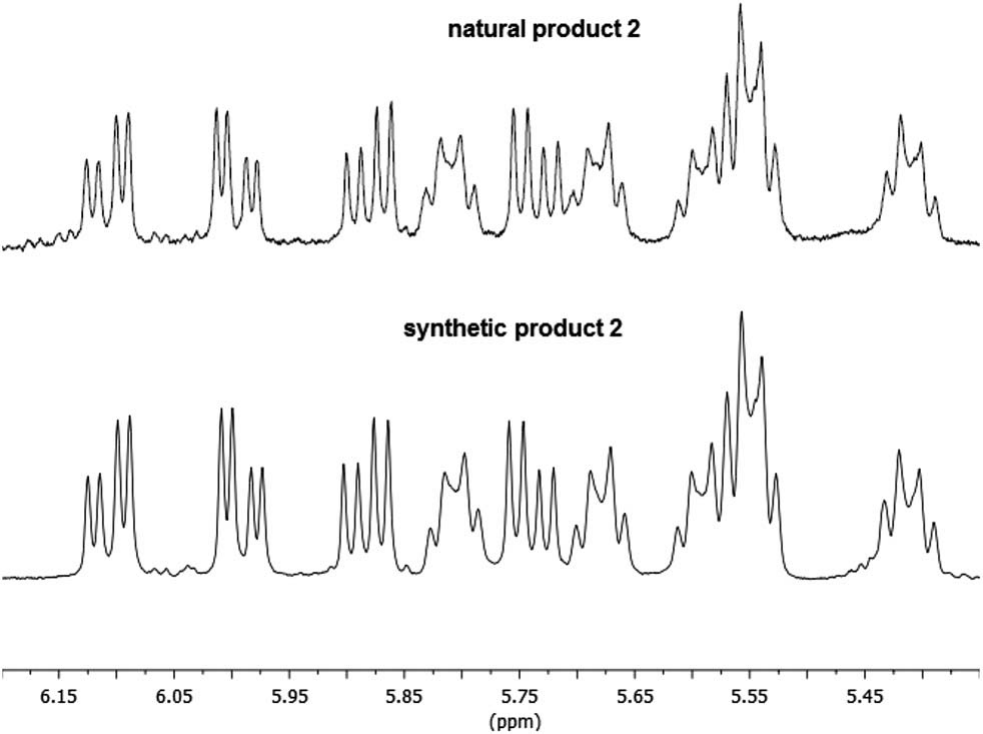
Olefin region of the ^1^H NMR of synthetic and natural nigricanoside A dimethyl ester.

The absence of biological activity for synthetic **2** presents an enigma that remains to be resolved. No ambiguity exists regarding the structure; the natural and synthetic material yield matching spectra and optical rotations. Moreover, all of the diastereomers we have prepared show clearly distinct ^1^H NMR spectra, minimizing the likelihood that we prepared a diastereomer of the natural product that coincidentally yields identical spectra.^24^ To highlight the identity of our synthetic material with the naturally derived material, Fig. 1 shows an overlay of the olefin region of synthetic and natural **2**, which illustrates that not only do the chemical shifts and coupling constants match, the complex patters of the peaks are identical. Likewise, the isolation group documented several biological activities associated with nigricanoside A dimethyl ester including cytotoxicity, mitotic arrest and tubulin polymerization. These activities were observed in both crude fractions and purified materials. The rigor of their studies argues against an artifactual result. The naturally occurring samples show approximately 90% purity. Minor resonances in the published ^1^H NMR spectrum could represent an unidentified source for the observed biological activity. Intriguingly, the only clear peaks for the minor component resemble the C7–C8 olefin resonances, suggesting a structural relationship to nigricanoside A. Finally, diester **2** was isolated along with a close congener, nigricanoside B dimethyl ester (**3**, Scheme 1 above). The Anderson and Roberge groups found that this secondary metabolite was more than 100-fold less active than nigricanoside A, which would be a surprisingly large drop in activity for a small structural change. A more likely interpretation in our view is that an unidentified natural product co-eluted with nigricanoside A dimethyl ester. Remarkably, the high potency reported for the natural product (∼3 nM) would require sub-nanomolar toxicity for any minor contaminant. This possibility should provide incentive for future efforts to identify the unknown highly active antimitotic.
