# Supplementary material for: Structure elucidation of nigricanoside A through enantioselective total synthesis
Source: Chem Sci. 2015 Mar 12;6(5):2932–7. doi: 10.1039/c5sc00281h (PMC4751885; doi:10.1039/c5sc00281h)
Supplement: Supplementary file 1 [file SC-006-C5SC00281H-s001.pdf]

Electronic Supplementary Material (ESI) for Chemical Science.
This journal is © The Royal Society of Chemistry 2015

Structure Elucidation of Nigricanoside A Through Enantioselective Total Synthesis

Jie Chen, Panduka Koswatta, J. Robb DeBergh, Peng Fu, Ende Pan, John B. MacMillan* and Joseph M. Ready*

**^{1}H NMR and ^{13}C NMR of synthetic Nigricanoside A Dimethyl Ester 2**	S2
**Figure S1.-S5. Comparison of synthetic Nigricanoside A Dimethyl Ester 2 to natural product**	S3-S5
**Table S1. ^{1}H and ^{13}C NMR Data for synthetic Nigricanoside A Dimethyl Ester 2**	S6-S7
**Methods and Materials**	S8
**Experimental procedures and characterization data**	
**Synthesis of the 20-C fatty acid 13**	S9-S13
**Synthesis of the 16-C fatty acid 14**	S13-S16
**Synthesis of nigricanoside A dimethyl ether epimer 32**	S16-S26
**Synthesis of nigricanoside A dimethyl ether 2**	S27-S32
**Figure S6.-S7. Comparison of 4 diasteromeric triols (22-25) to nigricanoside A dimethyl ester 2**	S33-S34
**Figure S8. Selective TOCSY experiment of the mixture (23 (anti, anti) and 22 (syn, syn))**	S35
**NMR Spectra**	S36-S106

^{1}H NMR Spectrum of synthetic Nigricanoside A Dimethyl Ester **2** at 600 MHz in 25:2 C_6D_6/DMSO-d_6

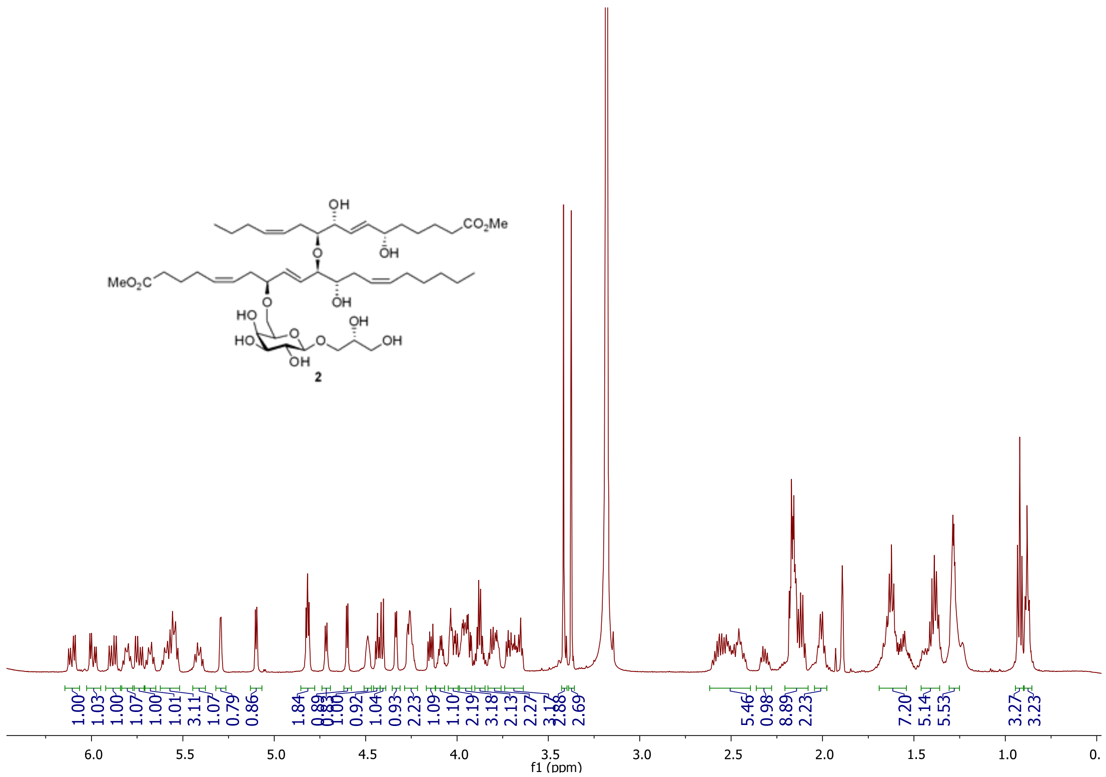

^{13}C NMR Spectrum of synthetic Nigricanoside A Dimethyl Ester **2** at 100 MHz in 25:2 C_6D_6/DMSO-d_6

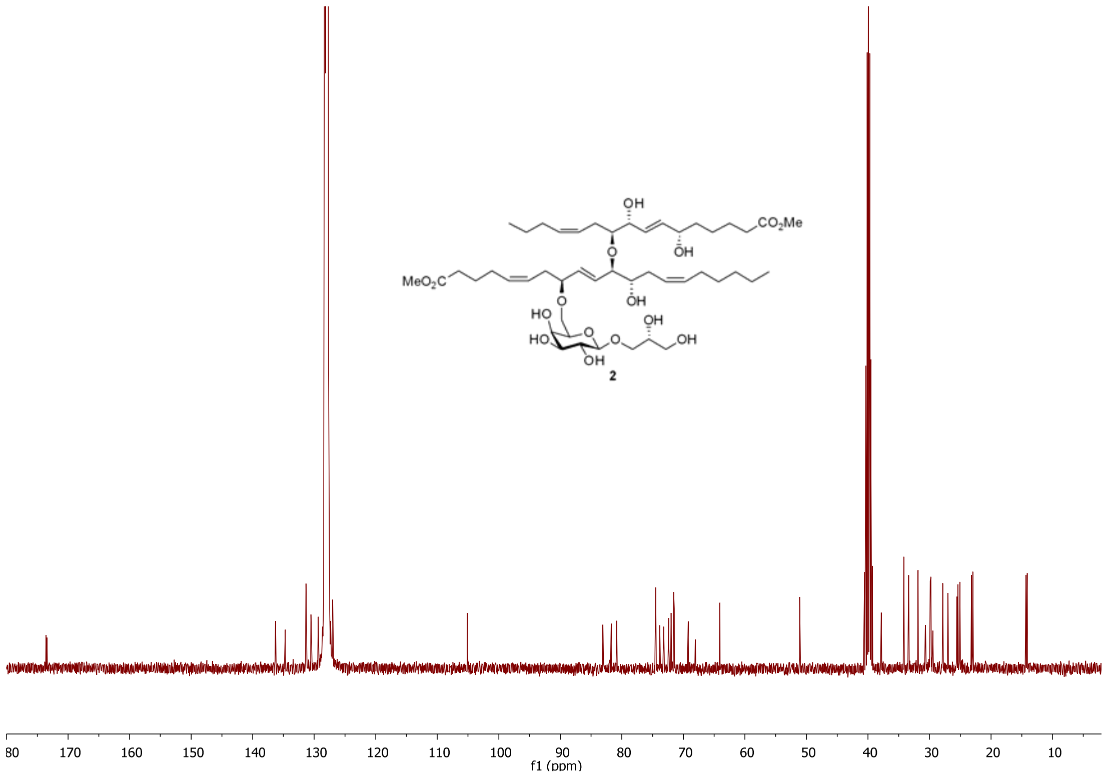

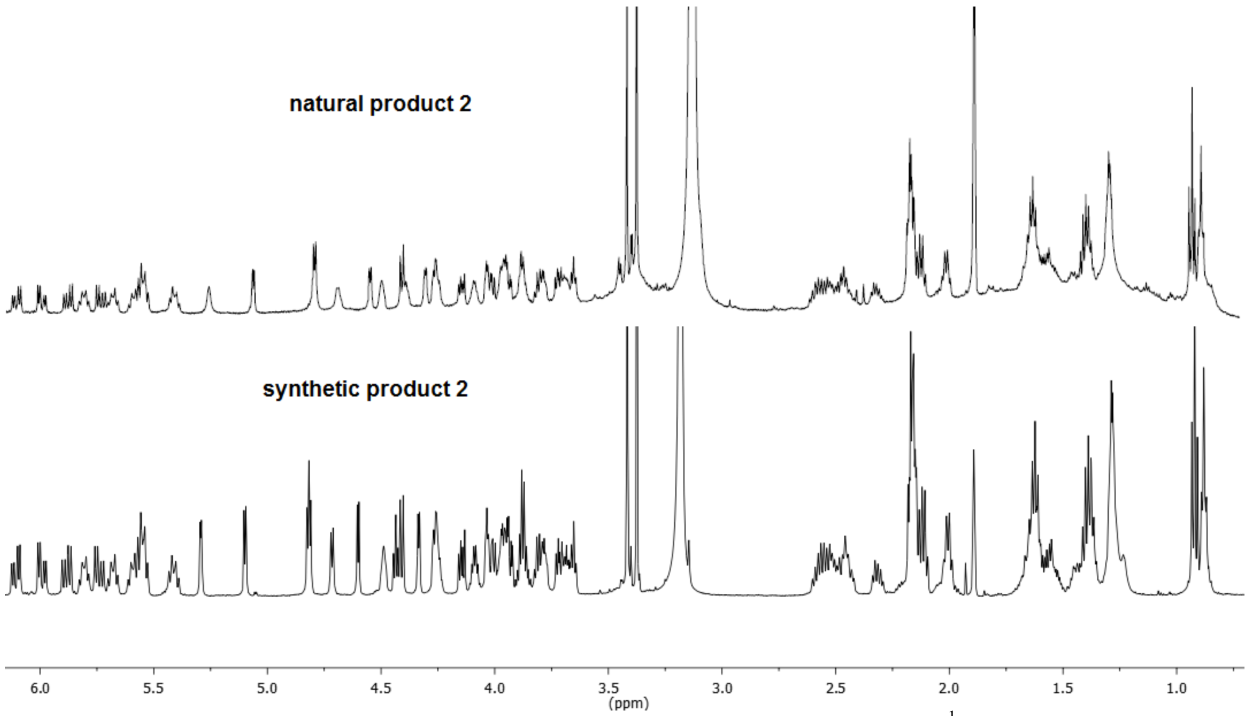

Figure S1. Comparison of synthetic nigricanoside A dimethyl ester **2** to natural product (^{1}H NMR). Spectrum of the natural product kindly provided by Prof. Raymond Andersen.

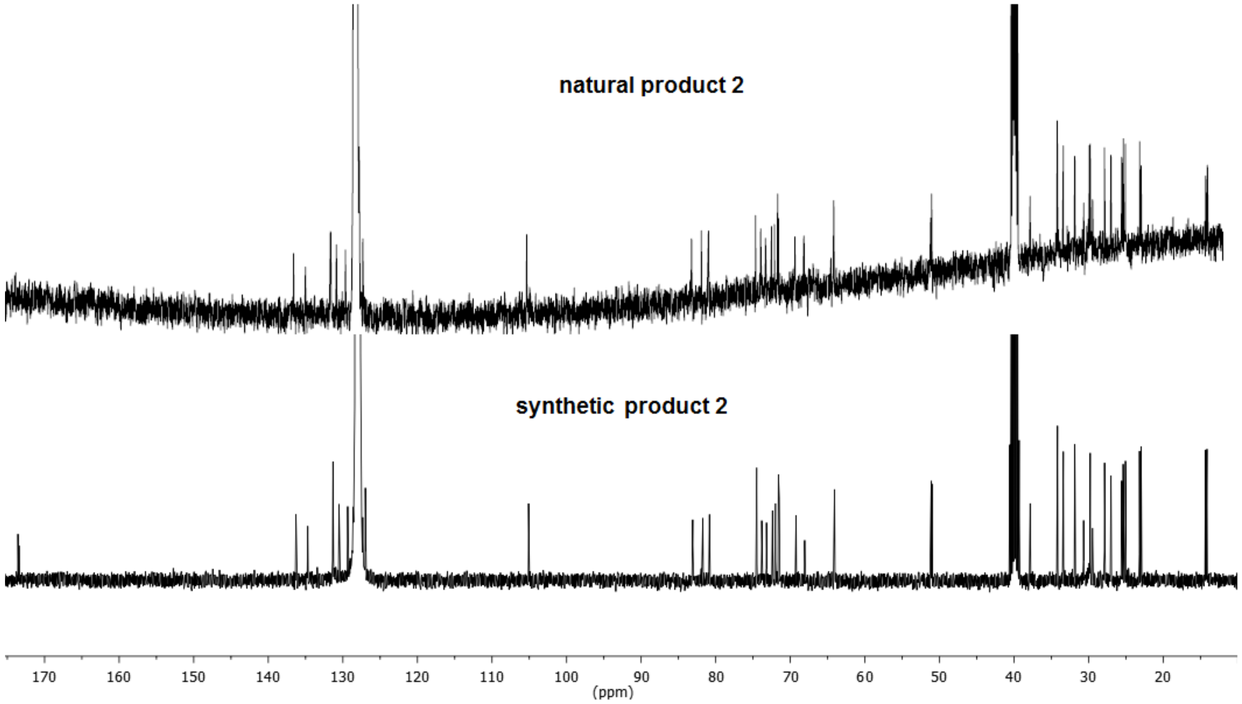

Figure S2. Comparison of synthetic nigricanoside A dimethyl ester **2** to natural product (^{13}C NMR). Spectrum of the natural product kindly provided by Prof. Raymond Andersen.

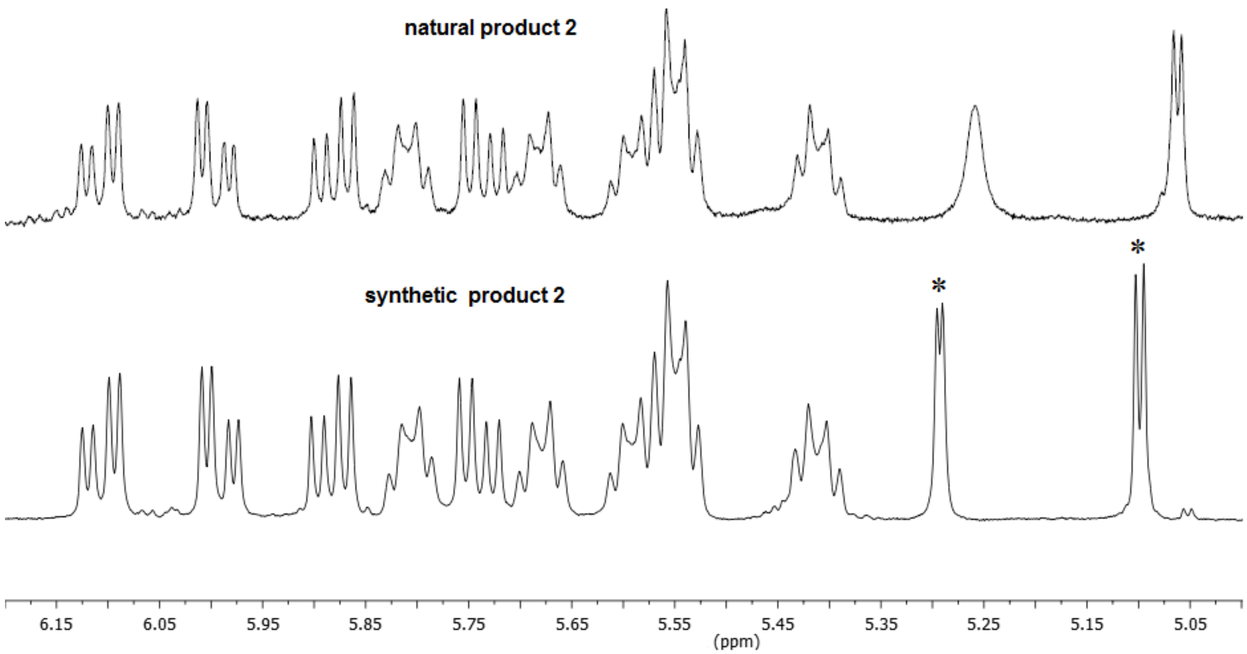

Figure S3. Comparison of synthetic nigricanoside A dimethyl ester **2** to natural product (6.20-5.00 ppm). Spectrum of the natural product kindly provided by Prof. Raymond Andersen. * = OH peak.

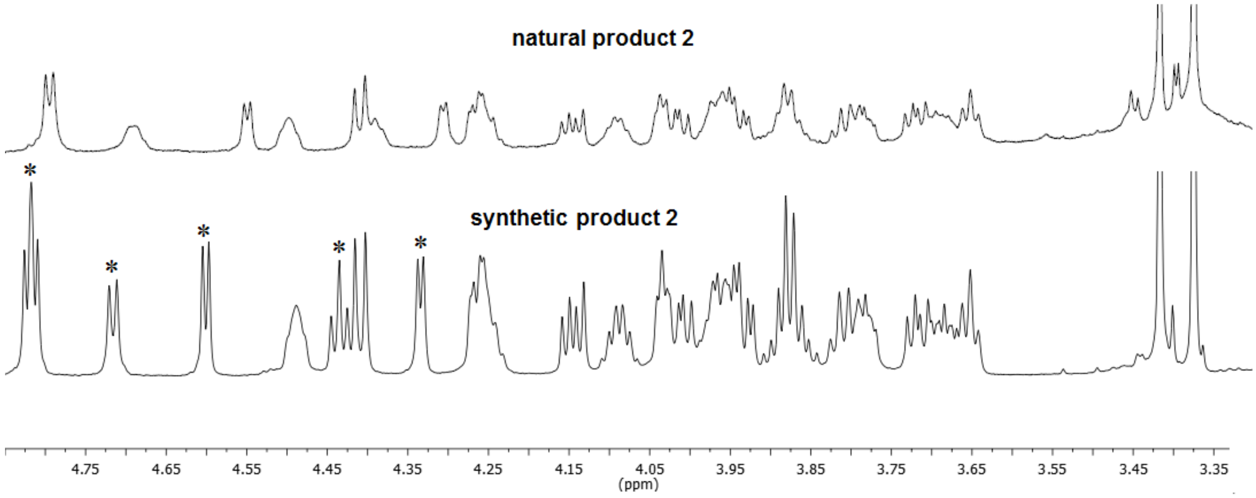

Figure S4. Comparison of synthetic nigricanoside A dimethyl ester **2** to natural product (4.85-3.30 ppm). Spectrum of the natural product kindly provided by Prof. Raymond Andersen. * = OH peak.

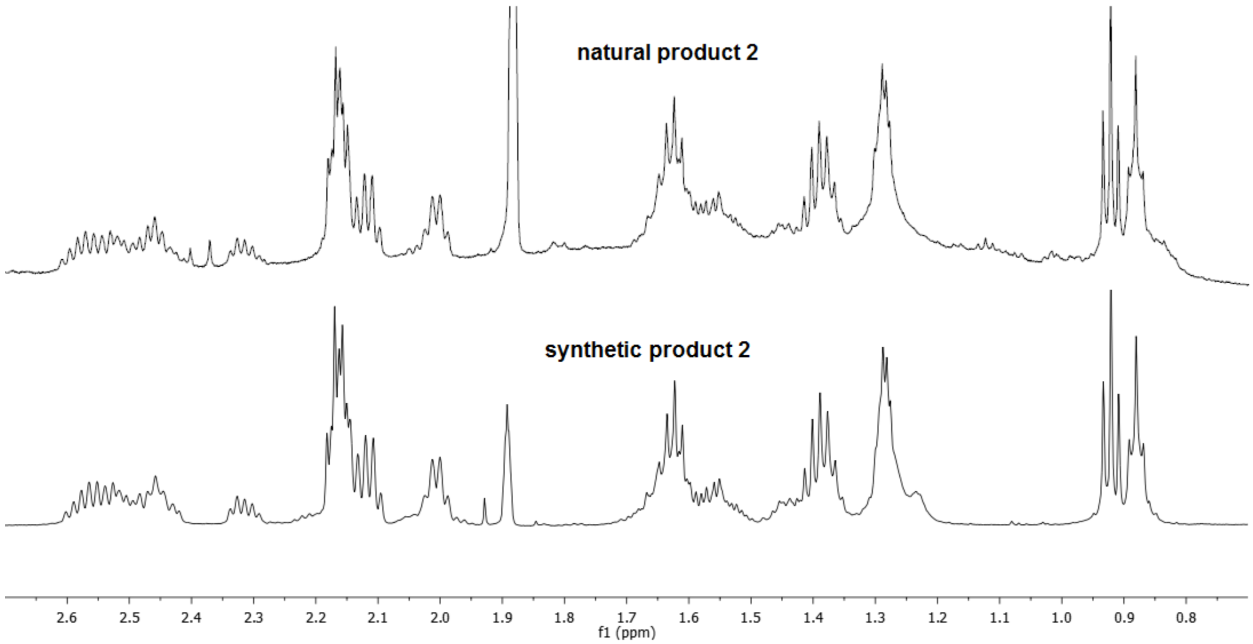

Figure S5. Comparison of synthetic nigricanoside A dimethyl ester **2** to natural product (2.70-0.70 ppm). Spectrum of the natural product kindly provided by Prof. Raymond Andersen.

Table S1. ^{1}H and ^{13}C NMR Data for synthetic Nigricanoside A Dimethyl Ester **2** in 25:2 C_6D_6/DMSO-d_6

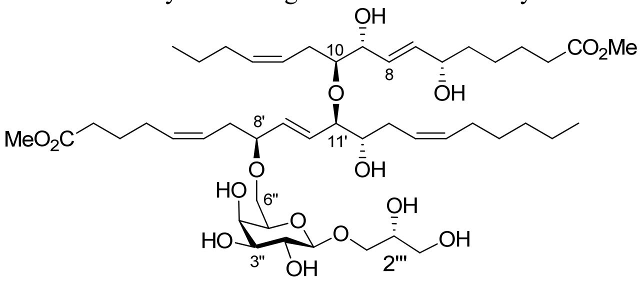

2

position	δ_C (synthetic product **2**)	δ_C (natural product)	δ_H, mult (*J* in Hz) (synthetic product **2**)	δ_H, mult (*J* in Hz) (natural product)
1	173.44, C	173.5		
2	34.17, CH_2	34.16	2.17<br>2.17	2.17<br>2.17
3	25.37, CH_2	25.36	1.62, m<br>1.62, m	1.62<br>1.62
4	25.57, CH_2	25.56	1.45, m<br>1.53, m	1.45, m<br>1.53, m
5	37.84, CH_2	37.84	1.56, m<br>1.65, m	1.56, m<br>1.65
6	71.48, CH	71.46	4.25, m	4.25
7	136.29, CH	136.29	5.99, dd (15.6, 5.8)	6.00, dd (15.5, 5.5)
8	129.35, CH	129.34	6.11, dd (15.5, 6.3)	6.11, dd (15.5, 6.4)
9	73.20, CH	73.17	4.49, m	4.50, m
10	81.73, CH	81.74	3.78, m	3.78, m
11	29.47, CH_2	29.45	2.45, m<br>2.58, m	2.45, m<br>2.58, m
12	127.51, CH	127.52	5.68, m	5.68, m
13	131.32, CH	131.32	5.55, m	5.55, m
14	29.88, CH_2	29.87	2.11, ddd (7.3, 7.3, 7.3)<br>2.11, ddd (7.3, 7.3, 7.3)	2.12, ddd (7.4, 7.4, 7.4)<br>2.12, ddd (7.4, 7.4, 7.4)
15	23.18, CH_2	23.16	1.40, qdd (7.4, 7.4, 7.4)<br>1.40, qdd (7.4, 7.4, 7.4)	1.40, qdd (7.4, 7.4, 7.4)<br>1.40, qdd (7.4, 7.4, 7.4)
16	14.13, CH_3	14.11	0.92, t (7.4)	0.92, t (7.4)
1-OCH_3	51.10, CH_3	51.10	3.42, s	3.41, s
6-OH			4.60, d (4.6)	4.55, d (4.5)
9-OH			5.10, d (4.7)	5.06, d (4.6)
1'	173.58, C	173.6		
2'	33.39, CH_2	33.38	2.16<br>2.16	2.16<br>2.16
3'	25.06, CH_2	25.05	1.62<br>1.62	1.62<br>1.62
4'	26.99, CH_2	26.98	2.01, ddd (7.4, 7.4, 7.4)	2.01, ddd (7.4, 7.4, 7.4)
5'	130.52, CH	130.52	5.41, m	5.41, m
6'	126.99, CH	126.99	5.59, m	5.59, m
7'	34.17, CH_2	34.16	2.31, m<br>2.48	2.32, m<br>2.48
8'	80.84, CH	80.84	3.81	3.80
9'	134.70, CH	134.70	5.74, dd (15.7, 7.5)	5.74, dd (15.7, 7.6)
10'	131.32, CH	131.34	5.88, dd (15.7, 7.4)	5.88, dd (15.7, 7.5)
11'	83.08, CH	83.08	4.26	4.26
12'	73.83, CH	73.84	3.96	3.96
13'	30.67, CH_2	30.66	2.53<br>2.53	2.53<br>2.53

14'	127.51, CH	127.55	5.81, m	5.81, m
15'	131.28, CH	131.27	5.55, m	5.55, m
16'	27.86, CH_2	27.85	2.15<br>2.15	2.15<br>2.15
17'	29.78, CH_2	29.77	1.29<br>1.38	1.29<br>1.38
18'	31.85, CH_2	31.84	1.29<br>1.29	1.29<br>1.29
19'	22.96, CH_2	22.95	1.29<br>1.29	1.29<br>1.29
20'	14.32, CH_3	14.31	0.88, bt (6.8)	0.88, bt (7.0)
1'-OCH_3	51.00, CH_3	51.0	3.38, s	3.37, s
12'-OH			4.82, d (5.5)	4.79, d (5.7)
1''	105.10, CH	105.11	4.41, d (7.7)	4.41, d (7.7)
2''	71.99, CH	71.98	3.95	3.95
3''	74.51, CH	74.50	3.68, m	3.69, m
4''	69.22, CH	69.22	4.03, m	4.03, m
5''	74.51, CH	74.51	3.65, bt (5.9)	3.65, bt (6.1)
6''	68.07, CH_2	68.06	3.72, dd (9.5, 6.0)<br>4.01, dd (9.5, 6.3)	3.72, dd (9.6, 6.1)<br>4.01, dd (9.6, 6.1)
2''-OH			5.29, d (3.2)	5.26, bs
3''-OH			4.72, d (5.6)	4.68, bs
4''-OH			4.33, d (4.1)	4.30, d (3.8)
1'''	72.39, CH_2	72.40	3.93, dd (10.3, 3.9)<br>4.15, dd (10.5, 5.6)	3.94, dd (10.4, 4.0)<br>4.14, dd (10.4, 5.6)
2'''	71.59, CH	71.58	4.08, m	4.09, m
3'''	64.07, CH_2	64.07	3.88, m<br>3.88, m	3.88, m<br>3.88, m
2'''-OH			4.81, d (5.6)	4.79, d (5.7)
3'''-OH			4.44, bt (6.0)	4.39, m

Methods and Materials

General: Unless otherwise stated, reactions were performed under argon using freshly purified solvents, which were purified using solvent purification columns purchased from Glass Contour, Laguna Beach, CA. All Reactions were monitored by thin-layer chromatography with E. Merck silica gel 60 F_{254} pre-coated plates (0.25 mm). Flash chromatography was performed with indicated solvents using silica gel (particle size 40-63 μm) purchased from Sorbent Technologies. ^{1}H and ^{13}C NMR spectra were recorded on Varian Inova- 400 MHz, 500 MHz or 600 MHz spectrometer. Chemical shift are reported relative to internal chloroform ($CDCl_3$: ^{1}H, δ = 7.26 ppm, ^{13}C, δ = 77.00 ppm), benzene (C_6D_6: ^{1}H, δ = 7.15 ppm, ^{13}C, δ = 128.00 ppm), methanol (CD_3OD: ^{1}H, δ = 3.31 ppm, ^{13}C, δ = 49.20 ppm) and Coupling constants are in Hz and are reported as d (doublet), t (triplet), q (quartet), and m (multiplet). Optical rotations were measured on a Rudolph Research Analytical Autopol® IV Polarimeter (50/60 Hz).Mass spectra were acquired on an Agilent Technologies 1200 series LC/MS using indicated ionization methods. Cytotoxicity measurements: HCT116 cells were plated in 96-well plates (1500, 5400, 1500 cells, respectively) in 10%RPMI + L-g+PenStrep (HCT116) or 5% RPMI + L-g media. DMSO or compound was added, at 5.5 mM to 0.85 nM in 3-fold dilutions, in duplicate. After 4 days at 37 ^{o}C, cell proliferation was measured using CellTiterGlow. IC_{50} data was calculated using Prism 6.

Materials. Chemicals were purchased from Aldrich, Fisher or Alfa Aesar, TCI, and used without purification.

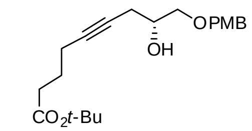

7

Glycidyl ether (*R*)-**6** was obtained from the reaction of commercially available (S)-glycidol (from Aldrich) with NaH (60% in mineral oil) and PMBCl in DMF following a known procedure[1].

To a stirred solution of 5-hexynoic acid tert-butyl ester **5**[2] (4.4 g, 26 mmol, 1.3 equiv) in 400 mL THF at -78˚C was added *n*-BuLi (9.6 mL, 24 mmol, 1.2 equiv). After stirring for 1 h at -78 ºC, $BF_3·OEt_2$ (2.5 mL, 20 mmol, 1.0 equiv) was added slowly to the stirred solution at this temperature. The solution was stirred for 20 minutes, at which time glycidyl ether (*R*)-**6** (3.88 g in 20 mL THF, 20 mmol, 1.0 equiv) was added dropwise at -78 ºC. The reaction was allowed to warm to room temperature and stirred overnight at this temperature. Then the reaction was quenched with saturated aqueous NH_4Cl, and extracted 3 times with Et_2O. The combined organic layer was washed with brine, dried with $MgSO_4$, filtered, concentrated, and purified by flash chromatography (5-25% EtOAc in hexanes) to afford the product **7** (5.39 g, 74% yield) as a colorless oil. ^{1}H NMR (400 MHz, $CDCl_3$) δ 7.26 (d, *J* = 7.7 Hz, 2H), 6.88 (d, *J* = 7.1 Hz, 2H), 4.49 (s, 2H), 3.90 (qd, *J* = 6.4, 4.0 Hz, 1H), 3.80 (s, 3H), 3.56 (dd, *J* = 9.6, 3.9 Hz, 1H), 3.45 (dd, *J* = 9.1, 7.1 Hz, 1H), 2.43–2.35 (m, 2H), 2.30 (t, *J* = 7.4 Hz, 2H), 2.23–2.16 (m, 2H), 1.80–1.69 (m, 2H), 1.44 (s, 9H); ^{13}C NMR (100 MHz, $CDCl_3$) δ 172.58, 159.26, 129.97, 129.38, 113.79, 81.62, 80.25, 76.37, 73.05, 72.69, 69.09, 55.25, 34.42, 28.09, 24.24, 23.86, 18.16; $[\alpha]_D^{20}$ = -6.8 (*c* = 1.0, $CHCl_3$); MS(ESI) calcd for $C_{21}H_{30}O_5Na$ $(M+Na)^+$ 385.2, found 385.1; HRMS(ESI) calcd for $C_{21}H_{30}O_5Na$ $(M+Na)^+$ 385.1991, found 385.1982.

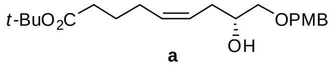

a

A stirred mixture of alcohol **7** (3.3 g, 9.1 mmol, 1.0 equiv), quinoline (32 μL, 0.27 mmol, 3 mol%), and Lindlar catalyst (~5% palladium on calcium carbonate; poisoned with lead) (0.388 g, 0.18 mmol, 2 mol %) in EtOAc (50 mL) was sparged with N_2 for 15 minutes. H_2 gas was then bubbled through the stirred mixture for 1 h. The reaction was filtered through a plug of Celite, and purified by flash chromatography (5-25% EtOAc in hexanes) to afford the product **a** (3.2 g, 96% yield) as a colorless oil. ^{1}H NMR (400 MHz, $CDCl_3$) δ 7.25 (d, *J* = 8.6 Hz, 2H), 6.88 (d, *J* = 8.6 Hz, 2H), 5.54–5.34 (m, 2H), 4.47 (s, 2H), 3.87–3.80 (m, 1H), 3.80 (s, 3H), 3.48 (dd, *J* = 9.5, 3.4 Hz, 1H), 3.33 (dd, *J* = 9.4, 7.4 Hz, 1H), 2.43 (s, 1H), 2.28–2.16 (m, 4H), 2.12–2.01 (m, 2H), 1.71–1.56 (m, 2H), 1.43 (s, 9H); ^{13}C NMR (100 MHz, $CDCl_3$) δ 173.01, 159.23, 131.57, 129.99, 129.37, 125.59, 113.78, 80.05, 73.63, 73.00, 70.20, 55.23, 34.92, 31.27, 28.06, 26.56, 24.85; $[\alpha]_D^{20}$ = +1.4 (*c* = 1.0, $CHCl_3$); MS(ESI) calcd for $C_{21}H_{32}O_5Na$ $(M+Na)^+$ 387.2, found 387.2; HRMS(ESI) calcd for $C_{21}H_{32}O_5Na$ $(M+Na)^+$ 387.2147, found 387.2149.

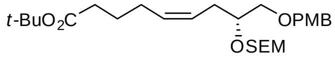

b

To a stirred solution of alcohol **a** (5.95 g, 16 mmol, 1.0 equiv) in 160 mL DCM at 0 ºC was added *i*-Pr_2NEt (5.7 mL, 33 mmol, 2.0 equiv). Then SEMCl (2-(Trimethylsilyl)ethoxymethyl chloride) (4.4 mL, 25 mmol, 1.5equiv) was added slowly to the stirred solution at this temperature. The reaction was allowed to warm to room temperature and stirred overnight. Then the reaction was quenched with H_2O, and extracted 3 times with DCM. The combined organic layer was washed with brine, dried with $MgSO_4$, filtered, concentrated, and purified by flash chromatography (5-10% EtOAc in hexanes) to afford the product **b** (7.79 g, 96% yield) as a colorless oil. ^{1}H NMR (400 MHz, $CDCl_3$) δ 7.26–7.21 (m, 2H), 6.89–6.83 (m, 2H), 5.49–5.35 (m, 2H), 4.74 (dd, *J* = 18.3, 7.0 Hz, 2H), 4.53–4.40 (m, 2H), 3.83–3.74 (m, 1H), 3.80 (s, 3H), 3.69–3.56 (m, 2H), 3.45 (d, *J* = 5.1 Hz, 2H), 2.35–2.27 (m, 2H), 2.24–2.16 (m, 2H), 2.11–2.00 (m, 2H), 1.70–1.57 (m, 2H), 1.43 (s, 9H), 0.94–0.83 (m, 2H), -0.00 (s, 9H); ^{13}C NMR (100 MHz, $CDCl_3$) δ 172.99, 159.08, 131.04, 130.35, 129.16, 125.96, 113.69, 93.98, 79.99, 75.59, 72.93, 71.85, 65.02, 55.23, 35.01, 29.83, 28.09, 26.69, 24.90, 18.03, -1.45; $[\alpha]_D^{20}$ = +3.8 (*c* = 1.0, $CHCl_3$); MS(ESI) calcd for $C_{27}H_{50}NO_6Si$ $(M+NH_4)^+$ 512.3, found 512.3; HRMS(ESI) calcd for $C_{27}H_{46}O_6SiNa$ $(M+Na)^+$ 517.2961, found 517.2969.

[1] a) Dias, L. C.; Ferreira, M. A. B. *J. Org. Chem.* **2012**, *77*, 4046. b) White, J. D.; Lincoln, C. M.; Yang, J.; Martin, W. H. C.; Chan, D. B. *J. Org. Chem.* **2008**, *73*, 4139.

[2] McGinley, C. M.; Jacquot, C.; van der Donk, W. A. *Bioorg. Med. Chem. Lett.* **2007**, *17*, 4049.

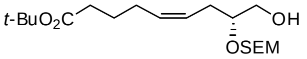

c

DDQ (2.64 g, 11.6 mmol, 1.5 equiv) was added to a biphasic solution of PMB ether **b** (137 mg, 0.10 mmol) in 50 mL DCM and 2.5 mL phosphate buffer (pH = 7) at 0 ºC . The resulting solution was stirred for 1 h at 0 ºC, then warmed to room temperature for additional 1.5 h. Then the reaction was quenched with saturated aqueous $NaHCO_3$ solution and extracted 3 times with DCM. The combined organic layer was washed with brine, dried with $MgSO_4$, filtered, concentrated, and purified by flash chromatography (5-25% EtOAc in hexanes) to afford the product **c** (2.81g, 97% yield) as a colorless oil. ^{1}H NMR (400 MHz, $CDCl_3$) δ 5.53–5.32 (m, 2H), 4.79 (d, *J* = 7.1 Hz, 1H), 4.70 (d, *J* = 7.2 Hz, 1H), 3.81–3.69 (m, 1H), 3.64–3.53 (m, 3H), 3.53–3.43 (m, 1H), 3.24 (s, 1H), 2.32–2.15 (m, 4H), 2.11–1.98 (m, 2H), 1.69–1.57 (m, 2H), 1.43 (s, 9H), 1.03–0.87 (m, 2H), 0.01 (s, 9H); ^{13}C NMR (100 MHz, $CDCl_3$) δ 172.98, 131.32, 125.52, 95.07, 81.74, 80.08, 65.69, 65.28, 34.94, 29.69, 28.09, 26.66, 24.83, 18.10, -1.49; $[\alpha]_D^{20}$ = -27.6 (*c* = 1.0, $CHCl_3$); MS(ESI) calcd for $C_{19}H_{39}O_5Si$ $(M+H)^+$ 375.3, found 375.2; HRMS(ESI) calcd for $C_{19}H_{38}O_5SiNa$ $(M+Na)^+$ 397.2386, found 397.2382.

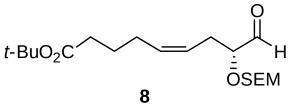

8

$NaHCO_3$ (6.24 g, 74.2 mmol, 5.0 equiv) was added to the solution of the alcohol **c** (5.56 g, 14.8 mmol, 1.0 equiv) in 150 mL DCM at 0 ºC. Dess-Martin periodinane (7.56 g, 17.8 mmol, 1.2 equiv) was added to the stirred suspension in 3 portions. After stirring overnight at this temperature, the reaction was concentrated. Then the reaction was quenched with a solution of 20% aqueous $Na_2S_2O_3$/saturated aqueous $NaHCO_3$ (1:1), and extracted three times with EtOAc, The combined organic layer was washed with brine, and dried with $MgSO_4$, concentrated, filtered, and purified by flash chromatography (5-10% EtOAc in hexanes) to afford the product **8** (4.95 g, 90% yield) as a colorless oil. ^{1}H NMR (400 MHz, $CDCl_3$) δ 9.61 (d, *J* = 1.9 Hz, 1H), 5.55–5.33 (m, 2H), 4.74 (dd, *J* = 19.6, 7.0 Hz, 2H), 3.93 (td, *J* = 6.5, 1.8 Hz, 1H), 3.75–3.57 (m, 2H), 2.43 (t, *J* = 6.5 Hz, 2H), 2.19 (t, *J* = 7.5 Hz, 2H), 2.13–1.97 (m, 2H), 1.70–1.56 (m, 2H), 1.42 (s, 9H), 0.97–0.79 (m, 2H), 0.00 (s, 9H); ^{13}C NMR (100 MHz, $CDCl_3$) δ 202.52, 172.86, 132.37, 123.77, 94.80, 81.65, 80.05, 65.76, 34.86, 28.15, 28.05, 26.62, 24.70, 17.91, -1.49; $[\alpha]_D^{20}$ = +14.6 (*c* = 1.0, $CHCl_3$); MS(ESI) calcd for $C_{19}H_{36}O_5SiNa$ $(M+Na)^+$ 395.2, found 395.2; HRMS(ESI) calcd for $C_{19}H_{36}O_5SiNa$ $(M+Na)^+$ 395.2230, found 395.2217.

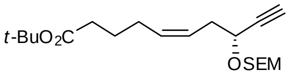

10

A solution of MeONa (5.4 M in MeOH, 2.6 mL, 4.0 equiv) in 70 mL THF was cooled to -78 ºC. Dimethyl 1-diazo-2-oxopropylphosphonate **9**[3] (2.69 g, 14.0 mmol, 4.0 equiv) in 70 mL THF was added to the above solution at -78 ºC. Afer 15 minutes, the aldehyde (1.30 g, 3.5 mmol, 1.0 equiv) was added to the stirred solution at -78 ºC. After stirring for 0.5 h at this temperature, the reaction was quenched with saturated aqueous NH_4Cl at 0 ºC, and extracted 3 times with EtOAc. The combined organic layer was washed with brine, dried with $MgSO_4$, concentrated, filtered, and purified by flash chromatography (5-10% EtOAc in hexanes) to afford the product **10** (1.21 g, 94% yield) as a colorless oil. ^{1}H NMR (400 MHz, $CDCl_3$) δ 5.56–5.32 (m, 2H), 4.87 (d, *J* = 7.0 Hz, 1H), 4.63 (d, *J* = 7.0 Hz, 1H), 4.30 (td, *J* = 6.5, 2.0 Hz, 1H), 3.71–3.61 (m, 1H), 3.55–3.46 (m, 1H), 2.44 (t, *J* = 5.9 Hz, 2H), 2.37 (d, *J* = 2.0 Hz, 1H), 2.17 (t, *J* = 7.5 Hz, 2H), 2.10–2.00 (m, 2H), 1.70–1.53 (m, 2H), 1.40 (s, 9H), 0.96–0.81 (m, 2H), -0.03 (s, 9H); ^{13}C NMR (100 MHz, $CDCl_3$) δ 172.77, 131.79, 124.72, 92.25, 82.26, 79.85, 73.45, 65.30, 64.84, 34.83, 33.42, 28.01, 26.69, 24.75, 17.93, -1.52; $[\alpha]_D^{20}$ = +87.2 (*c* = 1.0, $CHCl_3$); MS(ESI) calcd for $C_{20}H_{36}O_4SiNa$ $(M+Na)^+$ 391.2, found 391.2; HRMS(ESI) calcd for $C_{20}H_{36}O_4SiNa$ $(M+Na)^+$ 391.2281, found 391.2280.

The (*R*) and (*S*) Mosher's esters were synthesized from deprotecting SEM product of **10** and the (*S*) and (*R*) acid chloride, respectively (pyridine, DMAP, CH_2Cl_2, rt, 2 h). The resulting differences in chemical shifts are consistent with (*R*) stereochemistry at C8' as shown.

---

[3] Wijtmans, M.; de Graaf, C.; de Kloe, G.; Istyastono, E. P.; Smit, J., Lim, H.; Boonnak, R.; Nijmeijer, S.; Smits, R. A.; Jongejan, A.; Zuiderveld, O.; de Esch, I. J. P.; Leurs, R. *J. Med. Chem.* **2011**, *54*, 1693.

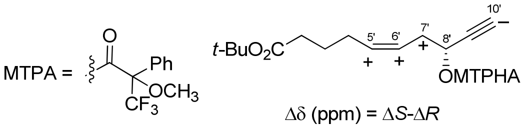

	S	R	Δδ (ppm)
H5'	5.4310	5.3270	+0.1040
H6'	5.5720	5.4880	+0.0840
H7'	2.6290	2.5490	+0.0800
H8'	5.5075	5.5360	-0.0285
H10'	2.5015	2.5760	-0.0745

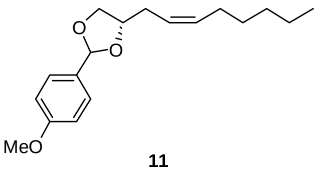

11

Glycidyl ether (*S*)-**6** was obtained from the reaction of commercially available (R)-glycidol (from Aldrich) with NaH (60% in mineroil oil) and PMBCl in DMF following a known procedure[4].

To a stirred solution of 1-heptyne (7.2 mL, 54.5 mmol, 2.0 equiv) in 150 mL THF at -78 ºC was added *n*-BuLi (22 mL, 54.5 mmol, 2.0 equiv). After stirring for 1 h at -78 ºC, $BF_3 \cdot OEt_2$ (5.2 mL, 40.9 mmol, 1.5 equiv) was added at this temperature. The solution was stirred for 15 minutes, at which time glycidyl ether (5.29 g in 20 mL THF, 27.3 mmol, 1.0 equiv) was added dropwise at -78 ºC. The reaction was stirred at - 78 ºC for 4 hours, quenched with saturated aqueous NH_4Cl, and extracted 3 times with Et_2O. The combined organic layer was washed with brine, dried with $MgSO_4$, filtered, and concentrated to afford the product as a yellow oil. The ^{1}HNMR spectrum of the crude sample was very clean and so the above alcohol was brought into the following reaction without further purification. ^{1}H NMR (400 MHz, $CDCl_3$) δ 7.29–7.18 (m, 2H), 6.91–6.79 (m, 2H), 4.48 (s, 2H), 3.94–3.83 (m, 1H), 3.79 (s, 3H), 3.55 (dd, *J* = 9.5, 3.9 Hz, 1H), 3.44 (dd, *J* = 9.5, 6.7 Hz, 1H), 2.42–2.35 (m, 2H), 2.16–2.06 (m, 2H), 1.87–1.78 (m, 2H), 1.38–1.21 (m, 4H), 0.87 (t, *J* = 7.1 Hz, 3H).

A stirred mixture of the above alcohol (3.29 g, 11.3 mmol, 1.0 equiv), quinoline (40 µL, 0.34 mmol, 3 mol%), and Lindlar catalyst (~5% palladium on calcium carbonate; poisoned with lead) (481.6 g, 0.11 mmol, 1.0 mol %) in 120 mL EtOAc was sparged with N_2 for 15 minutes. H_2 gas was then bubbled through the stirred mixture for 1 hour. The reaction mixture was filtered through a plug of Celite and concentrated to afford the desired alkene as a yellow oil. The ^{1}HNMR spectrum of crude alkene was clean and therefore was used in the following reaction without further purification. ^{1}H NMR (400 MHz, $CDCl_3$) δ 7.29–7.21 (m, 2H), 6.90–6.83 (m, 2H), 5.55–5.43 (m, 1H), 5.42–5.32 (m, 1H), 4.47 (s, 2H), 3.85–3.76 (m, 1H), 3.79 (s, 3H), 3.48 (dd, *J* = 9.5, 3.2 Hz, 1H), 3.32 (dd, *J* = 9.4, 7.5 Hz, 1H), 2.29 (d, *J* = 3.5 Hz, 1H), 2.27–2.17(m, 2H), 2.08– 1.94 (m, 2H), 1.37–1.16 (m, 6H), 0.87 (t, *J* = 6.8 Hz, 3H).

The alkene (5.6 g, 19.2 mmol, 1.0 equiv) was dissolved in 300 mL DCM. 4Å MS (8.4 g) and DDQ (2,3-dichloro-5,6-dicyano-1,4-benzoquinone) (4.88 g, 21.1 mmol, 1.1 equiv) were added at 0 ºC. The resulting solution was allowed to stir for 10 min at 0 ºC, then warmed to room temperature and stirred for additional 2 hours. The reaction mixture was filtered, and the filtrate was washed with saturated aqueous $NaHCO_3$. The organic layer was separated and the aqueous layer was extracted 3 times with DCM. The combined organic layer was washed with brine, dried with $MgSO_4$, filtered, concentrated, and purified by flash chromatography (3-25% EtOAc in hexanes) to afford the product **11** (3.7 g, 67% yield over three steps) as a mixture of diasteroisomers. ^{1}H NMR (400 MHz, $CDCl_3$) δ 7.47–7.36 (m, 2H), 6.93–6.87 (m, 2H), 5.90, 5.76 (s, 1H), 5.61–5.49 (m, 1H), 5.46–5.34 (m, 1H), 4.32–4.17, 4.10–4.03 (m, 2H), 3.81, 3.80 (s, 3H), 3.77–3.71, 3.68–3.62 (m, 1H), 2.59–2.46 (m, 1H), 2.46–2.27 (m, 1H), 2.14–1.94 (m, 2H), 1.41–1.18 (m, 6H), 0.94–0.80 (m, 3H).

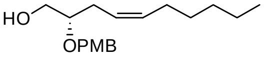

d

To a stirred solution of the compound **11** (3.7 g, 12.7 mmol, 1.0 equiv) in 125 mL DCM at -78 ºC was added DIBAL-H (diisobutylaluminium hydride, 1.0 M in DCM) (32 mL, 31.75 mmol, 2.5 equiv). After stirring for 0.5 hour at -78 ºC, the reaction was quenched with 10% citric acid, and the heterogeneous mixture was stirred for 1 hour at room temperature until two clear phases were present. The organic layer was separated and the aqueous layer was extracted 3 times with DCM. The combined organic layer was washed with brine, dried with $MgSO_4$, filtered, concentrated, and purified by flash chromatography (3-25% EtOAc in hexanes) to afford the product **d** (2.93 g, 79% yield) as a colorless oil. ^{1}H NMR (400 MHz, $CDCl_3$) δ 7.28 (d, *J* = 8.5 Hz, 2H), 6.89 (d, *J* = 8.5 Hz, 2H), 5.57–5.44

[4] Nicolaou, K. C.; Li, Y.; Fylaktakidou, K. C.; Mitchell, H. J.; Wei, H.; Weyershausen, B. *Angew. Chem. Int. Ed.* **2001**, *40*, 3849.

(m, 1H), 5.43–5.31 (m, 1H), 4.62 (d, J = 11.2 Hz, 1H), 4.47 (d, J = 11.4 Hz, 1H), 3.81 (s, 3H), 3.69–3.61 (m, 1H), 3.56–3.46 (m, 2H), 2.44–2.32 (m, 1H), 2.32–2.22 (m, 1H), 2.09–1.98 (m, 2H), 1.88 (s, 1H), 1.42–1.17 (m, 6H), 0.89 (t, J = 6.8 Hz, 3H); ^{13}C NMR (100 MHz, $CDCl_3$) δ 159.22, 132.62, 130.39, 129.34, 124.25, 113.81, 79.21, 71.14, 64.14, 55.20, 31.47, 29.17, 28.65, 27.28, 22.51, 14.01; $[\alpha]_D^{20}$ = +33.2 (c = 1.0, $CHCl_3$); MS(ESI) calcd for $C_{18}H_{28}O_3Na$ $(M+Na)^+$ 315.2, found 315.2; HRMS(ESI) calcd for $C_{18}H_{28}O_3Na$ $(M+Na)^+$ 315.1936, found 315.1924.

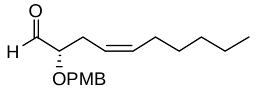

12

Pyridine (4.0 mL, 50.0 mmol, 5.0 equiv) was added to the solution of the alcohol **d** (2.93 g, 10.0 mmol, 1.0 equiv) in 70 mL DCM at 0 ºC. Dess-Martin periodinane (5.2 g, 13.0 mmol, 1.2 equiv) was added to the stirred suspension in 2 portions. After stirring overnight at this temperature, the reaction was concentrated. Then the reaction was quenched with a solution of 20% aqueous $Na_2S_2O_3$/saturated aqueous $NaHCO_3$ (1:1), and extracted 3 times with EtOAc. The combined organic layer was washed with brine, dried with $MgSO_4$, concentrated, filtered, and purified by flash chromatography (5-10% EtOAc in hexanes) to afford the product **12** (2.4 g, 82% yield) as a colorless oil. ^{1}H NMR (400 MHz, $CDCl_3$) δ 9.62 (d, J = 2.1 Hz, 1H), 7.27 (d, J = 5.6 Hz, 2H), 6.88 (d, J = 8.6 Hz, 2H), 5.61–5.46 (m, 1H), 5.44–5.34 (m, 1H), 4.56 (q, J = 11.5 Hz, 2H), 3.80 (s, 3H), 3.76 (td, J = 6.5, 2.1 Hz, 1H), 2.46 (t, J = 6.8 Hz, 2H), 2.08–1.96 (m, 2H), 1.43–1.18 (m, 6H), 0.88 (t, J = 6.9 Hz, 3H); ^{13}C NMR (100 MHz, $CDCl_3$) δ 203.40, 159.47, 133.51, 129.63, 129.30, 122.60, 113.86, 82.84, 72.20, 55.23, 31.44, 29.07, 28.32, 27.32, 22.50, 14.01; $[\alpha]_D^{20}$ = -22.6 (c = 1.0, $CHCl_3$); MS(ESI) calcd for $C_{18}H_{26}O_3Na$ $(M+Na)^+$ 313.2, found 313.2; HRMS(ESI) calcd for $C_{18}H_{26}O_3Na$ $(M+Na)^+$ 313.1780, found 313.1772.

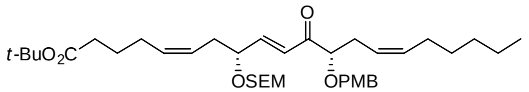

e

$Cp_2Zr(H)Cl$ (1.47 g, 5.7 mmol, 1.5 equiv) was weighed and added to a flask in a glove box, suspended in 14 mL DCM and cooled to 0 ºC. A solution of alkyne **10** (1.54 g, 4.2 mmol, 1.1 equiv) in 14 mL DCM was added to the stirred suspension of $Cp_2Zr(H)Cl$ at 0 ºC. Hydrozirconation was complete after 1.5 hours, after which the solution was cooled to -78 ºC. At this time, a solution of Me_2Zn (2.0 M in toluene, 2.84 mL, 5.7 mmol, 2.0 equiv) was added slowly to the solution of vinyl zirconium at -78 ºC. After stirring for 15 minutes at -78 ºC, the reaction was allowed to warm to 0 ºC. The solution was then allowed to stir for additional 15 minutes at 0 ºC, after which a solution of the aldehyde **12** (1.10 g, 3.8 mmol, 1.0 equiv) in 7.0 mL toluene was added at the same temperature. The reaction was allowed to stir overnight at 0 ºC. The reaction was quenched with a 10% aqueous solution of citric acid, and the heterogeneous mixture was stirred for 1 h at room temperature until two clear phases were present. The organic layer was separated and the aqueous layer was extracted 3 times with DCM. The combined organic portion was washed with brine, dried with $MgSO_4$, filtered, concentrated, and purified by flash chromatography (3-25% EtOAc in hexanes) to afford the desired alcohol (2.45 g) as a mixture of diastereoisomers.

$NaHCO_3$ (1.55 g, 18.5 mmol, 5.0 equiv) was added to the solution of the above alcohol (2.44 g, 3.7 mmol, 1.0 equiv) in 15 mL DCM at 0 ºC. Dess-Martin periodinane (1.88 g, 4.4 mmol, 1.2 equiv) was added to the stirred suspension in 2 portions. After stirring overnight at this temperature, the reaction was concentrated. Then the reaction was quenched with a solution of 20% aqueous $Na_2S_2O_3$/saturated aqueous $NaHCO_3$ (1:1), and extracted three times with EtOAc. The combined organic layer was washed with brine, dried with $MgSO_4$, concentrated, filtered, and purified by flash chromatography (5-15% EtOAc in hexanes) to afford the product **e** (2.07 g, 83% yield over two steps) as a colorless oil. ^{1}H NMR (400 MHz, $CDCl_3$) δ 7.25–7.19 (m, 2H), 6.91–6.80 (m, 3H), 6.65 (dd, J = 15.8, 1.1 Hz, 1H), 5.54–5.30 (m, 4H), 4.65 (d, J = 7.0 Hz, 1H), 4.60 (d, J = 7.0 Hz, 1H), 4.48 (d, J = 11.4 Hz, 1H), 4.34 (d, J = 11.4 Hz, 1H), 4.30–4.23 (m, 1H), 3.94–3.87 (m, 1H), 3.79 (s, 3H), 3.76–3.65 (m, 1H), 3.58–3.46 (m, 1H), 2.52–2.24 (m, 4H), 2.19 (t, J = 7.5 Hz, 2H), 2.09–2.00 (m, 2H), 2.00–1.93 (m, 2H), 1.70–1.55 (m, 2H), 1.43 (s, 9H), 1.35–1.17 (m, 6H), 0.96–0.75 (m, 5H), 0.01 (s, 9H); ^{13}C NMR (100 MHz, $CDCl_3$) δ 200.60, 172.82, 159.30, 146.92, 133.04, 131.73, 129.52, 129.42, 124.87, 124.67, 123.28, 113.74, 92.78, 83.68, 79.99, 75.02, 71.78, 65.30, 55.20, 34.91, 32.72, 31.44, 30.37, 29.12, 28.07, 27.32, 26.73, 24.78, 22.51, 17.97, 14.02, -1.45; $[\alpha]_D^{20}$ = +14.6 (c = 0.5, $CHCl_3$); MS(ESI) calcd for $C_{38}H_{62}O_7SiNa$ $(M+Na)^+$ 681.4, found 681.4; HRMS(ESI) calcd for $C_{38}H_{62}O_7SiNa$ $(M+Na)^+$ 681.4163, found 681.4161.

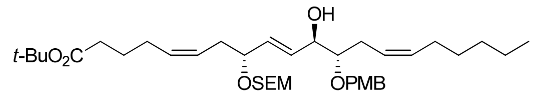

13

Ketone **e** (2.07 g, 3.14 mmol, 1.0 equiv) was dissolved in 60 mL DCM and cooled to -78 ºC. Super-Hydride (1.0 M in THF, 4.1 mL, 4.08 mmol, 1.3 equiv) was added slowly and the resulting solution was allowed to stir for 30 minutes at -78 ºC. Then the reaction was quenched with a 10% aqueous solution of citric acid, and the heterogeneous mixture was stirred for 1 hour at room temperature until two clear phases were present. The organic layer was separated and the aqueous layer was extracted 3 times with DCM. The combined organic layer was washed with brine, dried with $MgSO_4$, filtered, concentrated, and purified by flash chromatography (3-25% EtOAc in hexanes) to afford the desired alcohol **13** (> 10:1 selectivity, 2.06 g, 97 % yield) as a colorless oil. ^{1}H NMR (400 MHz, $CDCl_3$) δ 7.29–7.19 (m, 2H), 6.92–6.82 (m, 2H), 5.73 (dd, *J* = 15.6, 6.0 Hz, 1H), 5.60 (ddd, *J* = 15.6, 7.4, 0.9 Hz, 1H), 5.50–5.36 (m, 4H), 4.67 (d, *J* = 6.9 Hz, 1H), 4.59 (d, *J* = 7.2 Hz, 1H), 4.57 (d, *J* = 11.2 Hz, 1H), 4.50 (d, *J* = 11.3 Hz, 1H), 4.29–4.22 (m, 1H), 4.13–4.04 (m, 1H), 3.80 (s, 3H), 3.78–3.69 (m, 1H), 3.54–3.47 (m, 1H), 3.47–3.40 (m, 1H), 2.43–2.23 (m, 4H), 2.14–2.13 (m, 3H), 2.11–1.95 (m, 4H), 1.70–1.56 (m, 2H), 1.43 (s, 9H), 1.37–1.20 (m, 6H), 0.98–0.81 (m, 5H), 0.01 (s, 9H); ^{13}C NMR (100 MHz, $CDCl_3$) δ 173.02, 159.21, 132.20, 131.97, 131.59, 130.87, 130.41, 129.33, 125.86, 125.22, 113.77, 91.82, 81.82, 80.02, 75.67, 72.70, 71.79, 65.01, 55.24, 34.99, 33.44, 31.52, 29.25, 28.09, 27.71, 27.40, 26.76, 24.87, 22.57, 18.05, 14.05, -1.42; $[\alpha]_D^{20}$ = +59.0 (*c* = 1.0, $CHCl_3$); MS(ESI) calcd for $C_{38}H_{64}O_7SiNa$ $(M+Na)^+$ 683.4, found 683.4; HRMS(ESI) calcd for $C_{38}H_{64}O_7SiNa$ $(M+Na)^+$ 683.4319, found 683.4314.

The (*R*) and (*S*) Mosher's esters were synthesized from **13** and the (*S*) and (*R*) acid chloride, respectively (pyridine, DMAP, CH_2Cl_2, rt, 24 h). The resulting differences in chemical shifts are consistent with (*R*) stereochemistry at C11' as shown.

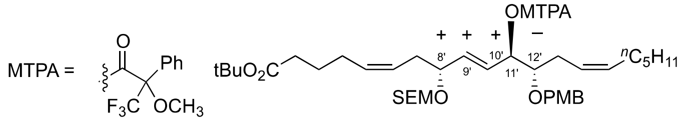

Δδ (ppm) = Δ*S*-Δ*R*

	S	*R*	Δδ (ppm)
H8'	4.0946	4.0394	+0.055
H9'	5.7424	5.5738	+0.169
H10'	5.7925	5.6394	+0.153
H11'	5.6471	5.7502	-0.103
H12'	3.4719	3.5097	-0.038

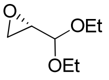

(*S*)-**A**

(*S*)-Glycidaldehyde diethyl acetal (*S*)-**A**[5], was resolved from racemic glycidaldehyde diethyl acetal using the HKR method developed by Jacobsen[6].

p-Toluenesulfonic acid (0.8 g, 1.33 mmol, 1.4 mol%) was added to a 100 mL round-bottomed flask containing a stirred solution of (*R*, *R*)-(-)-*N,N'*-bis(3,5-di-*tert*-butylsalicylidene)-1,2-cyclohexanediaminocobalt (II) (0.27 g, 1.4 mmol, 1.5 mol%) in 20 mL DCM at room temperature. The reaction was allowed to stir open to air for 1 hour, after which the stir bar was removed and the solution was concentrated by rotary evaporation. The contents of the flask were placed under high vacuum for 1 hour to remove the remaining solvent. The stir bar was returned to the flask and racemic glycidaldehyde diethyl acetal (13.7 g, 93.7 mmol, 1.0 equiv) was added to the (salen)Co^{III}-OTs catalyst at room temperature. The flask was capped and stirred vigorously for 24 hours. The epoxide was transferred to a -78˚C receiving flask by distillation (60˚C, 10 mmHg) to afford (*S*)-**A** (5.81 g, 40.1 mmol, 43% yield, 97% ee) as a clear liquid, which was stored over 4Å molecular sieves in the freezer. The ee of (*S*)-**A** (97%) was determined by chiral GC. ^{1}H NMR (400 MHz, $CDCl_3$) δ = 4.34 (dd, *J* = 4.3, 1.5 Hz, 1H), 3.78–3.72 (m, 2H), 3.65–3.61 (m, 2H), 3.16–3.08 (m, 1H), 2.82–2.75 (m, 2H), 1.28–1.20 (m, 6H); ^{13}C NMR (100 MHz, $CDCl_3$) δ 101.5, 62.9, 62.3, 51.8, 43.8, 15.2, 15.1; $[\alpha]_D^{20}$ = -5.4 (*c* = 1.0, EtOH); lit. data: $[\alpha]_D^{25}$ = -5.4 (*c* = 1.04, EtOH)[7].

---

[5] a) Weisblat, D. I.; Magerlein, B. J.; Myers, D. R.; Hanze, A. R.; Fairburn, E. I.; Rolfson, S. T. *J. Am. Chem. Soc.* **1953,** *75*, 5893. b) Franck, C. P. D.; Virgil H.; Soth, S.; Laurence, H. *Eur. J. Org. Chem.* **2006**, *2006*, 5526.

[6] Nielsen, L. P. C.; Stevenson, C. P.; Blackmond, D. G.; Jacobsen, E. N. *J. Am. Chem. Soc.* **2004**, *126*, 1360.

[7] Pederson, R. L.; Liu, K. K. C.; Rutan, J. F.; Chen, L.; Wong, C. H. *J. Org. Chem.* **1990**, *55*, 4897.

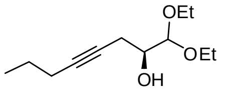

B

To a stirred solution of 1-pentyne (3.0 mL, 30 mmol, 2.0 equiv) in 100 mL THF at -78˚C was added *n*-BuLi (12 mL, 30 mmol, 2.0 equiv). After stirring for 1 hour at -78˚C, $BF_3{\cdot}OEt_2$ (2.5 mL, 19.5 mmol, 1.3 equiv) was added at this temperature. The solution stirred for 25 minutes, at which time (*S*)-**A** (2.2 g in 15 mL THF, 15 mmol, 1.0 equiv) was added dropwise at -78˚C. The reaction was stirred at -78˚C for 4 hours, quenched with saturated aqueous NH_4Cl, and extracted 3 times with Et_2O. The combined organic layer was washed with brine, dried with $MgSO_4$, filtered, concentrated, and purified by flash chromatography (6-30% EtOAc/Hexanes) to give the product **B** (2.98 g, 13.9 mmol, 93% yield) as a yellow oil. ^{1}H NMR (400 MHz, $CDCl_3$) δ 4.48 (d, *J* = 5.6, 1H), 3.80–3.75 (m, 2H), 3.72–3.65 (m, 1H), 3.66–3.58 (m, 2H), 2.56–2.48 (m, 1H), 2.46–2.38 (m, 1H), 2.32 (d, *J* = 4.1 Hz, 1H), 2.19–2.11 (m, 2H), 1.55–1.47 (m, 2H), 1.24 (t, *J* = 7.1 Hz, 3H), 1.23 (t, *J* = 7.1 H, 3H), 0.97 (t, *J* = 7.4 Hz, 3H); ^{13}C NMR (100 MHz, $CDCl_3$) δ 103.7, 82.2, 76.1, 70.6, 63.8, 63.5, 22.6, 22.5, 20.9, 15.4, 13.6, 13.5; $[\alpha]_D^{20}$ = -14 (*c* = 1.19, $CHCl_3$).

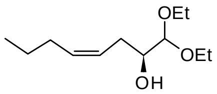

C

A stirred mixture of **B** (1.5 g, 7.0 mmol, 1.0 equiv), quinoline (42 µL, 0.35 mmol, 5 mol%), and Lindlar catalyst (0.149 g, 0.07 mmol, 1.0 mol %) in MeOH (25 mL) was sparged with N_2 for 15 minutes. H_2 gas was then bubbled through the stirred mixture for 1 hour. The reaction was filtered through a plug of Celite and concentrated to afford the product **C** (1.51 g, quantitative yield) as a yellow oil which was used without further purification. ^{1}H NMR (400 MHz, $CDCl_3$) δ 5.64–5.38 (m, 2H), 4.31 (d, *J* = 5.8 Hz, 1H), 3.87–3.68 (m, 2H), 3.68–3.49 (m, 3H), 2.46–2.32 (m, 1H), 2.32–2.19 (m, 1H), 2.15 (d, *J* = 3.5 Hz, 1H), 2.08–2.01 (m, 2H), 1.47–1.31 (m, 2H), 1.24 (q, *J* = 7.0 Hz, 6H), 0.91 (t, *J* = 7.3 Hz, 3H); ^{13}C NMR (100 MHz, $CDCl_3$) δ 132.2, 125.4, 104.6, 71.2, 63.5, 63.4, 29.9, 29.6, 22.9, 15.6, 15.5, 13.9; $[\alpha]_D^{20}$ = -6.4 (*c* = 1.06, $CHCl_3$).

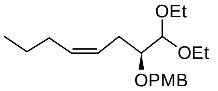

D

To a stirred suspension of NaH (0.21 g, 5.25 mmol, 1.5 equiv) and tetrabutylammonium iodide (0.132 g, 0.35 mmol, 0.1 equiv) in 25 mL DMF at 0 ºC was slowly added **C** (0.76 g in 5 mL DMF, 3.5 mmol, 1.0 equiv). After stirring for 1 hour at 0 ºC, PMBCl (0.97 mL, 7.0 mmol, 2.0 equiv) was added at this temperature and the reaction was allowed to warm to room temperature. The reaction was allowed to stir for 24 hours, after which it was quenched by slow addition of H_2O at 0 ºC. The reaction was extracted 3 times with Et_2O, and the combined organic layer was washed with brine, dried with $MgSO_4$, filtered, concentrated and purified by flash chromatography (2-10% EtOAc in hexanes) to afford **D** (1.11 g, 94% yield) as a light yellow oil. ^{1}H NMR (400 MHz, $CDCl_3$) δ 7.36–7.19 (m, 2H), 6.90–6.76 (m, 2H), 5.56–5.36 (m, 2H), 4.64 (d, *J* = 11.2 Hz, 1H), 4.54 (d, *J* = 11.2 Hz, 1H), 4.37 (d, *J* = 6.0 Hz, 1H), 3.76 (s, 3H), 3.76–3.66 (m, 2H), 3.66–3.57 (m, 1H), 3.57–3.48 (m, 1H), 3.46–3.37 (m, 1H), 2.46–2.33 (m, 1H), 2.33–2.22 (m, 1H), 2.10–1.91 (m, 2H), 1.44–1.29 (m, 2H), 1.29–1.14 (m, 6H), 0.89 (t, *J* = 7.4 Hz, 3H); ^{13}CNMR (100 MHz, $CDCl_3$) δ 159.3, 131.6, 131.2, 129.6, 126.0, 113.8, 104.8, 79.8, 72.9, 64.0, 63.4, 55.3, 29.7, 28.7, 23.0, 15.7, 15.5, 14.0; $[\alpha]_D^{20}$ = +13 (*c* = 1.0, $CHCl_3$).

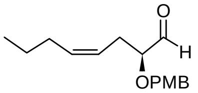

E

To a stirred solution of **D** (0.22 g, 0.654 mmol, 1.0 equiv) in 45 mL THF at 0 ºC was added a 15 mL freshly prepared solution of 10% HCl. Hydrolysis of the diethyl acetal was complete after 44 hours of stirring at 0 ºC, after which the solution was poured into a stirred solution of saturated aqueous $NaHCO_3$ at 0 ºC. The crude aldehyde **E** was extracted from the aqueous layer by washing once with EtOAc and twice with DCM. The organic layer was pooled, dried with $MgSO_4$, filtered and concentrated. The crude aldehyde was unstable so it was purified quickly by flash chromatography (2.5-10% EtOAc in hexanes with 0.1% Et_3N) afforded the aldehyde **E** (0.145 g, 85% yield) as a colorless oil. This α–hydroxy aldehyde **E** is very unstable and was used directly in the following reaction without being completely characterized.

Significant decomposition occurs when stored overnight in the freezer; however, a small aliquot was removed to obtain the ^{1}H NMR spectrum. ^{1}H NMR (400MHz, $CDCl_3$) δ 9.62 (d, *J* = 2.1 Hz, 1H), 7.28 (d, *J* = 8.5 Hz, 2H), 6.88 (d, *J* = 8.6 Hz, 2H), 5.57–5.47 (m, 1H), 5.46–5.35 (m, 1H), 4.59 (d, *J* = 11.5 Hz, 2H), 4.53 (d, *J* = 11.5 Hz, 2H), 3.81 (s, 3H), 3.77 (dt, *J* = 6.5, 2.2 Hz, 1H), 2.49–2.42 (m, 2H), 2.04–1.95 (m, 2H), 1.42–1.30 (m, 2H), 0.89 (t, *J* = 7.4 Hz, 3H).

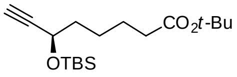

F

TBSCl (0.82 g, 5.28 mmol, 1.5 eqiv) was added to a solution of **k** (1.0 g, 3.52 mmol, 1.0 equiv), imidazole (0.72 g, 10.56 mmol, 3.0 equiv), and DMAP (43 mg, 0.352 mmol, 0.1 equiv) in 20 mL DCM at 0 ºC. The reaction was stirred for 30 minutes at 0 ºC and 2.5 hours at room temperature. MeOH (30 mL) and K_2CO_3 (1.7 g, 12.32 mmol, 3.5 equiv) were then added to the reaction sequentially at room temperature. After stirring overnight, the reaction was quenched with saturated aqueous $NaHCO_3$ and extracted 3 times with EtOAc. The organic layer was combined, washed with brine, dried with $MgSO_4$, filtered and concentrated. The crude mixture was filtered through a plug of SiO_2 with 10% EtOAc in hexanes to afford **F** (1.18 g, quantitative yield) which was used without further purification. ^{1}H NMR (400 MHz, $CDCl_3$) δ 4.34 (dt, *J* = 6.4, 2.1 Hz, 1H), 2.37 (d, *J* = 2.1 Hz, 1H), 2.22 (t, *J* = 7.5 Hz, 2H), 1.73–1.64 (m, 2H), 1.64–1.56 (m, 2H), 1.51–1.45 (m, 2H), 1.45 (s, 9H), 0.90 (s, 9H), 0.11 (d, *J* = 10.1 Hz, 6H); ^{13}C NMR (100 MHz, $CDCl_3$) δ 173.0, 85.5, 80.0, 72.3, 62.7, 38.3, 35.6, 28.2, 25.9, 24.9, 24.7, 18.3, 4.43, 4.96; $[\alpha]_D^{20}$ = +30.9 (*c* = 1.1, $CHCl_3$).

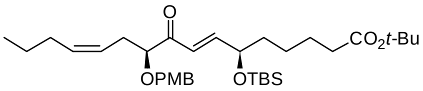

G

Solid $Cp_2Zr(H)Cl$ (0.614 g, 2.26 mmol, 1.7 equiv) was added to a stirred solution of **F** (0.652 g, 2.00 mmol, 1.5 equiv) in 7 mL DCM at 0 ºC. The hydrozirconation was complete after 2 hours, at which time a solution of $ZnBr_2$ (2.0 M in toluene, 0.133 mL, 0.133 mmol, 0.1 equiv) was added to the reaction mixture. After stirring for an addition 15 minutes at 0 ºC, a solution of the aldehyde (**E**, 0.35 g, 1.33 mmol, 1.0 equiv) in 2 mL DCM was added at the same temperature. The aldehyde was allowed to react overnight at 0 ºC and the reaction was quenched with a 10% aqueous solution of citric acid. The organic layer was separated, and the aqueous layer was washed 3 times with DCM. The organic layer was combined, washed with brine, dried with $MgSO_4$, filtered and concentrated to afford the desired alcohol as a mixture of diastereomers. The crude oil was taken directly into the Dess-Martin oxidation without further purification.

The above crude alcohol was dissolved in 15 mL DCM, treated with $NaHCO_3$ (0.328 g, 3.9 mmol, 3.0 equiv), and cooled to 0 ºC. Dess-Martin periodinane (1.14 g, 2.6 mmol, 2.0 equiv) was added to the stirred suspension in 3 portions. After 15 minutes, the reaction was removed from the ice bath and allowed to slowly warm to room temperature. The oxidation was complete after stirring for 1.5 hours at room temperature. The reaction was quenched with a solution of 20% $Na_2S_2O_3$/saturated aqueous $NaHCO_3$ (1:1), extracted 3 times with DCM. The combined layer was washed with brine, dried with $MgSO_4$, filtered, concentrated and purified by flash chromatography (5-8% EtOAc in hexanes) afforded of the ketone **G** (0.646 g, 84% yield over two steps) as a yellow oil. ^{1}H NMR (400 MHz, $CDCl_3$) δ 7.25–7.21 (m, 2H), 6.98 (dd, *J* = 15.6, 4.5 Hz, 1H), 6.88–6.84 (m, 2H), 6.66 (dd, *J* = 15.6, 1.6 Hz, 1H), 5.52–5.44 (m, 1H), 5.42–5.32 (m, 1H), 4.53 (d, *J* = 11.5 Hz, 1H), 4.34–4.29 (m, 2H), 3.92 (t, *J* = 6.6 Hz, 1H), 3.80 (s, 3H), 2.44 (t, *J* = 7.1 Hz, 2H), 2.19 (t, *J* = 7.5 Hz, 2H), 2.02–1.92 (m, 2H), 1.63–1.51 (m, 4H), 1.43 (s, 9H), 1.40–1.30 (m, 4H), 0.89 (s, 9H), 0.87 (t, *J* = 7.4 Hz, 3H), 0.07–-0.02 (m, 6H); ^{13}C NMR (100 MHz, $CDCl_3$) δ 173.4, 159.4, 136.28, 132.1, 130.8, 129.5, 127.9, 125.9, 114.0, 82.3, 80.2, 73.2, 72.9, 72.1, 55.5, 38.2, 35.8, 29.7, 28.4, 27.9, 26.1, 25.3, 24.9, 22.9, 18.5, 14.1, -4.03, -4.52; MS (ESI) calcd for $C_{34}H_{56}O_6SiNa$ $(M+Na)^+$ 611.9, found 611.4.

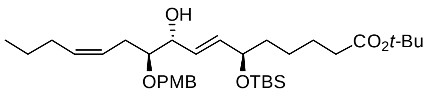

H

Ketone **G** (0.3 g, 0.5 mmol, 1.0 equiv) was dissolved in 10 mL DCM and cooled to -78 ºC. Super Hydride (1.0 M in THF, 0.6 mL, 1.2 equiv) was added slowly and the resulting solution was allowed to stir for 20 minutes at -78 ºC. Then the reaction was quenched with a 10% aqueous solution of citric acid, allowed to warm to room temperature, and stirred until two clear layers were present The aqueous layer was washed 3 times with DCM. The organic layer was combined, washed with brine, dried with $MgSO_4$, filtered, concentrated and purified by flash chromatography (8-18% EtOAc in hexanes) to afford the desired alcohol **H** (19:1 selectivity, 0.244 g, 83% yield) ^{1}H NMR (400 MHz, $CDCl_3$) δ 7.26 (d, *J* = 8.7 Hz, 2H), 6.87 (d, *J* = 8.7 Hz, 2H), 5.76–5.59 (m, 2H), 5.53–5.35 (m, 2H), 4.58 (d, *J* = 11.3 Hz, 1H), 4.52 (d, *J* = 11.3 Hz, 1H), 4.29–4.21 (m, 1H), 4.18–4.09 (m, 1H), 3.81 (s, 3H), 3.51–3.39 (m, 1H), 2.42–2.30 (m, 1H), 2.24–2.12 (m, 4H), 2.04–1.96 (m, 2H), 1.62–1.46 (m, 4H), 1.43 (s, 9H), 1.39–1.28 (m, 4H), 0.91–0.85 (m, 12H), 0.03 (d, *J* = 8.8 Hz, 6H); ^{13}C NMR (100 MHz, $CDCl_3$) δ 173.4, 159.4, 136.3, 132.1, 130.8, 129.5, 127.9, 125.9, 114.0, 82.3, 80.2, 73.2, 72.9, 72.1, 55.5, 38.2, 35.8, 29.7, 28.4, 27.9, 26.1, 25.3, 24.9, 23.0, 18.5, 14.1, -4.03, -4.52; MS (ESI) calcd $C_{34}H_{58}O_6SiNa$ $(M+Na)^+$ 613.9, found 613.4.

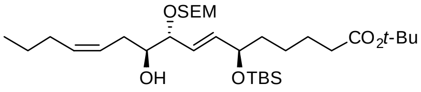

14

SEMCl (0.105 mL, 0.563 mmol, 1.3 equiv) was added dropwise to a stirred solution of **H** (0.256 g, 0.433 mmol, 1.0 equiv) and *i*-Pr_2NEt (0.13 mL, 0.736 mmol, 1.7 equiv) in 2.0 mL DCM at 0 ºC. The solution was allowed to slowly warm to room temperature and stir overnight. Then the reaction was quenched with saturated aqueous NH_4Cl, The organic layer was combined, washed with brine, dried with $MgSO_4$, filtered, and concentrated to afford the crude SEM-protected alcohol. The crude compound was taken directly into the following reaction without further purification.

Phosphate buffer (pH = 7, 0.31 mL) was added to the crude SEM-protected alcohol (0.433 mmol, 1.0 equiv) in 5.5 mL DCM and the heterogeneous mixture was cooled to 0 ºC. DDQ (0.151 g, 0.65 mmol, 1.5 equiv) was then added in two portions and the resulting mixture was allowed to stir overnight at 0 ºC, after which the reaction was filtered through a plug of Celite, washed with saturated aqueous $NaHCO_3$ and extracted 3 times with DCM. The organic layer was washed with brine, dried with $MgSO_4$, filtered, concentrated, and purified by flash chromatography (5-8% EtOAc in hexanes) to afford the product **14** (0.241 g, 95% yield) as a yellow oil. ^{1}H NMR (400 MHz, $CDCl_3$) δ 5.73 (dd, *J* = 15.6, 5.9 Hz, 1H), 5.62–5.49 (m, 2H), 5.49–5.39 (m, 1H), 4.70–4.62 (m, 2H), 4.19–4.11 (m, 1H), 4.04 (dd, *J* = 7.7, 3.6 Hz, 1H), 3.80–3.65 (m, 2H), 3.52 (dt, *J* = 9.8, 6.6 Hz, 1H), 2.25 (d, *J* = 4.5 Hz, 1H), 2.24–2.13 (m, 6H), 2.06–1.98 (m, 2H), 1.62–1.46 (m, 5H), 1.44 (s, 9H), 1.41–1.33 (m, 3H), 0.94–0.89 (m, 3H), 0.88 (s, 9H), 0.04 (s, 3H), 0.02 (s, 12H); ^{13}C NMR (100 MHz, $CDCl_3$) δ 173.3, 139.8, 132.7, 125.6, 124.8, 92.4, 80.2, 79.7, 73.9, 72.8, 65.7, 38.3, 35.8, 30.7, 29.9, 29.8, 28.3, 26.1, 25.3, 25.3, 25.0, 23.0, 18.4, 18.3, 14.0, -1.18, -4.10, -4.56; MS (ESI) calcd $C_{32}H_{64}O_6Si_2Na$ $(M+Na)^+$ 624.0, found 623.4.

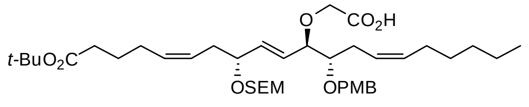

f

NaH (60 % dispersion in mineral oil, 454.4 mg, 11.4 mmol, 4.0 equiv) was weighed and added to a flask in a glove box, suspended in 4 mL DMF and cooled to 0 ºC. A solution of bromoacetic acid (789.2 g, 5.7 mmol, 2.0 equiv) in 4 mL THF was added dropwise to the stirred suspension of NaH at 0 ºC. The reaction was allowed to stir for 30 minutes at this temprature, and a solution of **13** (1.88 g, 2.84 mmol, 1.0 equiv) in 4 mL THF was added dropwise via cannula at 0 ºC. After 30 minutes, the solution was allowed to warm to room temperature and stir overnight. Then the reaction was quenched with saturated aqueous NaH_2PO_4 at 0 ºC, extracted with EtOAc, and dried with $MgSO_4$. The crude mixture was filtered through a plug of SiO_2 with 60% EtOAc in hexanes to afford the desired product **f** (1.86 g, 91% yield), which was used in the following reaction without further purification. ^{1}H NMR (400 MHz, $CDCl_3$) δ 7.32–7.17 (m, 2H), 6.90–6.82 (m, 2H), 5.61–5.53 (m, 2H), 5.52–5.32 (m, 4H), 4.63 (d, *J* = 6.8 Hz, 1H), 4.60 (d, *J* = 11.6 Hz, 1H), 4.59 (d, *J* = 6.8 Hz, 1H), 4.55 (d, *J* = 11.6 Hz, 1H),), 4.18

(d, J = 17.1 Hz, 1H), 4.14–4.07 (m, 1H), 3.90 (d, J = 17.2 Hz, 1H), 3.90–3.85 (m, 1H), 3.80 (s, 3H), 3.77–3.67 (m, 1H), 3.57–3.43 (m, 2H), 2.48–2.13 (m, 6H), 2.11–1.92 (m, 4H), 1.71–1.56 (m, 2H), 1.43 (s, 9H), 1.38–1.19 (m, 6H), 0.98–0.81 (m, 5H), 0.01 (s, 9H); ^{13}C NMR (100 MHz, $CDCl_3$) δ 173.11, 171.87, 159.42, 135.28, 132.48, 131.19, 129.87, 129.39, 127.89, 125.46, 125.06, 113.80, 91.98, 83.67, 80.20, 80.16, 75.38, 72.48, 66.51, 65.16, 55.21, 34.96, 33.25, 31.50, 29.19, 28.08, 27.43, 27.37, 26.77, 24.86, 22.55, 18.00, 14.04, -1.43; MS(ESI) calcd for $C_{40}H_{66}O_9SiNa$ $(M+Na)^+$ 741.4, found 741.4; HRMS(ESI) calcd for $C_{40}H_{66}O_9SiNa$ $(M+Na)^+$ 741.4374, found 741.4357.

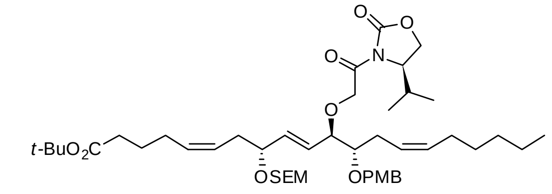

g

Carboxylic acid **f** (1.86 g, 2.6 mmol, 1.0 equiv) was added to a flask and dissolved in 30 mL Et_2O. Triethylamine (578 μL, 4.2 mmol, 1.6 equiv) was added and the stirred solution was cooled to -78 ºC. Pivaloyl chloride (479 μL, 3.9 mmol, 1.5 equiv) was then added dropwise to the reaction, which was stirred for additional 20 minutes at -78 ºC and allowed to slowly warm to 0 ºC. After stirring for 1 hour at this temperature, the solution was cooled once again to -78 ºC.

In a separate 100 mL flask was placed (*R*)-(+)-4-isopropyl-2-oxazolidinone (569.3 mg, 4.4 mmol, 1.7 equiv) in 30 mL THF. The solution was cooled to -78 ºC, and *n*-BuLi (2.5 M in hexanes, 1.66 mL, 4.2 mmol, 1.6 equiv) was added dropwise via syringe. The solution was stirred for 20 minutes, then the contents in the flask were subsequently transferred via cannula into the stirred suspension of mixed anhydride at -78 ºC. After stirring for 20 minutes at -78 °C, the solution was allowed to warm to 0 ºC and stir for 1 hour at this temperature. Then the reaction was quenched with saturated aqueous NH_4Cl, extracted 3 times with EtOAc. The combined organic layer was washed with brine, dried with $MgSO_4$, filtered, concentration and purified by flash chromatography (5-25% EtOAc in hexanes) to afford the desired oxazolidinone **g** (1.96 g, 91% yield over two steps) as a colorless oil. ^{1}H NMR (400 MHz, $CDCl_3$) δ 7.29–7.23 (m, 2H), 6.87–6.77 (m, 2H), 5.71 (dd, J = 15.7, 7.9 Hz, 1H), 5.57 (dd, J = 15.7, 7.4 Hz, 1H), 5.49–5.34 (m, 4H), 4.75–4.48 (m, 6H), 4.45–4.36 (m, 1H), 4.30 (t, J = 8.7 Hz, 1H), 4.23 (dd, J = 9.1, 3.1 Hz, 1H), 4.16–4.07 (m, 1H), 3.91 (dd, J = 7.9, 4.0 Hz, 1H), 3.78 (s, 3H), 3.77–3.67 (m, 1H), 3.66–3.57 (m, 1H), 3.53–3.42 (m, 1H), 2.49–2.14 (m, 6H), 2.52–2.11 (m, 4H), 1.68–1.55 (m, 2H), 1.43 (s, 9H), 1.37–1.18 (m, 7H), 0.98–0.77 (m, 11H), 0.01 (s, 9H); ^{13}C NMR (100 MHz, $CDCl_3$) δ 172.92, 170.06, 158.93, 153.81, 135.64, 131.96, 131.00, 130.96, 129.38, 129.34, 125.73, 125.31, 113.53, 91.83, 82.67, 80.70, 79.90, 75.48, 72.27, 67.87, 65.03, 64.15, 58.13, 55.18, 34.97, 33.39, 31.51, 29.26, 29.20, 28.07, 27.39, 26.77, 24.85, 22.55, 18.01, 17.87, 14.47, 14.04, -1.44; $[\alpha]_D^{20}$ = -10.6 (c = 1.0, $CHCl_3$); MS(ESI) calcd for $C_{46}H_{79}N_2O_{10}Si$ $(M+NH_4)^+$ 847.5, found 847.6; HRMS(ESI) calcd for $C_{46}H_{75}NO_{10}SiNa$ $(M+Na)^+$ 852.5058, found 852.5042.

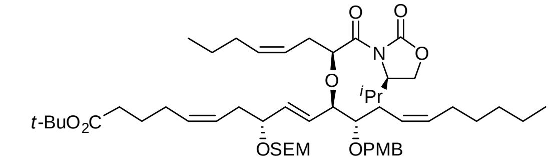

15

(*Z*)-1-Iodohex-2-ene was prepared using a modification of a known procedure.[8]

Alkylation of the oxazolidinone **g** with (*Z*)-1-iodohex-2-ene was performed using a modification of a known procedure.[9] A solution of sodium bistrimethylsilylamide (0.6 M in toluene, 1.09 mL, 0.65 mmol, 0.9 equiv) was added slowly to a stirred solution of **g** (601.0 g, 0.72 mmol, 1.0 equiv) in 12 mL THF at -78 ºC. The solution was stirred at -78 ºC for 40 minutes, after which (*Z*)-1-iodohex-2-ene (760.4 mg, 3.62 mmol, 5.0 equiv) was added in one portion (not dropwise). The solution was allowed to stir for an additional 2 hours at -78 °C, then the reaction was quenched with saturated aqueous NH_4Cl at this temperature. The suspension was allowed to warm to room temperature, and extracted 3 times with EtOAc. The combined layer was washed with brine, dried with $MgSO_4$, filtered, concentration and purified by flash chromatography (5-25% EtOAc in hexanes) to provide the desired product **15** (523.0 mg, 79%) as a colorless oil and recover the oxazolidinone **g** (55.0 mg, 9% yield). ^{1}H NMR (400 MHz, $CDCl_3$) δ 7.23 (d, J = 8.5 Hz, 2H), 6.82 (d, J

[8] a) Vankar, Y. D.; Rao, C. T. *Tetrahedron Lett.* **1985**, *26*, 2717. b) Kurashina, Yusuke. K; Shigefumi, *K., Biosci. Biotechnol. and Biochem.* **2012**, *76*, 605.
[9] Crimmins, M. T.; Emmitte, K. A.; Katz, J. D. *Org. Lett.* **2000**, *2*, 2165.

= 8.5 Hz, 2H), 5.65 (dd, J = 15.7, 7.4 Hz, 1H), 5.51 (dd, J = 15.6, 7.6 Hz, 1H), 5.49–5.33 (m, 6H), 5.18 (dd, J = 7.6, 4.1 Hz, 1H), 4.63 (d, J = 8.8 Hz, 1H), 4.62 (d, J = 9.2, Hz, 1H), 4.54 (d, J = 6.9 Hz, 1H), 4.46 (d, J = 11.1 Hz, 1H), 4.42–4.33 (m, 1H), 4.16–4.02 (m, 3H), 3.81 (dd, J = 7.4, 3.3 Hz, 1H), 3.77 (s, 3H), 3.76–3.67 (m, 1H), 3.57–3.51 (m, 1H), 3.49–3.39 (m, 1H), 2.58–2.39 (m, 2H), 2.38–2.13 (m, 6H), 2.10–1.91 (m, 6H), 1.68–1.54 (m, 2H), 1.41 (s, 9H), 1.38–1.13 (m, 9H), 0.95–0.74 (m, 14H), -0.01 (s, 9H); ^{13}C NMR (100 MHz, $CDCl_3$) δ 172.97, 172.56, 158.91, 153.39, 134.91, 132.52, 131.68, 131.14, 130.89, 129.91, 129.23, 125.94, 125.86, 124.12, 113.49, 91.73, 81.97, 81.78, 79.87, 75.53, 72.64, 64.99, 63.70, 58.08, 55.21, 35.02, 33.44, 31.65, 31.56, 29.39, 29.32, 28.25, 28.08, 27.40, 26.77, 24.90, 22.63, 22.57, 18.02, 17.84, 14.71, 14.05, 13.75, -1.44; $[\alpha]_D^{20}$ = -9.4 (c = 1.0, $CHCl_3$); MS(ESI) calcd for $C_{52}H_{85}NO_{10}SiNa$ $(M+Na)^+$ 934.6, found 934.5; HRMS(ESI) calcd for $C_{52}H_{85}NO_{10}SiNa$ $(M+Na)^+$ 934.5840, found 934.5834.

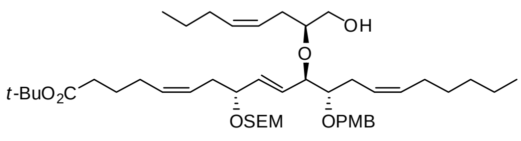

h

Compound **15** (432.0 mg, 0.47 mmol, 1.0 equiv) was dissolved in 8 mL THF, treated with anhydrous methanol (19 μL, 0.47 mL, 1.0 equiv), and cooled to 0 ºC. Lithium borohydride (2.0 M in THF, 284 μL, 0.57 mmol, 1.0 equiv) was added dropwise to the solution at 0 ºC and the reaction was allowed to stir overnight at this temperature. Then the reaction was quenched with a 10% aqueous solution of citric acid and the heterogeneous mixture was stirred for 1 hour at room temperature until two clear phases were present. The organic layer was separated and the aqueous layer was extracted 3 times with DCM. The combined layer was washed with brine, dried with $MgSO_4$, filtered, concentrated, and purified by flash chromatography (6-25% EtOAc in hexanes) to afford the desired alcohol **h** (349.9 g, 94 % yield) as a colorless oil. ^{1}H NMR (400 MHz, $CDCl_3$) δ 7.25 (d, J = 8.9 Hz, 2H), 6.85 (d, J = 8.6 Hz, 2H), 5.67–5.50 (m, 2H), 5.48–5.21 (m, 6H), 4.64 (d, J = 6.9 Hz, 1H), 4.57 (d, J = 6.9 Hz, 1H), 4.52 (s, 2H), 4.14–4.03 (m, 2H), 3.78 (s, 3H), 3.77–3.69 (m, 1H), 3.65–3.56 (m, 1H), 3.55–3.30 (m, 4H), 3.14–3.06 (m, 1H), 2.46–2.14 (m, 8H), 2.10–1.92 (m, 6H), 1.71–1.52 (m, 2H), 1.43 (s, 9H), 1.40–1.18 (m, 8H), 1.00–0.79 (m, 8H), 0.01 (s, 9H); ^{13}C NMR (100 MHz, $CDCl_3$) δ 172.88, 159.18, 132.59, 131.91, 131.89, 131.46, 130.90, 130.05, 129.52, 125.89, 125.77, 125.12, 113.67, 91.69, 81.66, 80.89, 79.96, 79.78, 75.47, 72.01, 65.01, 64.18, 55.17, 34.96, 33.39, 31.50, 30.33, 29.34, 29.21, 28.06, 27.42, 27.35, 26.76, 24.85, 22.63, 22.55, 18.02, 14.04, 13.76, -1.44; $[\alpha]_D^{20}$ = +41.6 (c = 1.0, $CHCl_3$); MS(ESI) calcd for $C_{46}H_{78}O_8SiNa$ $(M+Na)^+$ 809.5, found 809.5; HRMS(ESI) calcd for $C_{46}H_{78}O_8SiNa$ $(M+Na)^+$ 809.5364, found 809.5347.

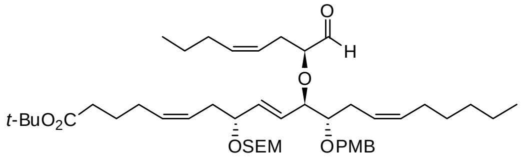

16

$NaHCO_3$ (220.3 mg, 2.62 mmol, 5.0 equiv) was added to the solution of the alcohol **h** (412.9 mg, 0.52 mmol, 1.0 equiv) in 5 mL DCM at 0 ºC. Dess-Martin periodinane (267.0 mg, 0.63 mmol, 1.2 equiv) was added to the stirred suspension in 2 portions. After stirring overnight at this temperature, the reaction was concentrated, then the reaction was quenched with a solution of 20% aqueous $Na_2S_2O_3$/saturated aqueous $NaHCO_3$ (1:1), and extracted 3 times with EtOAc. The combined organic layer was washed with brine, dried with $MgSO_4$, concentrated, filtered, and purified by flash chromatography (5-20% EtOAc in hexanes) to afford the product **16** (385.8 mg, 94% yield) as a colorless oil. ^{1}H NMR (400 MHz, $CDCl_3$) δ 9.62 (d, J = 2.2 Hz, 1H), 7.27–7.20 (m, 2H), 6.88–6.81 (m, 2H), 5.62 (dd, J = 15.7, 7.3 Hz, 1H), 5.52 (dd, J = 16.0, 7.2 Hz, 1H), 5.49–5.32 (m, 6H), 4.64 (d, J = 6.9 Hz, 1H), 4.58 (d, J = 11.2 Hz, 1H), 4.56 (d, J = 6.8 Hz, 1H), 4.51 (d, J = 11.3 Hz, 1H), 4.14–4.07 (m, 1H), 3.87 (dd, J = 7.3, 3.8 Hz, 1H), 3.79 (s, 3H), 3.78–3.69 (m, 2H), 3.56–3.51 (m, 1H), 3.52–3.43 (m, 1H), 2.43–2.14 (m, 8H), 2.12-1.90 (m, 6H), 1.70–1.51 (m, 2H), 1.43 (s, 9H), 1.38–1.22 (m, 8H), 1.00–0.80 (m, 8H), 0.01 (s, 9H)); ^{13}C NMR (100 MHz, $CDCl_3$) δ 203.90, 172.86, 159.04, 135.23, 132.94, 132.07, 131.02, 130.69, 129.37, 129.33, 125.73, 125.24, 123.19, 113.59, 91.79, 81.81, 81.27, 80.92, 79.95, 75.41, 72.27, 65.06, 55.19, 34.95, 33.39, 31.50, 29.38, 29.22, 28.83, 28.78, 28.07, 27.42, 26.78, 24.86, 22.56, 22.54, 18.03, 14.04, 13.75, -1.44; $[\alpha]_D^{20}$ = +12.6 (c = 1.0, $CHCl_3$); MS(ESI) calcd for $C_{46}H_{76}O_8SiNa$ $(M+Na)^+$ 807.5, found 807.4; HRMS(ESI) calcd for $C_{46}H_{76}O_8SiNa$ $(M+Na)^+$ 807.5207, found 807.5197.

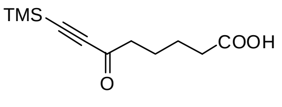

i

Following a similar procedure[10], freshly distilled adipic anhydride monomer[11] (12.8 g, 100 mmol, 2.0 equiv) and bis(trimethylsilyl)-acetylene (8.7 g, 50 mmol, 1.0 equiv) were dissolved in 600 mL DCM and brought to 0 ºC. Anhydrous $AlCl_3$ (20 g, 150 mmol, 3.0 equiv) was added in four portions to the stirred solution at 0 ºC. The dark mixture was stirred for additional 2.5 hours at this temperature and 19 hours at room temperature. The reaction was quenched by slowly adding 1N HCl at 0 ºC and the organic layer was separated, washed with 1N HCl, H_2O, and brine. The organic layer was dried with $MgSO_4$, filtered, concentrated, and purified by flash chromatography (5-50% EtOAc in hexanes) to afford the product **i** (6.91 g, 61% yield) as a viscous oil. ^{1}H NMR (400 MHz, $CDCl_3$) δ 2.59 (t, *J* = 7.0 Hz, 2H), 2.38 (t, *J* = 7.1 Hz, 2H), 1.84–1.54 (m, 4H), 0.23 (s, 9H); ^{13}C NMR (100 MHz, $CDCl_3$) δ 187.17, 179.42, 101.82, 98.09, 44.74, 33.63, 23.82, 23.11, -0.81; MS(ESI) calcd for $C_{11}H_{17}O_3Si$ $(M-H)^-$ 225.1, found 225.1; HRMS(ESI) calcd for $C_{11}H_{17}O_3Si$ $(M-H)^-$ 225.0947, found 225.0949.

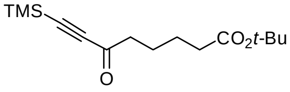

j

Trifluoroacetic anhydride (5.6 mL, 40.3 mmol, 2.2 equiv) was added slowly (over 15 minutes) to **i** (4.1 g, 18.3 mmol, 1.0 equiv) in 80 mL THF at 0 ºC and the reaction solution was allowed to stir for 2.5 hours at this temperature. *t*-BuOH (5 mL) was then added slowly to the stirred solution at 0 ºC and the reaction was allowed to slowly warm to room temperature. After stirring overnight at room temperature, the reaction was quenched with H_2O, diluted with DCM, and extracted 3 times with DCM. The combined organic layer was washed with brine, dried with $MgSO_4$, filtered, concentrated, and purified by flash chromatography (2-5% EtOAc in hexanes) to afford the product **j** (4.6 g, 89% yield) as a yellow oil. ^{1}H NMR (400 MHz, $CDCl_3$) δ 2.53 (t, *J* = 7.1 Hz, 2H), 2.18 (t, *J* = 7.0 Hz, 2H), 1.69–1.48 (m, 4H), 1.39 (s, 9H), 0.19 (s, 9H); ^{13}C NMR (100 MHz, $CDCl_3$) δ 187.19, 172.50, 101.83, 97.63, 80.05, 44.77, 35.07, 27.98, 24.22, 23.12, -0.88; MS(ESI) calcd for $C_{15}H_{26}O_3SiNa$ $(M+Na)^+$ 305.2, found 305.1; HRMS(ESI) calcd for $C_{15}H_{26}O_3SiNa$ $(M+Na)^+$ 305.1549, found 305.1538.

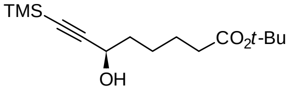

k

Noyori catalyst[12] (6.0 mg, 0.01 mmol, 1 mol %) was added to a stirred solution of **j** (282.4 mg, 1.0 mmol, 1.0 equiv) in 10 mL *i*-PrOH at room temperature and the reaction was allowed to stir overnight. The solution was concentrated and purified by flash chromatography (5-20% EtOAc) to afford the product **k** (271.0 mg, 95% yield, 99% ee) as a yellow oil. The ee of **k** (99%) was determined from the benzoylated analog **l** (BzCl, Et_3N, cat. DMAP, DCM) by HPLC (Chiralcel OD-H column, 1 mL/ min flow rate, 1% *i*PrOH/Hexanes; retention time of major: 7.95 min, retention time of minor: 9.18 min). ^{1}H NMR (400 MHz, $CDCl_3$) δ 4.35 (t, *J* = 6.6 Hz, 1H), 2.23 (t, *J* = 7.4 Hz, 2H), 1.87 (s, 1H), 1.74–1.67 (m, 2H), 1.67–1.57 (m, 2H), 1.52–1.45 (m, 2H), 1.44 (s, 9H), 0.16 (s, 9H); ^{13}C NMR (100 MHz, $CDCl_3$) δ 173.04, 106.61, 89.41, 80.09, 62.63, 37.30, 35.42, 28.10, 24.66, 24.59, -0.14; $[\alpha]_D^{20}$ = +0.6 (*c* = 1.04, $CHCl_3$); MS(ESI) calcd for $C_{15}H_{28}O_3SiNa$ $(M+Na)^+$ 307.2, found 307.1; HRMS(ESI) calcd for $C_{15}H_{28}O_3SiNa$ $(M+Na)^+$ 307.1705, found 307.1705; For the ent-**k**, $[\alpha]_D^{20}$ = -1.0 (*c* = 1.0, $CHCl_3$).

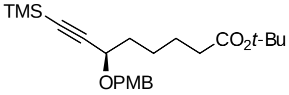

m

4-Methoxybenzyl-2,2,2-trichloroacetimidate[13] (1.3 g, 4.6 mmol, 1.5 equiv) was added slowly to **k** (881.5 mg, 3.1 mmol, 1.0 equiv) in 15 mL toluene at 0 ºC. $Cu(OTf)_2$ (112.1 mg, 0.31 mmol, 0.1 equiv) in 5 mL toluene was then added slowly to the stirred solution at 0 ºC. After stirring for 2 h at this temperature, the reaction was quenched with MeOH and sat. $NaHCO_3$, diluted with Et_2O, and extracted 3 times with Et_2O. The combined organic layer was washed with saturated aqueous NH_4Cl, 10% NH_4OH, H_2O and brine. The combined organic layer was dried with $MgSO_4$, filtered, concentrated, and purified by flash chromatography (5-20% EtOAc in hexanes) to afford the product **m** (1.08 g, 86% yield) as a colorless oil. ^{1}H NMR (500 MHz, $CDCl_3$) δ 7.28 (d, *J* =

[10] a) Jian, Y.-J.; Tang, C.-J.; Wu, Y. *J. Org. Chem.* **2007**, *72*, 4851. b) Nayyar, N. K.; Hutchison, D. R.; Martinelli, M. J. *J. Org. Chem.* **1997**, *62*, 982.

[11] a) Hill, J. W. *J. Am. Chem. Soc.* **1930**, *52*, 4110. b) Kazemi, F.; Kiasat, A. R.; Mombaini, B. *Synth. Commun.* **2007**, *37*, 3219.

[12] Haack, K. J.; Hashiguchi, S.; Fujii, A.; Ikariya, T.; Noyori, R. *Angew. Chem. Int. Ed.* **1997**, *36*, 285.

[13] Joly, G. D.; Jacobsen, E. N. *Org. Lett.* **2002**, *4*, 1795.

8.5 Hz, 2H), 6.87 (d, J = 8.6 Hz, 2H), 4.70 (d, J = 11.4 Hz, 1H), 4.43 (d, J = 11.4 Hz, 1H), 4.03 (t, J = 6.5 Hz, 1H), 3.80 (s, 3H), 2.20 (t, J = 7.5 Hz, 2H), 1.80–1.64 (m, 2H), 1.63–1.52 (m, 2H), 1.51–1.44 (m, 2H), 1.43 (s, 9H), 0.19 (s, 9H); ^{13}C NMR (100 MHz, $CDCl_3$) δ 172.80, 159.06, 129.90, 129.49, 113.56, 104.73, 90.37, 79.74, 69.88, 68.29, 55.02, 35.28, 35.15, 27.95, 24.63, -0.13; $[\alpha]_D^{20}$ = +83.8 (c = 1.0, $CHCl_3$); MS(ESI) calcd for $C_{23}H_{36}O_4SiNa$ $(M+Na)^+$ 427.2, found 427.2; HRMS(ESI) calcd for $C_{23}H_{36}O_4SiNa$ $(M+Na)^+$ 427.2281, found 427.2267; For the ent-**m**, $[\alpha]_D^{20}$ = -84.6 (c = 1.0, $CHCl_3$).

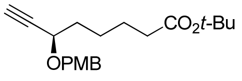

(R)-**17**

K_2CO_3 (739.6 mg, 5.4 mmol, 2.0 equiv) was added slowly to **m** (1.08 g, 2.7 mmol, 1.0 equiv) in 30 mL MeOH at room temperature. After stirring for 1 h at this temperature, the reaction was quenched with H_2O, diluted with Et_2O, and extracted 3 times with Et_2O. The combined organic layer was washed with saturated aqueous NH_4Cl, H_2O and brine. The organic layer was dried with $MgSO_4$, filtered, concentrated, and purified by flash chromatography (5-20% EtOAc in hexanes) to afford the product (R)-**17** (0.8 g, 90% yield) as a colorless oil. ^{1}H NMR (400 MHz, $CDCl_3$) δ 7.30–7.26 (m, 2H), 6.90–6.83 (m, 2H), 4.72 (d, J = 11.3 Hz, 1H), 4.43 (d, J = 11.3 Hz, 1H), 4.04 (td, J = 6.5, 2.0 Hz, 1H), 3.80 (s, 3H), 2.46 (d, J = 2.0 Hz, 1H), 2.20 (t, J = 7.4 Hz, 2H), 1.83–1.68 (m, 2H), 1.64–1.53 (m, 2H), 1.52–1.45 (m, 2H), 1.43 (s, 9H); ^{13}C NMR (100 MHz, $CDCl_3$) δ 173.00, 159.21, 129.82, 129.62, 113.73, 82.85, 80.00, 73.79, 70.11, 67.80, 55.23, 35.41, 35.26, 28.07, 24.73, 24.64; $[\alpha]_D^{20}$ = +80.8 (c = 1.0, $CHCl_3$); MS(ESI) calcd for $C_{20}H_{28}O_4Na$ $(M+Na)^+$ 355.2, found 355.1; HRMS(ESI) calcd for $C_{20}H_{28}O_4Na$ $(M+Na)^+$ 355.1885, found 355.1879.

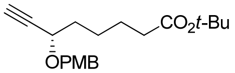

(S)-**17**

^{1}H NMR (400 MHz, $CDCl_3$) δ 7.29–7.22 (m, 2H), 6.88–6.82 (m, 2H), 4.70 (d, J = 11.3 Hz, 1H), 4.41 (d, J = 11.3 Hz, 1H), 4.02 (td, J = 6.5, 2.0 Hz, 1H), 3.78 (s, 3H), 2.44 (d, J = 2.0 Hz, 1H), 2.19 (t, J = 7.4 Hz, 2H), 1.81–1.64 (m, 2H), 1.61–1.51 (m, 2H), 1.51–1.43 (m, 2H), 1.41 (s, 9H); ^{13}C NMR (100 MHz, $CDCl_3$) δ 172.95, 159.19, 129.81, 129.59, 113.71, 82.84, 79.96, 73.77, 70.09, 67.78, 55.20, 35.38, 35.24, 28.05, 24.71, 24.62; $[\alpha]_D^{20}$ = -81.8 (c = 1.0, $CHCl_3$); MS(ESI) calcd for $C_{20}H_{28}O_4Na$ $(M+Na)^+$ 355.2, found 355.1; HRMS(ESI) calcd for $C_{20}H_{28}O_4Na$ $(M+Na)^+$ 355.1885, found 355.1881.

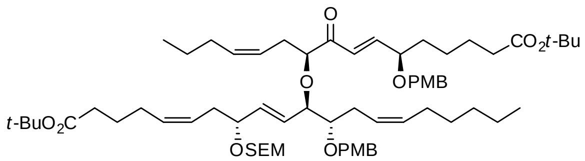

n

$Cp_2Zr(H)Cl$ (389.0 mg, 1.51 mmol, 2.0 equiv) was weighed and added to a flask in a glove box, suspended in 7 mL DCM and cooled to 0 ºC. A solution of alkyne (R)-**17** (501.5 mg, 1.51 mmol, 2.0 equiv) in 7 mL DCM was added to the stirred suspension of $Cp_2Zr(H)Cl$ at 0 ºC. Hydrozirconation was complete after 1.5 hours, after which the solution was cooled to -78 ºC. At this time, a solution of Me_2Zn (2.0 M in toluene, 754 µL, 1.51 mmol, 2.0 equiv) was added slowly to the solution of vinyl zirconium at -78 ºC. After stirring for 20 minutes at -78 ºC, the reaction was allowed to warm to 0 ºC. The solution was then allowed to stir for additional 20 minutes at 0 ºC, after which a solution of the aldehyde **16** (592.3 mg, 0.75 mmol, 1.0 equiv) in 3.5 mL toluene was added at the same temperature. The reaction was allowed to stir overnight at 0 ºC. Then the reaction was quenched with 10% aqueous solution of citric acid and the heterogeneous mixture was stirred for 1 h at room temperature until two clear phases were present. The organic layer was separated and the aqueous layer was extracted 3 times with DCM. The combined organic portion was washed with brine, dried with $MgSO_4$, filtered, concentrated, and purified by flash chromatography (3-25% EtOAc in hexanes) to afford the alcohol (855.3 mg) as a mixture of diastereomers.

$NaHCO_3$ (192.5 mg, 2.29 mmol, 3.0 equiv) was added to the solution of the above alcohol (855.3 mg, 0.76 mmol, 1.0 equiv) in 10 mL DCM at 0 ºC. Dess-Martin periodinane (356.4 mg, 0.84 mmol, 1.1 equiv) was added to the stirred suspension in 2 portions. After 0.5 hour, the reaction was allowed to slowly warm to room temperature. After 1.5 hours, the reaction was concentrated, then the reaction was quenched with a solution of 20% aqueous $Na_2S_2O_3$/saturated aqueous $NaHCO_3$ (1:1) and the aqueous layer was extracted 3 times with EtOAc, The combined organic layer was washed with brine, dried with $MgSO_4$, concentrated, filtered, and purified by flash chromatography (5-20% EtOAc in hexanes) to afford the product **n** (770.3 mg, 90% yield over two steps) as a colorless oil. ^{1}H NMR (400 MHz, $CDCl_3$) δ 7.27–7.16 (m, 4H), 6.89–6.78 (m, 5H), 6.66 (dd, J = 15.8, 0.9 Hz, 1H), 5.64 (dd, J = 15.7, 8.1 Hz, 1H), 5.53–5.28 (m, 7H), 4.67–4.39 (m, 5H), 4.21 (d, J = 11.3 Hz, 1H), 4.14–4.06 (m, 1H),

3.97–3.91 (m, 1H), 3.90–3.82 (m, 1H), 3.77 (s, 3H), 3.76 (s, 3H), 3.75–3.64 (m, 2H), 3.55–3.49 (m, 1H), 3.49–3.41 (m, 1H), 2.48–2.10 (m, 10H), 2.11–2.01 (m, 2H), 2.00–1.84 (m, 4H), 1.66–1.56 (m, 3H), 1.55–1.46 (m, 3H), 1.42 (s, 9H), 1.41 (s, 9H), 1.37–1.17 (m, 10H), 0.97–0.75 (m, 8H), -0.01 (s, 9H); ^{13}C NMR (100 MHz, $CDCl_3$) δ 200.65, 172.90, 172.86, 159.21, 158.97, 147.76, 135.81, 132.54, 131.97, 131.04, 130.97, 130.10, 129.27, 129.23, 128.94, 125.77, 125.36, 124.58, 123.81, 113.76, 113.55, 91.82, 82.03, 81.63, 81.07, 79.97, 79.93, 77.70, 75.48, 72.48, 70.77, 65.06, 55.20, 55.19, 35.35, 35.00, 34.69, 33.46, 31.50, 30.87, 29.49, 29.43, 29.22, 28.09, 28.07, 27.41, 26.81, 24.89, 24.88, 24.76, 22.62, 22.54, 18.03, 14.03, 13.77, -1.42; $[\alpha]_D^{20}$ = +14.4 (*c* = 0.25, $CHCl_3$); MS(ESI) calcd for $C_{66}H_{104}O_{12}SiNa$ $(M+Na)^+$ 1139.7, found 1139.7; HRMS(ESI) calcd for $C_{66}H_{104}O_{12}SiNa$ $(M+Na)^+$ 1139.7195, found 1139.7177.

Analytical sample of **23** (anti, anti) was prepared by deprotection of **19** with DDQ in phosphate buffer (pH = 7) and DCM. Selected chemical shifts and coupling constants for the syn, syn diastereomer were determined from the TOCSY spectrum of a mixture with **23** (anti, anti). (see Figure S2)

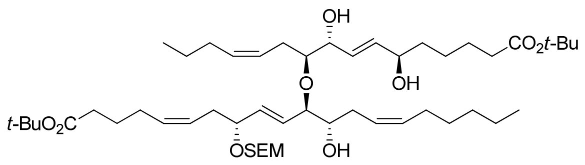

23

^{1}H NMR (600 MHz, C_6D_6:DMSO-d_6=25:2) δ 6.09–5.96 (m, 2H), 5.84 (dd, *J* = 14.8, 6.8 Hz, 1H), 5.88–5.79 (m, 1H), 5.69 (dd, *J* = 15.6, 7.3 Hz, 1H), 5.67–5.59 (m, 2H), 5.59–5.41 (m, 3H), 5.10 (d, *J* = 4.4 Hz, 1H), 4.88 (d, *J* = 6.2 Hz, 1H), 4.84 (d, *J* = 6.7 Hz, 1H), 4.65 (d, *J* = 6.7 Hz, 1H), 4.54–4.47 (m, 1H), 4.29–4.18 (m, 4H), 3.93–3.87 (m, 1H), 3.85 (dd, *J* = 17.0, 8.7 Hz, 1H), 3.69–3.63 (m, 1H), 3.53 (dd, *J* = 16.7, 8.8 Hz, 1H), 2.64–2.31 (m, 6H), 2.21–2.00 (m, 10H), 1.70–1.42 (m, 10H), 1.38 (s, 9H), 1.36 (s, 9H), 1.33–1.21 (m, 6H), 0.96 (t, *J* = 8.0 Hz, 2H), 0.90 (t, *J* = 7.7 Hz, 3H), 0.87 (t, *J* = 7.2 Hz, 3H), 0.00 (s, 9H).

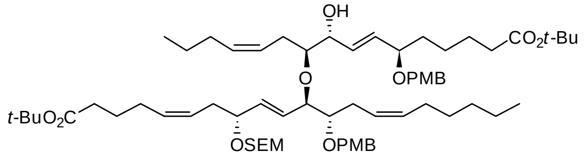

19

Ketone **n** (426.2 mg, 0.38 mmol, 1.0 equiv) was dissolved in 10 mL DCM and cooled to -78 ºC. Super-Hydride (1.0 M in THF, 496 µL, 0.5 mmol, 1.3 equiv) was added slowly and the resulting solution was allowed to stir for 0.5 h at -78 ºC. Then the reaction was quenched with a 10% aqueous solution of citric acid and the heterogeneous mixture was stirred for 1 h at room temperature until two clear phases were present. The organic layer was separated and the aqueous layer was extracted 3 times with DCM. The organic layer was washed with brine, dried with $MgSO_4$, filtered, concentrated, and purified by flash chromatography (3-25% EtOAc in hexanes) to afford the desired alcohol **19** (> 20:1 selectivity, 400.9 mg, 94 % yield) as a colorless oil. ^{1}H NMR (400 MHz, $CDCl_3$) δ 7.27–7.14 (m, 4H), 6.88–6.78 (m, 4H), 5.68 (dd, *J* = 15.6, 6.2 Hz, 1H), 5.63–5.49 (m, 3H), 5.46–5.28 (m, 6H), 4.62 (d, *J* = 6.9 Hz, 1H), 4.58–4.41 (m, 4H), 4.23 (d, *J* = 11.4 Hz, 1H), 4.18 (t, *J* = 4.8 Hz, 1H), 4.10 (dd, *J* = 5.1, 2.9 Hz, 1H), 4.09–4.02 (m, 1H), 3.77 (s, 3H), 3.76 (s, 3H), 3.75–3.67 (m, 2H), 3.58 (d, *J* = 6.3 Hz, 1H), 3.50–3.40 (m, 2H), 3.41–3.33 (m, 1H), 2.44–2.08 (m, 10H), 2.06–1.90 (m, 6H), 1.66–1.46 (m, 6H), 1.41 (s, 9H), 1.40 (s, 9H), 1.38–1.13 (m, 10H), 0.97–0.76 (m, 8H), -0.00 (s, 9H); ^{13}C NMR (100 MHz, $CDCl_3$) δ 173.07, 172.86, 159.22, 158.99, 132.77, 132.75, 131.89, 131.64, 131.35, 131.23, 130.93, 130.77, 130.01, 129.58, 129.24, 125.91, 125.79, 125.77, 113.70, 113.68, 91.70, 82.64, 81.86, 81.54, 79.96, 79.86, 78.93, 75.42, 72.83, 72.11, 69.62, 65.02, 55.19, 55.17, 35.47, 35.44, 34.97, 33.42, 31.52, 29.48, 29.23, 29.20, 28.08, 27.47, 27.36, 26.78, 24.98, 24.97, 24.87, 22.65, 22.57, 18.04, 14.05, 13.79, -1.43; $[\alpha]_D^{20}$ = +44.8 (*c* = 0.25, $CHCl_3$); MS(ESI) calcd for $C_{66}H_{106}O_{12}SiNa$ $(M+Na)^+$ 1141.7, found 1141.6.

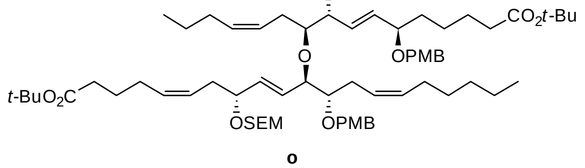

o

NaH (60% in mineral oil, 5.2 mg, 0.13 mmol, 1.5 equiv) was weighed and added to a vial in a glove box, suspended in 1.0 mL DMF and cooled to 0 ºC. A solution of the alcohol **19** (97.0 mg, 0.087 mmol, 1.0 equiv) in 1.0 mL DMF was dropwise added to the stirred suspension of NaH at 0 ºC. The reaction was stirred for 0.5 hour at this temperature. Then *p*-methoxybenzyl chloride (35 µL, 0.26

mmol, 3.0 equiv) and TBAI (tetrabutylammonium iodide, 6.4 mg, 0.017 mmol, 0.2 equiv) was added slowly to the stirred solution at 0 ºC. After 10 minutes, the reaction was allowed to slowly warm to room temperature. The reaction was allowed to stir overnight at room temperature. Then the reaction was quenched with saturated aqueous NaH_2PO_4 and the aqueous layer was extracted 3 times with EtOAc. The combined organic layer was washed with brine, dried with $MgSO_4$, concentrated, filtered, and purified by flash chromatography (5-15% EtOAc in hexanes) to afford the product **o** (107.9 mg, quantitative yield) as a colorless oil. ^{1}H NMR (400 MHz, $CDCl_3$) δ 7.25–7.17 (m, 6H), 6.88–6.76 (m, 6H), 5.75 (dd, *J* = 15.7, 7.9 Hz, 1H), 5.69 (dd, *J* = 15.9, 8.5 Hz, 1H), 5.54 (dd, *J* = 14.4, 7.5 Hz, 1H), 5.50 (dd, *J* = 15.4, 7.4 Hz, 1H), 5.46–5.33 (m, 6H), 4.70–4.62 (m, 2H), 4.58–4.44 (m, 4H), 4.28 (d, *J* = 6.6 Hz, 1H), 4.25 (d, *J* = 6.6 Hz, 1H), 4.20 (dd, *J* = 8.2, 3.5 Hz, 1H), 4.15–4.07 (m, 1H), 3.86–3.69 (m, 3H), 3.79 (s, 3H), 3.77 (s, 3H), 3.76 (s, 3H), 3.66–3.59 (m, 1H), 3.55–3.48 (m, 1H), 3.49–3.42 (m, 1H), 2.44–2.11 (m, 10H), 2.10–2.02 (m, 2H), 2.01–1.90 (m, 4H), 1.69–1.50 (m, 6H), 1.43 (s, 9H), 1.42 (s, 9H), 1.38–1.19 (m, 10H), 0.95–0.79 (m, 8H), 0.01 (s, 9H); ^{13}C NMR (100 MHz, $CDCl_3$) δ 173.03, 172.94, 159.02, 158.95, 158.86, 135.56, 134.55, 131.55, 131.30, 131.29, 131.16, 130.81, 130.71, 130.62, 130.36, 129.31, 129.25, 128.96, 126.06, 126.03, 125.93, 113.71, 113.64, 113.49, 91.70, 82.23, 82.17, 81.29, 79.96, 79.93, 78.90, 78.51, 75.62, 72.32, 70.06, 69.65, 65.01, 55.22, 55.21, 55.20, 35.61, 35.49, 35.02, 33.59, 31.54, 29.70, 29.65, 29.57, 29.30, 28.10, 27.45, 26.83, 25.13, 25.02, 24.91, 22.71, 22.59, 18.05, 14.08, 13.82, -1.40; $[\alpha]_D^{20}$ = +14.4 (*c* = 0.25, $CHCl_3$); MS(ESI) calcd for $C_{74}H_{114}O_{13}SiNa$ $(M+Na)^+$ 1261.8, found 1261.5; HRMS(ESI) calcd for $C_{74}H_{114}O_{13}SiNa$ $(M+Na)^+$ 1261.7926, found 1261.7887.

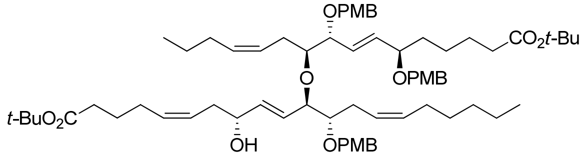

26

TASF (tris(dimethylamino)sulfonium difluorotrimethylsilicate, 420.8 mg, 1.53 mmol, 20.0 equiv) and 4Å MS (500 mg) were weighed, added to a flask in a glove box, and suspended in 5.0 mL HMPA (hexamethylphosphoramide) at room temperature. A solution of the SEM-ether **o** (94.7 mg, 0.076 mmol, 1.0 equiv) in 5.0 mL HMPA was added to the stirred suspension solution. The reaction was stirred for 48 hours at 80 ºC. Then the reaction was cooled to room temperature and filtered through a plug of Celite. Distilled water was added to the filtrate and the aqueous layer was extracted 3 times with EtOAc. The combined organic portion was washed with brine, dried with $MgSO_4$, concentrated, filtered, and purified by flash chromatography (5-25% EtOAc in hexanes) to afford the product **26** (75.1 mg, 89% yield) as a colorless oil and recover the starting material SEM-ether **o** (6.4 mg, 7% yield). ^{1}H NMR (400 MHz, $CDCl_3$) δ 7.25–7.18 (m, 6H), 6.87–6.77 (m, 6H), 5.75 (dd, *J* = 15.7, 7.9 Hz, 1H), 5.71–5.67 (m, 2H), 5.55 (dd, *J* = 15.6, 8.0 Hz, 1H), 5.51–5.32 (m, 6H), 4.67 (d, *J* = 11.2 Hz, 1H), 4.54–4.44 (m, 3H), 4.28 (d, *J* = 11.6 Hz, 1H), 4.26 (d, *J* = 11.6 Hz, 1H), 4.21–4.14 (m, 2H), 3.79 (s, 3H), 3.85–3.70 (m, 2H), 3.77 (s, 3H), 3.76 (s, 3H), 3.66–3.59 (m, 1H), 3.57–3.51 (m, 1H), 2.37–2.13 (m, 10H), 2.12–2.03 (m, 2H), 2.02–1.88 (m, 4H), 1.71–1.51 (m, 6H), 1.43 (s, 9H), 1.42 (s, 9H), 1.38–1.17 (m, 10H), 0.86 (t, *J* = 7.2 Hz, 6H); ^{13}C NMR (100 MHz, $CDCl_3$) δ 173.01, 172.98, 159.01, 158.94, 158.92, 137.25, 135.49, 132.06, 131.57, 131.32, 131.21, 130.69, 130.60, 130.34, 129.33, 129.28, 128.97, 128.56, 126.08, 125.88, 125.54, 113.70, 113.62, 113.52, 82.32, 82.03, 81.39, 80.05, 79.91, 78.87, 78.57, 72.51, 71.72, 70.06, 69.64, 55.20, 55.18, 35.57, 35.46, 35.15, 34.91, 31.51, 30.18, 29.79, 29.54, 29.26, 28.07, 27.46, 26.71, 25.09, 24.99, 24.90, 22.69, 22.56, 14.05, 13.80; $[\alpha]_D^{20}$ = -12.4 (*c* = 0.5, $CHCl_3$); MS(ESI) calcd for $C_{68}H_{100}O_{12}Na$ $(M+Na)^+$ 1131.6, found 1131.7.

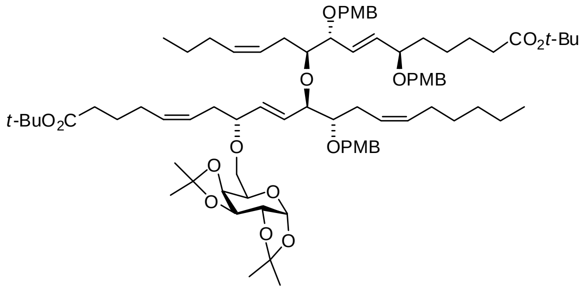

P

NaH (60 % dispersion in mineral oil, 10.0 mg, 0.25 mmol, 3.0 equiv) was weighed and added to a flask in a glove box, suspended in 0.5 mL THF/DMF (2/1) and cooled to 0 ºC. A solution of the alcohol **26** (92.2 g, 0.083 mmol, 1.0 equiv) in 0.8 mL THF/DMF (2/1) was added dropwise to the stirred suspension of NaH at 0 ºC. The reaction was allowed to stir for 30 minutes at this temperature, and a solution of sugar triflate **27**[14] (163.0 mg, 0.416 mmol, 5.0 equiv) in 0.2 mL THF/DMF (2/1) was added dropwise at 0 ºC. After 30 minutes, the solution was allowed to warm to room temperature and stir overnight. Then the reaction was quenched with saturated aqueous NaH_2PO_4 at 0 ºC, and extracted 3 times with EtOAc. The combined organic layer was washed with brine, dried with $MgSO_4$, concentrated, filtered, and purified by flash chromatography (5-25% EtOAc in hexanes) to afford the product galactose ether **p** (77.0 mg, 69% yield) as a colorless oil. ^{1}H NMR (400 MHz, $CDCl_3$) δ 7.24–7.17 (m, 6H), 6.87–6.76 (m, 6H), 5.75 (dd, *J* = 15.8, 7.9 Hz, 1H), 5.68 (dd, *J* = 15.2, 8.4 Hz, 1H), 5.59–5.48 (m, 3H), 5.47–5.30 (m, 6H), 4.69 (d, *J* = 11.2 Hz, 1H), 4.60–4.44 (m, 4H), 4.30–4.18 (m, 5H), 3.95–3.88 (m, 1H), 3.79 (s, 3H), 3.76 (s, 3H), 3.75 (s, 3H), 3.85–3.59 (m, 5H), 3.57–3.49 (m, 1H), 3.45 (dd, *J* = 10.0, 6.5 Hz, 1H), 2.44–2.11 (m, 10H), 2.09–1.90 (m, 6H), 1.65–1.47 (m, 6H), 1.51 (s, 3H), 1.43 (s, 9H), 1.42 (s, 9H), 1.40 (s, 3H), 1.31 (s, 3H), 1.30 (s, 3H), 1.37–1.17 (m, 10H), 0.85 (t, *J* = 7.2 Hz, 3H), 0.84 (t, *J* = 7.2 Hz, 3H); ^{13}C NMR (100 MHz, $CDCl_3$) δ 173.03, 172.96, 159.00, 158.93, 158.85, 135.47, 135.27, 131.53, 131.35, 131.22, 130.71, 130.63, 130.42, 129.30, 128.94, 126.25, 126.11, 125.98, 113.70, 113.64, 113.50, 108.99, 108.33, 96.27, 82.31, 82.11, 81.47, 80.60, 79.94, 79.91, 78.87, 78.43, 72.39, 70.99, 70.66, 70.59, 70.05, 69.61, 67.11, 66.38, 55.21, 55.19, 55.17, 35.59, 35.48, 35.00, 33.57, 31.48, 29.67, 29.56, 29.28, 28.09, 28.08, 27.39, 26.82, 26.09, 25.95, 25.11, 25.01, 24.92, 24.38, 22.70, 22.59, 14.06, 13.81; $[\alpha]_D^{20}$ = -28.4 (*c* = 1.0, $CHCl_3$); MS(ESI) calcd for $C_{80}H_{118}O_{17}Na$ $(M+Na)^+$ 1173.8, found 1173.7.

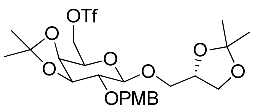

28

^{1}H NMR (400 MHz, $CDCl_3$) δ 7.32 (d, *J* = 8.2 Hz, 2H), 6.86 (d, *J* = 8.1 Hz, 2H), 4.73 (dd, *J* = 10.4, 10.4 Hz, 2H), 4.41–4.26 (m, 2H), 4.19–4.01 (m, 3H), 3.96 (dd, *J* = 12.7, 8.4 Hz, 1H), 3.90–3.74 (m, 4H), 3.80 (s, 3H), 3.67 (dd, *J* = 10.2, 5.8 Hz, 1H), 3.38 (dd, *J* = 6.8, 6.8 Hz, 1H), 1.57 (s, 3H), 1.43 (s, 3H), 1.37 (s, 3H), 1.33 (s, 3H).

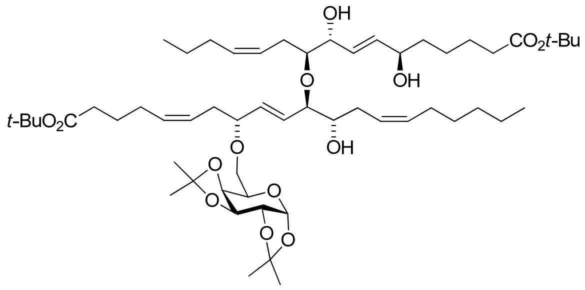

29

DDQ (29.2 mg, 0.13 mmol, 3.5 equiv) was added to a biphasic solution of PMB ether **p** (49.7 mg, 0.037 mmol) in 2 mL DCM and 200 μL phosphate buffer (pH = 7) at 0 ºC. The resulting solution was allowed to stir for 1 hour at 0 ºC, then warmed to room temperature for additional 1.0 hour. Then the reaction was quenched with saturated aqueous $NaHCO_3$ solution and extracted 3 times with DCM. The combined organic layer was washed with brine, dried with $MgSO_4$, concentrated, filtered, and purified by flash chromatography (5-50% EtOAc in hexanes) to afford the desired product triol **29** (29.5 mg, 81% yield) as a colorless oil. ^{1}H NMR (400 MHz, $CDCl_3$) δ 5.77–5.69 (m, 2H), 5.64–5.57 (m, 2H), 5.56–5.46 (m, 2H), 5.46–5.29 (m, 5H), 4.56 (dd, *J* = 8.0, 2.3 Hz, 1H), 4.27 (dd, *J* = 5.0, 2.3 Hz, 1H), 4.22 (dd, *J* = 7.9, 1.8 Hz, 1H), 4.20–4.16 (m, 1H), 4.15–4.09 (m, 1H), 4.05–4.00 (m, 1H), 3.93–3.87 (m, 1H), 3.80–3.73 (m, 1H), 3.70–3.54 (m, 2H), 3.51–3.42 (m, 2H), 2.41–2.08 (m, 10H), 2.08–1.90 (m, 6H), 1.67–1.53 (m, 6H), 1.51 (s, 3H), 1.42 (s, 9H), 1.41 (s, 9H), 1.40 (s, 3H), 1.30 (s, 3H), 1.30 (s, 3H), 1.38–1.20 (m, 10H), 0.87 (t, *J* = 7.4 Hz, 3H); 0.86 (t, *J* = 6.8 Hz, 3H); ^{13}C NMR (100 MHz, $CDCl_3$) δ 173.12, 173.06, 135.84, 134.64, 133.20, 131.72, 130.65, 129.83, 128.88, 125.89, 125.61, 125.18, 109.05, 108.41, 96.24, 82.14, 80.84, 80.48, 80.09, 80.05, 73.50, 73.00, 72.02, 71.02, 70.57, 67.32, 66.64, 36.71, 35.38, 34.98, 33.34, 31.45, 29.48, 29.25, 28.90, 28.08, 27.35,

[14] Xie, W.; Tanabe, G.; Akaki, J.; Morikawa, T.; Ninomiya, K.; Minematsu, T.; Yoshikawa, M.; Wu, X.; Muraoka, O. *Bioorg. Med. Chem.* **2011**, *19*, 2015.

26.79, 26.07, 25.93, 24.91, 24.89, 24.80, 24.36, 22.67, 22.55, 14.04, 13.81; $[\alpha]_D^{20}$ = -21.0 (*c* = 1.0, $CHCl_3$); MS(ESI) calcd for $C_{56}H_{94}O_{14}Na$ $(M+Na)^+$ 1013.7, found 1013.5; HRMS(ESI) calcd for $C_{56}H_{94}O_{14}Na$ $(M+Na)^+$ 1013.6541, found 1013.6521.

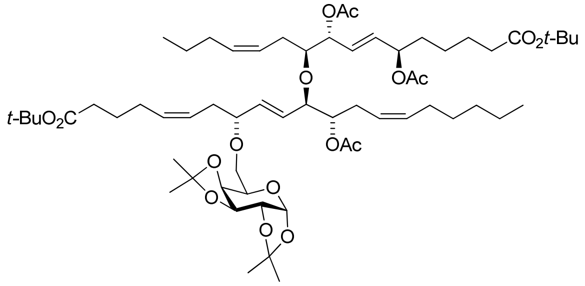

30

0.3 mL Pyridine and catalytic DMAP was added to a stirred solution of triol **29** (30.4 mg, 30.7 μmol) in 0.4 mL DCM at 0 ºC. Then 0.3 mL acetic anhydride was added dropwise to the stirred solution at 0 ºC. The mixture was allowed to warm to room temperature, stirred overnight and the solvent was removed azeotropically with toluene. The crude product was quenched with saturated aqueous NaH_2PO_4, and extracted 3 times with EtOAc. The combined organic layer was washed with brine, dried with $MgSO_4$, concentrated, filtered, and purified by flash chromatography (5-30% EtOAc in hexanes) to afford the product tri-acetate **30** (30.2 mg, 88% yield) as a colorless oil. ^1H NMR (400 MHz, $CDCl_3$) δ 5.74 (dd, *J* = 15.7, 6.9 Hz, 1H), 5.65 (dd, *J* = 15.7, 6.1 Hz, 1H), 5.56 (dd, *J* = 15.6, 6.8 Hz, 1H), 5.52–5.37 (m, 6H), 5.36–5.21 (m, 4H), 4.94–4.84 (m, 1H), 4.57 (dd, *J* = 7.9, 2.1 Hz, 1H), 4.27 (dd, *J* = 5.0, 2.2 Hz, 1H), 4.23 (dd, *J* = 8.0, 1.4 Hz, 1H), 4.01 (dd, *J* = 7.3, 5.4 Hz, 1H), 3.94–3.87 (m, 1H), 3.79–3.70 (m, 1H), 3.60 (dd, *J* = 9.8, 6.4 Hz, 1H), 3.54–3.47 (m, 1H), 3.40 (dd, *J* = 9.8, 6.5 Hz, 1H), 2.41–2.29 (m, 3H), 2.26–2.15 (m, 5H), 2.06 (s, 3H), 2.03 (s, 3H), 2.15–1.87 (m, 8H), 2.00 (s, 3H), 1.67–1.54 (m, 6H), 1.51 (s, 3H), 1.43 (s, 9H), 1.42 (s, 9H), 1.41 (s, 3H), 1.31 (s, 3H), 1.30 (s, 3H), 1.38–1.22 (m, 10H), 0.88 (t, *J* = 7.2 Hz, 3H), 0.87 (t, *J* = 7.2 Hz, 3H); ^{13}C NMR (100 MHz, $CDCl_3$) δ 172.90, 172.79, 170.40, 170.08, 169.78, 136.20, 133.49, 132.81, 131.83, 130.69, 129.94, 126.66, 125.85, 124.97, 124.28, 109.00, 108.35, 96.25, 80.53, 80.42, 80.01, 79.97, 77.39, 75.30, 74.82, 73.41, 70.96, 70.64, 70.57, 67.24, 66.49, 35.32, 34.96, 33.98, 33.41, 31.45, 29.51, 29.43, 29.25, 28.09, 28.07, 27.28, 26.79, 26.09, 25.95, 24.91, 24.88, 24.77, 24.61, 24.35, 22.62, 22.58, 21.22, 21.14, 14.05, 13.79; $[\alpha]_D^{20}$ = -26.8 (*c* = 1.0, $CHCl_3$); MS(ESI) calcd for $C_{62}H_{100}O_{17}Na$ $(M+Na)^+$ 1139.7, found 1139.4; HRMS(ESI) calcd for $C_{62}H_{100}O_{17}Na$ $(M+Na)^+$ 1139.7195, found 1139.7209.

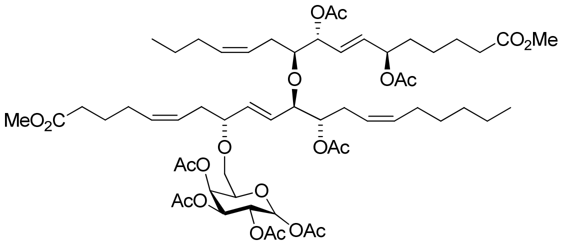

q

Trifluoroacetic acid (0.4 ml) was added to a stirred solution of tri-acetate **30** (9.0 mg, 8.05 μmol) in 1.0 mL DCM and 0.6 mL water at room temperature. Then the resulting solution was stirred overnight and monitored by ^1H NMR. When the reaction was completed, the solvent was removed azeotropically with toluene. After drying with high vacuum, the crude product was dissolved in 1.2 mL dry toluene and 0.8 mL dry MeOH, and $TMSCHN_2$ (2.0 M in hexanes, 300 μl) was added dropwise at 0 ºC until the gas evolution ceased. Then, the reaction was stirred for 30 minutes at 0 ºC and quenched with acetic acid. The solvent was removed azeotropically with toluene. The residual solvent was further removed by high vacuum and the crude product was dissolved in dry 0.8 mL DCM and 0.6 mL pyridine. After cooling to 0 ºC, catalytic DMAP was added to the stirred solution. Then 0.6 mL acetic anhydride was added dropwise to the solution at 0 ºC. The reaction was allowed to slowly warm to room temperature. After stirring overnight at room temperature, the solvent was removed azeotropically with toluene. The crude product was quenched with saturated aqueous NaH_2PO_4, and extracted 3 times with EtOAc. The combined organic layer was washed with brine, dried with $MgSO_4$, concentrated, filtered, and purified by a pipette column (5-50% EtOAc in hexanes) to afford the product **q** as a mixture of anomers (7.6 mg, 84% yield over three steps) as a colorless oil.

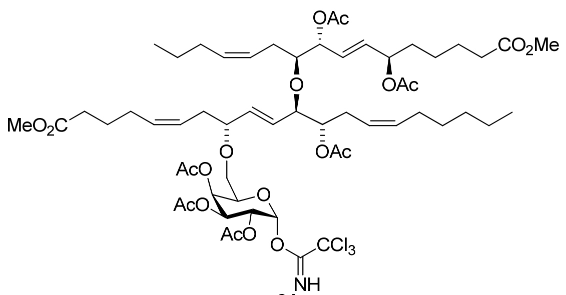

31

Hydrazine acetate (1.9 mg, 20.6 μmol, 3.0 equiv) was added to a stirred solution of the above product (7.6 mg, 6.8 μmol, 1.0 equiv) in 2.0 mL DMF at room temperature. After stirring for 3 h at room temperature, the reaction was quenched with saturated aqueous NaH_2PO_4, and extracted 3 times with EtOAc. The combined organic layer was washed with brine, dried with $MgSO_4$, concentrated, filtered, and purified by a pipette column (5-60% EtOAc in hexanes) to afford the product (5.5 mg, 75% yield) as a colorless oil.

Trichloroacetonitrile (8 μl, 80 μmol, 16.0 equiv) and catalytic DBU (1,8-diazabicyclo[5.4.0]undec-7-ene) were added to a stirred solution of the above product (5.5 mg, 5.1 μmol, 1.0 equiv) in 1.0 mL DCM at room temperature. After stirring for 3 hours at room temperature, the reaction was concentrated and purified quickly by a pipette column (5-60% EtOAc in hexanes) to afford the acetamidate **31** (3.8 mg) as a colorless oil. The sample was very unstable and so the acetamidate **31** was brought into the following reaction immediately. ^{1}H NMR (500 MHz, C_6D_6) δ 8.50 (s, 1H), 6.05–5.97 (m, 2H), 5.88 (dd, *J* = 10.9, 3.1 Hz, 1H), 5.79 (dd, *J* = 11.0, 3.5 Hz, 1H), 5.78–5.73 (m, 1H), 5.71–5.63 (m, 2H), 5.63–5.52 (m, 5H), 5.51–5.43 (m, 2H), 5.41–5.30 (m, 2H), 4.61 (t, *J* = 6.4 Hz, 1H), 4.23 (dd, *J* = 8.0, 5.6 Hz, 1H), 3.79–3.71 (m, 2H), 3.60–3.52 (m, 1H), 3.44–3.37 (m, 1H), 3.41 (s, 3H), 3.35 (s, 3H), 3.37–3.31 (m, 1H), 2.71–2.61 (m, 2H), 2.47–2.13 (m, 8H), 2.10–1.99 (m, 6H), 1.98 (s, 3H), 1.82 (s, 3H), 1.83 (s, 3H), 1.77 (s, 3H), 1.76 (s, 3H), 1.70–1.62 (m, 2H), 1.61 (s, 3H), 1.57–1.44 (m, 2H), 1.43–1.35 (m, 5H), 1.35–1.28 (m, 5H), 1.38–1.16 (m, 2H), 0.94 (t, *J* = 7.0 Hz, 3H), 0.93 (t, *J* = 7.0 Hz, 3H).

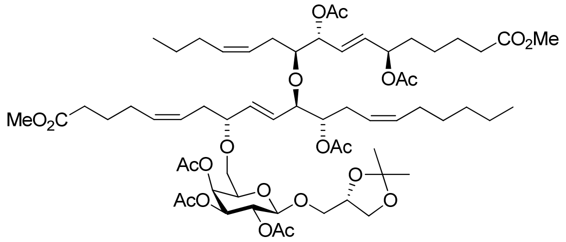

r

The acetamidate **31** (16.9 mg, 0.014 mmol, 1.0 equiv) and (*S*)-solketal **33** (17 μl, 0.14 mmol, 10.0 equiv) were dissolved in 2.0 mL DCM, and 200 mg freshly activated 5Å MS was added to the reaction. The resulting solution was stirred for 0.5 hour at room temperature and then cooled to -78 ºC. TMSOTf (1 μl, 0.006 mmol, 0.4 equiv) was added to the reaction at -78 ºC. After stirring for 2 hours at the same temperature, triethylamine (2 drops) was added to quench the reaction. Then the solvent was removed and the crude product was purified by a pipette column (5-60% EtOAc in hexanes) to afford the product **r** as a colorless oil (11.0 mg, 72 % yield). ^{1}H NMR (400 MHz, C_6D_6) δ 6.03 (dd, *J* = 15.6, 7.1 Hz, 1H), 5.77 (dd, *J* = 15.8, 6.8 Hz, 1H), 5.73–5.46 (m, 11H), 5.43–5.34 (m, 1H), 5.34–5.30 (m, 1H), 5.25 (dd, *J* = 10.5, 3.2 Hz, 1H), 4.40 (d, *J* = 7.9 Hz, 1H), 4.33–4.25 (m, 1H), 4.13–4.04 (m, 1H), 3.88–3.75 (m, 4H), 3.70 (dd, *J* = 9.1, 6.0 Hz, 1H), 3.63–3.47 (m, 3H), 3.41 (s, 3H), 3.36 (s, 3H), 3.42–3.26 (m, 1H), 2.77–2.60 (m, 2H), 2.49–2.13 (m, 8H), 2.13–1.92 (m, 6H), 2.00 (s, 3H), 1.84 (s, 3H), 1.79 (s, 3H), 1.78 (s, 3H), 1.77 (s, 3H), 1.76 (s, 3H), 1.72–1.61 (m, 2H), 1.43 (s, 3H), 1.58–1.08 (m, 14H), 1.26 (s, 3H), 0.93 (t, *J* = 7.2 Hz, 3H), 0.92 (t, *J* = 6.8 Hz, 3H); ^{13}C NMR (100 MHz, C_6D_6) δ 173.32, 173.03, 170.06, 169.97, 169.78, 169.44, 169.41, 168.98, 136.04, 133.95, 133.18, 132.21, 131.38, 131.06, 126.00, 125.79, 125.04, 109.24, 101.83, 81.07, 80.87, 78.13, 75.23, 75.14, 74.58, 73.56, 72.39, 71.66, 69.42, 68.11, 66.95, 66.59, 51.06, 50.96, 34.22, 33.85, 33.78, 33.38, 31.86, 29.88, 29.84, 29.71, 28.88, 27.80, 27.01, 26.90, 25.39, 25.07, 24.93, 24.83, 23.07, 22.99, 21.07, 20.82, 20.78, 20.45, 20.30, 20.19, 14.30, 14.02; $[\alpha]_D^{20}$ = -9.0 (*c* = 0.8, $CHCl_3$); MS (ESI) calculated for $C_{62}H_{96}O_{22}Na^+$ $(M+Na)^+$ 1215.6, found 1215.4.

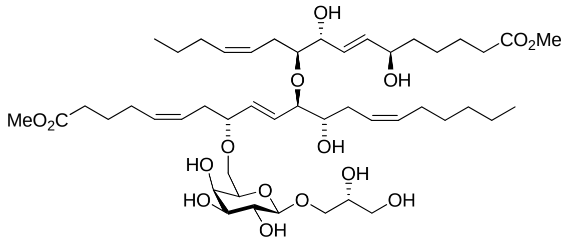

32

The per-acetate **r** (11.0 mg, 0.091 mmol, 1.0 equiv) was dissolved in 2.0 mL MeOH, treated with catalytic TsOH·H_2O (*p*-toluenesulfonic acid monohydrate) (0.35 mg, 1.8 μmol, 0.2 equiv), and the resulting solution was stirred overnight at room temperature. Then the reaction was concentrated and purified by a pipette column (5-60% EtOAc in hexanes) to afford the desired diol (6.5 mg). ^{1}H NMR (500 MHz, C_6D_6) δ 6.03 (dd, *J* = 15.6, 7.2 Hz, 1H), 5.77 (dd, *J* = 15.6, 6.7 Hz, 1H), 5.72–5.69 (m, 2H), 5.69–5.54 (m, 7H), 5.53–5.46 (m, 2H), 5.42–5.30 (m, 2H), 5.20 (dd, *J* = 10.5, 3.4 Hz, 1H), 4.31 (d, *J* = 7.9 Hz, 1H), 4.34–4.27 (m, 1H), 3.85–3.77 (m,

2H), 3.77–3.69 (m, 2H), 3.66–3.61 (m, 1H), 3.60–3.47 (m, 4H), 3.42 (s, 3H), 3.36 (s, 3H), 3.17 (dd, *J* = 9.4, 4.6 Hz, 1H), 2.78–2.61 (m, 2H), 2.52–2.26 (m, 4H), 2.24-2.13 (m, 4H), 2.10–2.03 (m, 6H), 2.01 (s, 3H), 1.85 (s, 3H), 1.83 (s, 3H), 1.78 (s, 3H), 1.75 (s, 3H), 1.74 (s, 3H), 1.71–1.60 (m, 2H), 1.57–1.34 (m, 8H), 1.34–1.27 (m, 4H), 1.27–1.17 (m, 2H), 0.93 (t, *J* = 7.5 Hz, 3H), 0.93 (t, *J* = 6.8 Hz, 3H); MS (ESI) calcd for $C_{59}H_{92}O_{22}Na^+$ $(M+Na)^+$ 1175.6, found 1175.4.

The diol (6.5 mg, 5.6 μmol, 1.0 equiv) was dissolved in 1.0 mL MeOH, treated with NaOMe (0.5 M in MeOH, 50 μL), and the resulting solution was stirred overnight at room temperature. Pure nigricanoside A dimethyl ester analog **32** (4.0 mg, 48% yield over two steps) was obtained via C18 reversed-phase HPLC using a phenomenex, luna 5 μ 250 × 10.00 mm 5 micro column, with 9:11 MeCN/H_2O as the eluent (no acid, flow rate = 2.5 mL/min, retention time: 68.0 min). The product was monitored at 194 nm wavelength. ^1H NMR (600 MHz, CD_3OD) δ 5.76 (dd, *J* = 15.7, 7.9 Hz, 1H), 5.71 (dd, *J* = 15.7, 7.8 Hz, 1H), 5.66 (dd, *J* = 15.7, 6.0 Hz, 1H), 5.61 (dd, *J* = 15.7, 6.0 Hz, 1H), 5.51–5.34 (m, 6H), 4.22 (d, *J* = 7.6 Hz, 1H), 4.20 (dd, *J* = 5.6, 3.0 Hz, 1H), 4.08 (ddd, *J* = 6.3, 6.3, 6.3 Hz, 1H), 4.02 (dd, *J* = 6.0, 3.5 Hz, 1H), 3.87 (dd, *J* = 10.4, 5.2 Hz, 1H), 3.85–3.80 (m, 2H), 3.80–3.75 (m, 1H), 3.70 (dd, *J* = 9.6, 6.1 Hz, 1H), 3.66 (dd, *J* = 10.4, 4.1 Hz, 1H), 3.66 (s, 3H), 3.65 (s, 3H), 3.56 (dd, *J* = 9.6, 6.1 Hz, 1H), 3.54 (dd, *J* = 9.7, 7.6 Hz, 1H), 3.62–3.52 (m, 4H), 3.47 (dd, *J* = 9.7, 3.4 Hz, 1H), 3.48–3.43 (m, 1H), 2.41–2.15 (m, 10H), 2.13–1.99 (m, 6H), 1.70–1.60 (m, 4H), 1.57–1.47 (m, 2H), 1.46–1.27 (m, 10H), 0.92 (t, *J* = 7.3 Hz, 3H); 0.91 (t, *J* = 7.1 Hz, 3H); ^1H NMR (600 MHz, C_6D_6:DMSO-d_6=25:2) δ 6.05 (dd, *J* = 15.5, 5.6 Hz, 1H), 6.00 (dd, *J* = 15.5, 5.6 Hz, 1H), 5.86 (dd, *J* = 15.6, 6.8 Hz, 1H), 5.84–5.80 (m, 1H), 5.79 (dd, *J* = 15.7, 6.9 Hz, 1H), 5.74–5.67 (m, 1H), 5.67–5.61 (m, 1H), 5.60–5.53 (m, 2H), 5.47–5.40 (m, 1H), 4.53–4.49 (m, 1H), 4.42 (d, *J* = 7.6 Hz, 1H), 4.28 (dd, *J* = 6.5, 3.0 Hz, 1H), 4.25–4.21 (m, 1H), 4.14 (dd, *J* = 10.4, 5.6 Hz, 1H), 4.11–4.07 (m, 1H), 4.06–4.03 (m, 1H), 3.93 (dd, *J* = 9.7, 5.9 Hz, 1H), 3.92 (dd, *J* = 10.4, 5.2 Hz, 1H), 3.99–3.90 (m, 2H), 3.90–3.84 (m, 3H), 3.81 (dd, *J* = 9.7, 5.9 Hz, 1H), 3.74–3.70 (m, 2H), 3.67 (bt, *J* = 6.0 Hz, 1H), 3.44 (s, 3H), 3.38 (s, 3H), 2.65–2.52 (m, 3H), 2.52–2.43 (m, 2H), 2.42–2.31 (m, 1H), 2.21–2.15 (m, 6H), 2.13 (ddd, *J* = 7.4, 7.4, 7.4 Hz, 2H), 2.04 (ddd, *J* = 7.3, 7.3, 7.3 Hz, 2H), 1.72–1.51 (m, 7H), 1.47–1.37 (m, 5H), 1.33–1.26 (m, 4H), 0.93 (t, *J* = 7.4 Hz, 3H), 0.90 (bt, *J* = 6.9 Hz, 3H). ^{13}C NMR (100 MHz, CD_3OD) δ 176.02, 175.93, 136.56, 135.74, 132.86, 132.50, 131.84, 130.62, 127.86, 127.58, 105.51, 84.44, 82.78, 82.03, 75.48, 75.01, 75.00, 74.34, 73.05, 72.76, 72.42, 72.40, 70.43, 68.64, 64.28, 52.27, 52.22, 38.25, 35.00, 34.73, 34.39, 32.95, 31.39, 30.83, 30.72, 30.19, 28.73, 27.97, 26.37, 26.21, 26.08, 24.10, 23.92, 14.72, 14.50; ^{13}C NMR (100 MHz, C_6D_6:DMSO-d_6=25:2) δ 173.57, 173.50, 136.28, 134.06, 131.44, 131.27, 131.24, 130.54, 129.43, 127.03, 104.95, 83.31, 82.04, 80.62, 74.46, 74.41, 73.95, 73.01, 72.26, 71.91, 71.57, 69.45, 68.22, 63.98, 51.14, 51.00, 37.80, 34.15, 33.98, 33.43, 31.86, 30.50, 29.86, 29.79, 29.42, 27.86, 27.02, 25.56, 25.35, 25.08, 23.18, 22.97, 14.35, 14.11; $[\alpha]_D^{20}$ = -10.4 (*c* = 0.25, $CHCl_3$); $[\alpha]_D^{20}$ = -4.8 (*c* = 0.25, CH_2Cl_2); MS (ESI) calcd for $C_{47}H_{80}O_{16}Na$ $(M+Na)^+$ 923.5, found 923.4; HRMS(ESI) calcd for $C_{47}H_{80}O_{16}Na$ $(M+Na)^+$ 923.5344, found 923.5316.

Nigricanoside A dimethyl ether **2** was made analogously with compound **16** and (*S*)-**17** according to the following scheme.

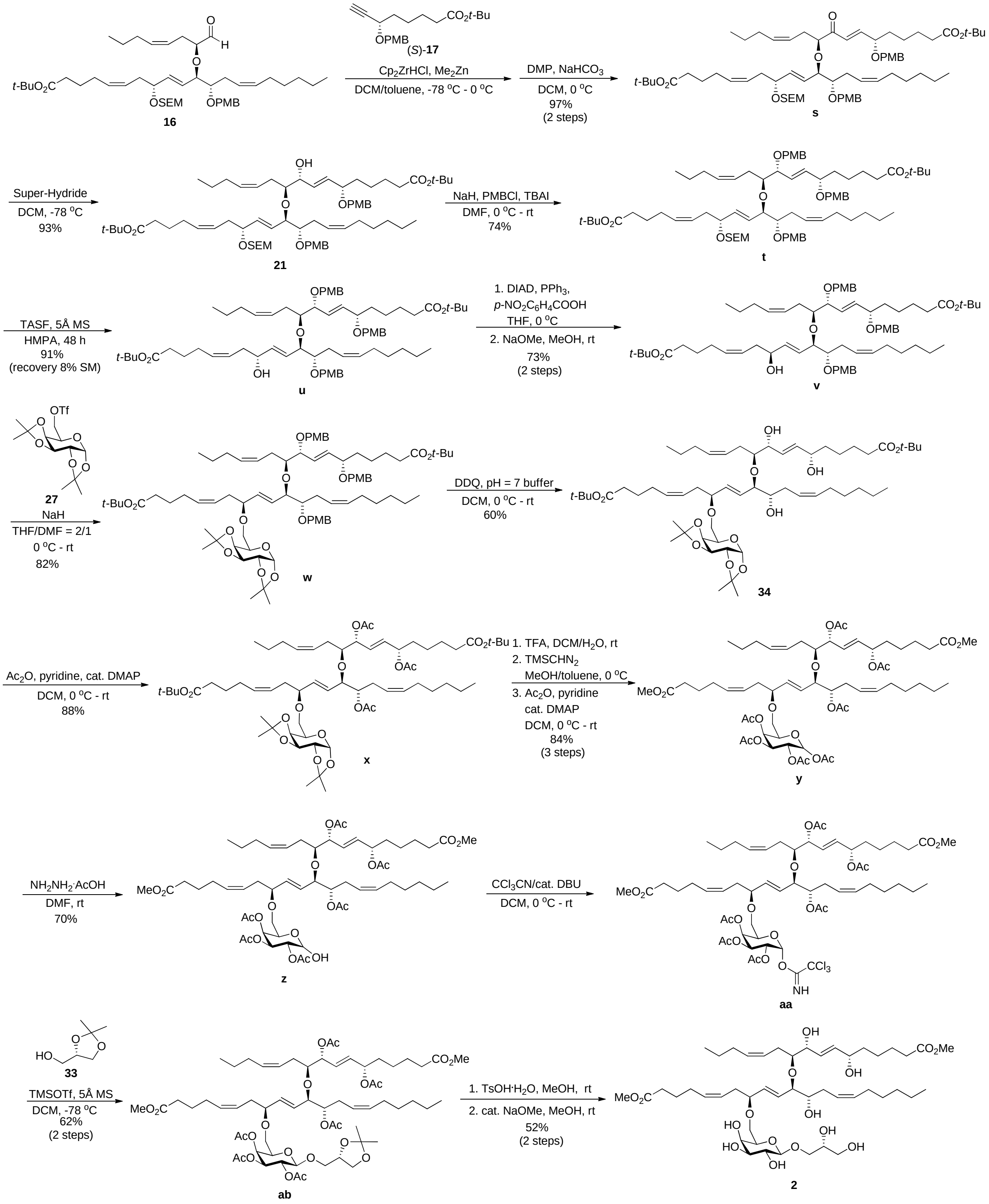

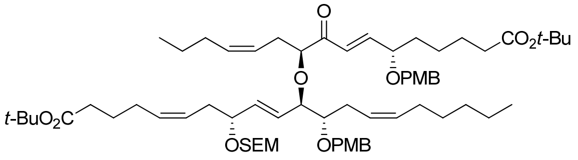

s

^{1}H NMR (400 MHz, $CDCl_3$) δ 7.29–7.16 (m, 4H), 6.94–6.79 (m, 5H), 6.68 (d, *J* = 15.8 Hz, 1H), 5.66 (dd, *J* = 15.7, 8.2 Hz, 1H), 5.53–5.26 (m, 7H), 4.70–4.39 (m, 5H), 4.21 (d, *J* = 11.2 Hz, 1H), 4.16–4.07 (m, 1H), 3.99–3.92 (m, 1H), 3.92–3.85 (m, 1H), 3.77 (s, 3H), 3.76 (s, 3H), 3.77–3.69 (m, 2H), 3.60–3.52 (m, 1H), 3.51–3.43 (m, 1H), 2.46–2.32 (m, 3H), 2.30–2.13 (m, 7H), 2.11–2.02 (m, 2H), 2.01–1.88 (m, 4H), 1.69–1.47 (m, 6H), 1.43 (s, 9H), 1.42 (s, 9H), 1.36–1.19 (m, 10H), 0.99–0.77 (m, 8H), 0.01 (s, 9H); ^{13}C NMR (100 MHz, $CDCl_3$) δ 200.82, 172.92, 172.88, 159.18, 158.96, 148.00, 135.86, 132.53, 132.02, 131.05, 130.97, 130.12, 129.26, 128.92, 125.77, 125.31, 124.72, 123.80, 113.75, 113.55, 91.81, 82.07, 81.64, 80.99, 80.00, 79.94, 77.94, 75.47, 72.48, 70.78, 65.06, 55.19, 35.35, 35.00, 34.75, 33.46, 31.50, 30.93, 29.54, 29.42, 29.22, 28.09, 28.08, 27.41, 26.81, 24.89, 24.86, 24.80, 22.63, 22.55, 18.03, 14.06, 13.78, -1.42; $[\alpha]_D^{20}$ = -13.4 (*c* = 1.0, $CHCl_3$); MS(ESI) calcd for $C_{66}H_{104}O_{12}SiNa$ $(M+Na)^+$ 1139.7, found 1139.6; HRMS(ESI) calcd for $C_{66}H_{104}O_{12}SiNa$ $(M+Na)^+$, 1139.7195, found 1139.7209.

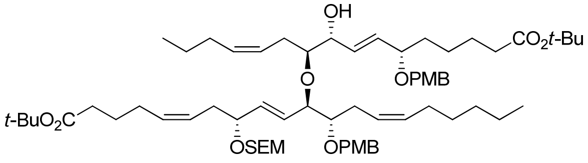

21

^{1}H NMR (500 MHz, $CDCl_3$) δ 7.29–7.20 (m, 4H), 6.88–6.82 (m, 4H), 5.69 (dd, *J* = 15.6, 6.0 Hz, 1H), 5.63–5.51 (m, 3H), 5.48–5.31 (m, 6H), 4.64 (d, *J* = 7.0 Hz, 1H), 4.60–4.50 (m, 4H), 4.25 (d, *J* = 11.4 Hz, 1H), 4.18 (d, *J* = 3.7 Hz, 1H), 4.14–4.10 (m, 1H), 4.08 (dd, *J* = 12.7, 6.4 Hz, 1H), 3.79 (s, 3H), 3.78 (s, 3H), 3.77–3.68 (m, 2H), 3.51–3.42 (m, 2H), 3.41–3.35 (m, 1H), 2.44–2.09 (m, 10H), 2.08–1.91 (m, 6H), 1.70–1.49 (m, 6H), 1.43 (s, 9H), 1.42 (s, 9H), 1.38–1.20 (ddt, *J* = 25.6, 18.6, 9.4 Hz, 10H), 0.98–0.80 (m, 8H), 0.01 (s, 9H); ^{13}C NMR (100 MHz, $CDCl_3$) δ 173.06, 172.87, 159.22, 158.95, 132.86, 132.72, 131.92, 131.60, 131.33, 131.15, 130.93, 130.84, 129.98, 129.59, 129.30, 125.90, 125.79, 125.72, 113.70, 113.64, 91.70, 82.65, 81.86, 81.50, 79.97, 79.87, 79.00, 75.40, 72.65, 72.10, 69.66, 65.03, 55.20, 55.17, 35.59, 35.49, 34.97, 33.41, 31.52, 29.50, 29.24, 29.11, 28.08, 27.43, 27.37, 26.78, 25.06, 24.98, 24.87, 22.66, 22.58, 18.03, 14.06, 13.81, -1.42; $[\alpha]_D^{20}$ = +10.0 (*c* = 1.0, $CHCl_3$); MS(ESI) calcd for $C_{66}H_{106}O_{12}SiNa$ $(M+Na)^+$ 1141.7, found 1141.7; HRMS(ESI) calcd for $C_{66}H_{106}O_{12}SiNa$ $(M+Na)^+$, 1141.7351, found 1141.7334.

The (*R*) and (*S*) Mosher's esters were synthesized from **21** and the (*S*) and (*R*) acid chloride, respectively (pyridine, DMAP, CH_2Cl_2, rt, 24 h). The resulting differences in chemical shifts are consistent with (*R*) stereochemistry at C9 as shown.

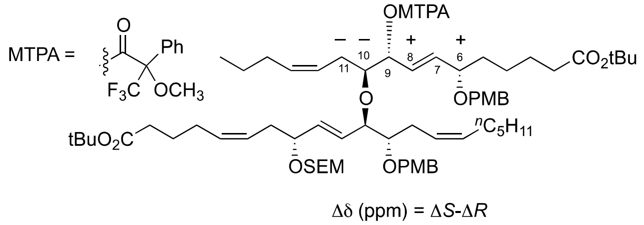

	S	*R*	Δδ (ppm)
H6	3.7491	3.6514	+0.098
H7	Overlapping signals		
H8	5.7612	5.5728	+0.188
H9	5.5826	5.7014	-0.119
H10	3.4953	3.5400	-0.045
H11	2.1283	2.2008	-0.073

Δδ (ppm) = Δ*S*-Δ*R*

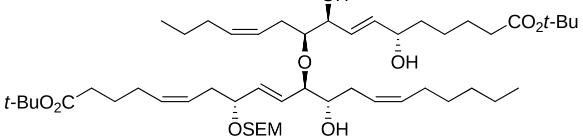

24

^{1}H NMR (600 MHz, C_6D_6:DMSO-d_6=25:2) δ 6.07–5.97 (m, 2H), 5.92 (dd, *J* = 15.6, 7.5 Hz, 1H), 5.85–5.78 (m, 1H), 5.76–5.70 (m, 1H), 5.67 (dd, *J* = 15.5, 7.3 Hz, 1H), 5.66–5.60 (m, 1H), 5.58–5.42 (m, 3H), 5.18 (d, *J* = 4.5 Hz, 1H), 4.97 (d, *J* = 5.4 Hz, 1H), 4.86 (d, *J* = 6.8 Hz, 1H), 4.65 (d, *J* = 6.8 Hz, 1H), 4.42–4.37 (m, 1H), 4.33 (d, *J* = 4.6 Hz, 1H),

4.28–4.20 (m, 3H), 4.01–3.92 (m, 1H), 3.90–3.80 (m, 1H), 3.60–3.50 (m, 2H), 2.63–2.34 (m, 6H), 2.23–2.02 (m, 10H), 1.72–1.42 (m, 10H), 1.38 (s, 9H), 1.36 (s, 9H), 1.31–1.21 (m, 6H), 0.96 (t, J = 8.1 Hz, 2H), 0.91 (t, J = 7.4 Hz, 3H), 0.87 (t, J = 6.9 Hz, 3H), 0.00 (s, 9H).

Analytical sample of **25** (anti, syn) was prepared by deprotection of **t** with DDQ in phosphate buffer (pH = 7) and DCM.

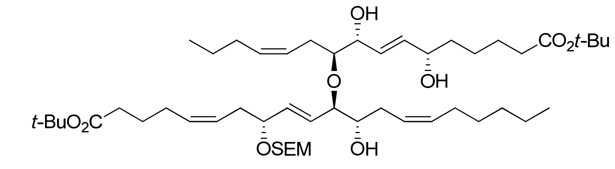

25

^{1}H NMR (600 MHz, C_6D_6:DMSO-d_6=25:2) δ 6.05 (dd, J = 15.5, 6.5 Hz, 1H), 5.96 (dd, J = 15.6, 6.1 Hz, 1H), 5.84 (dd, J = 15.3, 6.8 Hz, 1H), 5.87–5.81 (m, 1H), 5.68 (dd, J = 15.6, 7.5 Hz, 1H), 5.65–5.58 (m, 2H), 5.58–5.41 (m, 3H), 5.18 (d, J = 4.6 Hz, 1H), 4.92 (d, J = 6.2 Hz, 1H), 4.84 (d, J = 6.8 Hz, 1H), 4.65 (d, J = 6.8 Hz, 1H), 4.49–4.44 (m, 1H), 4.34 (d, J = 4.4 Hz, 1H), 4.28–4.18 (m, 3H), 3.94–3.88 (m, 1H), 3.84 (dd, J = 16.9, 8.9 Hz, 1H), 3.71–3.66 (m, 1H), 3.53 (dd, J = 16.7, 8.9 Hz, 1H), 2.62–2.28 (m, 6H), 2.19–1.99 (m, 10H), 1.70–1.42 (m, 10H), 1.38 (s, 9H), 1.36 (s, 9H), 1.31–1.22 (m, 6H), 0.96 (t, J = 8.1 Hz, 2H), 0.89 (t, J = 7.3 Hz, 3H), 0.87 (t, J = 7.1 Hz, 3H), -0.00 (s, 9H).

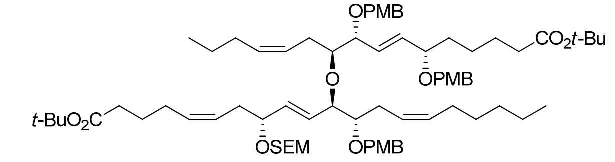

t

^{1}H NMR (400 MHz, $CDCl_3$) δ 7.24–7.18 (m, 6H), 6.87–6.78 (m, 6H), 5.73 (dd, J = 15.7, 8.0 Hz, 1H), 5.67 (dd, J = 15.6, 8.1 Hz, 1H), 5.54 (dd, J = 14.8, 7.0 Hz, 1H), 5.49 (dd, J = 15.6, 7.4 Hz, 1H), 5.45–5.31 (m, 6H), 4.69–4.60 (m, 2H), 4.57–4.43 (m, 4H), 4.30 (d, J = 11.5 Hz, 1H), 4.26 (d, J = 11.3 Hz, 1H), 4.15 (dd, J = 8.0, 3.7, Hz, 1H), 4.15–4.07 (m, 1H), 3.79 (s, 3H), 3.78 (s, 3H), 3.84–3.69 (m, 3H), 3.77 (s, 3H), 3.62–3.56 (m, 1H), 3.54–3.40 (m, 2H), 2.43–2.03 (m, 12H), 2.00–1.90 (m, 4H), 1.66–1.50 (m, 6H), 1.43 (s, 9H), 1.42 (s, 9H), 1.37–1.07 (m, 10H), 0.87 (t, J = 7.3 Hz, 3H), 0.86 (t, J = 6.9 Hz, 3H), 0.01 (s, 9H); ^{13}C NMR (100 MHz, $CDCl_3$) δ 173.00, 172.92, 159.03, 158.95, 158.86, 135.82, 134.50, 131.55, 131.27, 131.24, 131.22, 130.80, 130.70, 130.66, 129.93, 129.24, 129.21, 128.96, 126.04, 125.97, 125.91, 113.72, 113.65, 113.49, 91.69, 82.33, 82.05, 81.17, 79.95, 79.90, 79.15, 78.57, 75.60, 72.26, 70.07, 69.89, 65.00, 55.21, 55.18, 35.60, 35.49, 35.00, 33.57, 31.54, 30.10, 29.66, 29.58, 29.29, 28.09, 28.08, 27.44, 26.82, 25.10, 25.00, 24.90, 22.71, 22.58, 18.04, 14.07, 13.84, -1.41; $[\alpha]_D^{20}$ = -15.4 (c = 1.0, $CHCl_3$); MS(ESI) calcd for $C_{74}H_{114}O_{13}SiNa$ $(M+Na)^+$ 1261.8, found 1261.7; HRMS(ESI) calcd for $C_{74}H_{114}O_{13}SiNa$ $(M+Na)^+$, 1261.7926, found 1261.7942.

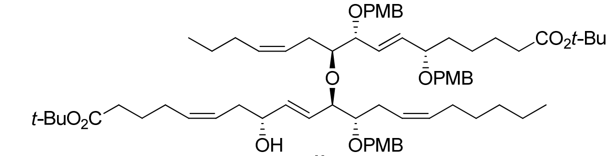

u

^{1}H NMR (400 MHz, $CDCl_3$) δ 7.25–7.19 (m, 6H), 6.87–6.79 (m, 6H), 5.73 (dd, J = 15.9, 8.0 Hz, 1H), 5.73–5.66 (m, 2H), 5.55 (dd, J = 15.7, 7.8 Hz, 1H), 5.53–5.31 (m, 6H), 4.65 (d, J = 11.3 Hz, 1H), 4.58–4.46 (m, 3H), 4.31 (d, J = 11.6 Hz, 1H), 4.27 (d, J = 11.4 Hz, 1H), 4.20–4.11 (m, 2H), 3.79 (s, 3H), 3.78 (s, 3H), 3.85–3.70 (m, 2H), 3.77 (s, 3H), 3.63–3.56 (m, 1H), 3.56–3.49 (m, 1H), 2.37–2.12 (m, 10H), 2.12–2.05 (m, 2H), 2.00–1.91 (m, 4H), 1.70–1.48 (m, 6H), 1.43 (s, 9H), 1.42 (s, 9H), 1.38–1.18 (m, 10H), 0.92–0.79 (m, 6H); ^{13}C NMR (100 MHz, $CDCl_3$) δ ^{13}C NMR (100 MHz, $CDCl_3$) δ 173.00, 172.99, 159.02, 158.95, 158.92, 137.20, 135.79, 132.06, 131.58, 131.27, 131.21, 130.68, 130.63, 129.94, 129.35, 129.20, 128.98, 128.64, 126.03, 125.87, 125.55, 113.72, 113.64, 113.52, 82.41, 81.93, 81.29, 80.06, 79.92, 79.13, 78.64, 72.47, 71.72, 70.08, 69.89, 55.21, 55.19, 35.58, 35.48, 35.14, 34.92, 31.53, 30.06, 29.82, 29.57, 29.28, 28.07, 27.47, 26.72, 25.08, 24.99, 24.90, 22.71, 22.57, 14.06, 13.84; $[\alpha]_D^{20}$ = -39.6 (c = 1.0, $CHCl_3$); MS(ESI) calcd for $C_{68}H_{100}O_{12}Na$ $(M+Na)^+$ 1131.7, found 1131.6; HRMS(ESI) calcd for $C_{68}H_{100}O_{12}Na$ $(M+Na)^+$, 1131.7112, found 1131.7108.

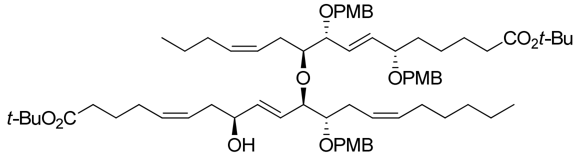
v

The compound **u** (77.7 mg, 0.07 mmol, 1.0 equiv) and 4-nitrobenzoic acid (17.6 mg, 0.105 mmol, 1.5 equiv) were dissolved in 1.0 mL DCM and cooled to 0 ºC DIAD (diisopropyl azodicarboxylate) (21 μL, 0.105 mmol, 1.5 equiv) was added slowly and the resulting solution was allowed to stir for 1 h at 0 ºC. Then the reaction was concentrated and purified by flash chromatography (5-25% EtOAc in hexanes) to afford the desired ester as a colorless oil The ester was dissolved in 1.0 mL MeOH, treated with NaOMe (0.5 M in MeOH, 50 μL), and the resulting solution was stirred overnight at room temperature. Then the reaction was concentrated and purified by flash chromatography (5-20% EtOAc in hexanes) to afford the desired alcohol (56.9 mg, 73% over two steps) as a colorless oil ^{1}H NMR (400 MHz, $CDCl_3$) δ 7.25–7.18 (m, 6H), 6.90–6.77 (m, 6H), 5.73 (dd, J = 15.7, 8.0 Hz, 1H), 5.73–5.66 (m, 2H), 5.55 (dd, J = 15.8, 7.9 Hz, 1H), 5.54–5.31 (m, 6H), 4.66 (d, J = 11.2 Hz, 1H), 4.56–4.44 (m, 3H), 4.31 (d, J = 11.5 Hz, 1H), 4.27 (d, J = 11.4 Hz, 1H), 4.21–4.13 (m, 1H), 4.13–4.08 (m, 1H), 3.79 (s, 3H), 3.84–3.72 (m, 2H), 3.78 (s, 3H), 3.77 (s, 3H), 3.66–3.59 (m, 1H), 3.56–3.49 (m, 1H), 2.36–2.12 (m, 10H), 2.12–2.03 (m, 2H), 2.02–1.91 (m, 4H), 1.70–1.51 (m, 6H), 1.43 (s, 9H), 1.42 (s, 9H), 1.40–1.19 (m, 10H), 0.87 (t, J = 7.3 Hz, 3H), 0.85 (t, J = 7.0 Hz, 3H); ^{13}C NMR (100 MHz, $CDCl_3$) δ 173.02, 172.99, 159.03, 158.96, 158.92, 137.18, 135.77, 132.01, 131.57, 131.24, 130.69, 130.65, 129.97, 129.35, 129.21, 129.01, 128.57, 126.19, 125.86, 125.54, 113.72, 113.65, 113.51, 82.39, 81.86, 81.23, 80.05, 79.93, 79.14, 78.71, 72.49, 71.74, 70.09, 69.88, 55.21, 35.56, 35.48, 35.13, 34.92, 31.54, 30.02, 29.84, 29.57, 29.28, 28.08, 27.46, 26.72, 25.07, 24.99, 24.91, 22.70, 22.57, 14.06, 13.84; $[\alpha]_D^{20}$ = -52.4 (c = 1.0, $CHCl_3$); MS(ESI) calcd for $C_{68}H_{100}O_{12}Na$ $(M+Na)^+$ 1131.7, found 1131.7; HRMS(ESI) calcd for $C_{68}H_{100}O_{12}Na$ $(M+Na)^+$, 1131.7112, found 1131.7049.

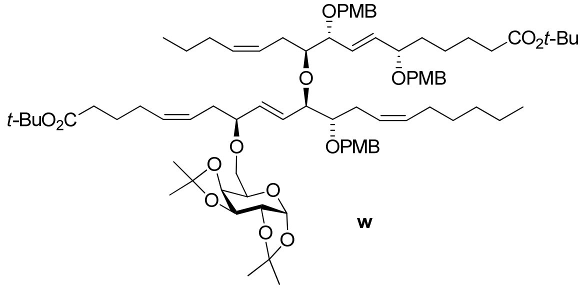
w

^{1}H NMR (400 MHz, $CDCl_3$) δ 7.25–7.17 (m, 6H), 6.87–6.77 (m, 6H), 5.75 (dd, J = 15.8, 8.0 Hz, 1H), 5.69 (dd, J = 15.8, 8.5 Hz, 1H), 5.58–5.47 (m, 3H), 5.45–5.30 (m, 6H), 4.66 (d, J = 11.3 Hz, 1H), 4.57–4.44 (m, 4H), 4.35–4.14 (m, 5H), 3.96–3.88 (m, 1H), 3.79 (s, 3H), 3.77 (s, 3H), 3.76 (s, 3H), 3.85–3.69 (m, 4H), 3.69–3.61 (m, 1H), 3.53–3.44 (m, 2H), 2.47–2.08 (m, 10H), 2.08–1.86 (m, 6H), 1.64–1.46 (m, 6H), 1.54 (s, 3H), 1.43 (s, 9H), 1.42 (s, 9H), 1.39 (s, 3H), 1.33 (s, 3H), 1.40–1.19 (m, 10H), 1.28 (s, 3H), 0.86 (t, J = 7.4 Hz, 3H), 0.85 (t, J = 6.9 Hz, 3H); ^{13}C NMR (100 MHz, $CDCl_3$) δ 172.98, 159.01, 158.93, 158.86, 135.78, 135.20, 131.47, 131.32, 131.22, 130.72, 130.66, 130.49, 129.94, 129.26, 129.20, 128.98, 126.07, 126.02, 125.99, 113.71, 113.63, 113.49, 109.02, 108.39, 96.30, 82.62, 82.16, 81.36, 80.56, 79.90, 79.89, 79.17, 78.52, 72.37, 71.17, 70.59, 70.57, 70.06, 69.87, 67.35, 67.24, 55.20, 55.17, 35.60, 35.49, 35.00, 33.55, 31.52, 30.29, 29.75, 29.59, 29.28, 28.08, 27.43, 26.80, 26.10, 25.93, 25.10, 25.00, 24.95, 24.91, 24.33, 22.71, 22.57, 14.06, 13.85; $[\alpha]_D^{20}$ = -61.2 (c = 1.0, $CHCl_3$); MS(ESI) calcd for $C_{80}H_{118}O_{17}Na$ $(M+Na)^+$ 1373.8, found 1373.8; HRMS(ESI) calcd for $C_{80}H_{118}O_{17}Na$ $(M+Na)^+$, 1373.8267, found 1373.8197.

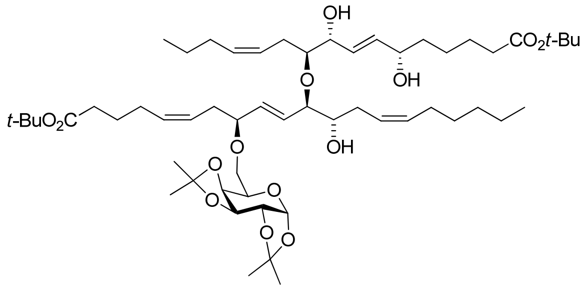
34

^{1}H NMR (400 MHz, $CDCl_3$) δ 5.78–5.75 (m, 2H), 5.64–5.59 (m, 2H), 5.64–5.59 (m, 1H), 5.51 (d, J = 4.9 Hz, 1H), 5.49–5.31 (m, 5H), 4.58 (dd, J = 8.0, 2.3 Hz, 1H), 4.29 (dd, J = 5.0, 2.3 Hz, 1H), 4.25 (dd, J = 7.9, 1.7 Hz, 1H), 4.22–4.18 (m, 1H), 4.17–4.11 (m, 1H), 4.08–4.04 (m, 1H), 3.93 (t, J = 5.4 Hz, 1H), 3.87–3.81 (m, 1H), 3.69 (dd, J = 10.3, 6.2 Hz, 1H), 3.73–3.62 (m, 1H), 3.57–3.51 (m, 1H), 3.47 (dd, J = 10.3, 6.2 Hz, 1H), 2.45–2.10 (m, 10H), 2.09–1.92 (m, 6H), 1.67–1.48 (m, 6H), 1.54 (s, 3H), 1.44 (s, 9H), 1.43 (s, 9H), 1.41 (s, 3H), 1.33 (s, 3H), 1.32 (s, 3H), 1.39–1.24 (m, 10H), 0.89 (t, J = 7.4 Hz, 3H), 0.88 (t, J = 6.9 Hz, 3H); ^{1}H NMR (600 MHz, C_6D_6:DMSO-d_6=25:2) δ 6.05 (dd, J = 15.6, 6.4 Hz, 1H), 5.96 (dd, J = 15.5, 6.1 Hz, 1H), 5.82 (dd, J = 15.9, 7.1 Hz, 1H), 5.78–5.83 (m, 1H), 5.68 (dd, J = 15.8, 7.4 Hz, 1H), 5.68–5.62 (m, 1H), 5.60–5.50 (m, 3H), 5.50 (d, J = 5.0 Hz, 1H), 5.43–5.35 (m, 1H), 5.17 (d, J = 4.5 Hz, 1H), 4.84 (d, J = 6.1 Hz, 1H), 4.54 (dd, J = 7.9, 1.9 Hz, 1H), 4.49–4.43 (m, 1H), 4.36 (d, J = 4.4 Hz, 1H), 4.27–4.18 (m, 4H), 4.19–4.14 (m, 1H), 3.96 (dd, J = 10.0, 6.1 Hz, 1H), 3.92–3.80 (m, 2H), 3.74–3.69 (m, 1H), 3.67 (dd, J = 9.9, 6.3 Hz, 1H), 2.61–2.25 (m, 6H), 2.19–1.95 (m, 10H), 1.66–1.50 (m, 8H), 1.49 (s, 3H), 1.44 (s, 3H), 1.40–1.23 (m, 8H), 1.38 (s, 9H), 1.36 (s, 9H), 1.23 (s, 3H), 1.08 (s,

3H), 0.96–0.81 (m, 6H); ^{13}C NMR (100 MHz, $CDCl_3$) δ 173.08, 173.01, 135.86, 134.65, 133.17, 131.78, 130.75, 129.62, 128.72, 125.80, 125.57, 125.20, 109.12, 108.46, 96.29, 82.49, 81.06, 80.21, 80.02, 73.62, 73.26, 72.00, 71.12, 70.57, 70.53, 67.20, 67.17, 36.74, 35.41, 34.98, 33.45, 31.48, 29.49, 29.26, 29.21, 28.07, 27.38, 26.77, 26.07, 25.92, 24.94, 24.91, 24.88, 24.34, 22.67, 22.55, 14.05, 13.83; $[\alpha]_D^{20}$ = -27.0 (*c* = 1.0, $CHCl_3$); MS(ESI) calcd for $C_{56}H_{94}O_{14}Na$ $(M+Na)^+$ 1013.7, found 1013.7; HRMS(ESI) calcd for $C_{56}H_{94}O_{14}Na$ $(M+Na)^+$, 1013.6541, found 1013.6533.

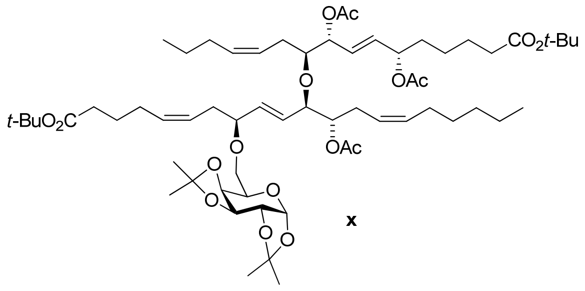

x

^{1}H NMR (400 MHz, $CDCl_3$) δ 5.76 (dd, *J* = 15.6, 6.9 Hz, 1H), 5.65 (dd, *J* = 15.6, 6.2 Hz, 1H), 5.58–5.50 (m, 3H), 5.50–5.37 (m, 4H), 5.35–5.21 (m, 4H), 4.88 (dd, *J* = 11.3, 6.7 Hz, 1H), 4.58 (dd, *J* = 8.0, 2.3 Hz, 1H), 4.29 (dd, *J* = 5.0, 2.3 Hz, 1H), 4.23 (dd, *J* = 7.9, 1.7 Hz, 1H), 4.10–4.03 (m, 1H), 3.92 (t, *J* = 5.1 Hz, 1H), 3.84 (d, *J* = 6.2 Hz, 1H), 3.67 (dd, *J* = 10.4, 5.5 Hz, 1H), 3.54 (d, *J* = 5.0 Hz, 1H), 3.44 (dd, *J* = 10.6, 6.6 Hz, 1H), 2.40–2.29 (m, 3H), 2.24–2.16 (m, 5H), 2.07 (s, 3H), 2.05 (s, 3H), 2.15–1.90 (m, 8H), 2.01 (s, 3H), 1.67–1.51 (m, 6H), 1.54 (s, 3H), 1.43 (s, 9H), 1.42 (s, 9H), 1.41 (s, 3H), 1.33 (s, 3H), 1.38–1.23 (m, 10H), 1.31 (s, 3H), 0.89 (t, *J* = 7.4 Hz, 3H), 0.88 (t, *J* = 6.8 Hz, 3H); ^{13}C NMR (100 MHz, $CDCl_3$) δ 172.92, 172.78, 170.33, 170.05, 169.74, 135.97, 133.23, 132.73, 131.86, 130.76, 129.56, 126.27, 125.75, 125.00, 124.36, 109.06, 108.40, 96.29, 80.42, 80.16, 80.00, 79.93, 77.46, 75.49, 75.11, 73.34, 71.17, 70.56, 70.55, 67.45, 67.36, 35.31, 34.97, 34.07, 33.42, 31.47, 29.62, 29.44, 29.23, 28.08, 28.06, 27.81, 27.29, 26.80, 26.08, 25.92, 24.93, 24.87, 24.78, 24.63, 24.33, 22.64, 22.57, 21.20, 21.12, 14.04, 13.80; $[\alpha]_D^{20}$ = -47.6 (*c* = 1.0, $CHCl_3$); MS(ESI) calcd for $C_{62}H_{100}O_{17}Na$ $(M+Na)^+$ 1139.7, found 1139.5

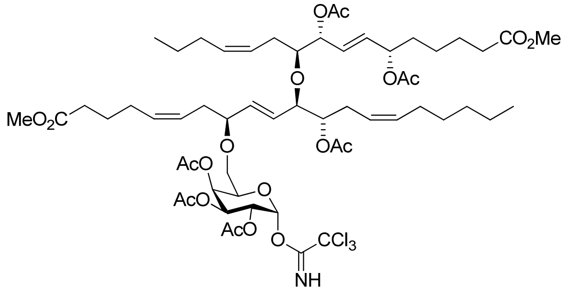

aa

^{1}H NMR (500 MHz, C_6D_6) δ 8.54 (s, 1H), 6.06 (dd, *J* = 15.9, 7.1 Hz, 1H), 6.02–5.97 (m, 1H), 5.87 (dd, *J* = 10.9, 3.1 Hz, 1H), 5.81–5.72 (m, 2H), 5.71–5.35 (m, 10H), 5.34–5.27 (m, 1H), 4.63 (t, *J* = 5.9 Hz, 1H), 4.34 (dd, *J* = 7.3, 4.7 Hz, 1H), 3.83–3.76 (m, 1H), 3.76–3.63 (m, 2H), 3.52 (dd, *J* = 10.0, 6.0 Hz, 1H), 3.44–3.31 (m, 1H), 3.39 (s, 3H), 3.35 (s, 3H), 2.71–2.53 (m, 2H), 2.49–2.33 (m, 4H), 2.26–1.97 (m, 10H), 1.92 (s, 3H), 1.91 (s, 3H), 1.82 (s, 3H), 1.78 (s, 3H), 1.76 (s, 3H), 1.67–1.62 (m, 2H), 1.60 (s, 3H), 1.53–1.18 (m, 14H), 0.97 (t, *J* = 7.3 Hz, 3H), 0.91 (t, *J* = 6.8 Hz, 3H).

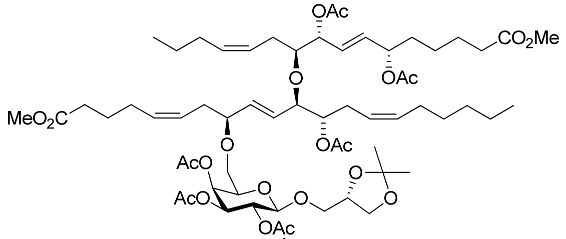

ab

^{1}H NMR (600 MHz, C_6D_6) δ 6.06 (dd, *J* = 15.7, 7.1 Hz, 1H), 5.77 (dd, *J* = 15.7, 6.4 Hz, 1H), 5.73–5.63 (m, 5H), 5.63–5.53 (m, 5H), 5.53–5.46 (m, 1H), 5.42–5.36 (m, 1H), 5.34–5.30 (m, 1H), 5.27 (dd, *J* = 10.5, 3.4 Hz, 1H), 4.43 (d, *J* = 8.0 Hz, 1H), 4.35 (dd, *J* = 7.5, 4.7 Hz, 1H), 4.15–4.09 (m, 1H), 3.90 (dd, *J* = 10.4, 3.9 Hz, 1H), 3.86 (dd, *J* = 8.2, 5.9 Hz, 1H), 3.80 (dd, *J* = 7.9, 6.8 Hz, 2H), 3.72–3.66 (m, 1H), 3.65–3.61 (m, 2H), 3.58 (dd, *J* = 10.4, 6.5 Hz, 1H), 3.51–3.43 (m, 1H), 3.41 (s, 3H), 3.35 (s, 3H), 2.69–2.56 (m, 2H), 2.48–2.35 (m, 3H), 2.30–2.23 (m, 1H), 2.23–2.18 (m, 2H), 2.16–2.11 (m, 2H), 2.11–2.01 (m, 6H), 1.93 (s, 3H), 1.82 (s, 3H), 1.81 (s, 3H), 1.783 (s, 3H), 1.780 (s, 3H), 1.76 (s, 3H), 1.70–1.65 (m, 2H), 1.58–1.25 (m, 14H), 1.43 (s, 3H), 1.26 (s, 3H), 0.95 (t, *J* = 7.4 Hz, 3H), 0.91 (t, *J* = 6.8 Hz, 3H).

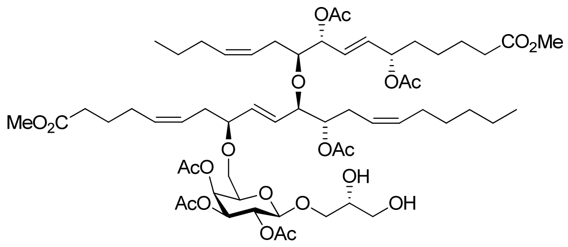

^{1}H NMR (500 MHz, C_6D_6) δ 6.05 (dd, *J* = 15.6, 7.0 Hz, 1H), 5.76 (dd, *J* = 15.5, 5.6 Hz, 1H), 5.74–5.52 (m, 10H), 5.52–5.43 (m, 1H), 5.42–5.36 (m, 1H), 5.35–5.29 (m, 1H), 5.26 (dd, *J* = 10.5, 3.4 Hz, 1H), 4.36 (d, *J* = 8.0 Hz, 1H), 4.37–4.32 (m, 1H), 3.92–3.86 (m, 1H), 3.81–3.71 (m, 4H), 3.71–3.66 (m, 1H), 3.64–3.61 (m, 1H), 3.59–3.53 (m, 2H), 3.51–3.43 (m, 2H), 3.40 (s, 3H), 3.35 (s, 3H), 3.26 (s, 1H), 2.68–2.59 (m, 2H), 2.54–2.44 (m, 2H), 2.34–2.27 (m, 2H), 2.22–2.02 (m, 10H), 1.92 (s, 3H), 1.83 (s, 3H), 1.78 (s, 3H), 1.77 (s, 3H), 1.75 (s, 3H), 1.74 (s, 3H), 1.70–1.61 (m, 2H), 1.56–1.19 (m, 14H), 0.95 (t, *J* = 7.4 Hz, 3H), 0.91 (t, *J* = 7.1 Hz, 3H).

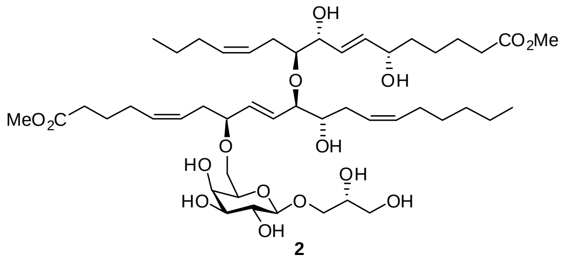

2

^{1}H NMR (600 MHz, C_6D_6:DMSO-d_6=25:2) δ 6.11 (dd, *J* = 15.5, 6.3 Hz, 1H), 5.99 (dd, *J* = 15.6, 5.8 Hz, 1H), 5.88 (dd, *J* = 15.7, 7.4 Hz, 1H), 5.84–5.77 (m, 1H), 5.74 (dd, *J* = 15.7, 7.5 Hz, 1H), 5.71–5.65 (m, 1H), 5.62–5.57 (m, 1H), 5.58–5.51 (m, 2H), 5.45–5.38 (m, 1H), 5.29 (d, *J* = 3.2 Hz, 1H), 5.10 (d, *J* = 4.7 Hz, 1H), 4.82 (d, *J* = 5.5 Hz, 1H), 4.81 (d, *J* = 5.6 Hz, 1H), 4.72 (d, *J* = 5.6 Hz, 1H), 4.60 (d, *J* = 4.6 Hz, 1H), 4.51–4.47 (m, 1H), 4.44 (t, *J* = 6.0 Hz, 1H), 4.41 (d, *J* = 7.7 Hz, 1H), 4.33 (d, *J* = 4.1 Hz, 1H), 4.28–4.21 (m, 2H), 4.15 (dd, *J* = 10.5, 5.6 Hz, 1H), 4.11–4.06 (m, 1H), 4.05–4.02 (m, 1H), 4.01 (dd, *J* = 9.5, 6.3 Hz, 1H), 3.99–3.95 (m, 2H), 3.93 (dd, *J* = 10.3, 3.9 Hz, 1H), 3.91–3.84 (m, 2H), 3.84–3.79 (m, 1H), 3.80–3.76 (m, 1H), 3.72 (dd, *J* = 9.5, 6.0 Hz, 1H), 3.71–3.66 (m, 1H), 3.65 (bt, *J* = 5.9 Hz, 1H), 3.42 (s, 3H), 3.38 (s, 3H), 2.62–2.41 (m, 5H), 2.35–2.28 (m, 1H), 2.20–2.13 (m, 6H), 2.11 (ddd, *J* = 7.3, 7.3, 7.3 Hz, 2H), 2.04 (ddd, *J* = 7.4, 7.4, 7.4 Hz, 2H), 1.70–1.50 (m, 7H), 1.48–1.34 (m, 2H), 1.40 (qdd, *J* = 7.4, 7.4, 7.4 Hz, 2H), 1.32–1.23 (m, 5H), 0.92 (t, *J* = 7.4 Hz, 3H), 0.88 (bt, *J* = 6.8 Hz, 3H). ^{13}C NMR (100 MHz, C_6D_6:DMSO-d_6=25:2) δ 173.58, 173.44, 136.29, 134.70, 131.32, 131.28, 130.52, 129.35, 127.51, 126.99, 105.10, 83.08, 81.73, 80.84, 74.51, 73.83, 73.20, 72.39, 71.99, 71.59, 71.48, 69.22, 68.07, 64.07, 51.10, 51.00, 37.84, 34.17, 33.39, 31.85, 30.67, 29.88, 29.78, 29.47, 27.86, 26.99, 25.57, 25.37, 25.06, 23.18, 22.96, 14.32, 14.13; $[\alpha]_D^{20}$ = -22.0 (*c* = 0.1, CH_2Cl_2); MS (ESI) calcd for $C_{47}H_{80}O_{16}Na^+$ $(M+Na)^+$ 923.5, found 923.4; HRMS(ESI) calcd for $C_{47}H_{80}O_{16}Na$ $(M+Na)^+$, 923.5344, found 923.5331.

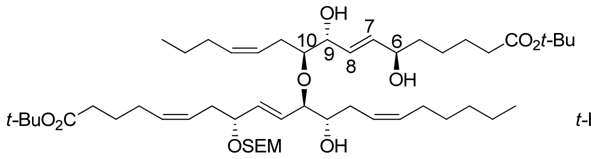

23 (anti, anti)

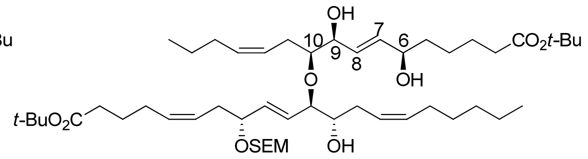

22 (syn, syn)

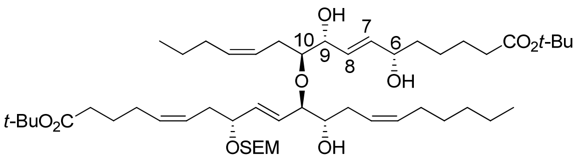

25 (anti, syn)

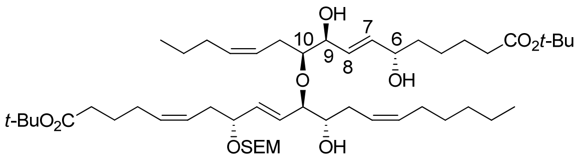

24 (syn, anti)

	Natural pdt **2**	**23** (anti, anti)	**22** (syn, syn)[a]	**25** (anti, syn)	**24** (syn, anti)
6	4.25	4.23	4.22	4.22	4.23
7	6.00	6.00	5.91	5.96	6.01
	dd J = 15.5, 5.5		dd J = 15.9, 6.0	dd J = 15.6, 6.1	
8	6.11	6.03	6.02	6.05	6.04
	dd J = 15.5, 6.4		dd J = 14.7, 6.3	dd J = 15.5, 6.5	
9	4.50	4.50	4.34	4.46	4.39
10	3.78	3.66	3.53	3.68	3.57

[a] The ^{1}H NMR of this compound **22** (syn, syn) was assigned by selective TOCSY experiment

Figure S6. Comparison of 4 diasteromeric triols (**22**-**25**) to nigricanoside A dimethyl ester **2**

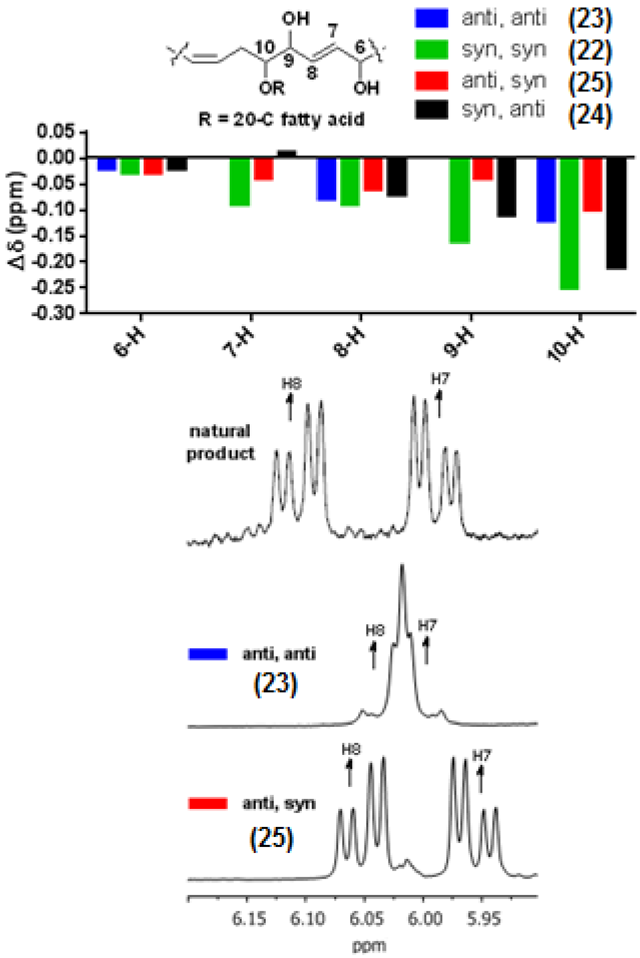

Figure S7. Comparison of 4 diasteromeric triols (**22**-**25**) to nigricanoside A dimethyl ester **2**

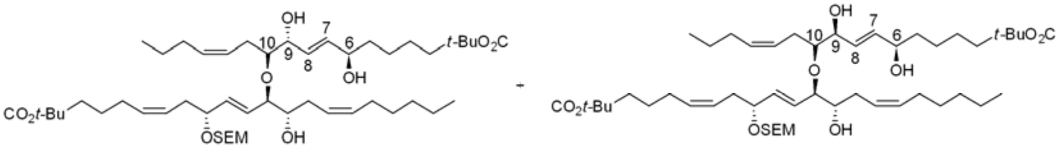

23 (anti, anti) **22** (syn, syn)

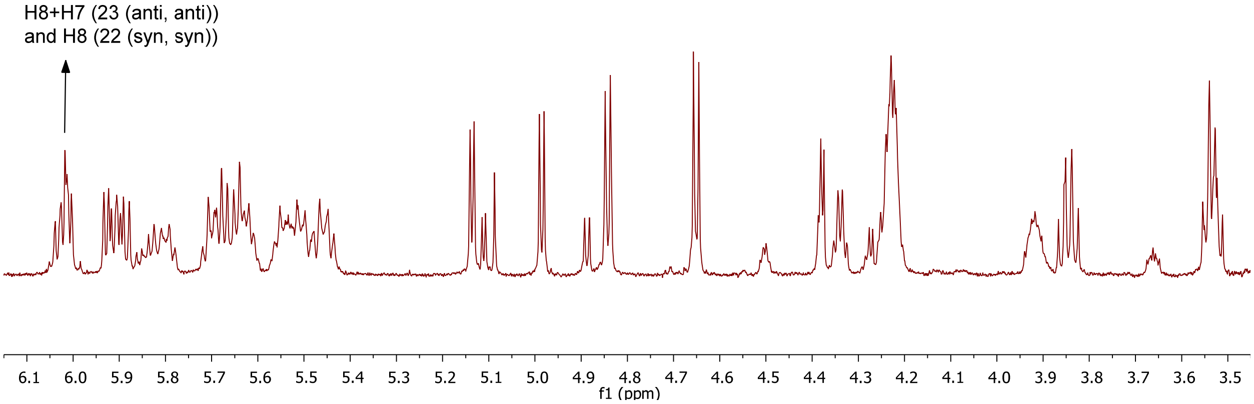

Selective irradiation at 6.01 ppm

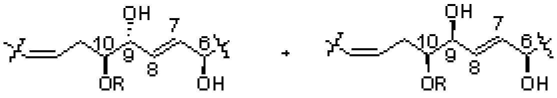

23 (anti, anti) 22 (syn, syn)

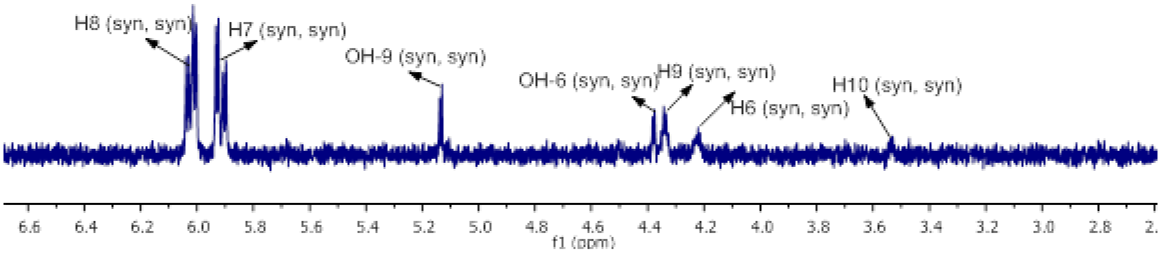

Figure S8. Selective TOCSY experiment of the mixture (**23** (anti, anti) and **22** (syn, syn))

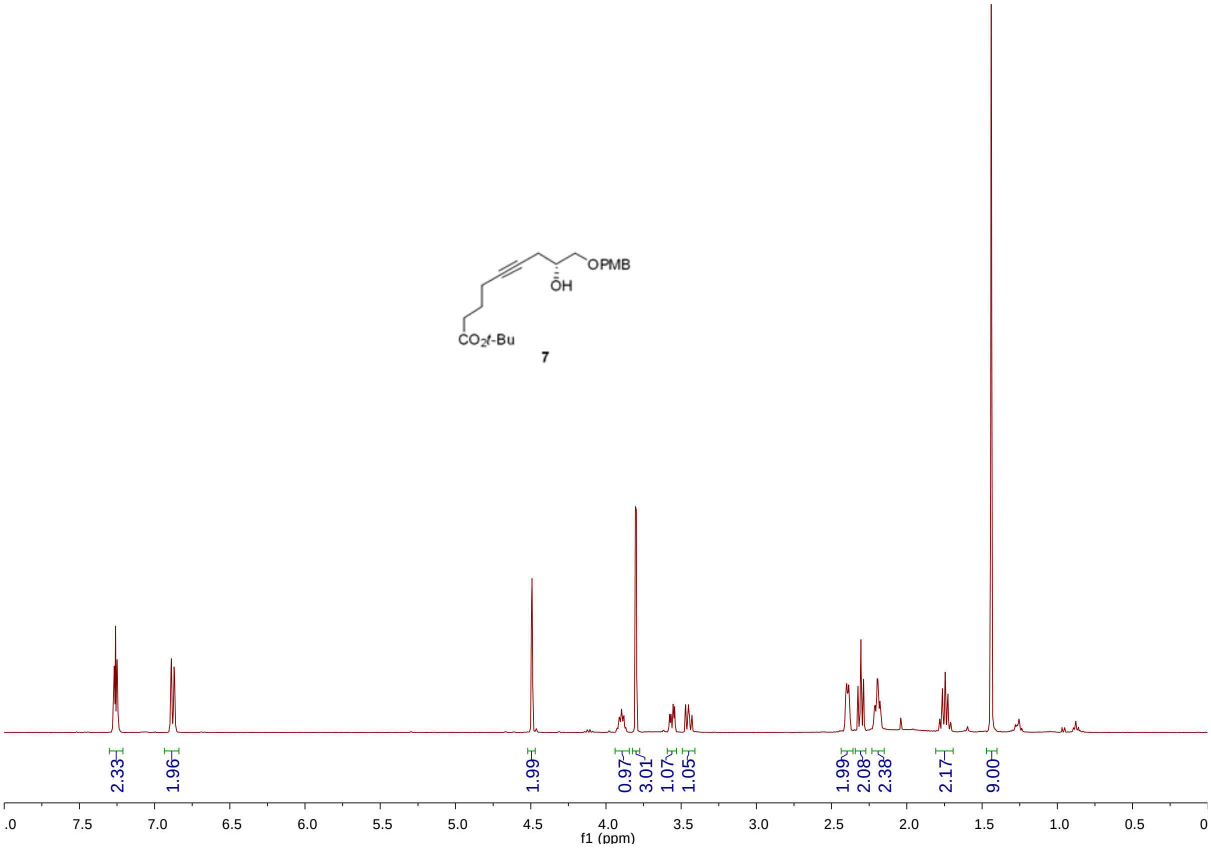
OPMB
OH
CO2t-Bu
7
2.33
1.96
1.99
0.97
3.01
1.07
1.05
1.99
2.08
2.38
2.17
9.00
.0
7.5
7.0
6.5
6.0
5.5
5.0
4.5
4.0
3.5
3.0
2.5
2.0
1.5
1.0
0.5
0
f1 (ppm)

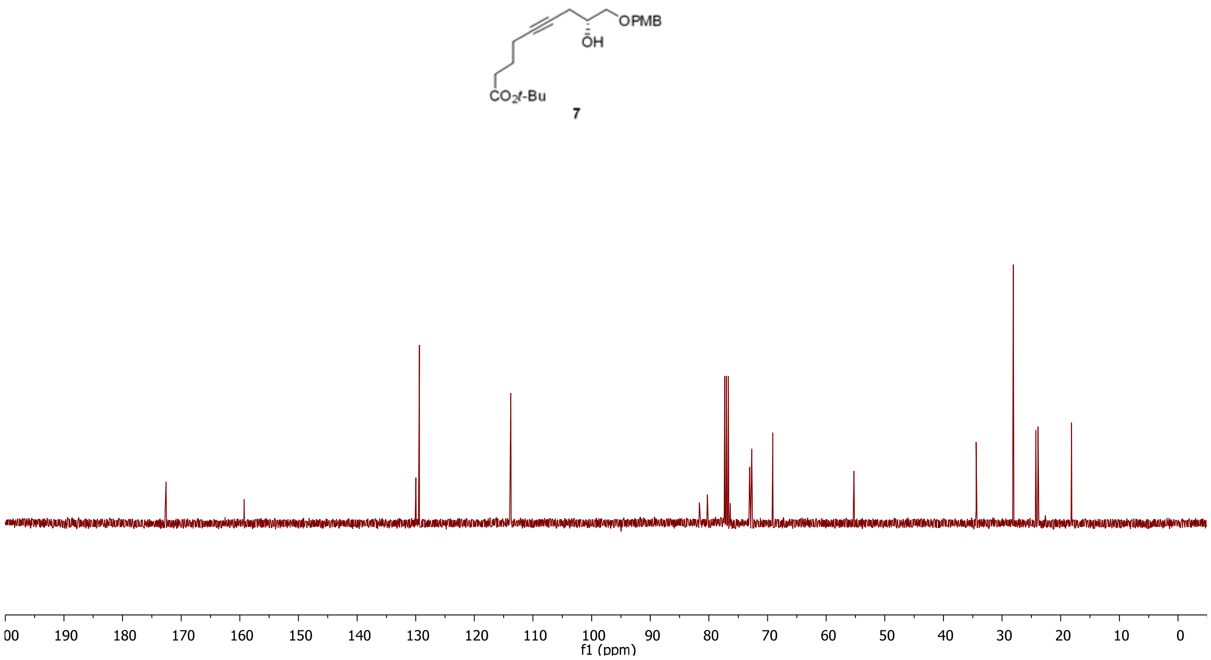
OPMB
OH
CO2t-Bu
7
00
190
180
170
160
150
140
130
120
110
100
90
80
70
60
50
40
30
20
10
0
f1 (ppm)

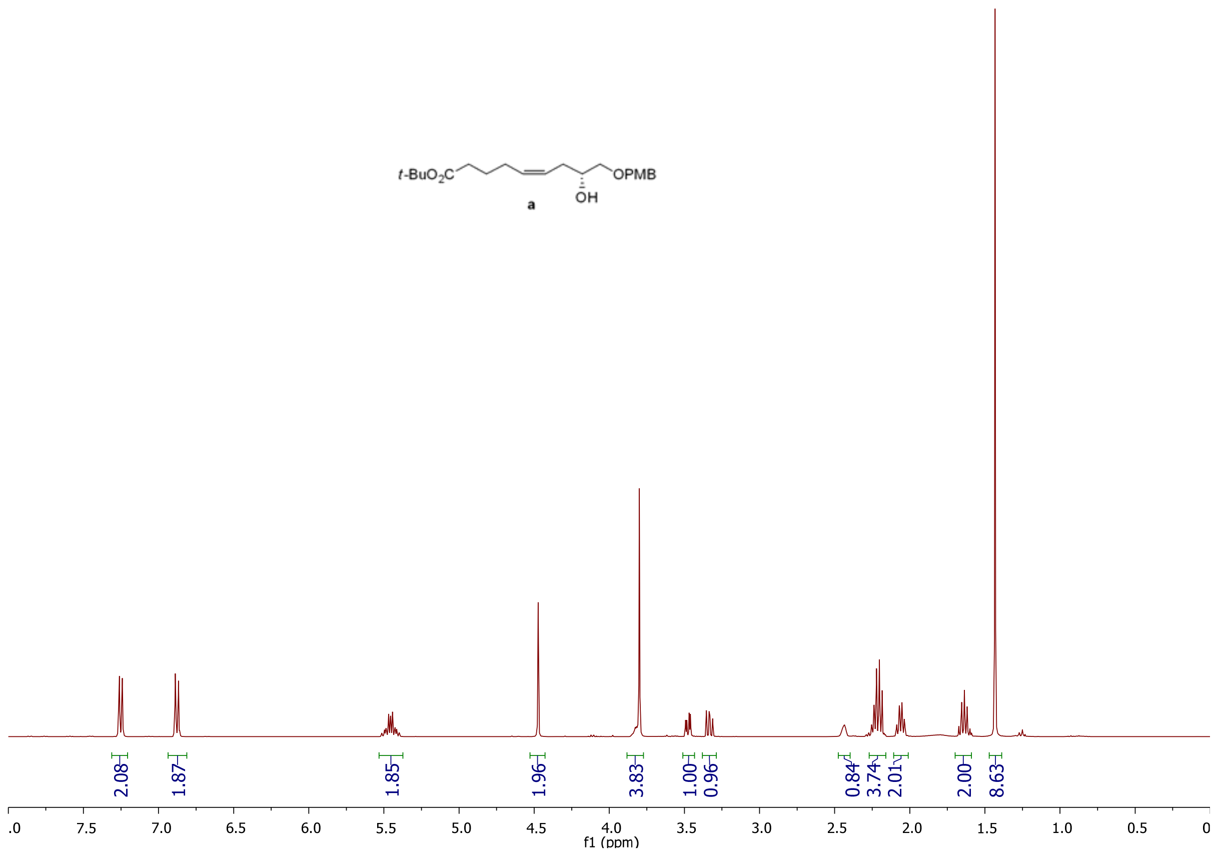
t-BuO2C
OPMB
OH
a
2.08
1.87
1.85
1.96
3.83
1.00
0.96
0.84
3.74
2.01
2.00
8.63
f1 (ppm)

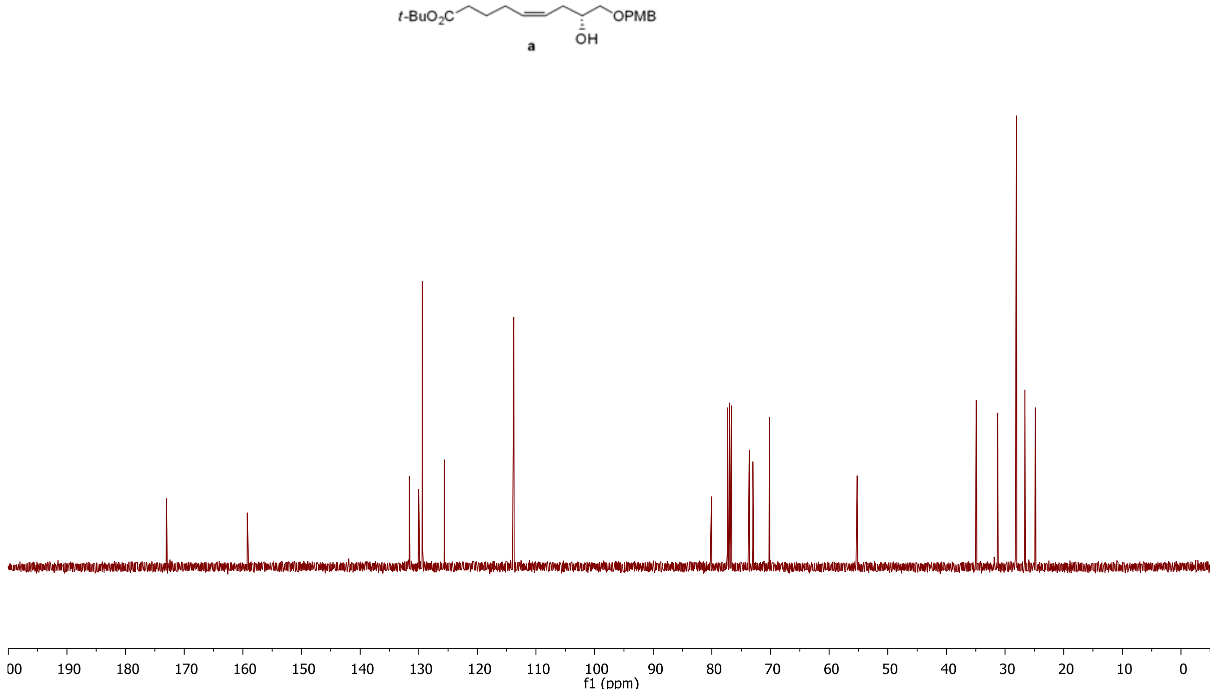
t-BuO2C
OPMB
OH
a
f1 (ppm)

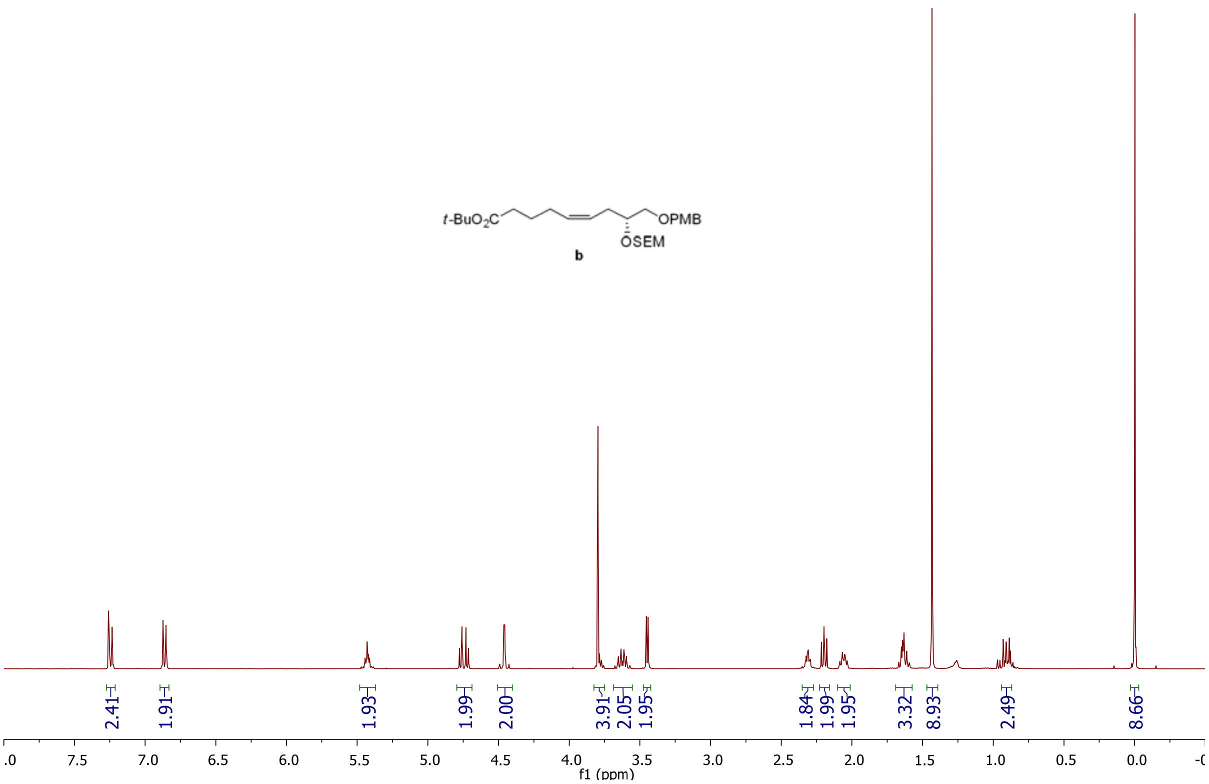
t-BuO2C
OPMB
OSEM
b
2.41
1.91
1.93
1.99
2.00
3.91
2.05
1.95
1.84
1.99
1.95
3.32
8.93
2.49
8.66
f1 (ppm)

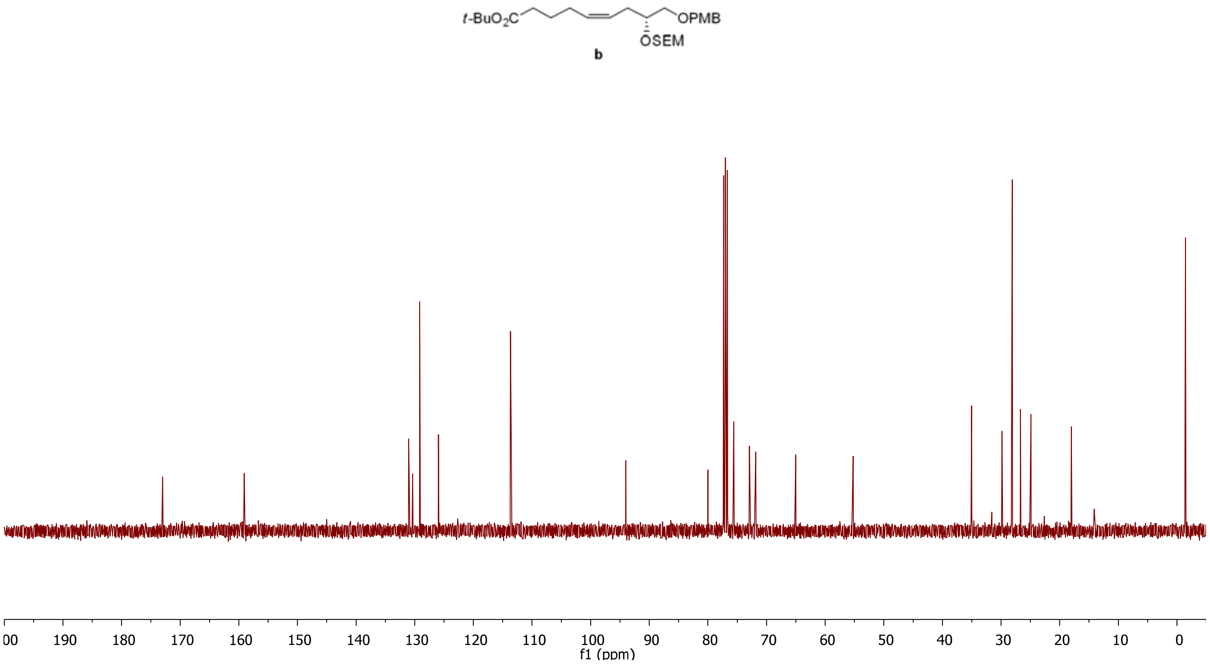
t-BuO2C
OPMB
OSEM
b
f1 (ppm)

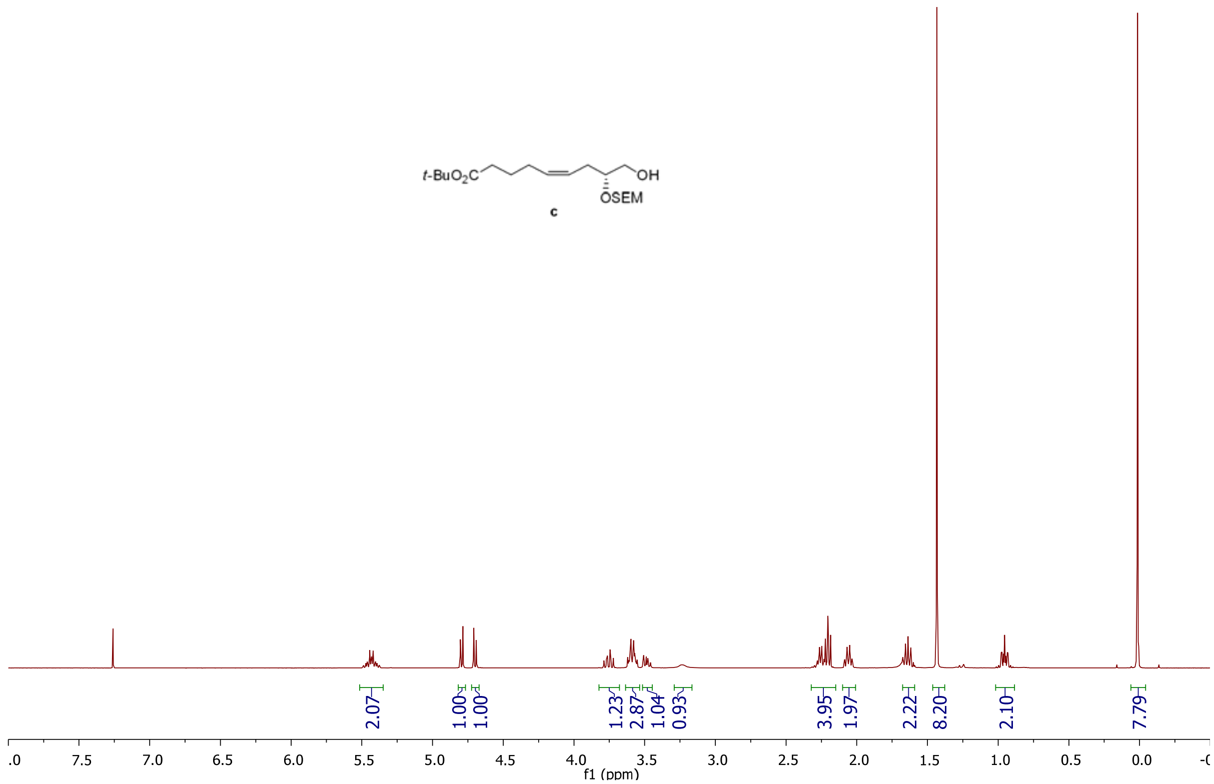
t-BuO$_2$C
OH
OSEM
c
2.07
1.00
1.00
1.23
2.87
1.04
0.93
3.95
1.97
2.22
8.20
2.10
7.79
7.5 7.0 6.5 6.0 5.5 5.0 4.5 4.0 3.5 3.0 2.5 2.0 1.5 1.0 0.5 0.0
f1 (ppm)

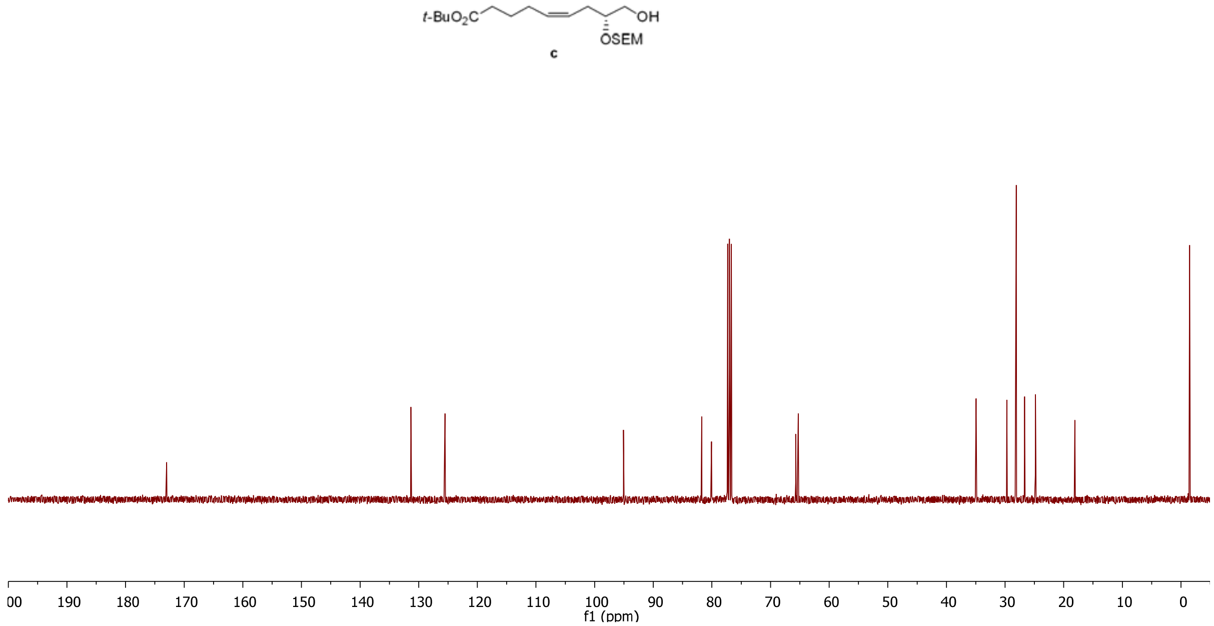
t-BuO$_2$C
OH
OSEM
c
190 180 170 160 150 140 130 120 110 100 90 80 70 60 50 40 30 20 10 0
f1 (ppm)

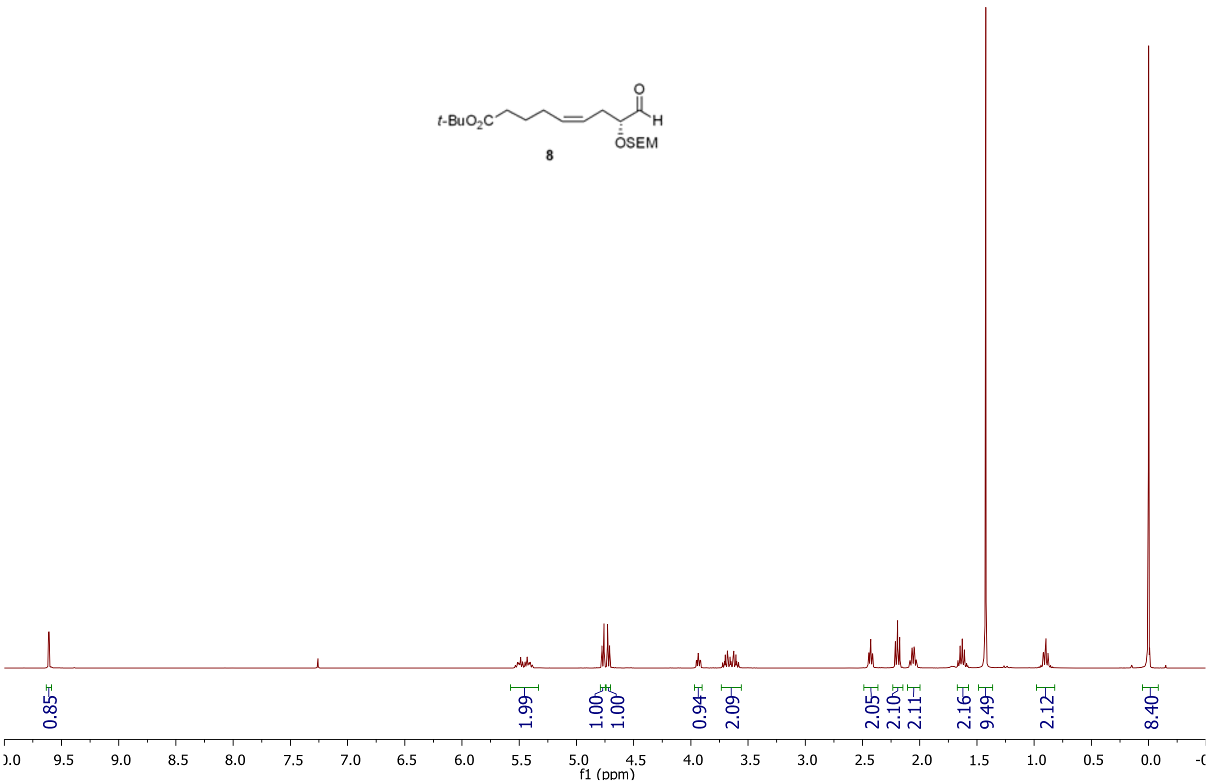
t-BuO2C
H
OSEM
8
0.85
1.99
1.00
1.00
0.94
2.09
2.05
2.10
2.11
2.16
9.49
2.12
8.40
9.5 9.0 8.5 8.0 7.5 7.0 6.5 6.0 5.5 5.0 4.5 4.0 3.5 3.0 2.5 2.0 1.5 1.0 0.5 0.0
f1 (ppm)

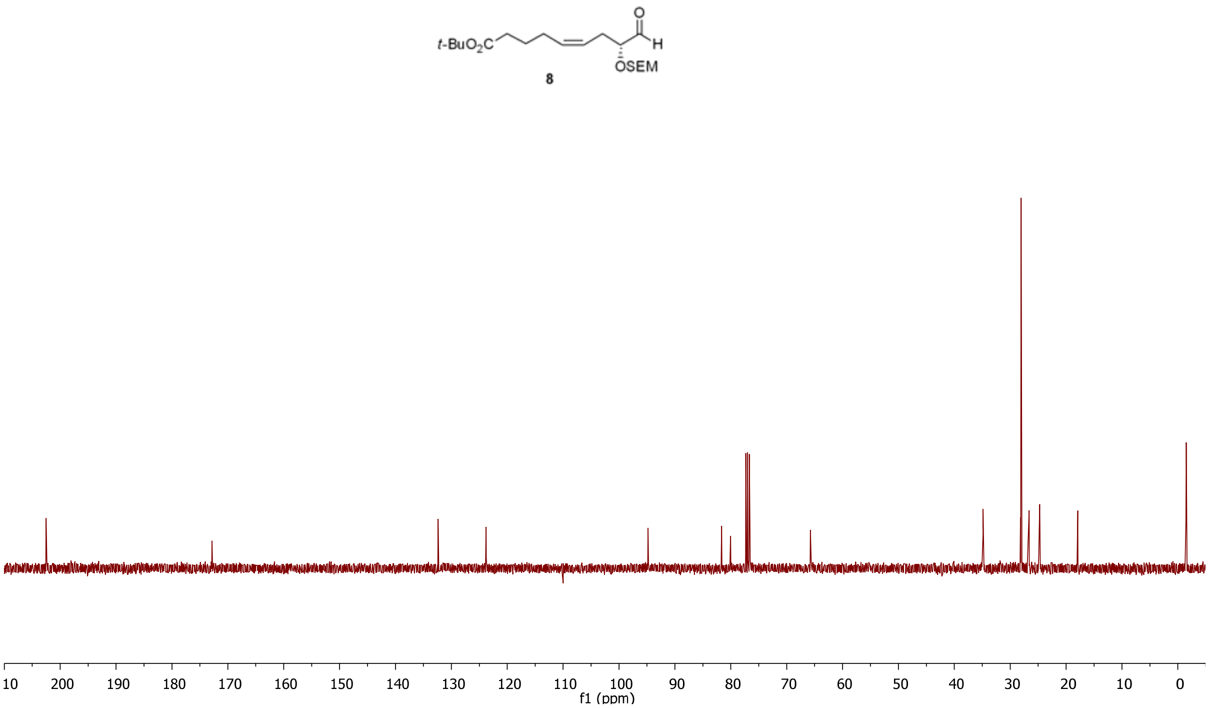
t-BuO2C
H
OSEM
8
200 190 180 170 160 150 140 130 120 110 100 90 80 70 60 50 40 30 20 10 0
f1 (ppm)

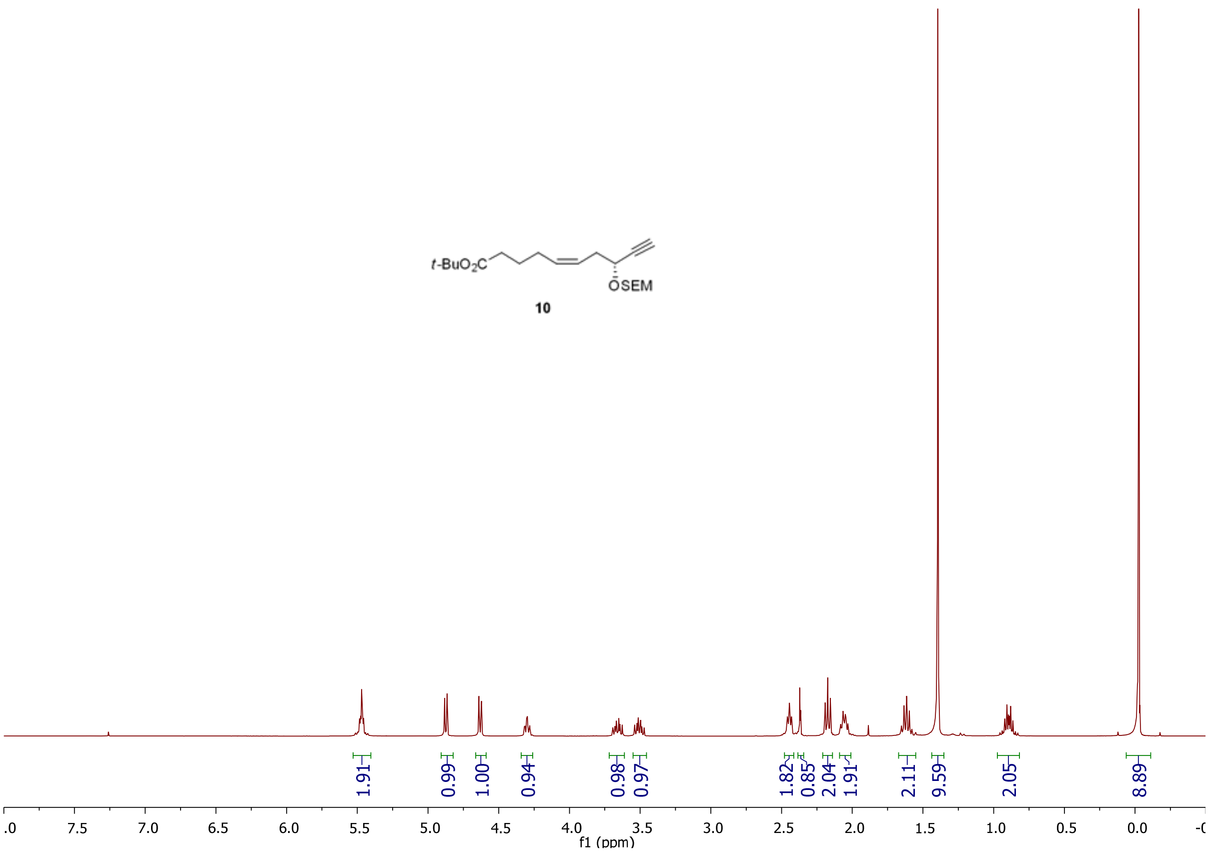
t-BuO2C
OSEM
10
1.91
0.99
1.00
0.94
0.98
0.97
1.82
0.85
2.04
1.91
2.11
9.59
2.05
8.89
f1 (ppm)

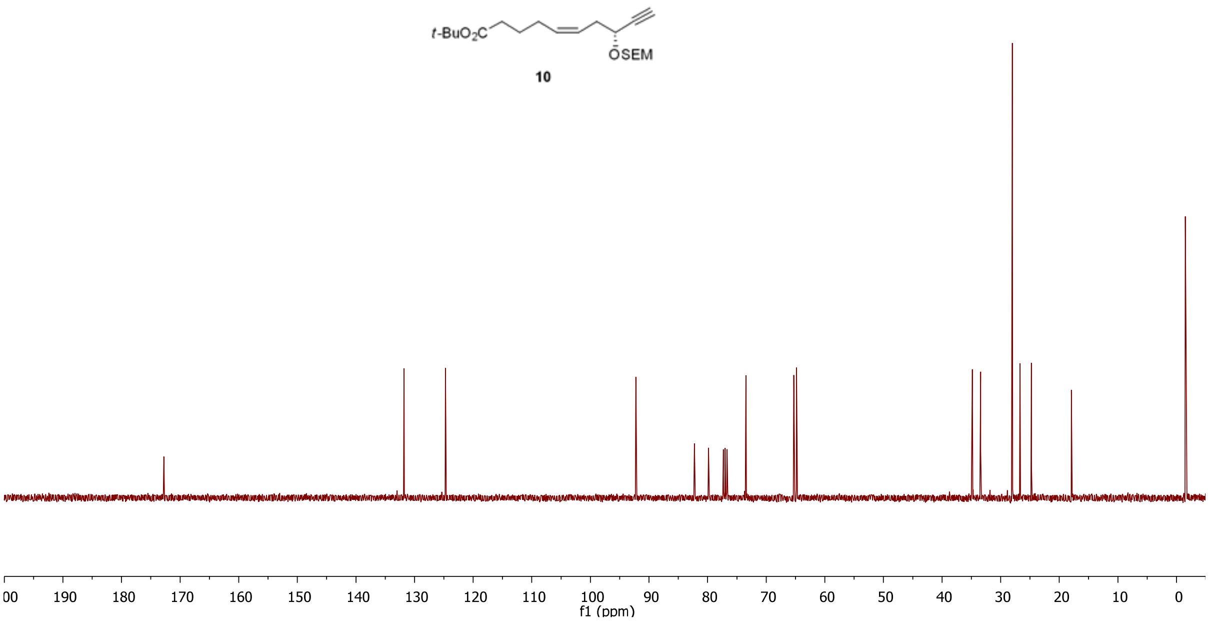
t-BuO2C
OSEM
10
f1 (ppm)

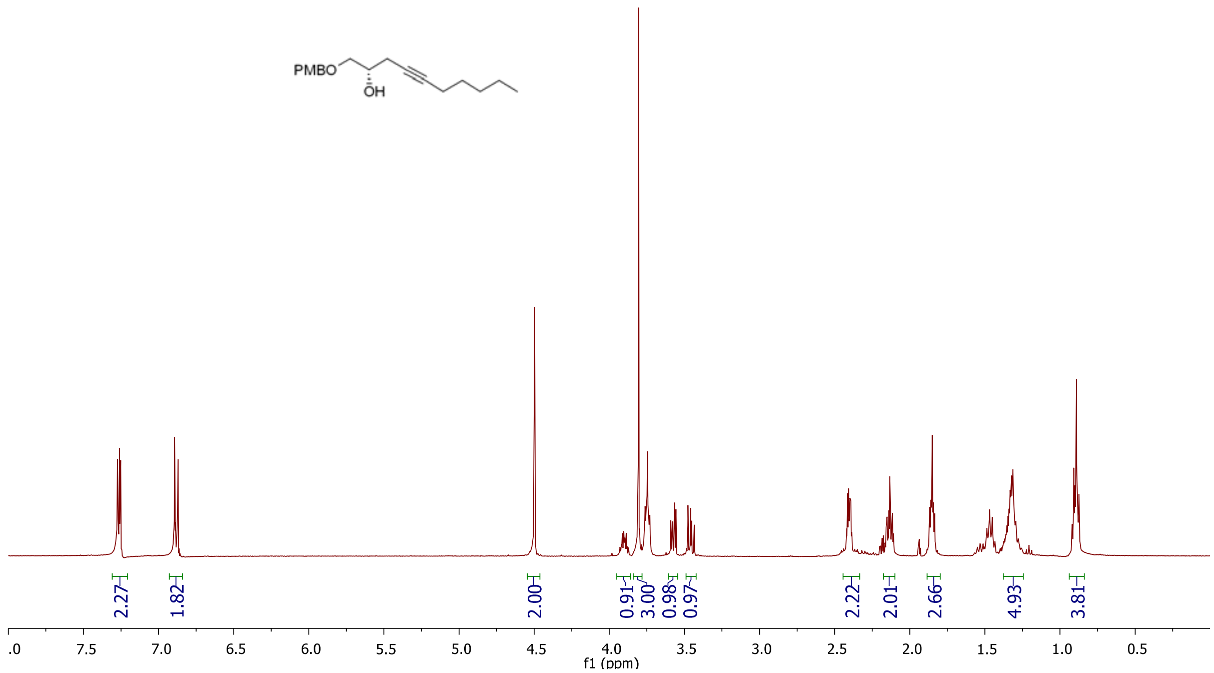
PMBO
OH
2.27
1.82
2.00
0.91
3.00
0.98
0.97
2.22
2.01
2.66
4.93
3.81
f1 (ppm)

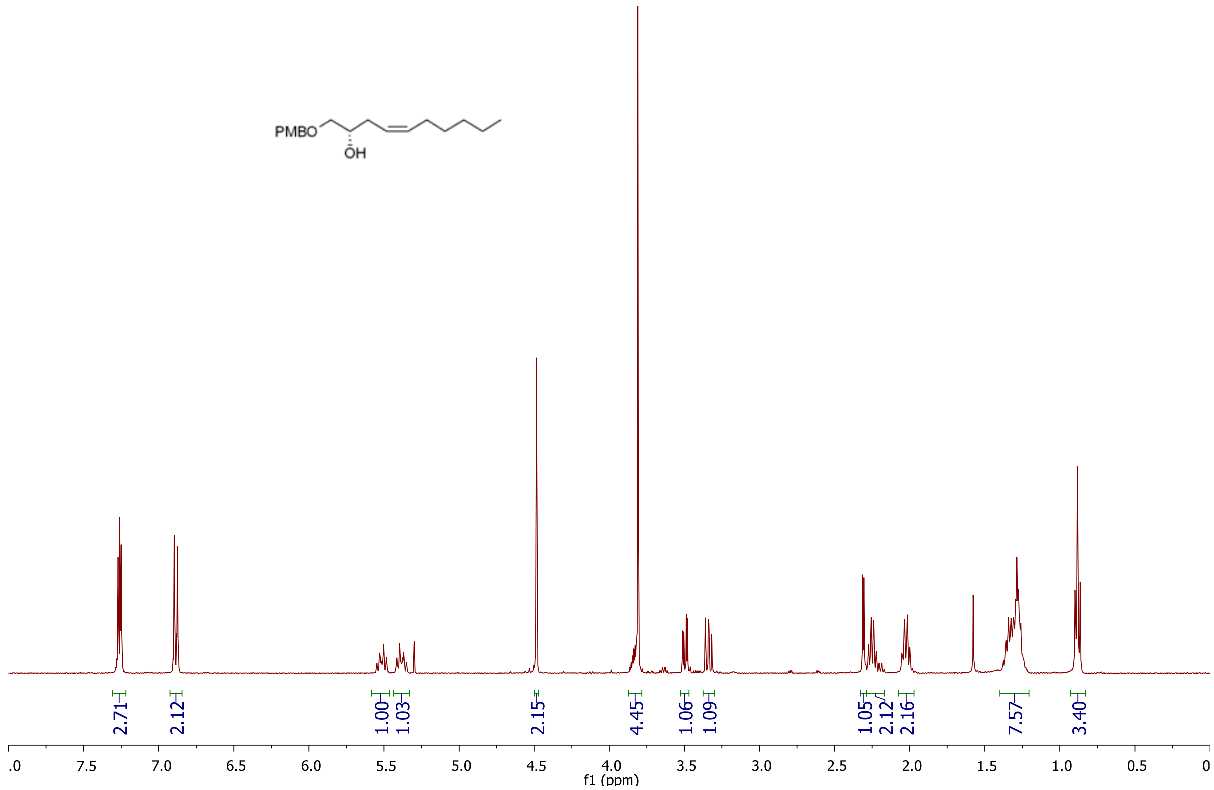
PMBO
OH
2.71
2.12
1.00
1.03
2.15
4.45
1.06
1.09
1.05
2.12
2.16
7.57
3.40
f1 (ppm)

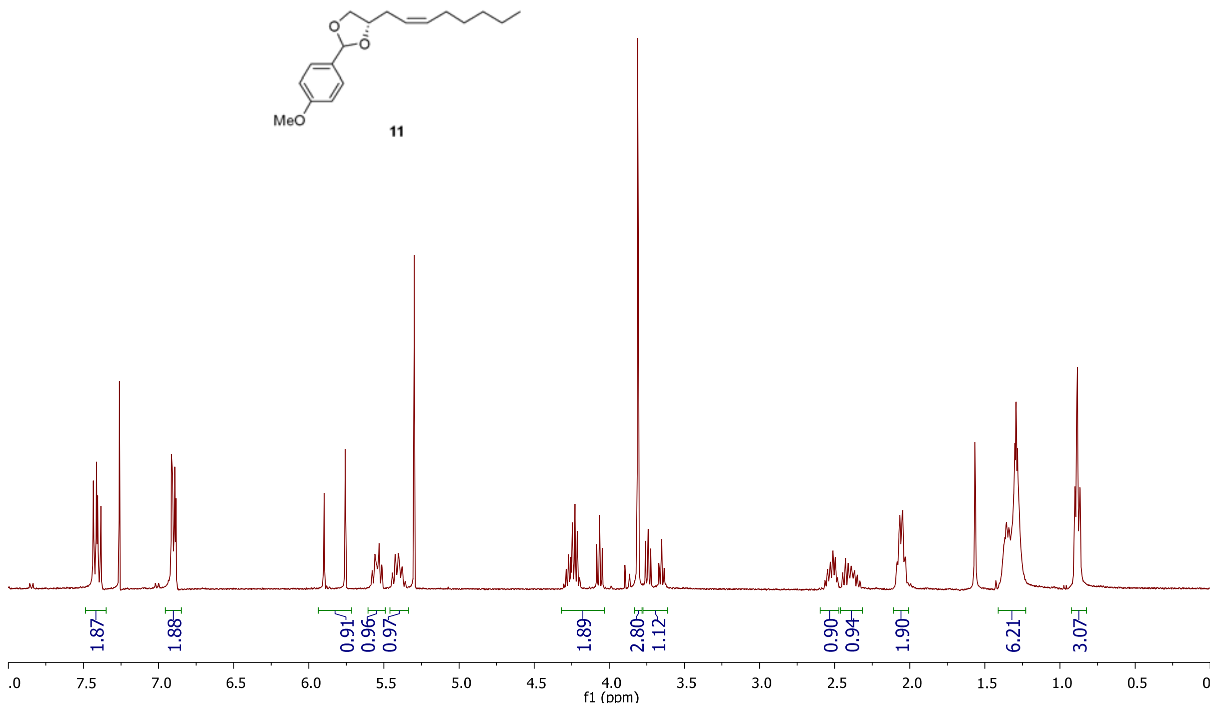
MeO
11
1.87
1.88
0.91
0.96
0.97
1.89
2.80
1.12
0.90
0.94
1.90
6.21
3.07
f1 (ppm)

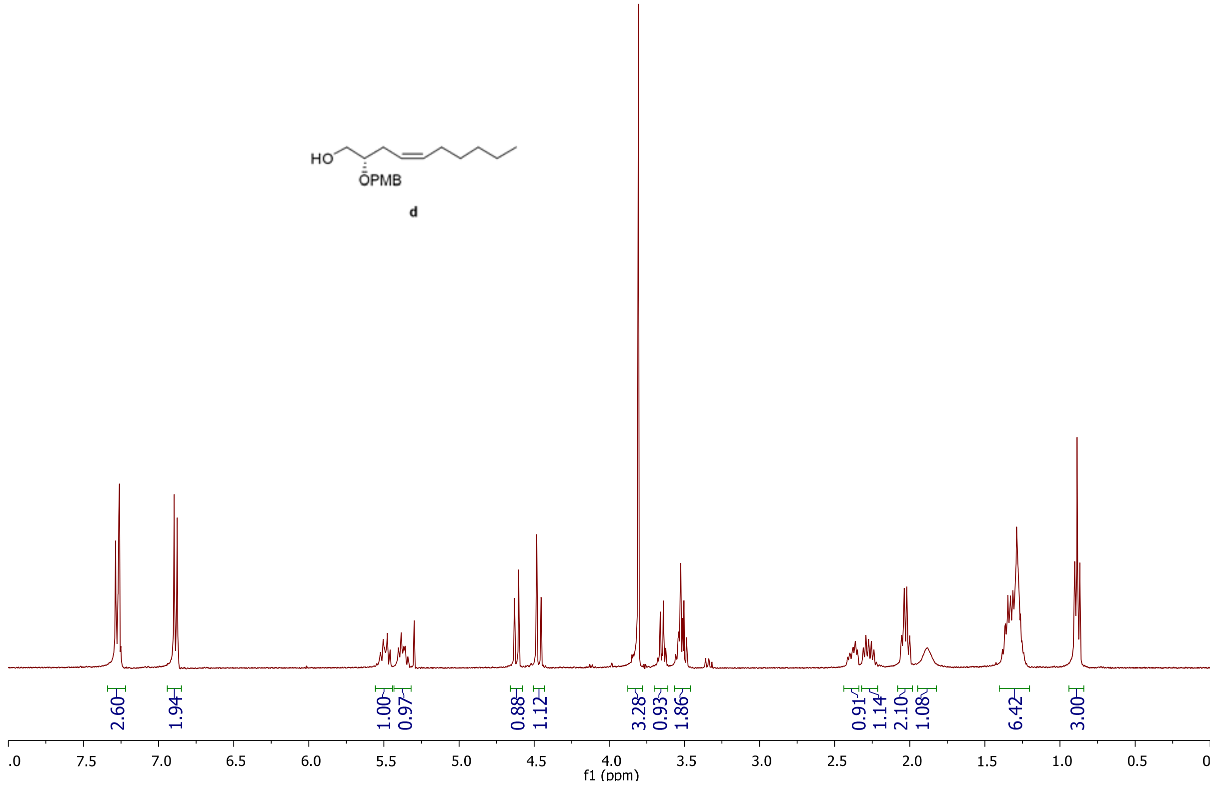
HO
OPMB
d
2.60
1.94
1.00
0.97
0.88
1.12
3.28
0.93
1.86
0.91
1.14
2.10
1.08
6.42
3.00
f1 (ppm)

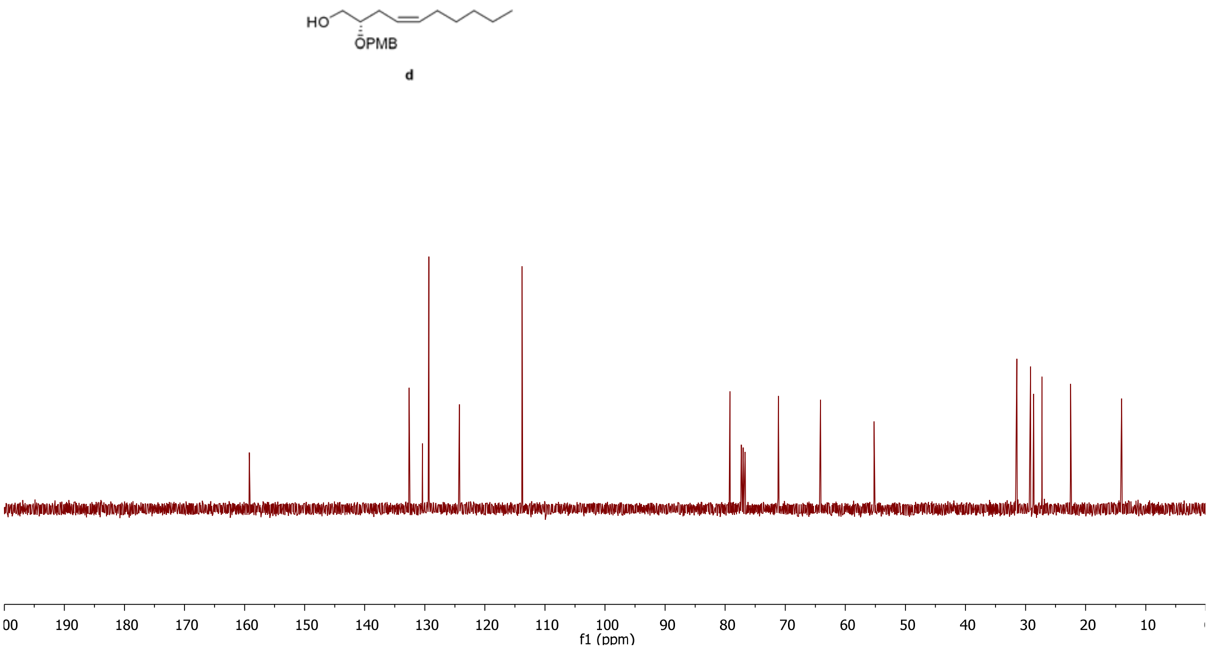
HO
OPMB
d
f1 (ppm)

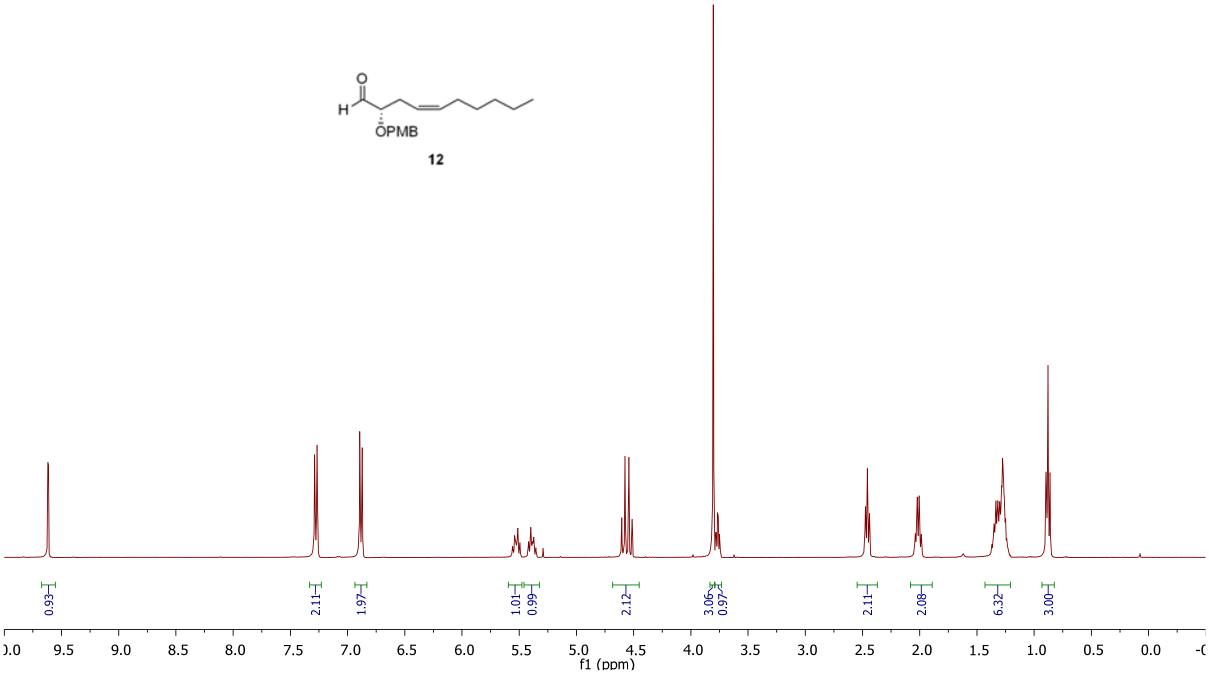
H
O
OPMB
12
0.93
2.11
1.97
1.01
0.99
2.12
3.06
0.97
2.11
2.08
6.32
3.00
f1 (ppm)

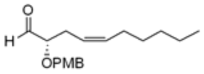
O
H
OPMB

12

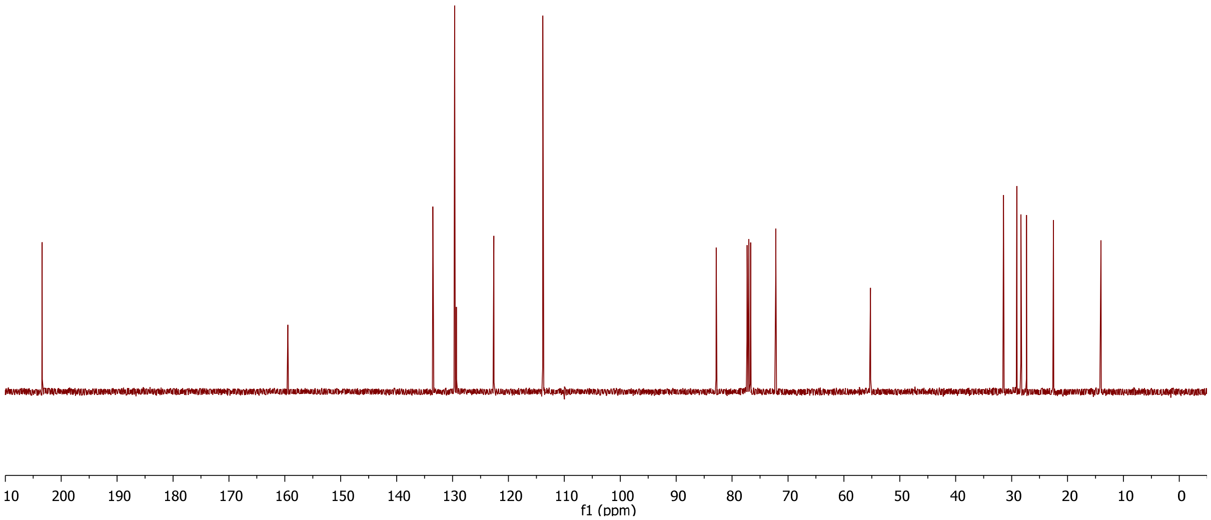
10 200 190 180 170 160 150 140 130 120 110 100 90 80 70 60 50 40 30 20 10 0
f1 (ppm)

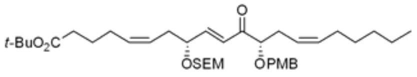
t-BuO2C
O
OSEM
OPMB

e

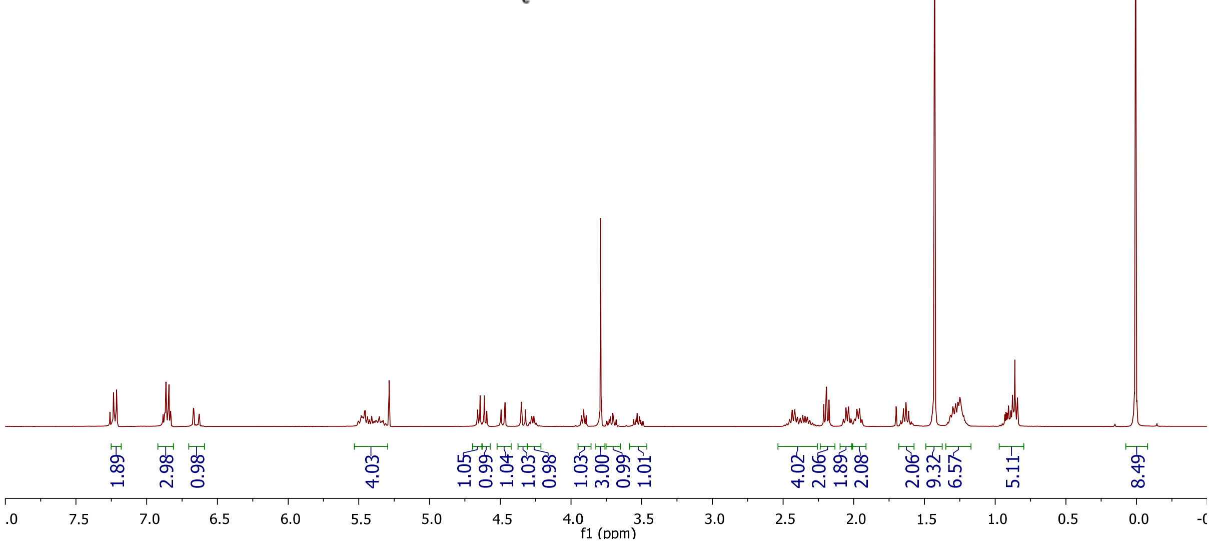
1.89
2.98
0.98
4.03
1.05
0.99
1.04
1.03
0.98
1.03
3.00
0.99
1.01
4.02
2.06
1.89
2.08
2.06
9.32
6.57
5.11
8.49
7.5 7.0 6.5 6.0 5.5 5.0 4.5 4.0 3.5 3.0 2.5 2.0 1.5 1.0 0.5 0.0
f1 (ppm)

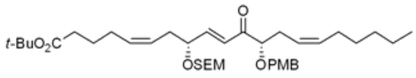
t-BuO2C
OSEM
OPMB
O

e

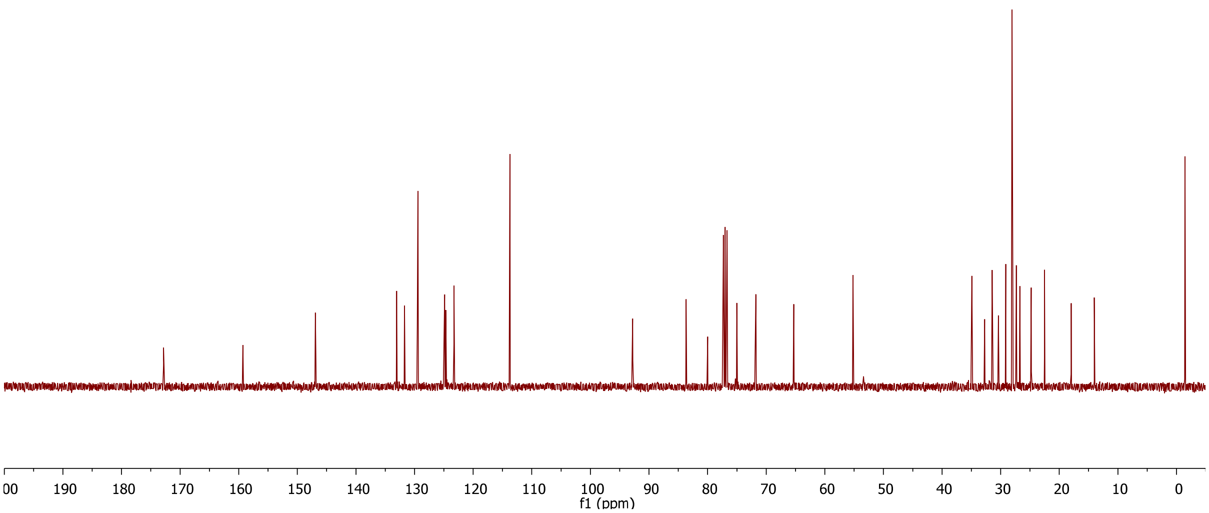
f1 (ppm)

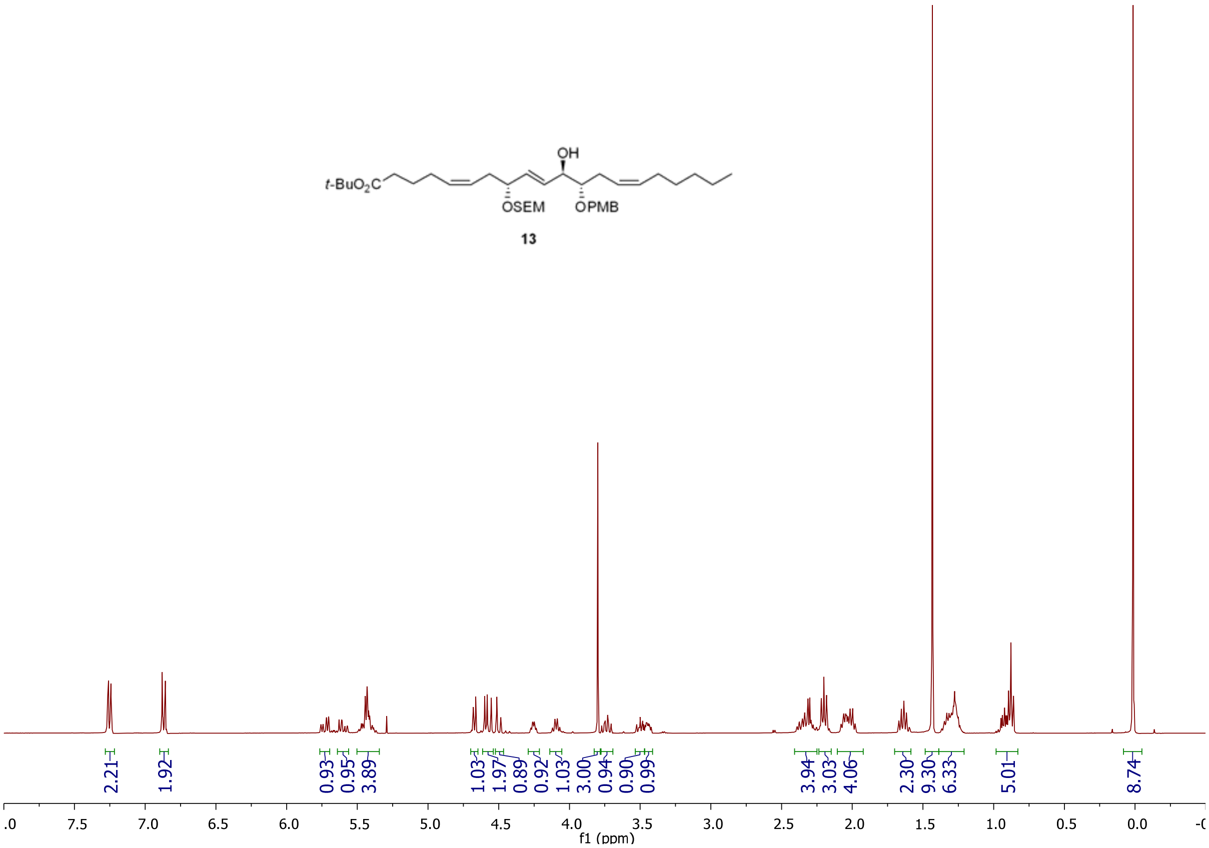
t-BuO2C
OSEM
OPMB
OH
13
2.21
1.92
0.93
0.95
3.89
1.03
1.97
0.89
0.92
1.03
3.00
0.94
0.90
0.99
3.94
3.03
4.06
2.30
9.30
6.33
5.01
8.74
f1 (ppm)

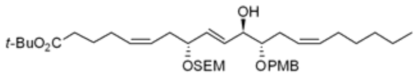
OH
t-BuO2C
OSEM
OPMB

13

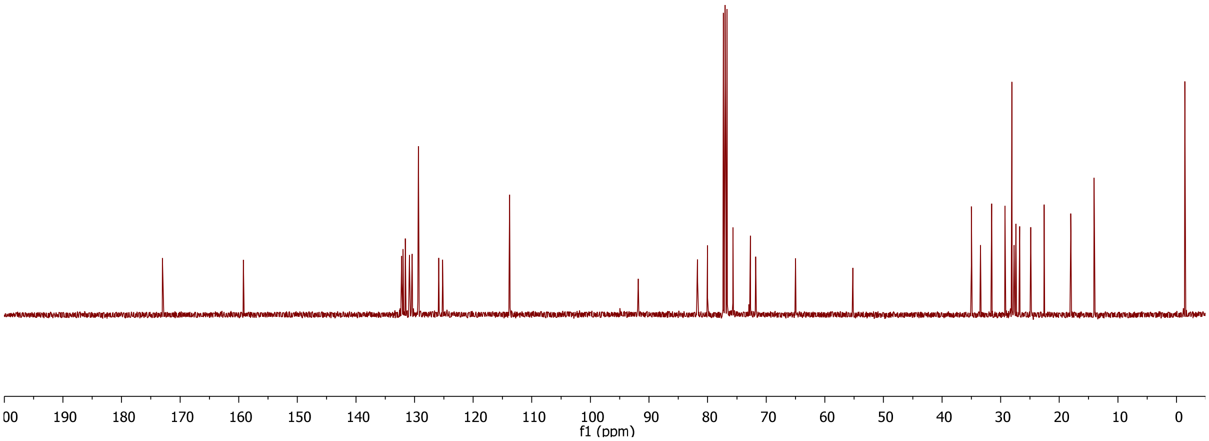
00
190
180
170
160
150
140
130
120
110
100
90
80
70
60
50
40
30
20
10
0
f1 (ppm)

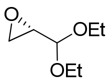

(*S*)-**A**

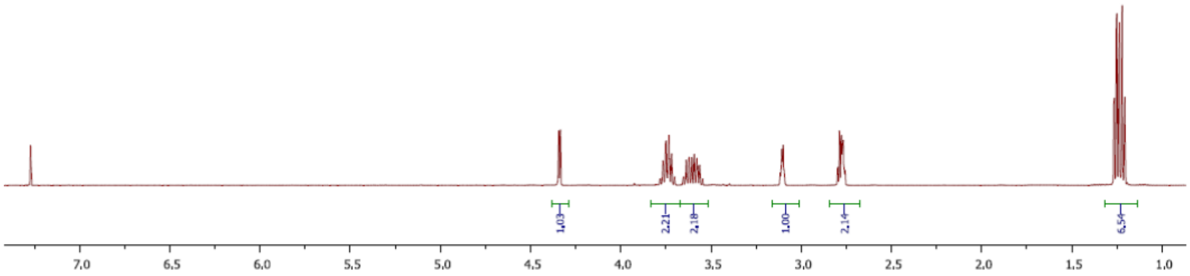

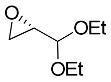

(*S*)-**A**

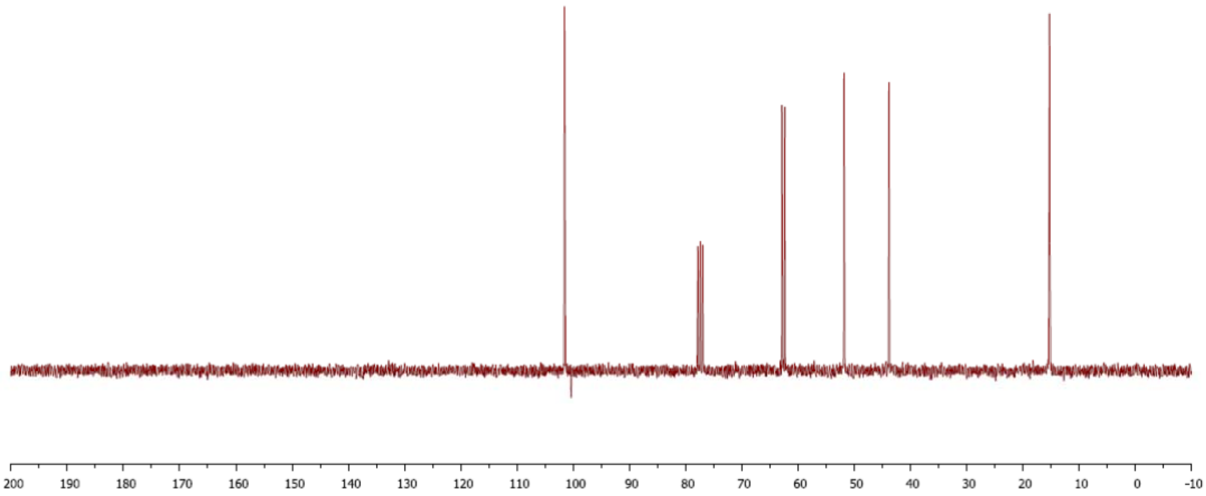

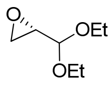

(*S*)-**A**

Racemate

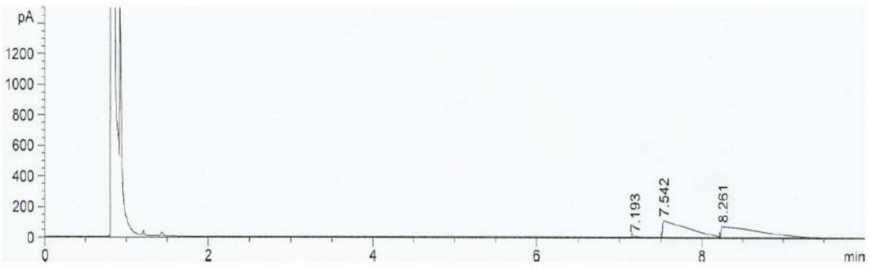

Peak #	RetTime [min]	Type	Width [min]	Area [pA*s]	Height [pA]	Area %
1	7.193	BB	0.0648	19.99274	4.47801	0.43338
2	7.542	BV	0.2568	2281.93335	107.70905	49.46466
3	8.261	VB	0.4064	2311.33398	68.69936	50.10197

GC Conditions: Lipodex E (Column V, Macherey-Nagel), 60°C, 19.4 min

Scalemic Compound

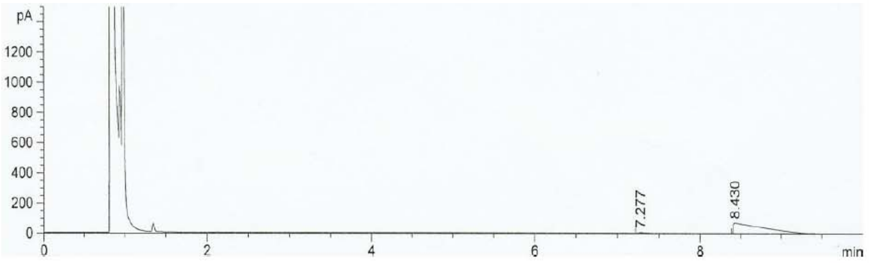

Peak #	RetTime [min]	Type	Width [min]	Area [pA*s]	Height [pA]	Area %
1	7.277	BB	0.0734	23.80262	4.59796	1.33889
2	8.430	BB	0.3102	1753.99121	68.50214	98.66111

GC chromatogram for compound (*S*)-**A**

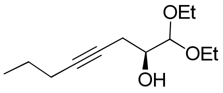
OEt
OEt
OH

B

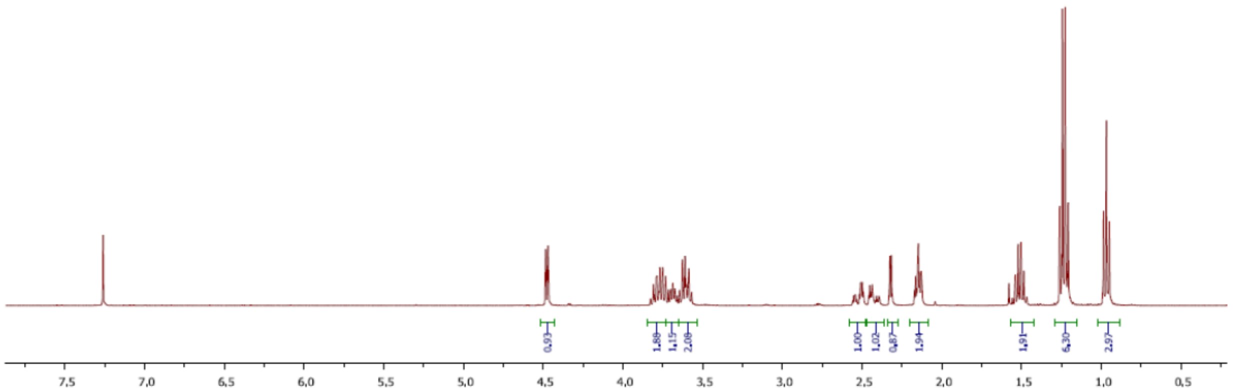
0,93
1,88
1,15
2,08
1,00
1,02
0,87
1,94
1,91
6,30
2,97
7,5
7,0
6,5
6,0
5,5
5,0
4,5
4,0
3,5
3,0
2,5
2,0
1,5
1,0
0,5

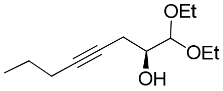
OEt
OEt
OH

B

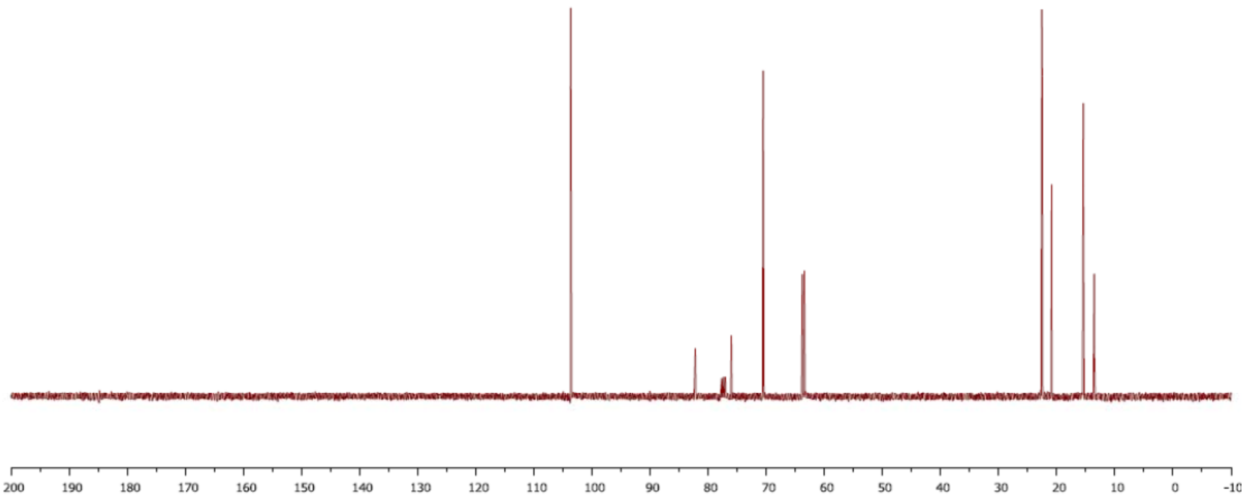
200
190
180
170
160
150
140
130
120
110
100
90
80
70
60
50
40
30
20
10
0

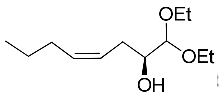

OEt
OEt
OH

C

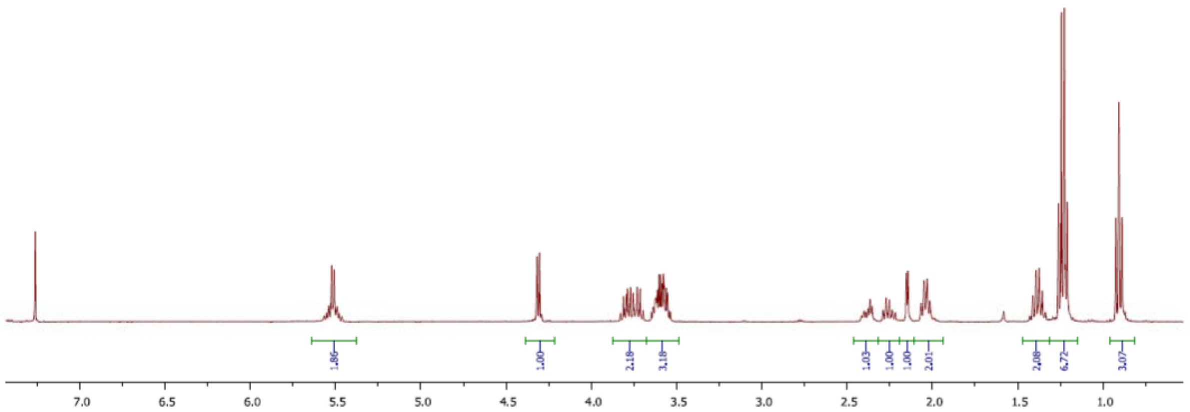

1.86
1.00
2.18
3.18
1.03
1.00
1.00
2.01
2.08
6.72
3.07
7.0
6.5
6.0
5.5
5.0
4.5
4.0
3.5
3.0
2.5
2.0
1.5
1.0

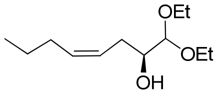

OEt
OEt
OH

C

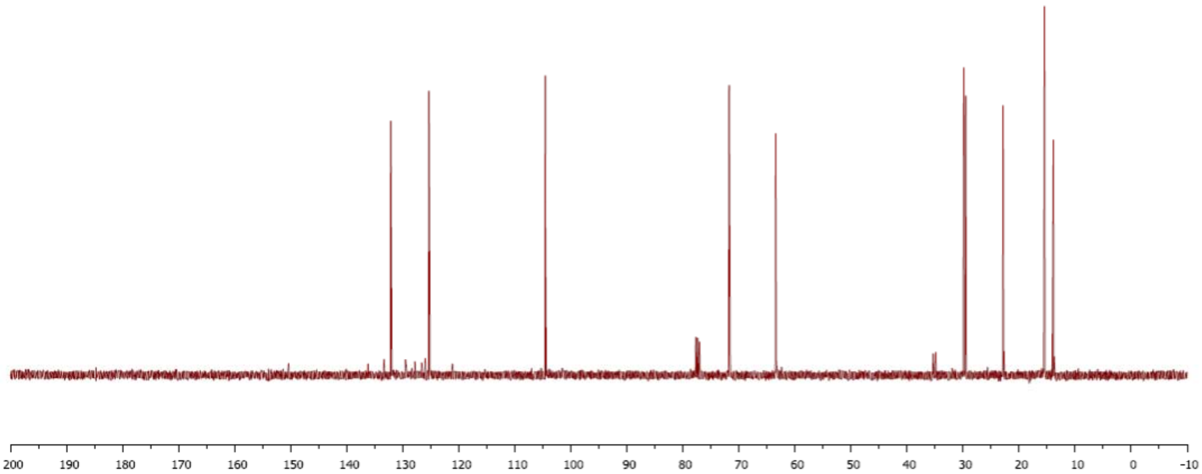

200
190
180
170
160
150
140
130
120
110
100
90
80
70
60
50
40
30
20
10
0
-10

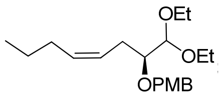
OEt
OEt
OPMB

D

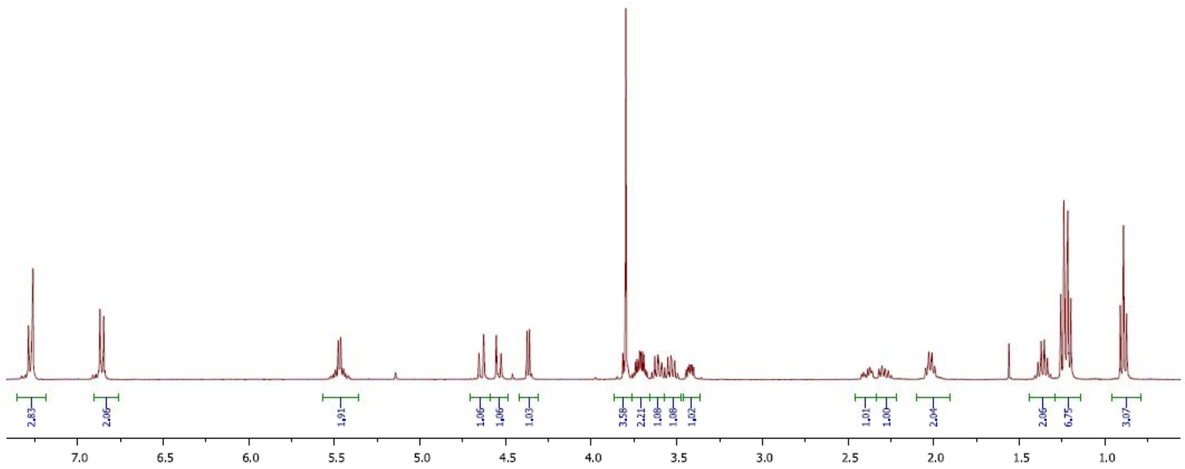
2.83
2.06
1.91
1.06
1.06
1.03
3.58
2.21
1.08
1.08
1.02
1.01
1.00
2.04
2.06
6.75
3.07
7.0
6.5
6.0
5.5
5.0
4.5
4.0
3.5
3.0
2.5
2.0
1.5
1.0

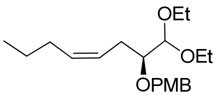
OEt
OEt
OPMB

D

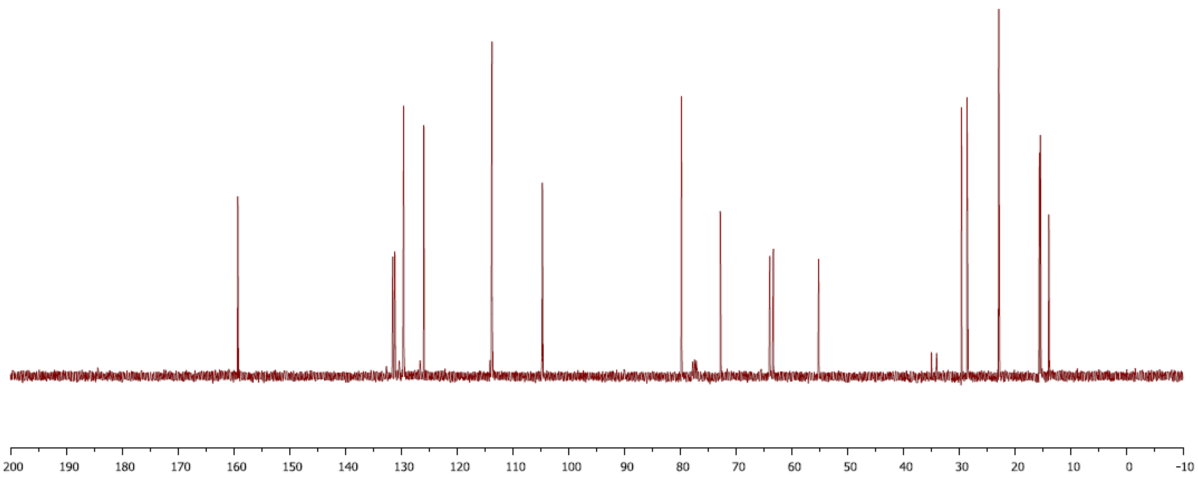
200
190
180
170
160
150
140
130
120
110
100
90
80
70
60
50
40
30
20
10
0
-10

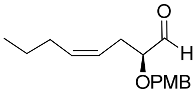

E

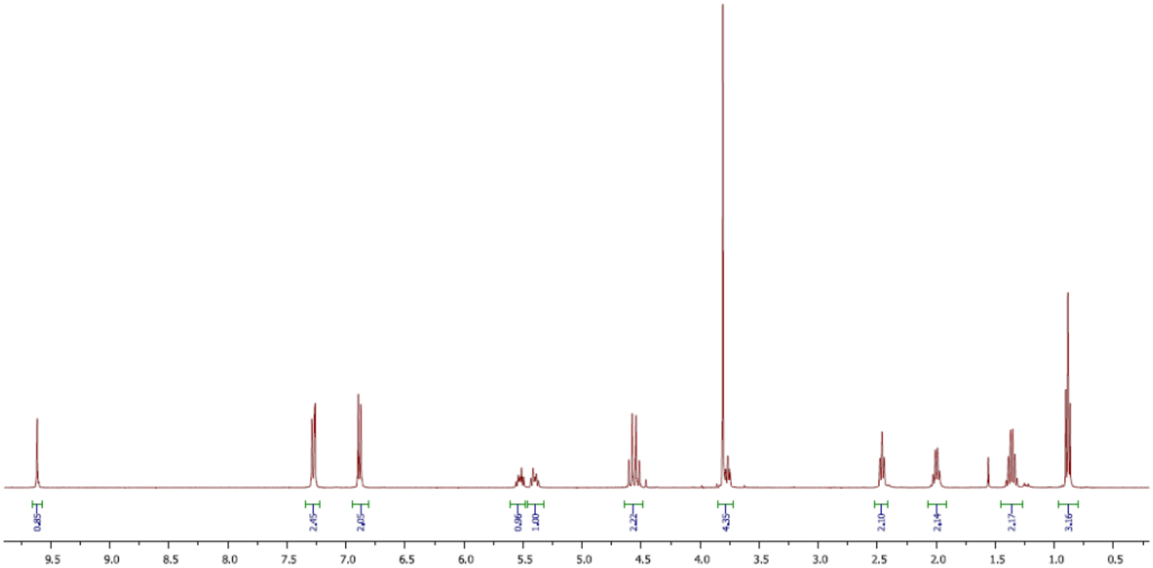

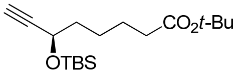
CO2t-Bu
OTBS

F

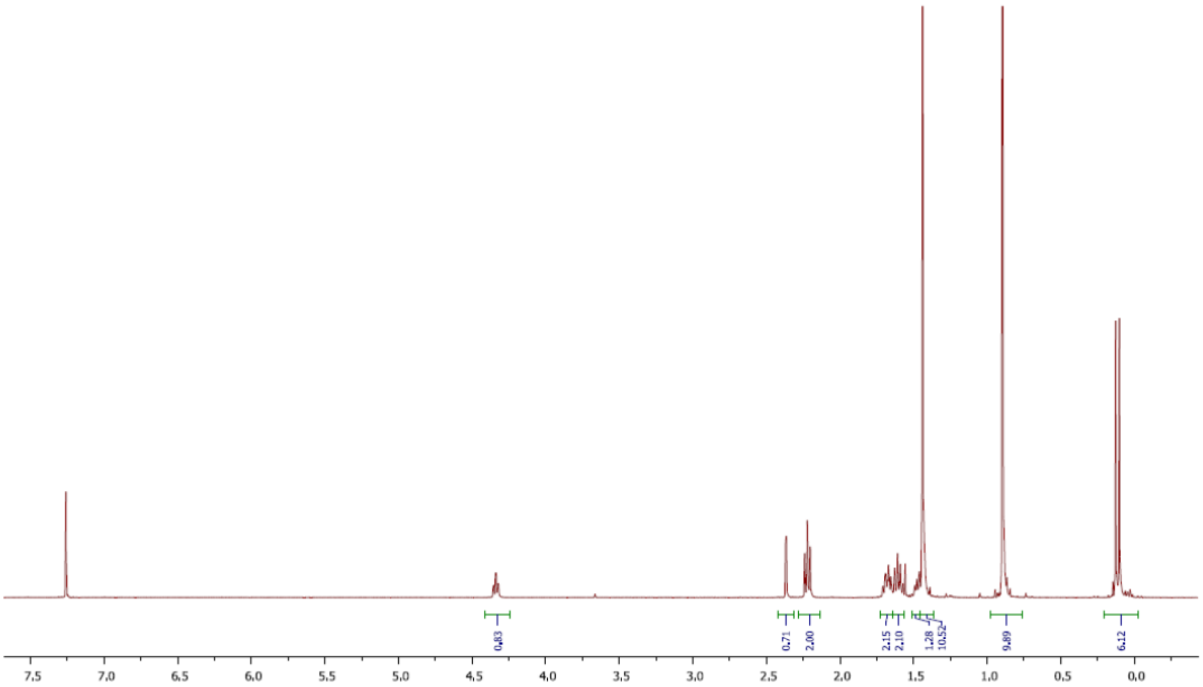
0.83
0.71
2.00
2.15
2.10
1.28
10.52
9.89
6.12
7.5 7.0 6.5 6.0 5.5 5.0 4.5 4.0 3.5 3.0 2.5 2.0 1.5 1.0 0.5 0.0

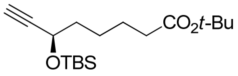
CO2t-Bu
OTBS

F

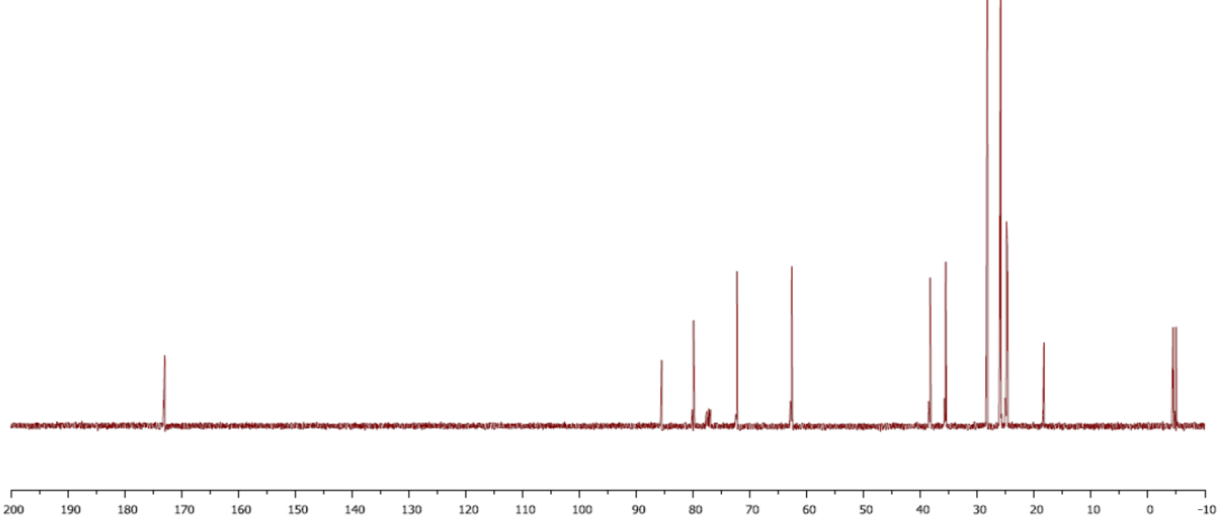
200 190 180 170 160 150 140 130 120 110 100 90 80 70 60 50 40 30 20 10 0 -10

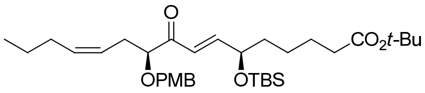

G

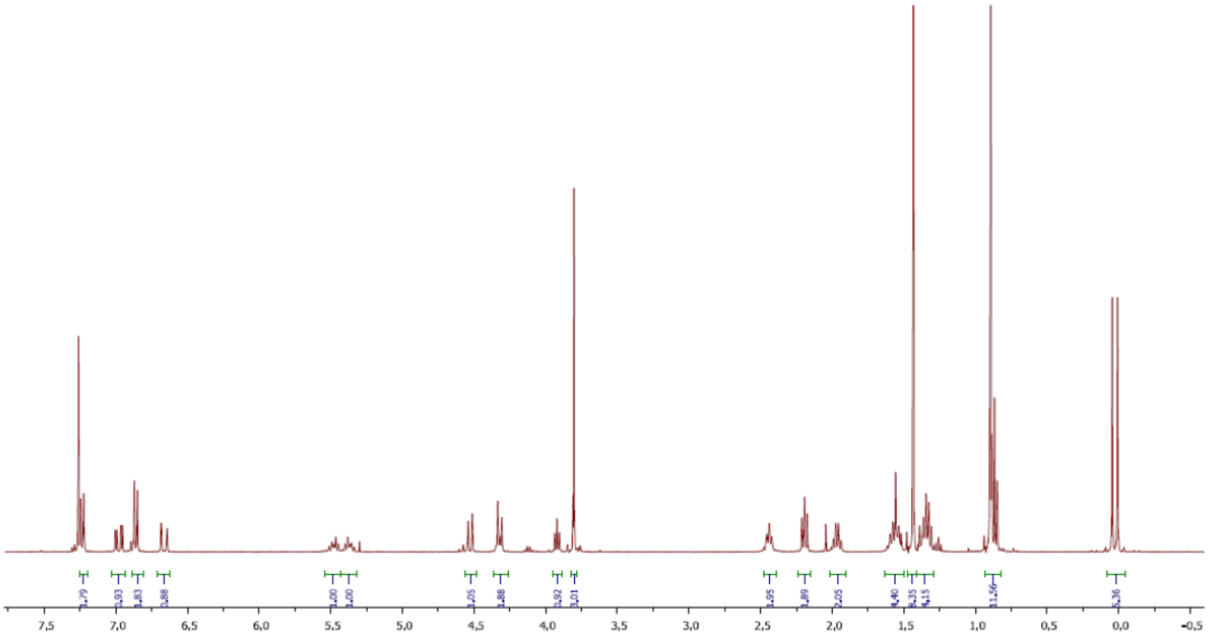

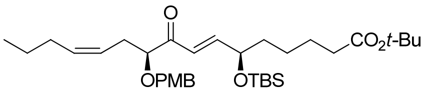

G

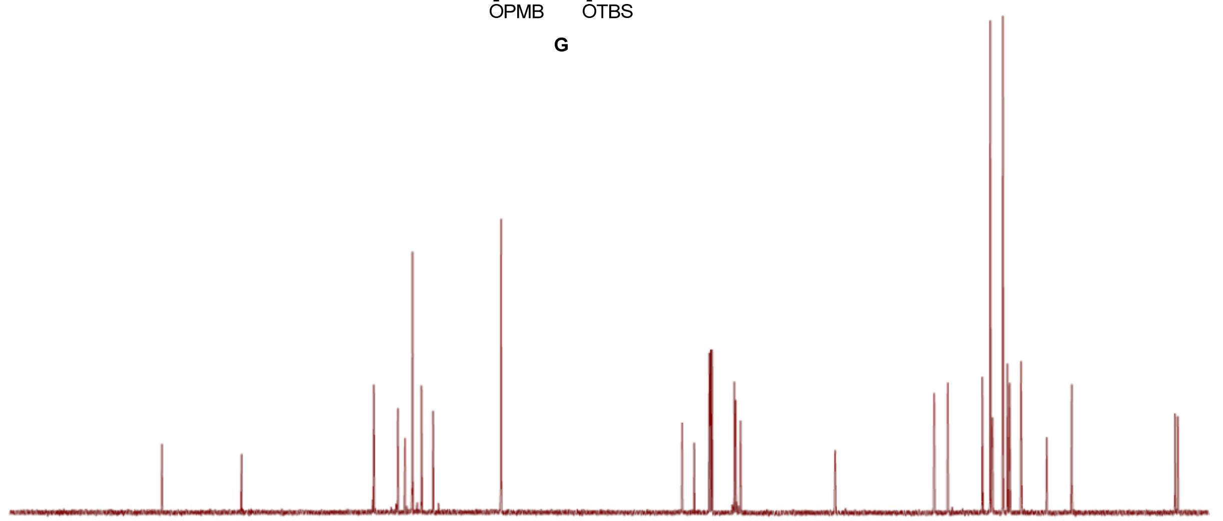

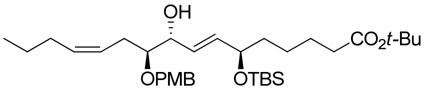
OH
CO_2t-Bu
OPMB
OTBS

H

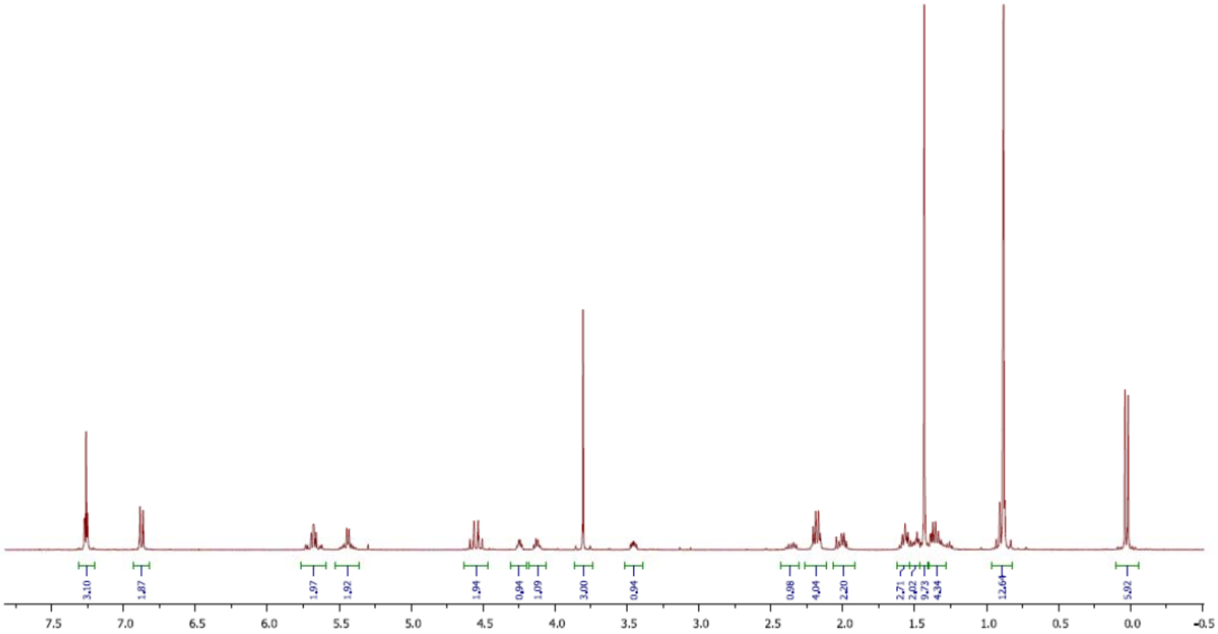
3.10
1.87
1.97
1.92
1.94
0.94
1.09
3.00
0.94
0.98
4.04
2.20
2.71
2.02
9.73
4.34
12.64
5.92
7.5 7.0 6.5 6.0 5.5 5.0 4.5 4.0 3.5 3.0 2.5 2.0 1.5 1.0 0.5 0.0 -0.5

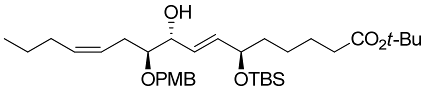
OH
CO_2t-Bu
OPMB
OTBS

H

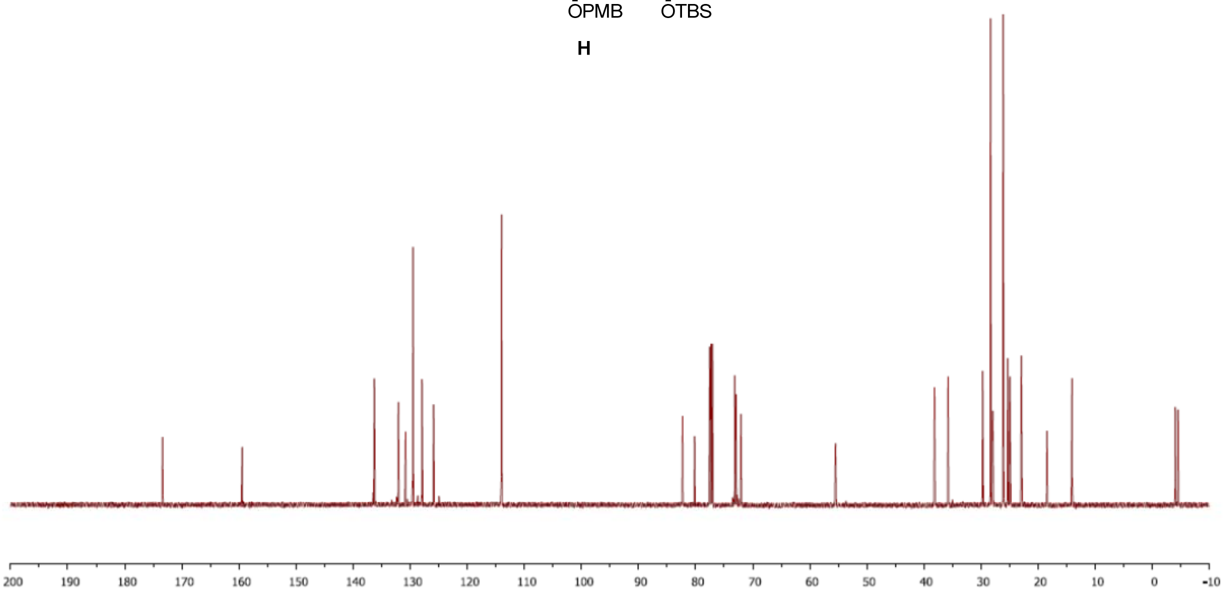
200 190 180 170 160 150 140 130 120 110 100 90 80 70 60 50 40 30 20 10 0 -10

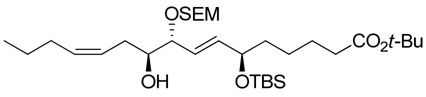

14

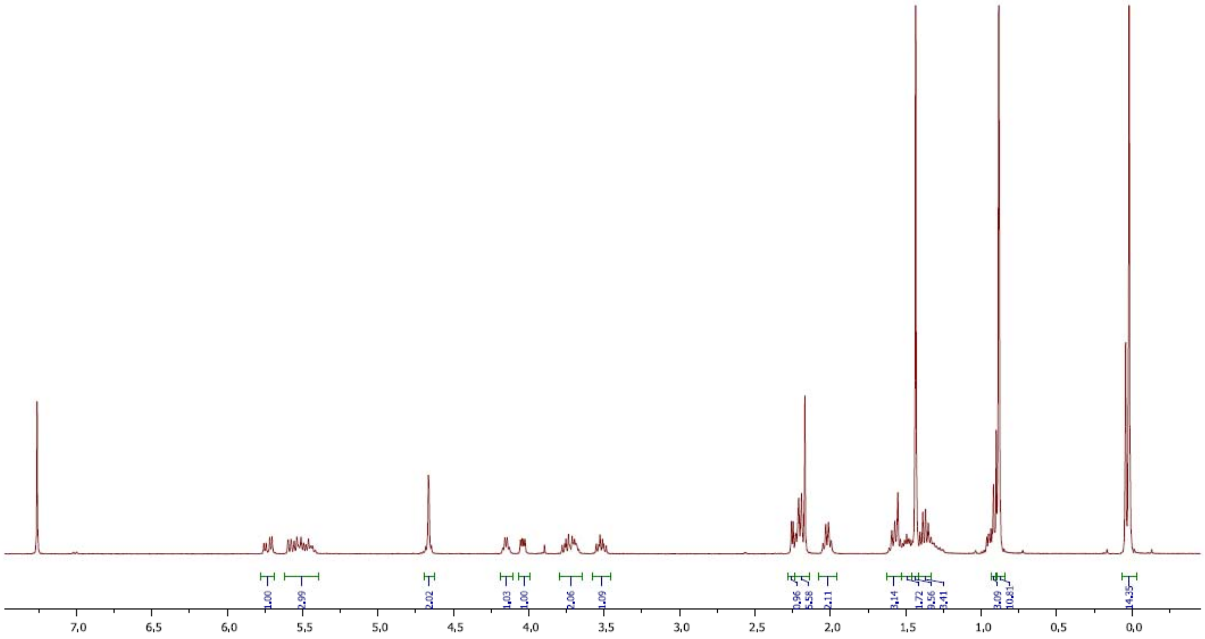

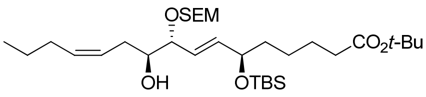

14

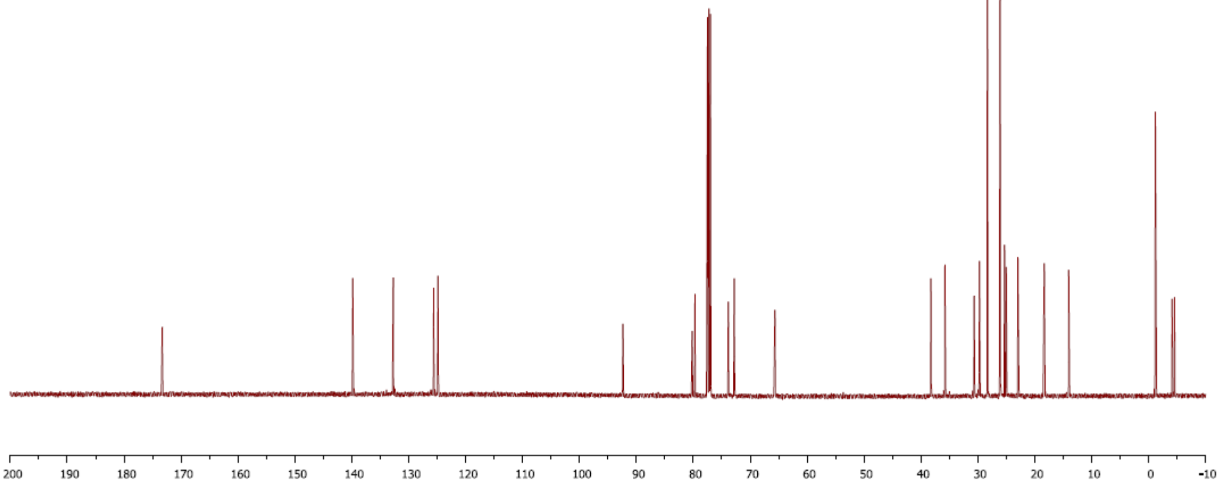

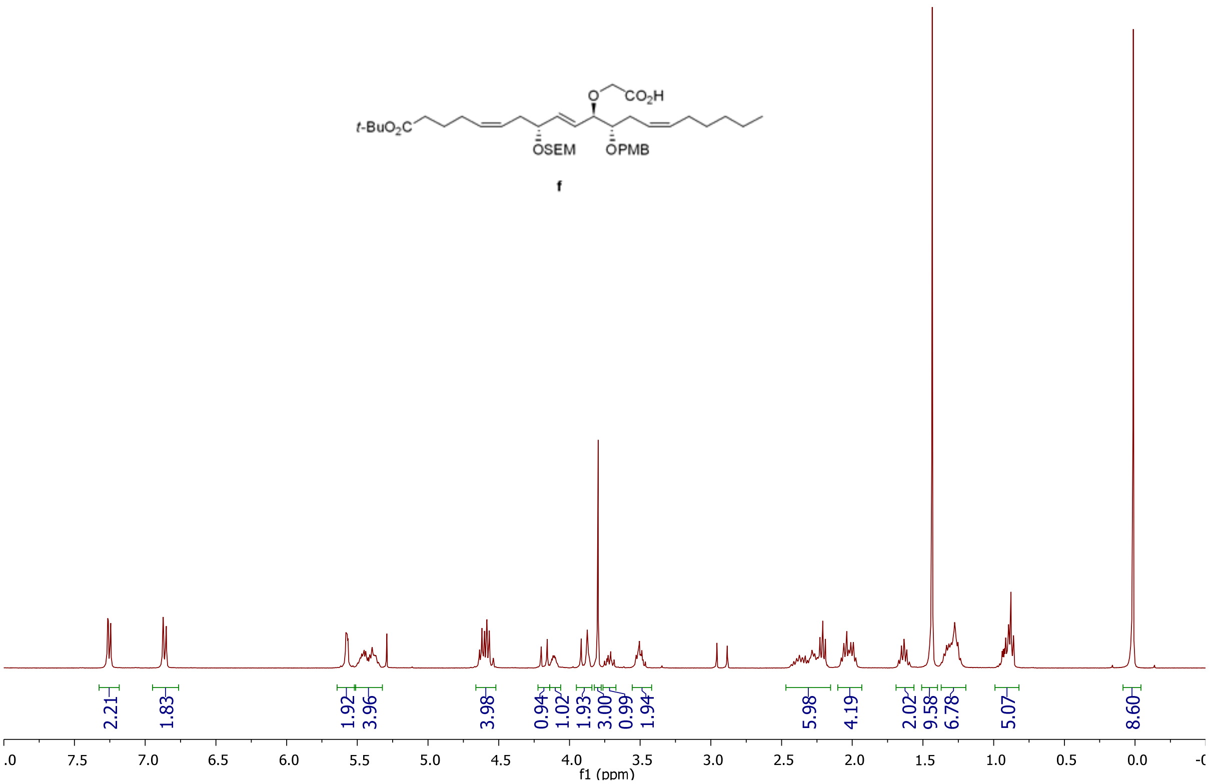
t-BuO2C
CO2H
OSEM
OPMB
f
2.21
1.83
1.92
3.96
3.98
0.94
1.02
1.93
3.00
0.99
1.94
5.98
4.19
2.02
9.58
6.78
5.07
8.60
f1 (ppm)

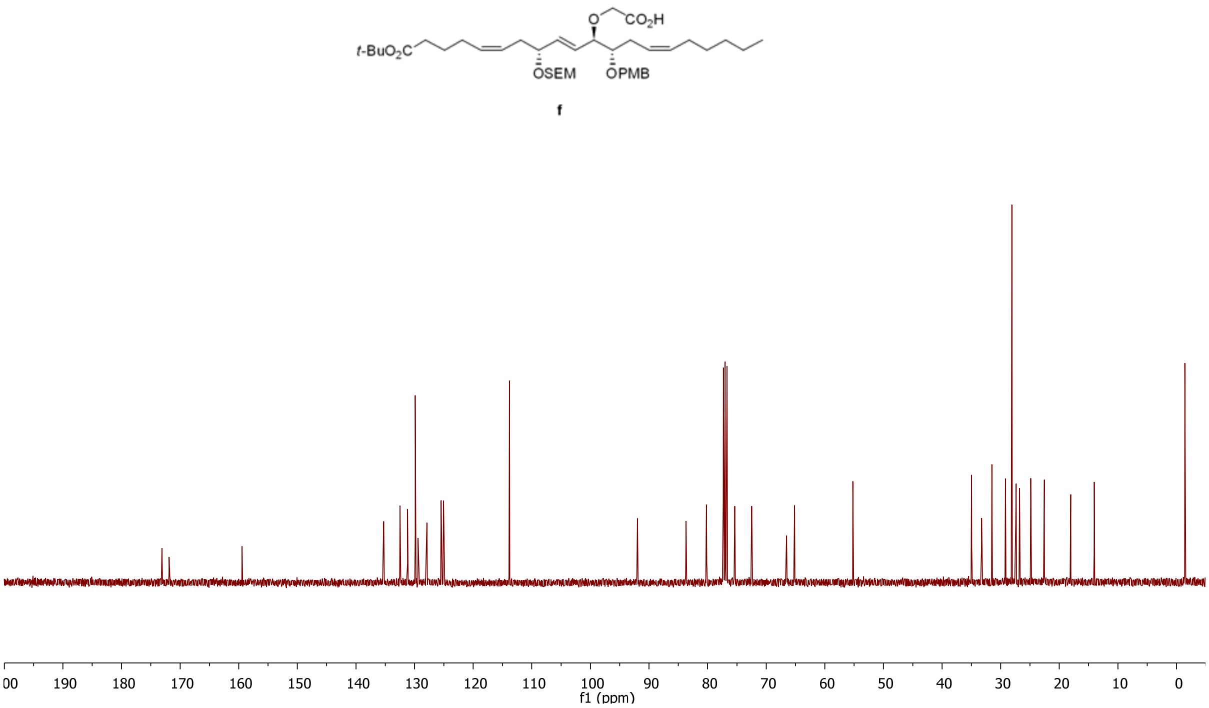
t-BuO2C
CO2H
OSEM
OPMB
f
f1 (ppm)

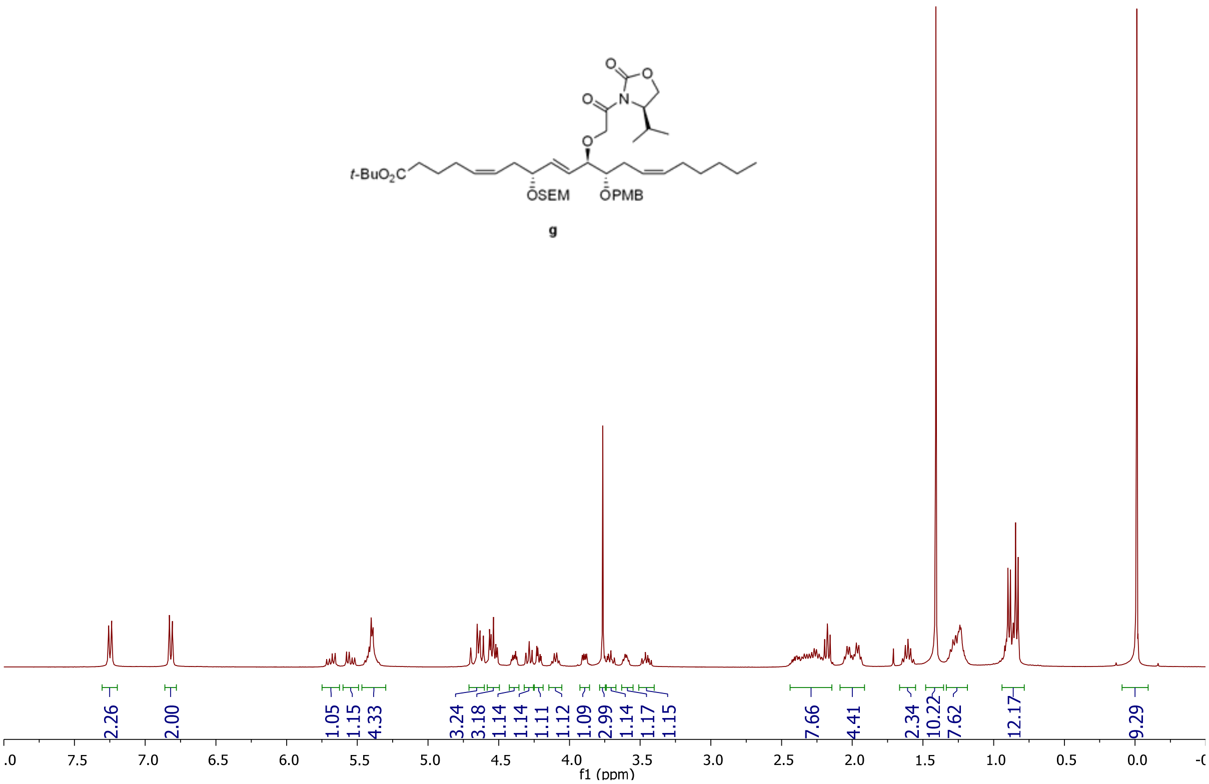
O
O
O
N
O
O
t-BuO2C
OSEM
OPMB
g
2.26
2.00
1.05
1.15
4.33
3.24
3.18
1.14
1.14
1.11
1.12
1.09
2.99
1.14
1.17
1.15
7.66
4.41
2.34
10.22
7.62
12.17
9.29
f1 (ppm)

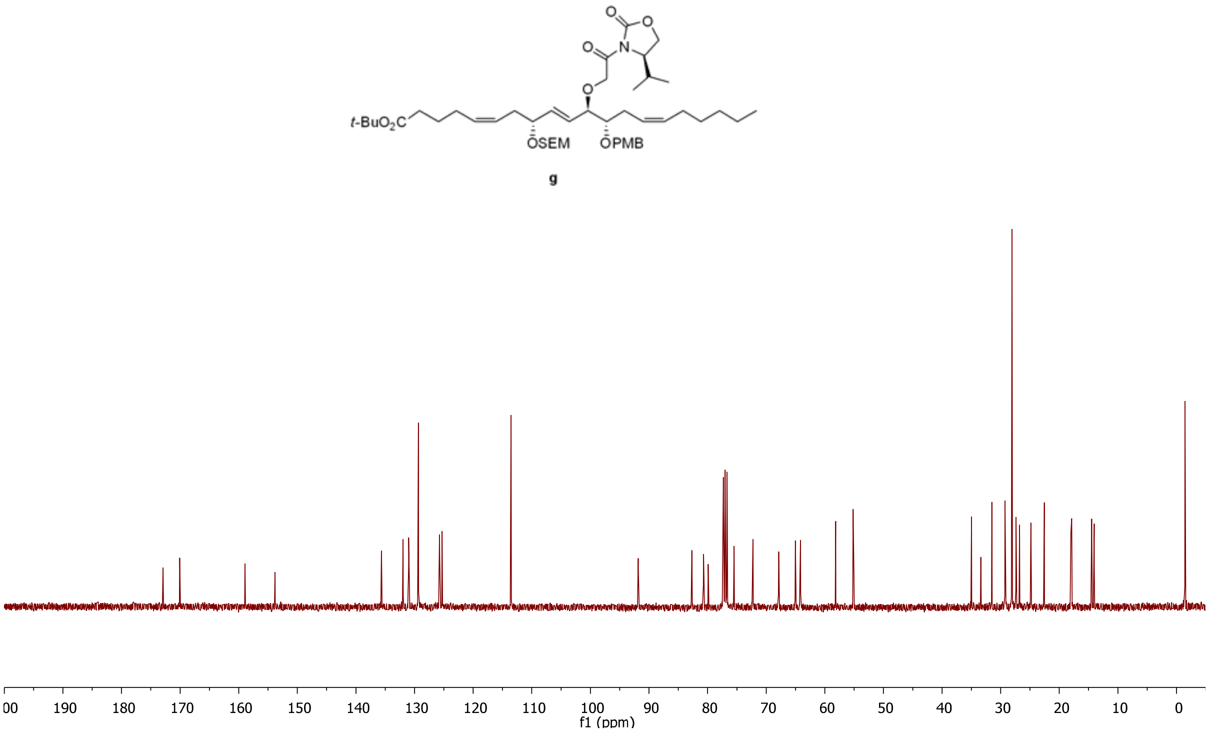
O
O
O
N
O
O
t-BuO2C
OSEM
OPMB
g
f1 (ppm)

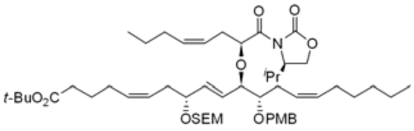

15

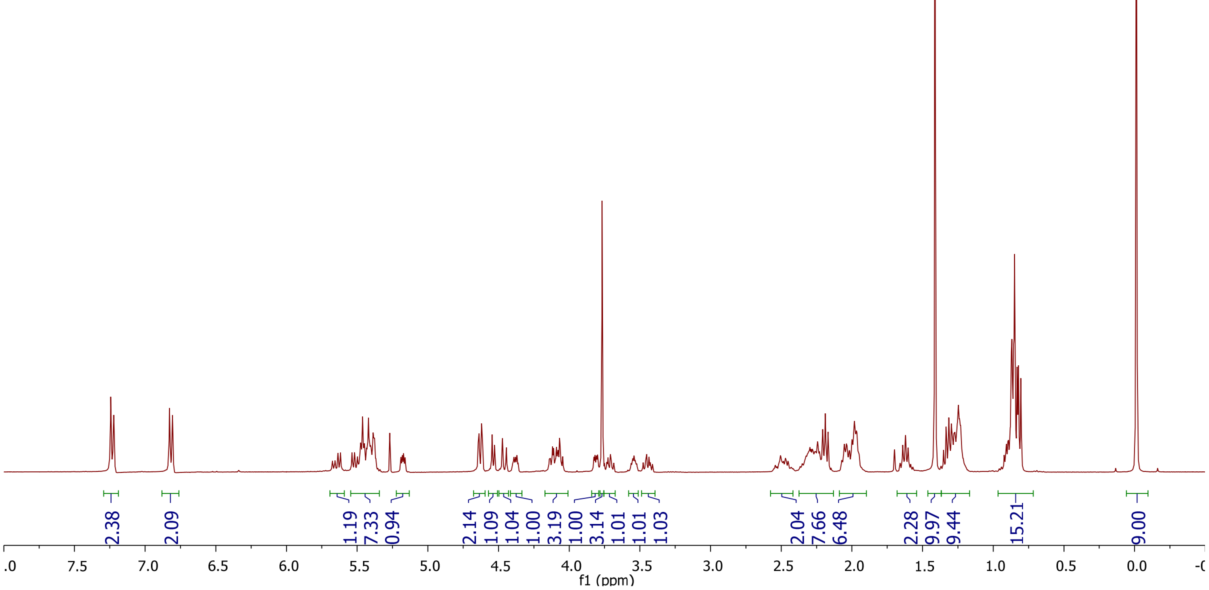

2.38
2.09
1.19
7.33
0.94
2.14
1.09
1.04
1.00
3.19
1.00
3.14
1.01
1.03
2.04
7.66
6.48
2.28
9.97
9.44
15.21
9.00
f1 (ppm)

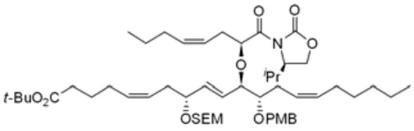

15

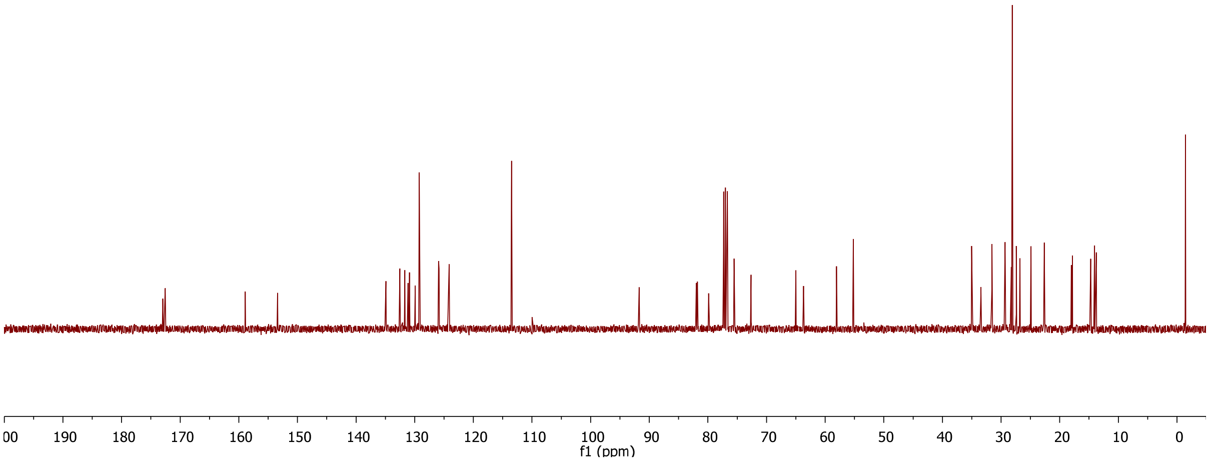

f1 (ppm)

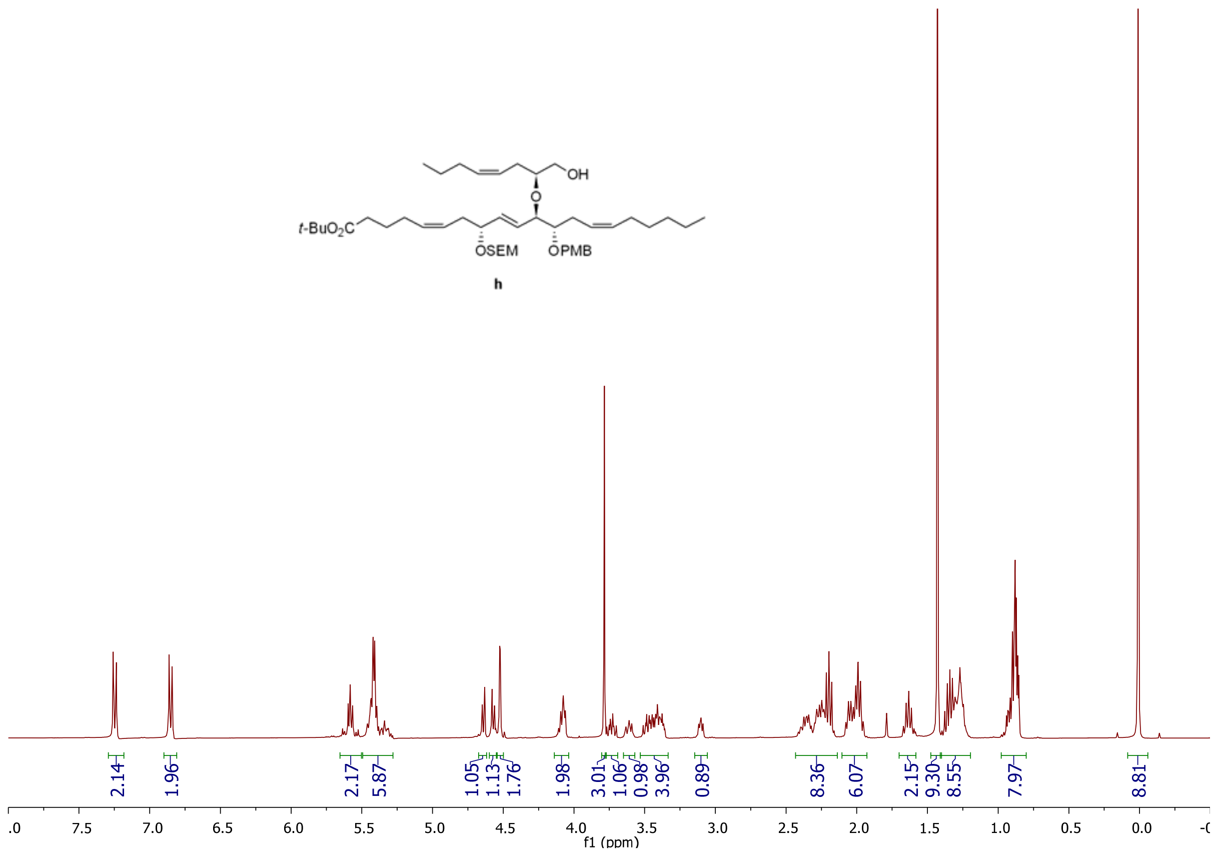
OH
O
t-BuO2C
OSEM
OPMB
h
2.14
1.96
2.17
5.87
1.05
1.13
1.76
1.98
3.01
1.06
0.98
3.96
0.89
8.36
6.07
2.15
9.30
8.55
7.97
8.81
f1 (ppm)

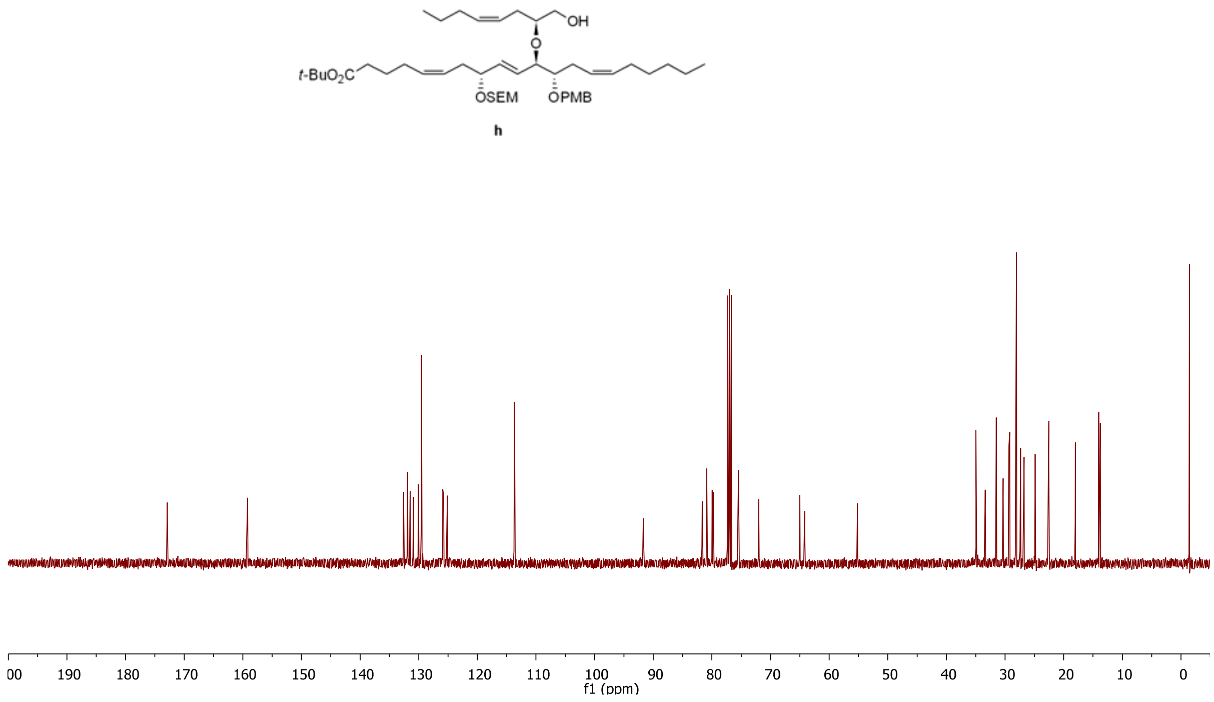
OH
O
t-BuO2C
OSEM
OPMB
h
f1 (ppm)

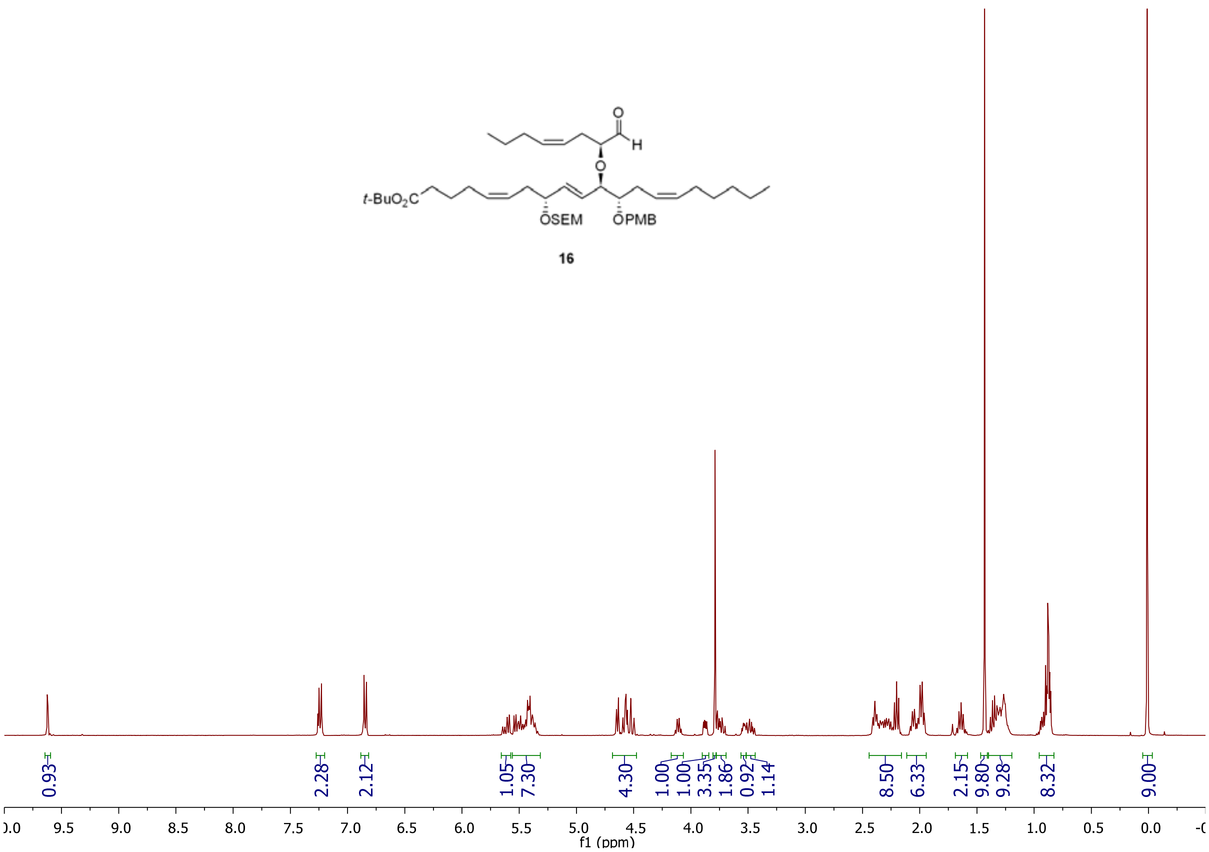
t-BuO2C
OSEM
OPMB
16
0.93
2.28
2.12
1.05
7.30
4.30
1.00
1.00
3.35
1.86
0.92
1.14
8.50
6.33
2.15
9.80
9.28
8.32
9.00
f1 (ppm)

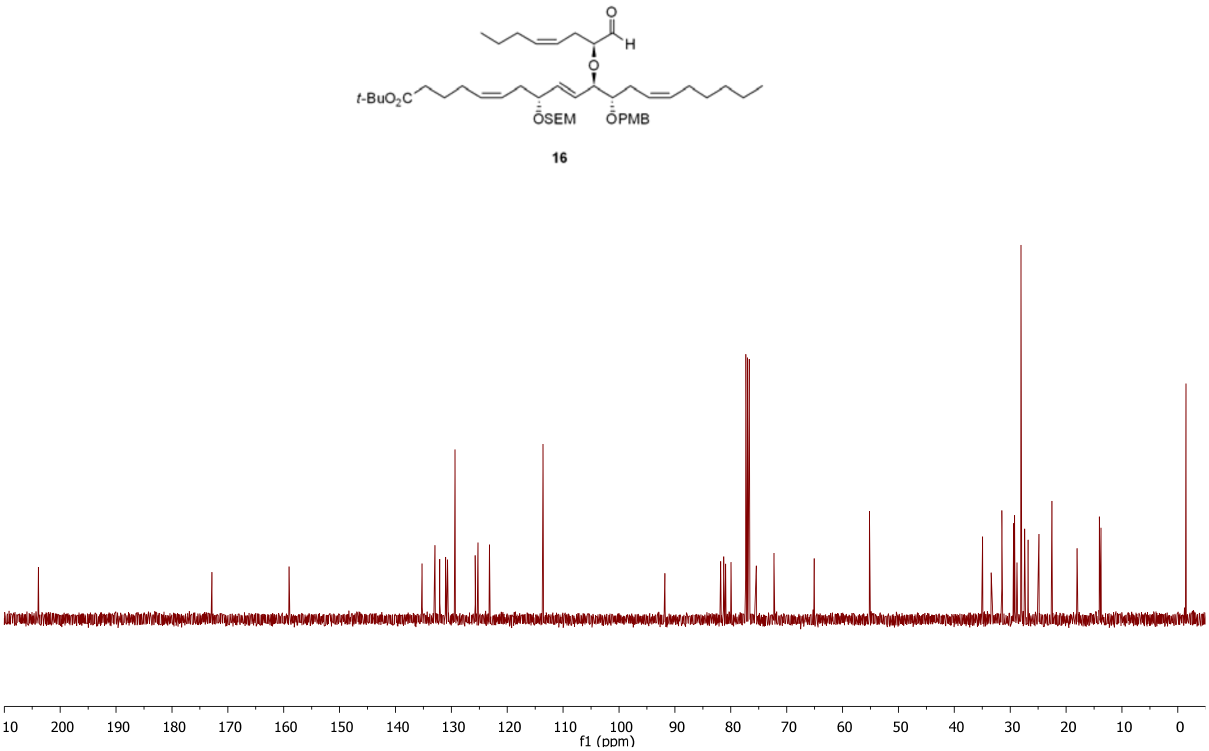
t-BuO2C
OSEM
OPMB
16
f1 (ppm)

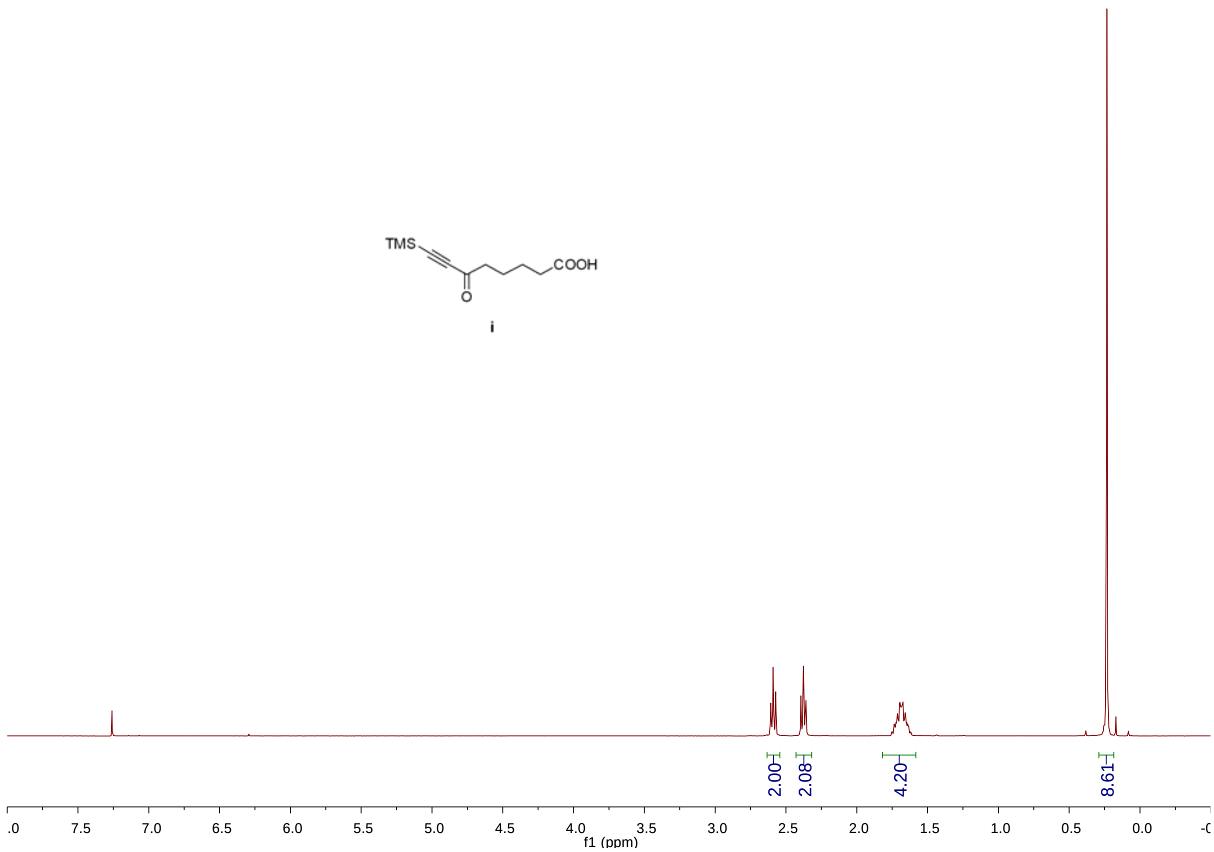
TMS
COOH
O
i
2.00
2.08
4.20
8.61
7.5
7.0
6.5
6.0
5.5
5.0
4.5
4.0
3.5
3.0
2.5
2.0
1.5
1.0
0.5
0.0
f1 (ppm)

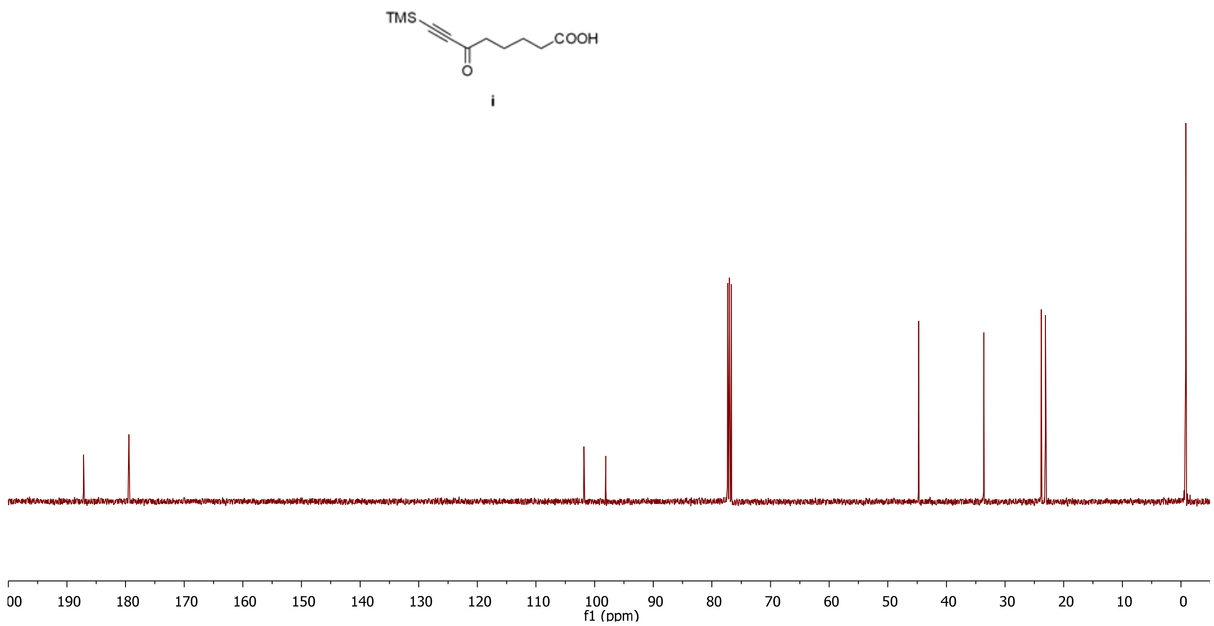
TMS
COOH
O
i
190
180
170
160
150
140
130
120
110
100
90
80
70
60
50
40
30
20
10
0
f1 (ppm)

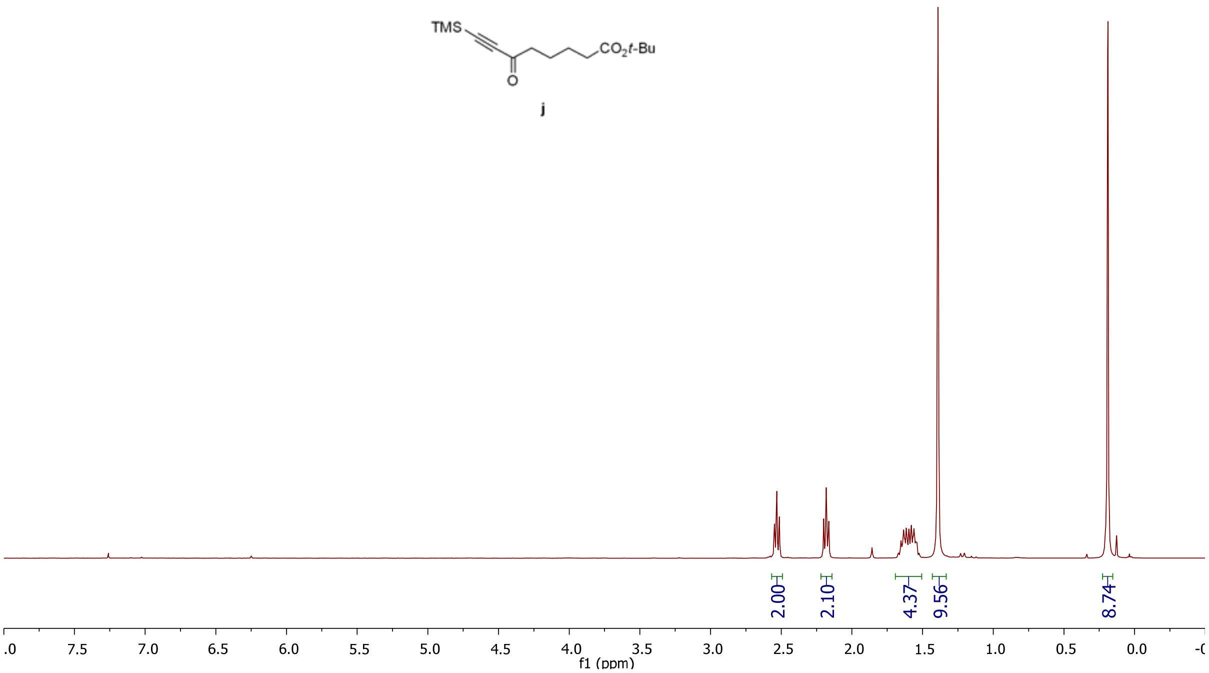
TMS
CO2t-Bu
O
j
2.00
2.10
4.37
9.56
8.74
f1 (ppm)

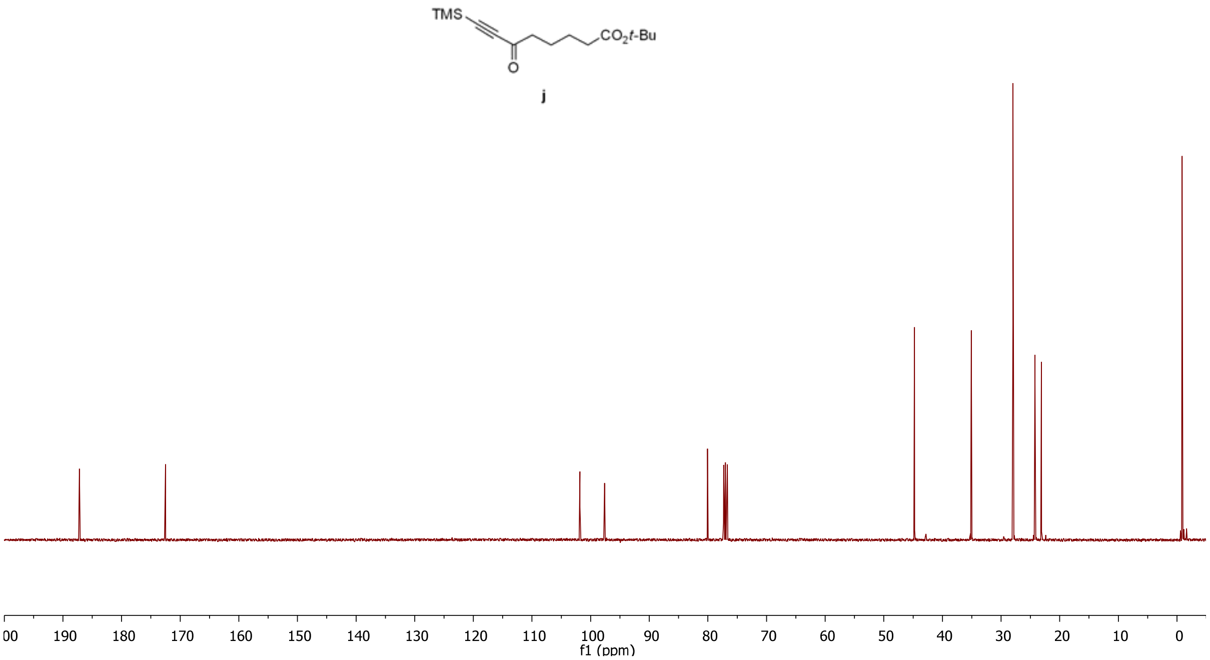
TMS
CO2t-Bu
O
j
f1 (ppm)

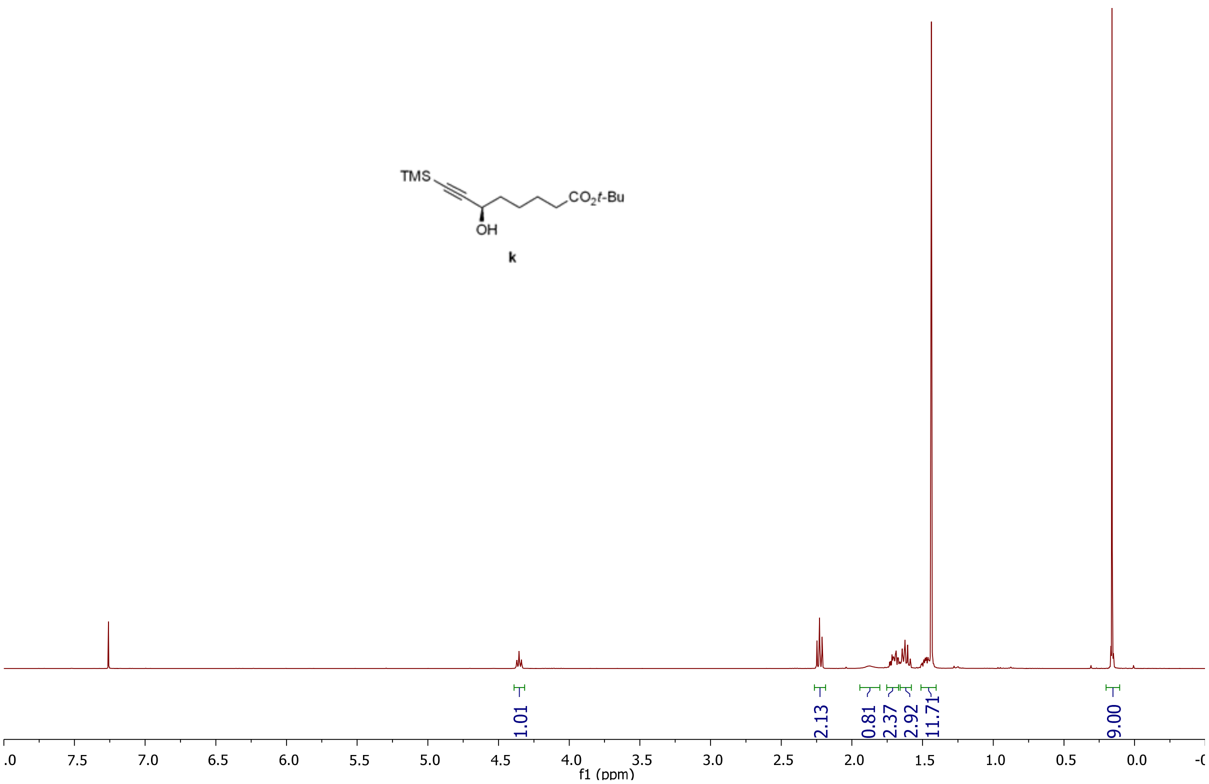
TMS
CO_2t-Bu
OH
k
1.01
2.13
0.81
2.37
2.92
11.71
9.00
f1 (ppm)

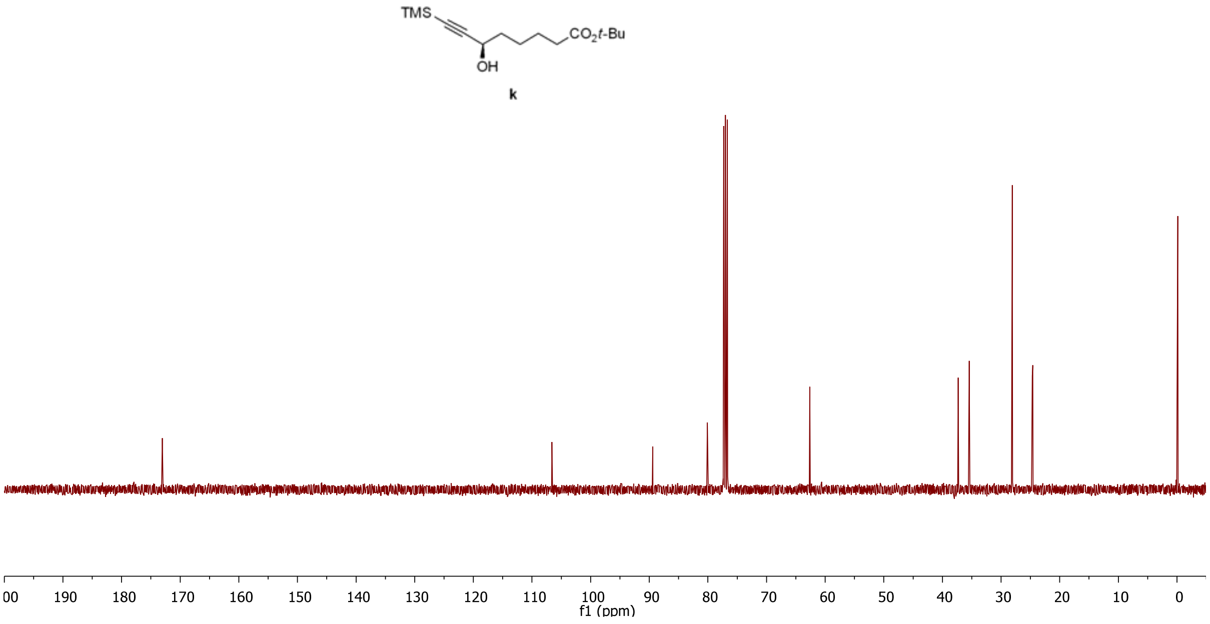
TMS
CO_2t-Bu
OH
k
f1 (ppm)

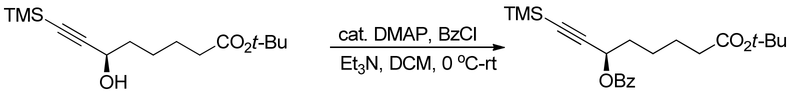

k **l**

Racemate

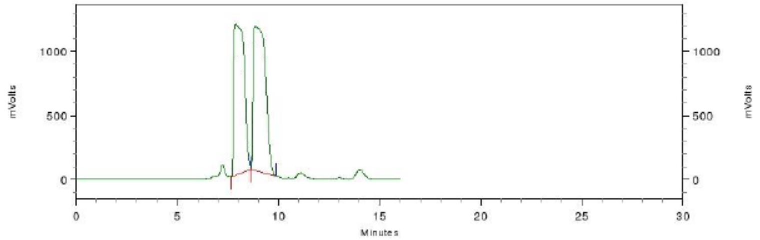

UV Detector
Ch2-229nm
Results

Retention Time	Area	Area %	Height	Height %
7.883	42989666	47.38	1174800	51.11
8.850	47741692	52.62	1123842	48.89
Totals	90731358	100.00	2298642	100.00

HPLC Conditions: Chiralcel OD-H column, 1 mL/ min flow rate, 1% i-PrOH in hexanes

Scalemic Compound

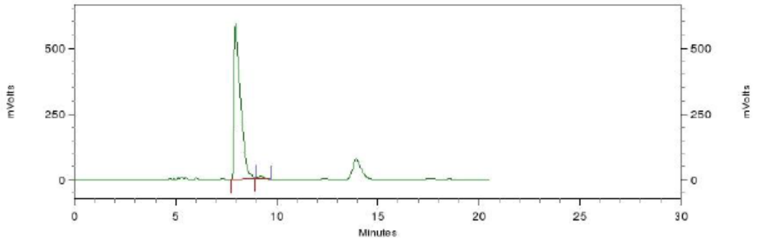

UV Detector
Ch1-254nm
Results

Retention Time	Area	Area %	Height	Height %
7.950	13252711	98.70	588536	98.51
9.183	174405	1.30	8931	1.49
Totals	13427116	100.00	597467	100.00

HPLC Chromatogram for compound **l**

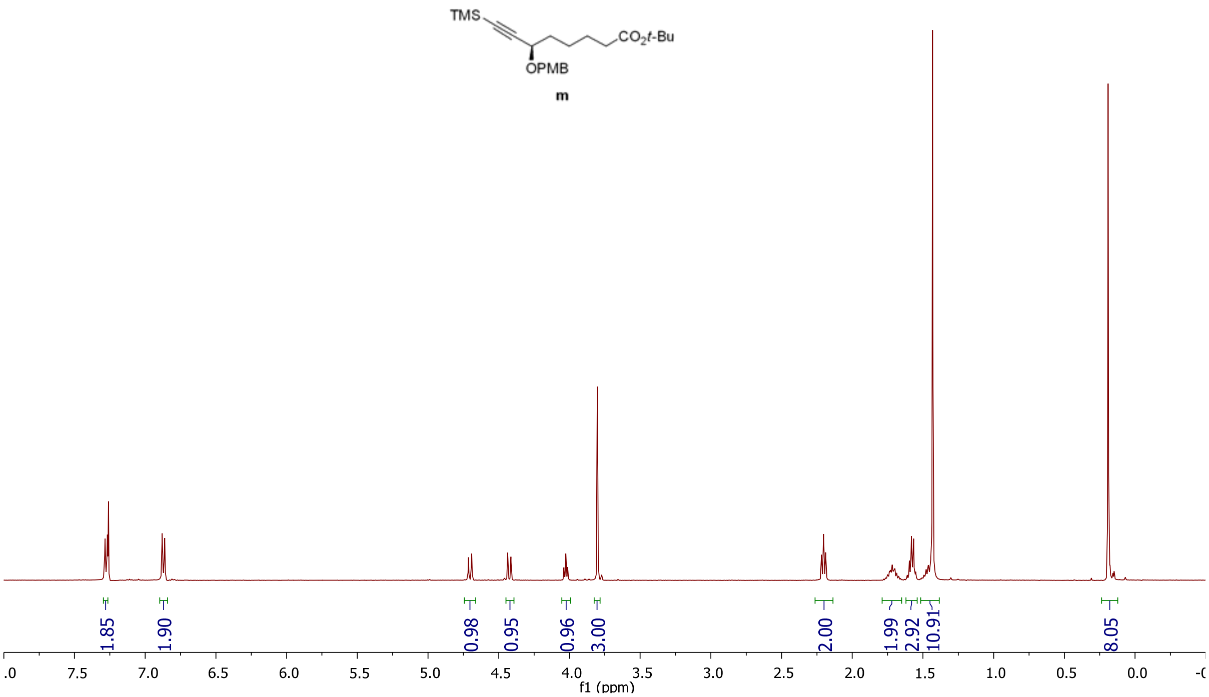
TMS
CO_2t-Bu
OPMB
m
1.85
1.90
0.98
0.95
0.96
3.00
2.00
1.99
2.92
10.91
8.05
7.5
7.0
6.5
6.0
5.5
5.0
4.5
4.0
3.5
3.0
2.5
2.0
1.5
1.0
0.5
0.0
f1 (ppm)

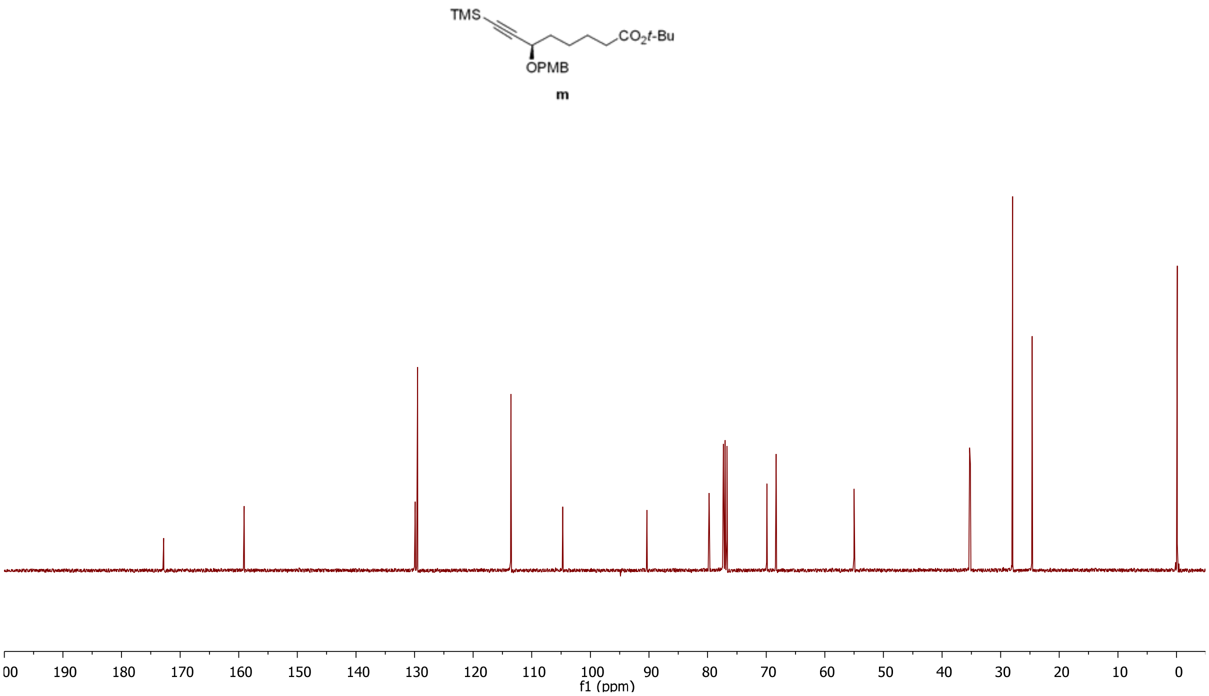
TMS
CO_2t-Bu
OPMB
m
190
180
170
160
150
140
130
120
110
100
90
80
70
60
50
40
30
20
10
0
f1 (ppm)

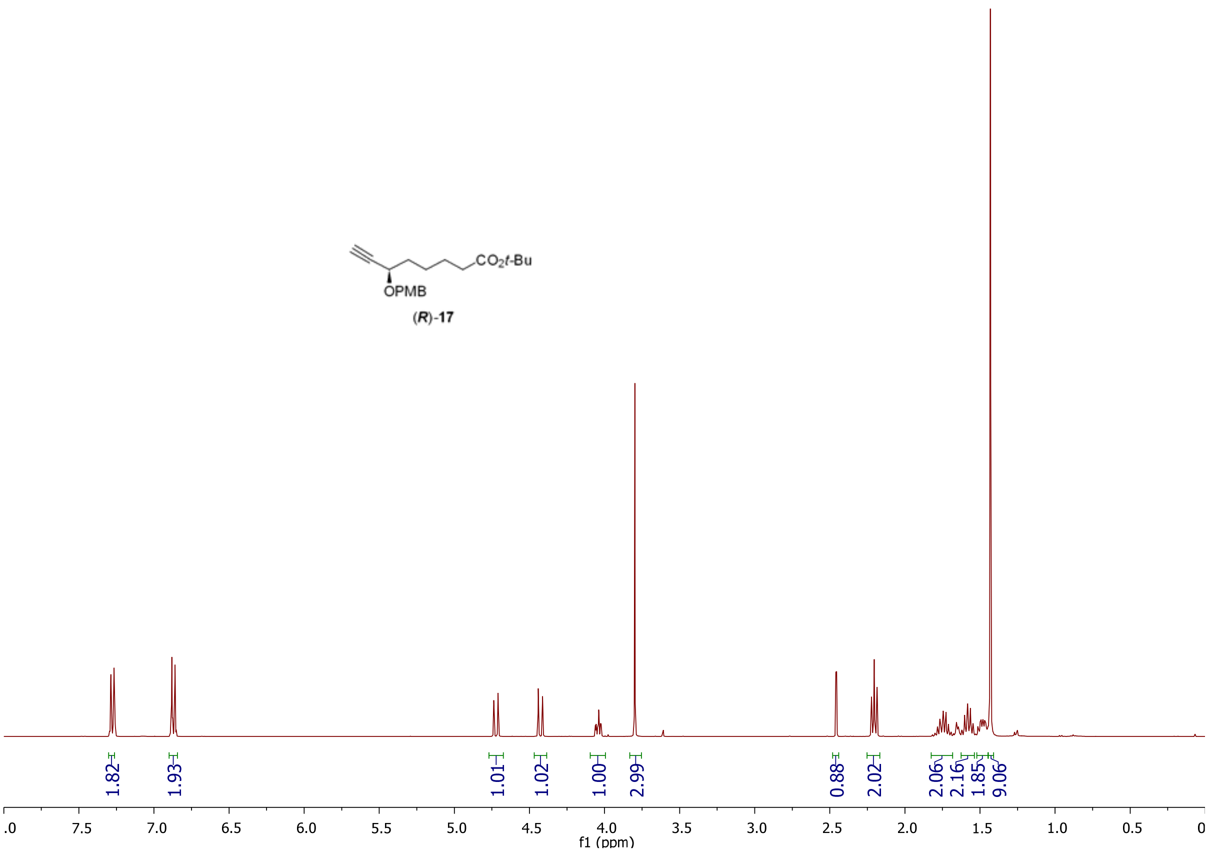
CO2t-Bu
OPMB
(R)-17
1.82
1.93
1.01
1.02
1.00
2.99
0.88
2.02
2.06
2.16
1.85
9.06
7.5
7.0
6.5
6.0
5.5
5.0
4.5
4.0
3.5
3.0
2.5
2.0
1.5
1.0
0.5
f1 (ppm)

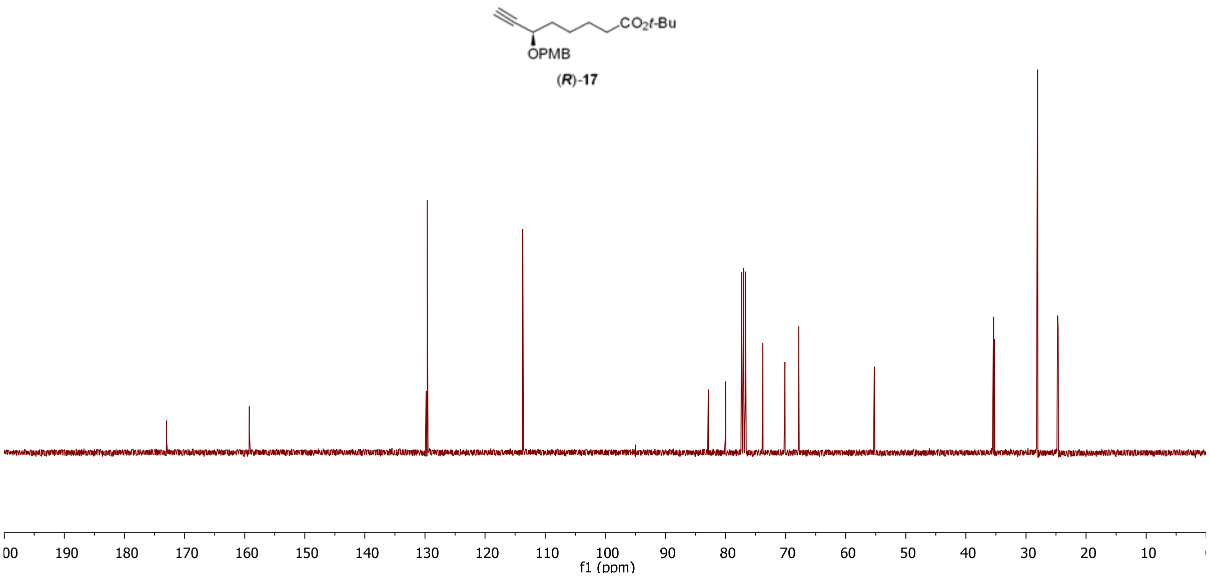
CO2t-Bu
OPMB
(R)-17
190
180
170
160
150
140
130
120
110
100
90
80
70
60
50
40
30
20
10
f1 (ppm)

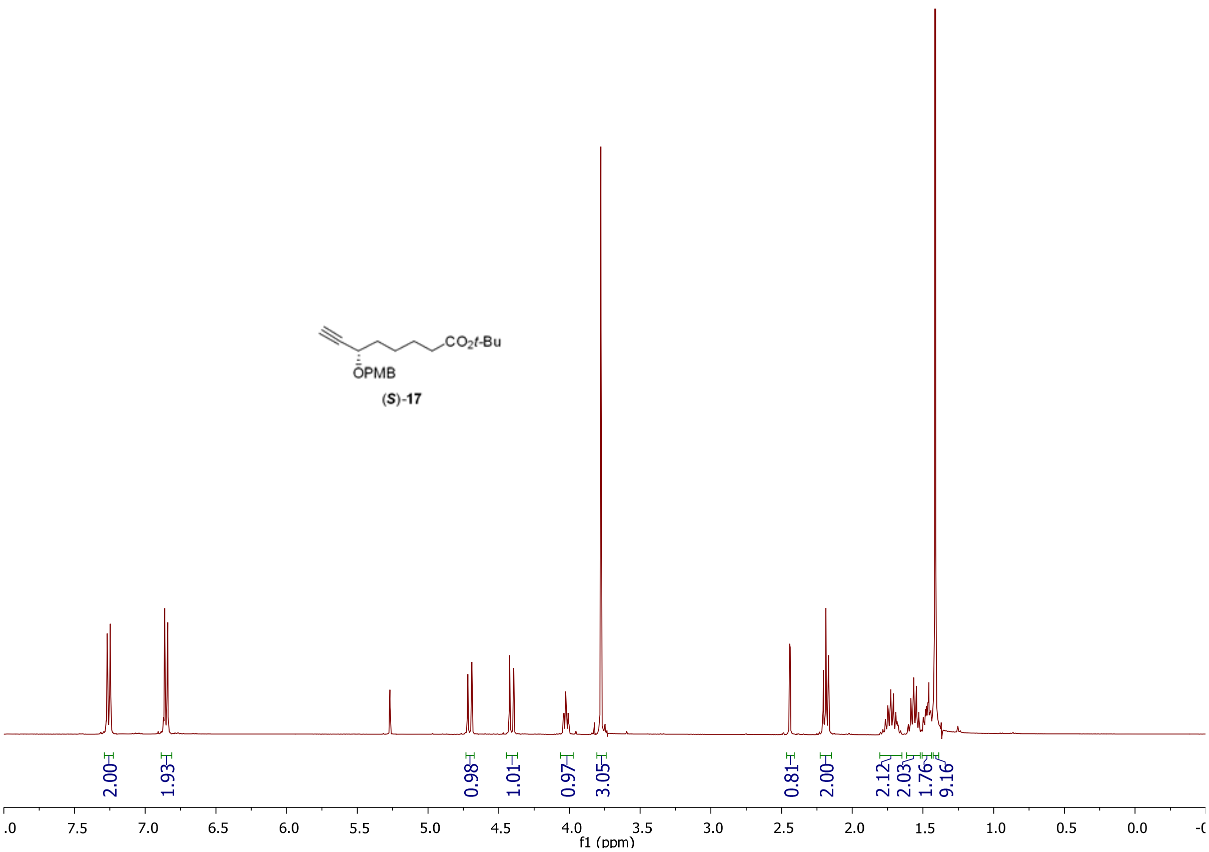
CO2t-Bu
OPMB
(S)-17
2.00
1.93
0.98
1.01
0.97
3.05
0.81
2.00
2.12
2.03
1.76
9.16
7.5
7.0
6.5
6.0
5.5
5.0
4.5
4.0
3.5
3.0
2.5
2.0
1.5
1.0
0.5
0.0
f1 (ppm)

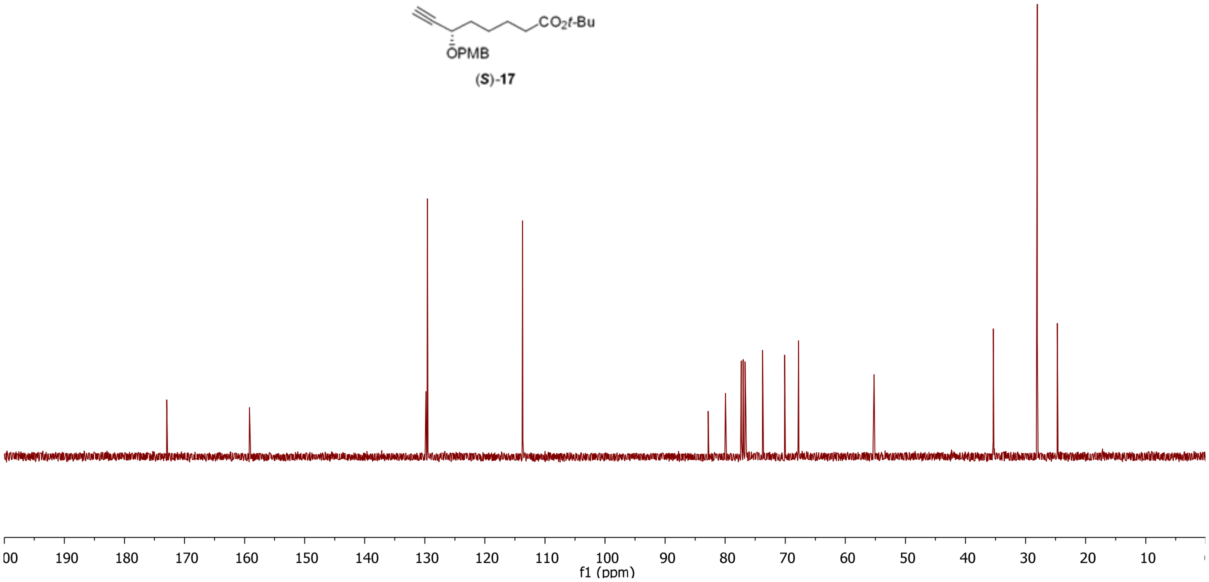
CO2t-Bu
OPMB
(S)-17
190
180
170
160
150
140
130
120
110
100
90
80
70
60
50
40
30
20
10
f1 (ppm)

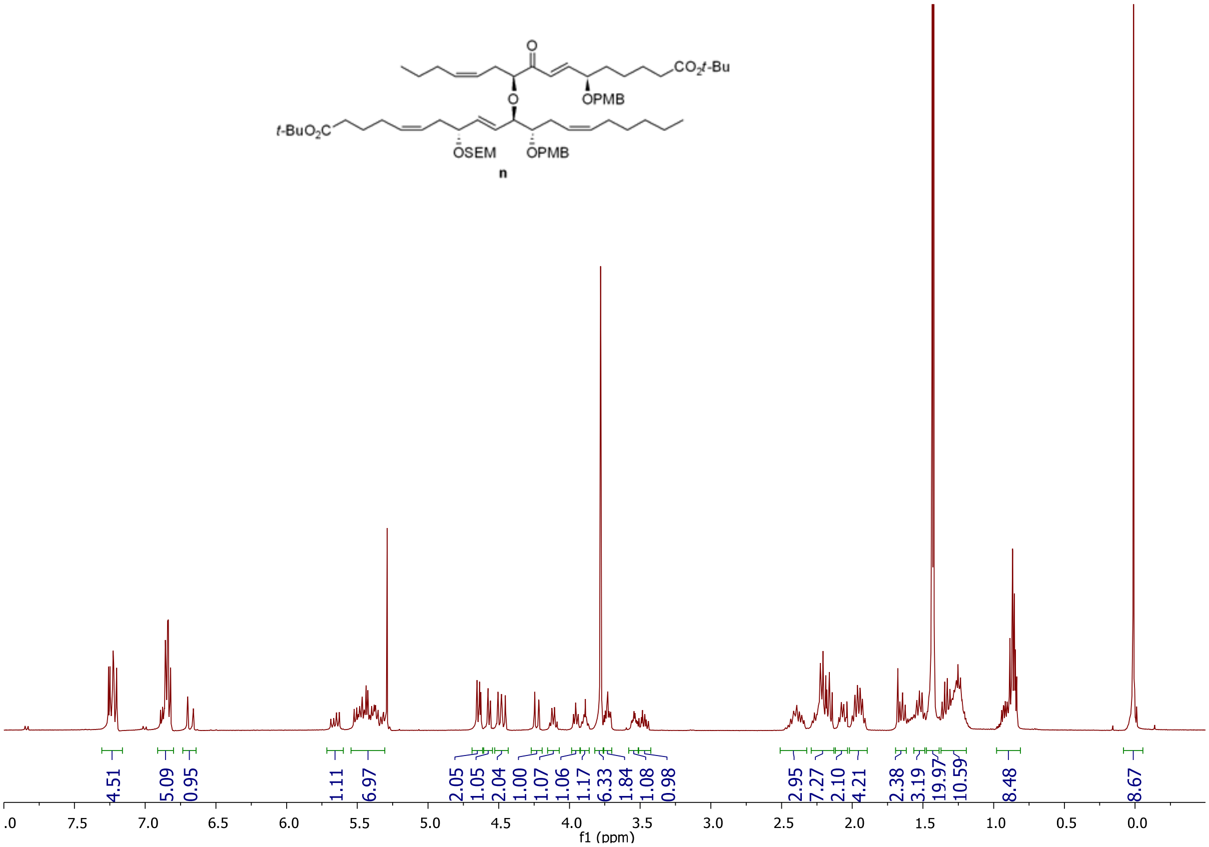
CO2t-Bu
OPMB
t-BuO2C
OSEM
OPMB
n
4.51
5.09
0.95
1.11
6.97
2.05
1.05
2.04
1.00
1.07
1.06
1.17
6.33
1.84
1.08
0.98
2.95
7.27
2.10
4.21
2.38
3.19
19.97
10.59
8.48
8.67
f1 (ppm)

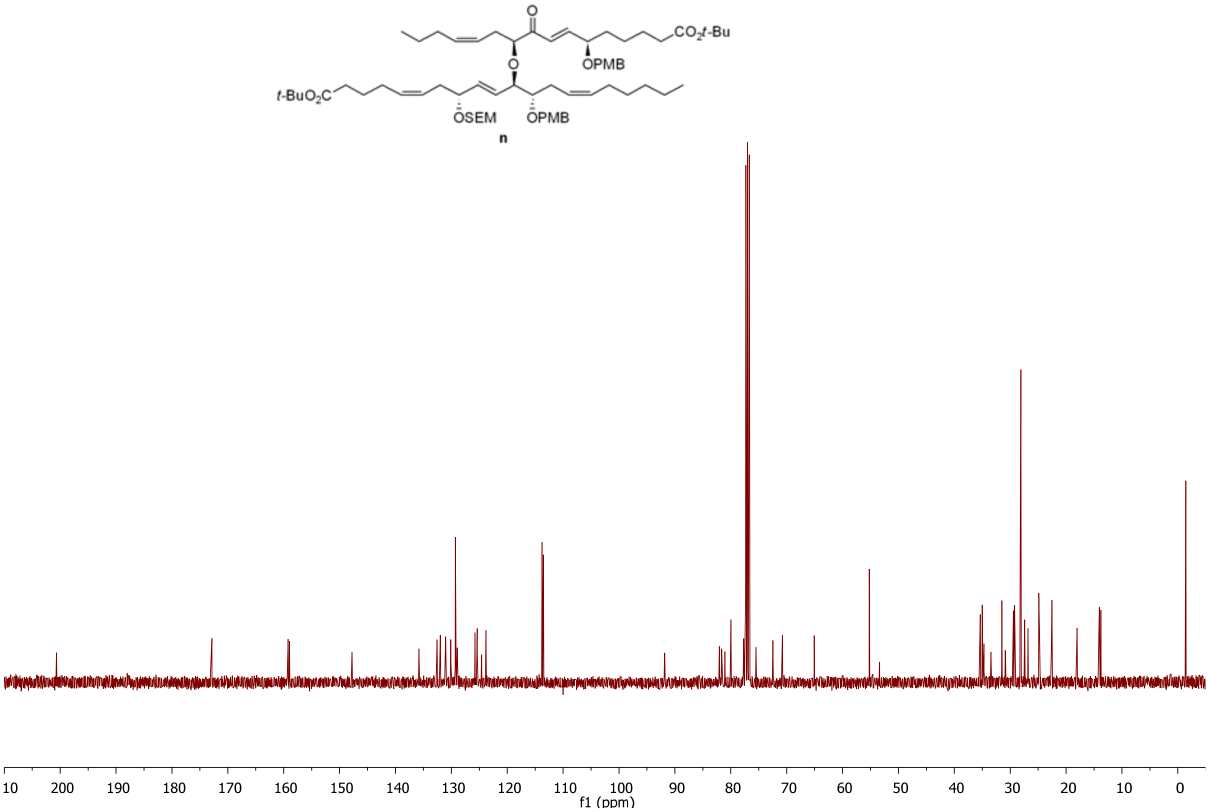
CO2t-Bu
OPMB
t-BuO2C
OSEM
OPMB
n
f1 (ppm)

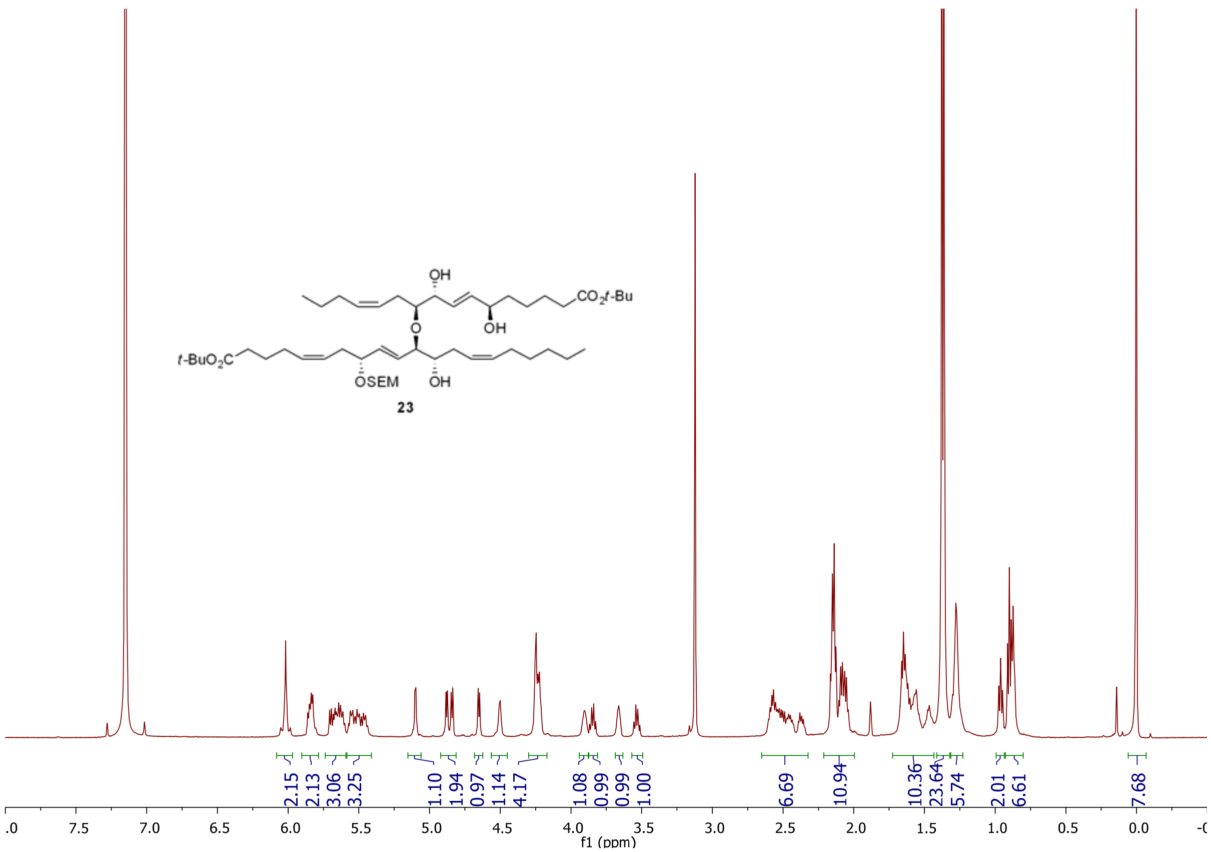

OH
CO2t-Bu
O
OH
t-BuO2C
OSEM
OH
23
2.15
2.13
3.06
3.25
1.10
1.94
0.97
1.14
4.17
1.08
0.99
0.99
1.00
6.69
10.94
10.36
23.64
5.74
2.01
6.61
7.68
f1 (ppm)

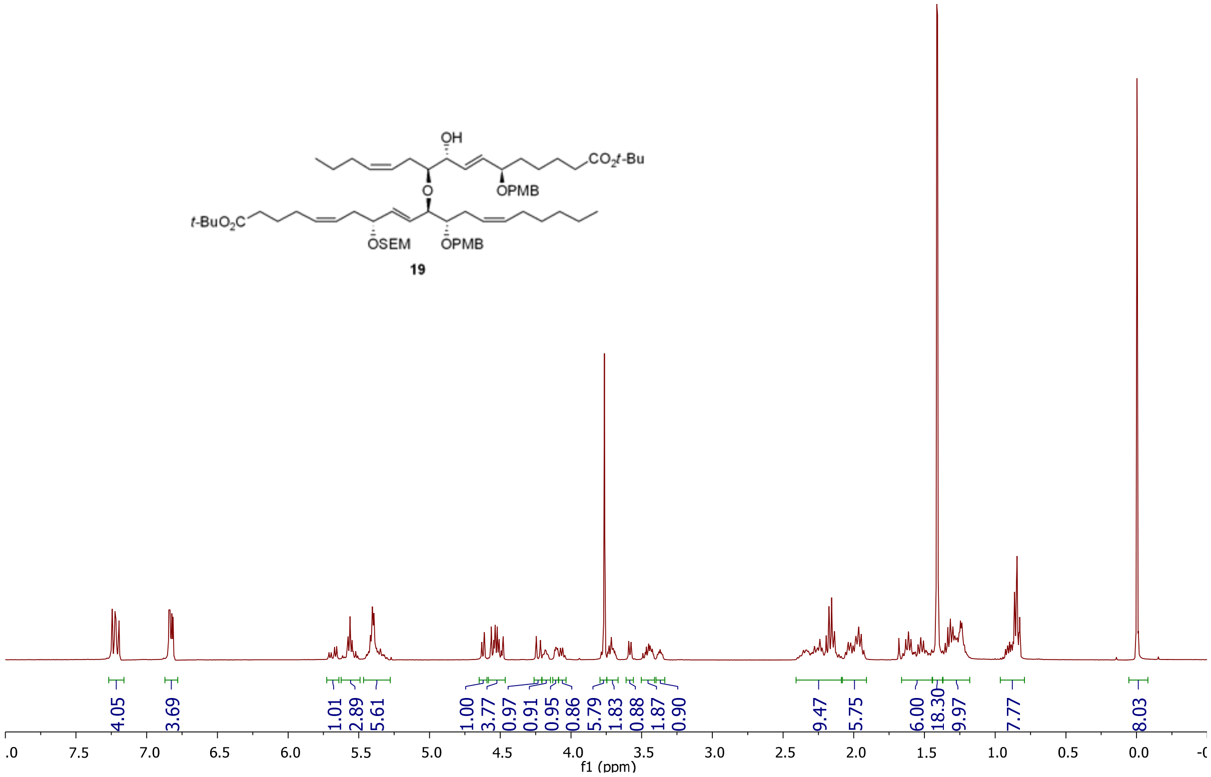

OH
CO2t-Bu
O
OPMB
t-BuO2C
OSEM
OPMB
19
4.05
3.69
1.01
2.89
5.61
1.00
3.77
0.97
0.91
0.95
0.86
5.79
1.83
0.88
1.87
0.90
9.47
5.75
6.00
18.30
9.97
7.77
8.03
f1 (ppm)

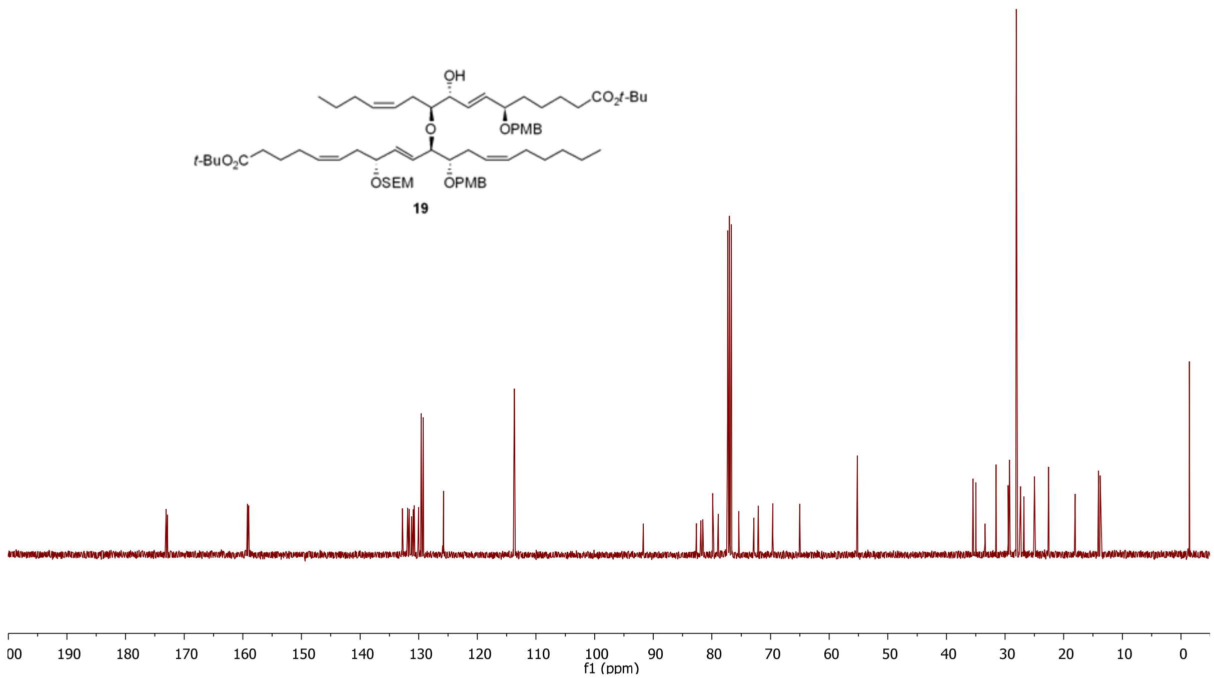
OH
CO_2t-Bu
OPMB
t-BuO_2C
OSEM
OPMB
19
f1 (ppm)

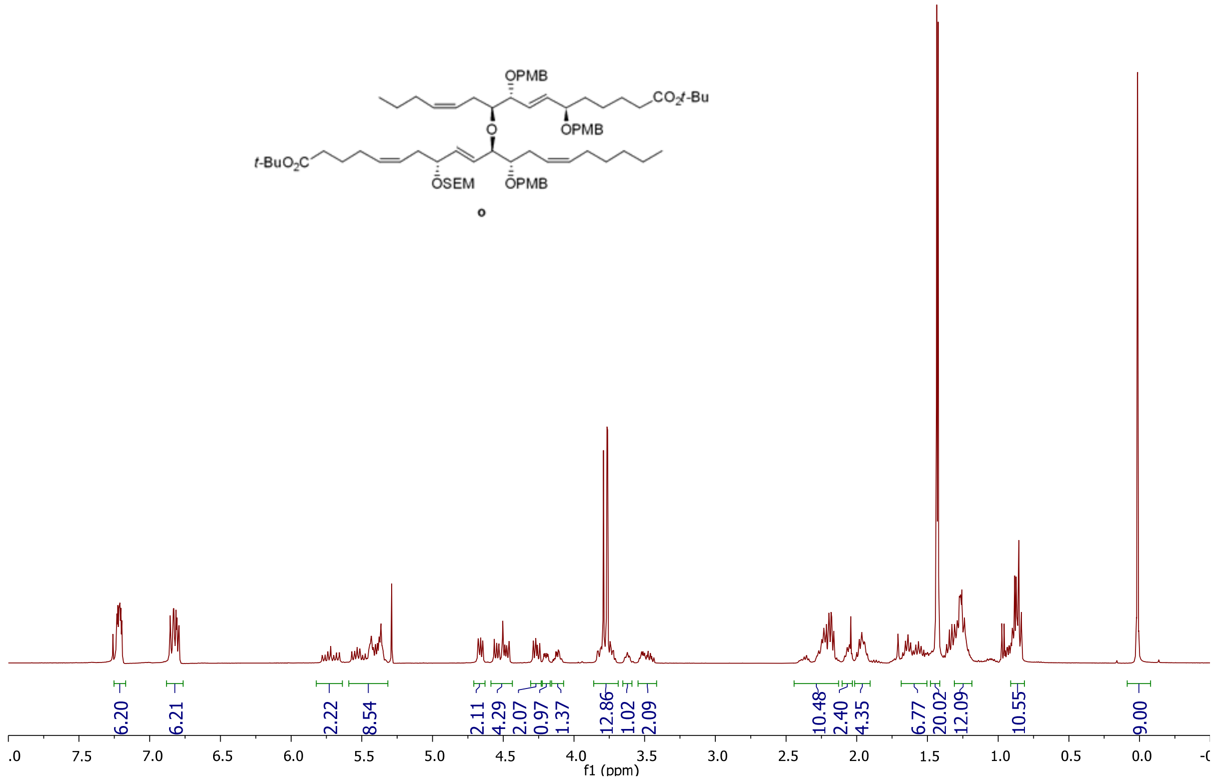
OPMB
CO_2t-Bu
OPMB
t-BuO_2C
OSEM
OPMB
o
6.20
6.21
2.22
8.54
2.11
4.29
2.07
0.97
1.37
12.86
1.02
2.09
10.48
2.40
4.35
6.77
20.02
12.09
10.55
9.00
f1 (ppm)

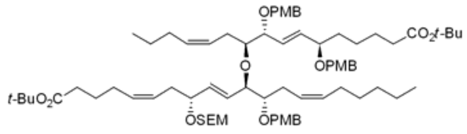
OPMB
CO2t-Bu
OPMB
O
t-BuO2C
OSEM
OPMB

o

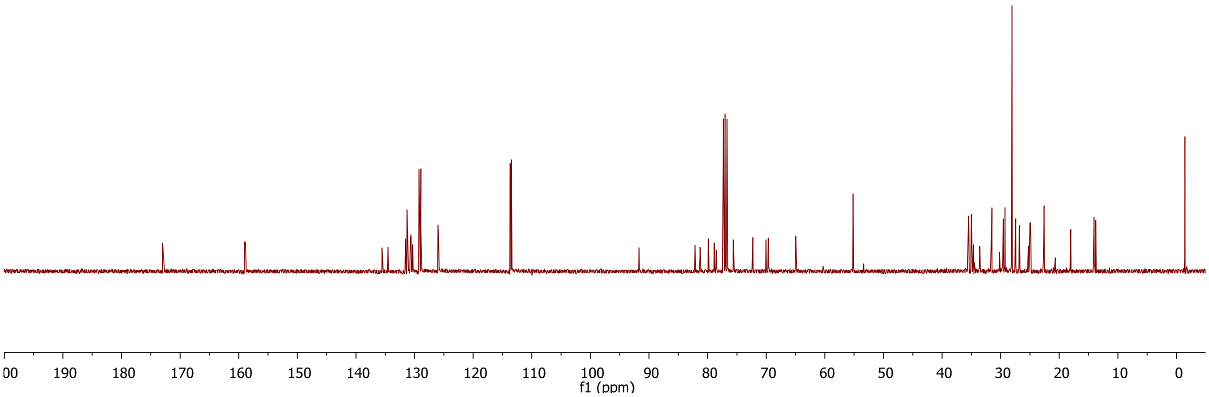
f1 (ppm)

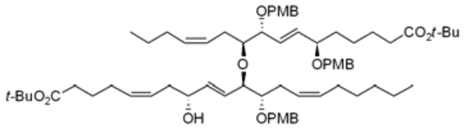
OPMB
CO2t-Bu
OPMB
O
t-BuO2C
OH
OPMB

26

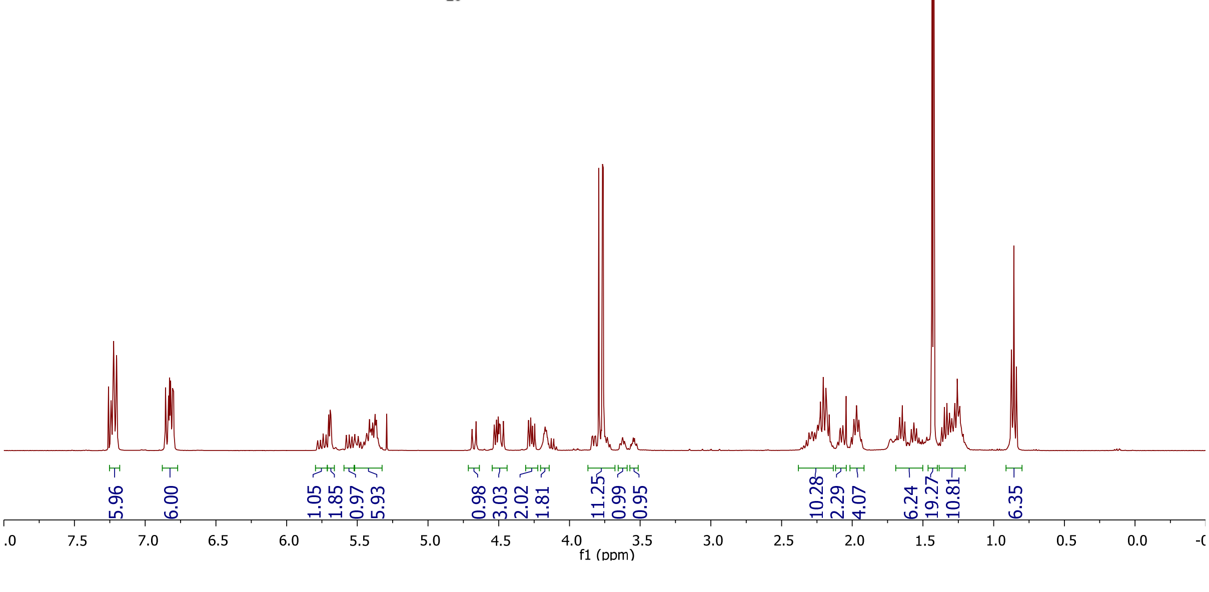
5.96
6.00
1.05
1.85
0.97
5.93
0.98
3.03
2.02
1.81
11.25
0.99
0.95
10.28
2.29
4.07
6.24
19.27
10.81
6.35
f1 (ppm)

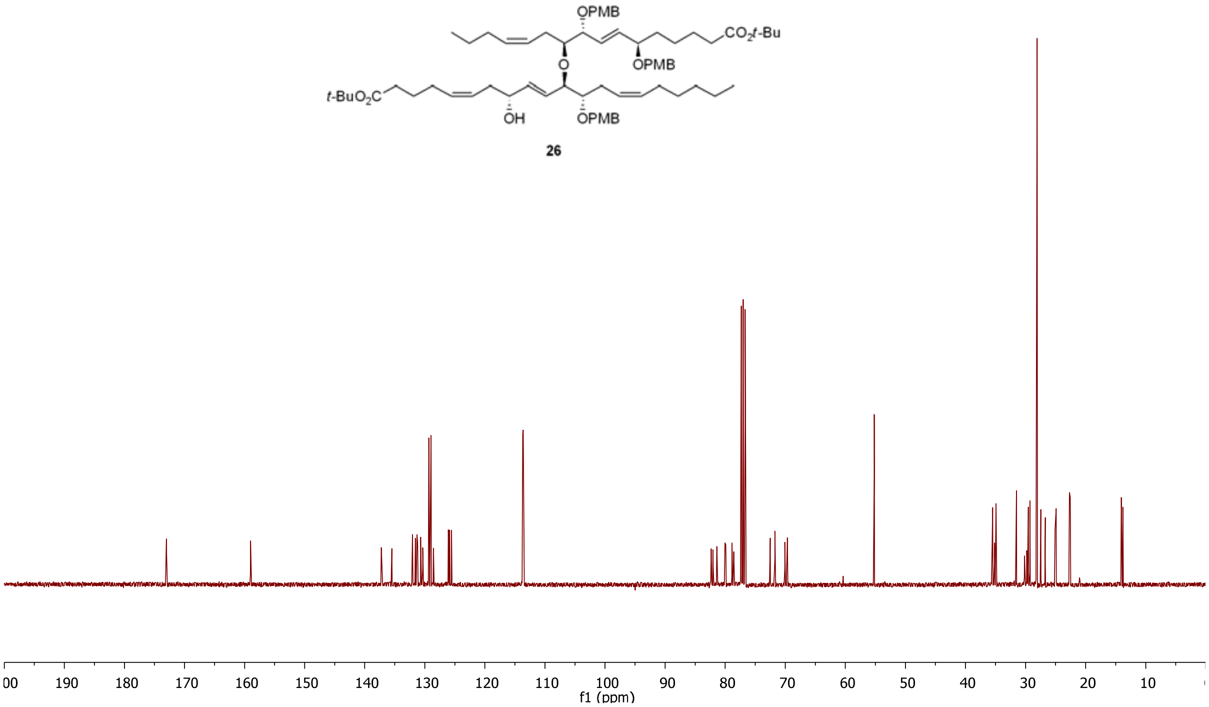
OPMB
CO2t-Bu
OPMB
O
t-BuO2C
OH
OPMB
26
f1 (ppm)

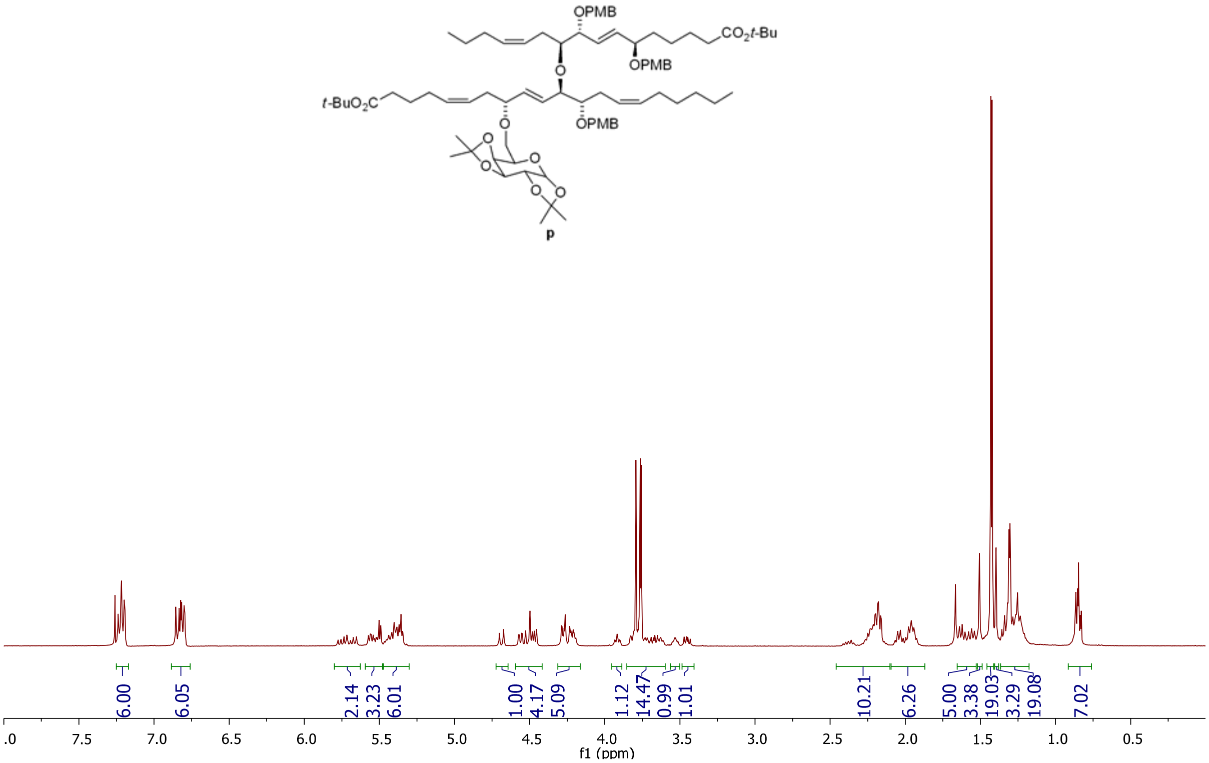
OPMB
CO2t-Bu
OPMB
O
t-BuO2C
O
OPMB
p
6.00
6.05
2.14
3.23
6.01
1.00
4.17
5.09
1.12
14.47
0.99
1.01
10.21
6.26
5.00
3.38
19.03
3.29
19.08
7.02
f1 (ppm)

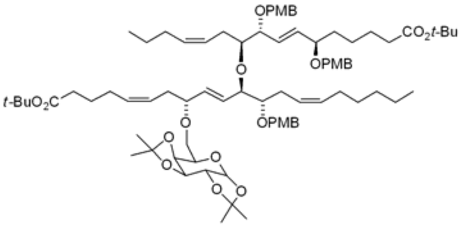
OPMB
CO_2t-Bu
OPMB
t-BuO_2C
OPMB

p

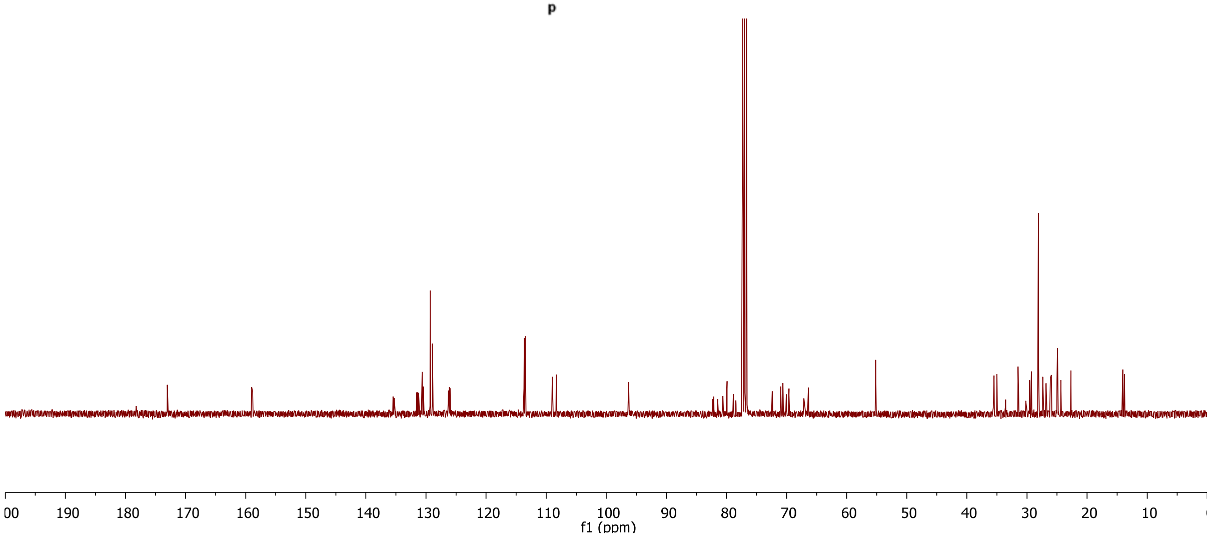
00 190 180 170 160 150 140 130 120 110 100 90 80 70 60 50 40 30 20 10
f1 (ppm)

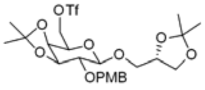
OTf
OPMB

28

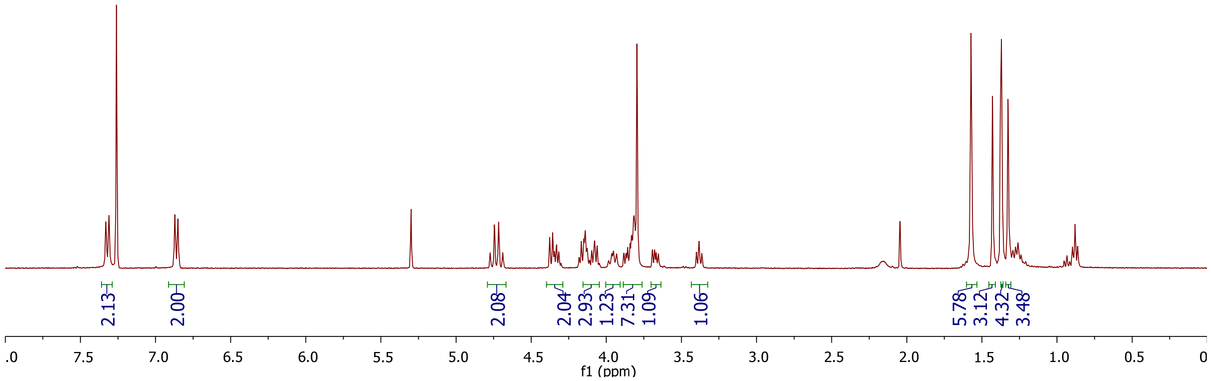
2.13
2.00
2.08
2.04
2.93
1.23
7.31
1.09
1.06
5.78
3.12
4.32
3.48
.0 7.5 7.0 6.5 6.0 5.5 5.0 4.5 4.0 3.5 3.0 2.5 2.0 1.5 1.0 0.5 0
f1 (ppm)

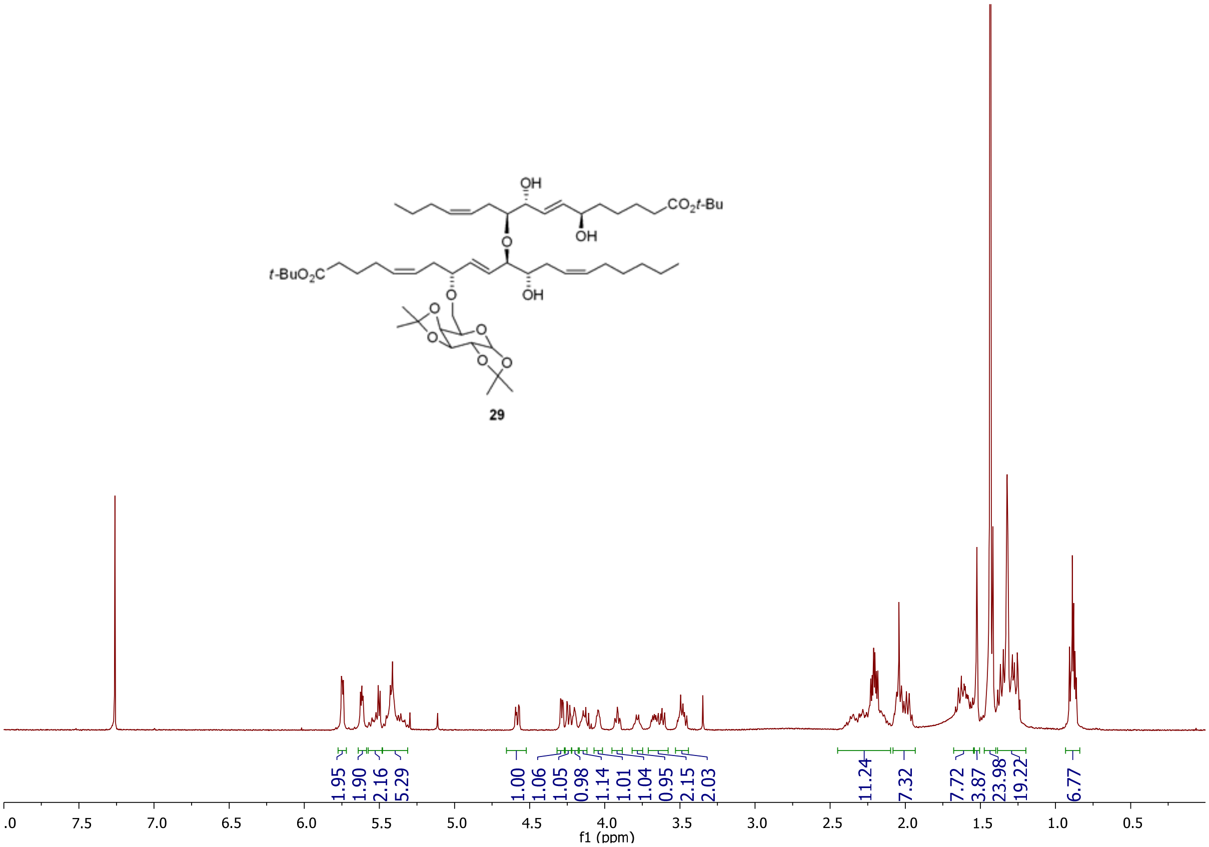
OH
CO2t-Bu
OH
O
t-BuO2C
O
OH
29
1.95
1.90
2.16
5.29
1.00
1.06
1.05
0.98
1.14
1.01
1.04
0.95
2.15
2.03
11.24
7.32
7.72
3.87
23.98
19.22
6.77
f1 (ppm)

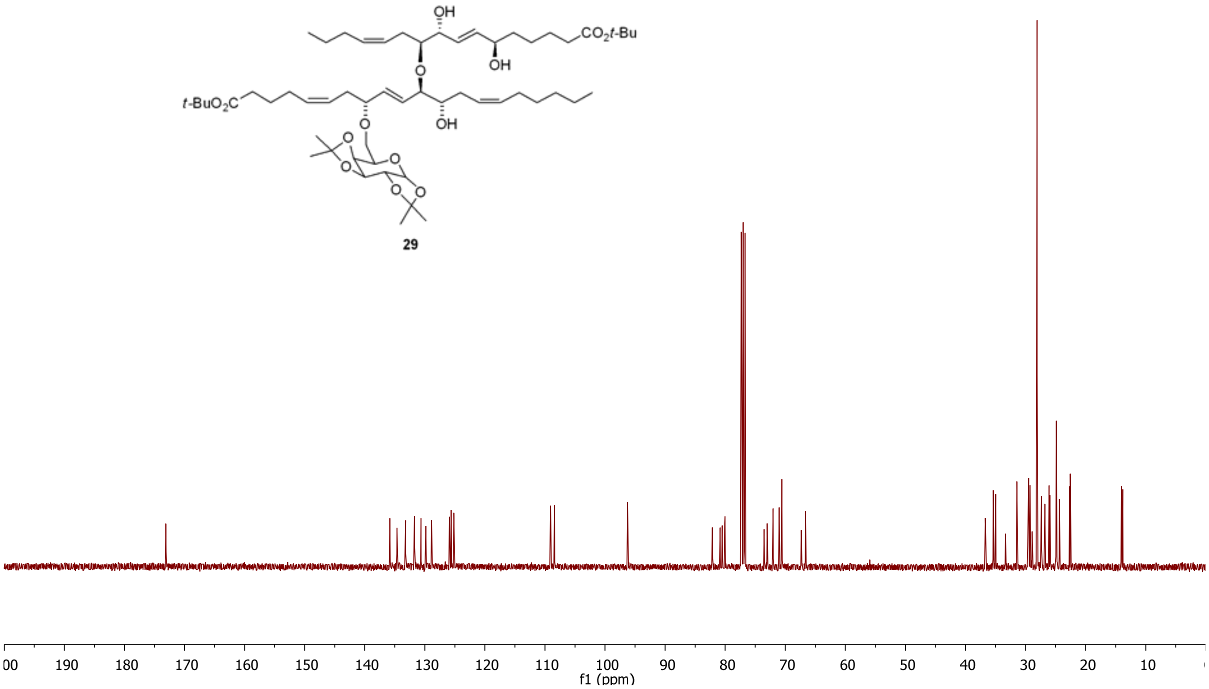
OH
CO2t-Bu
OH
O
t-BuO2C
O
OH
29
f1 (ppm)

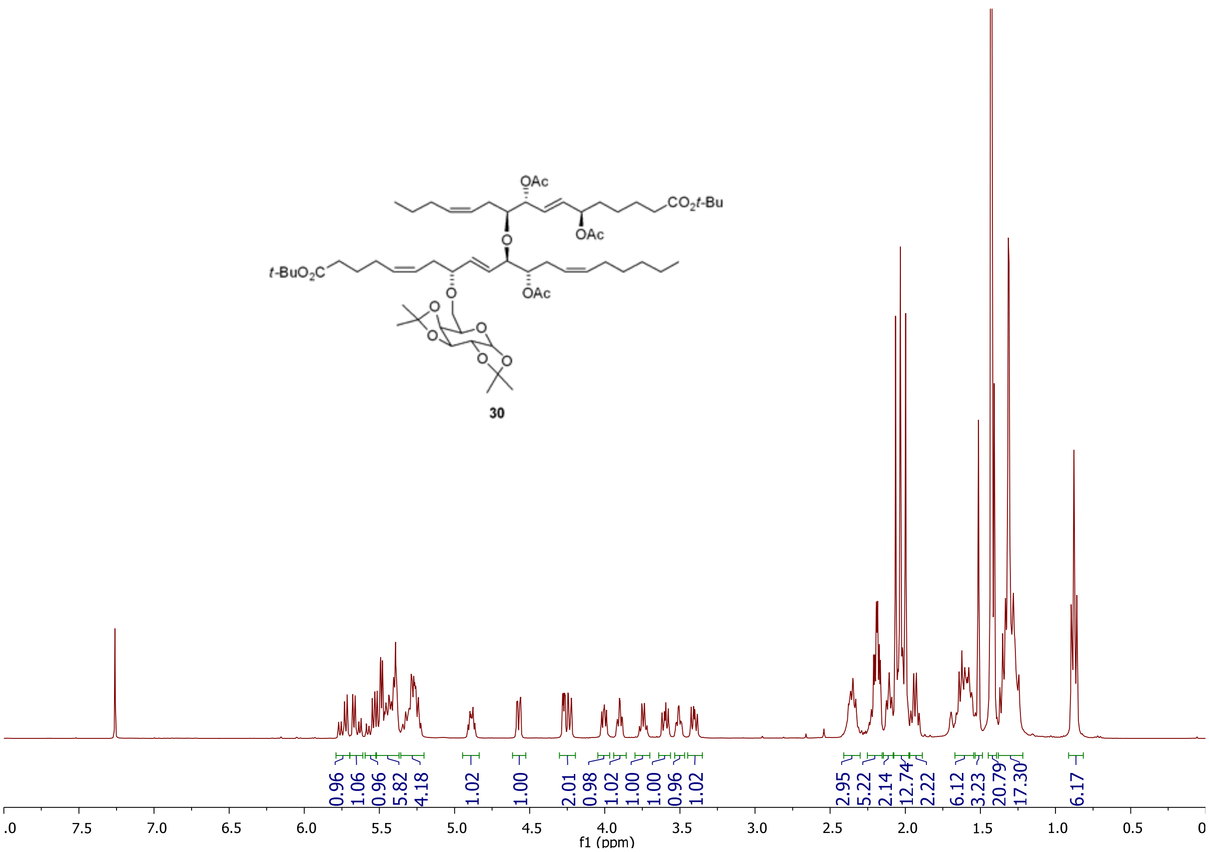

30
f1 (ppm)

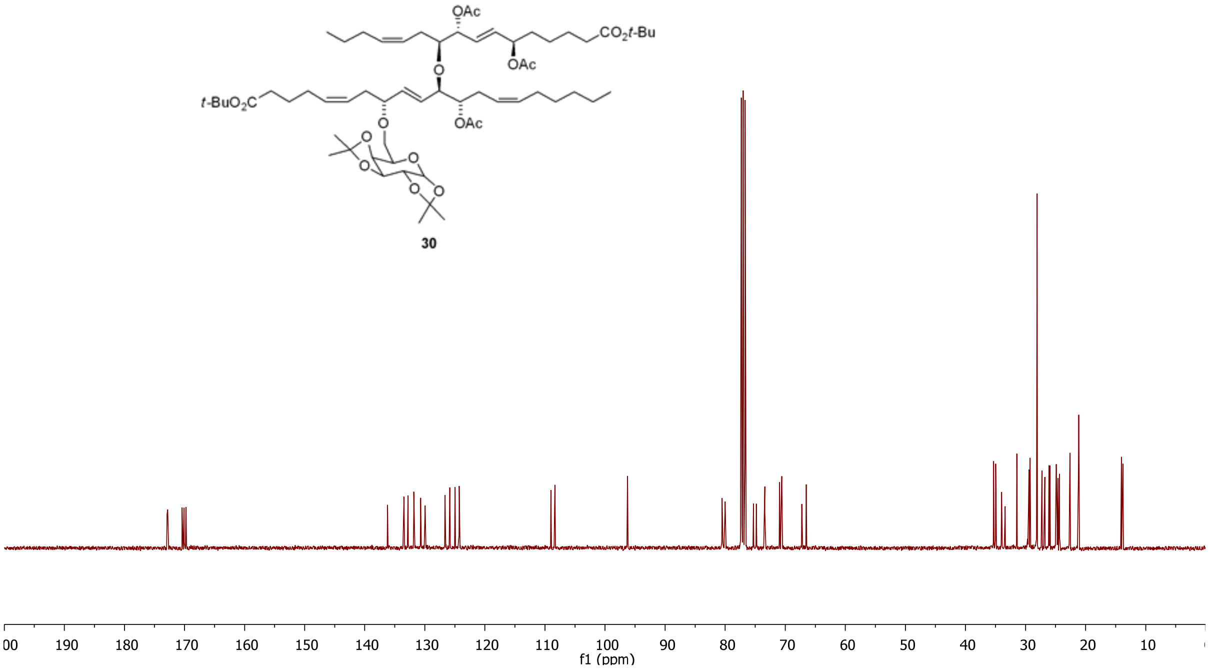

30
f1 (ppm)

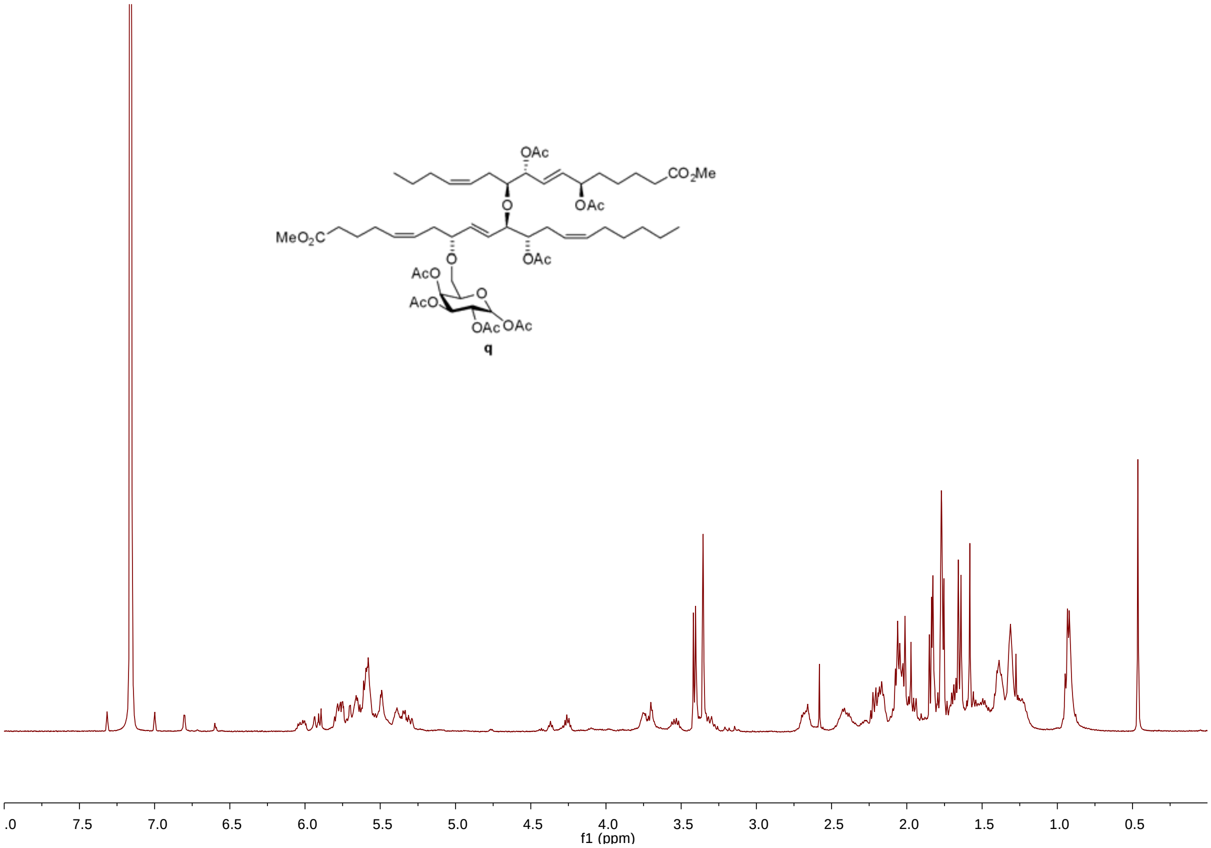
OAc
CO2Me
OAc
O
MeO2C
OAc
O
AcO
AcO
O
OAc
OAc
q
.0
7.5
7.0
6.5
6.0
5.5
5.0
4.5
4.0
3.5
3.0
2.5
2.0
1.5
1.0
0.5
f1 (ppm)

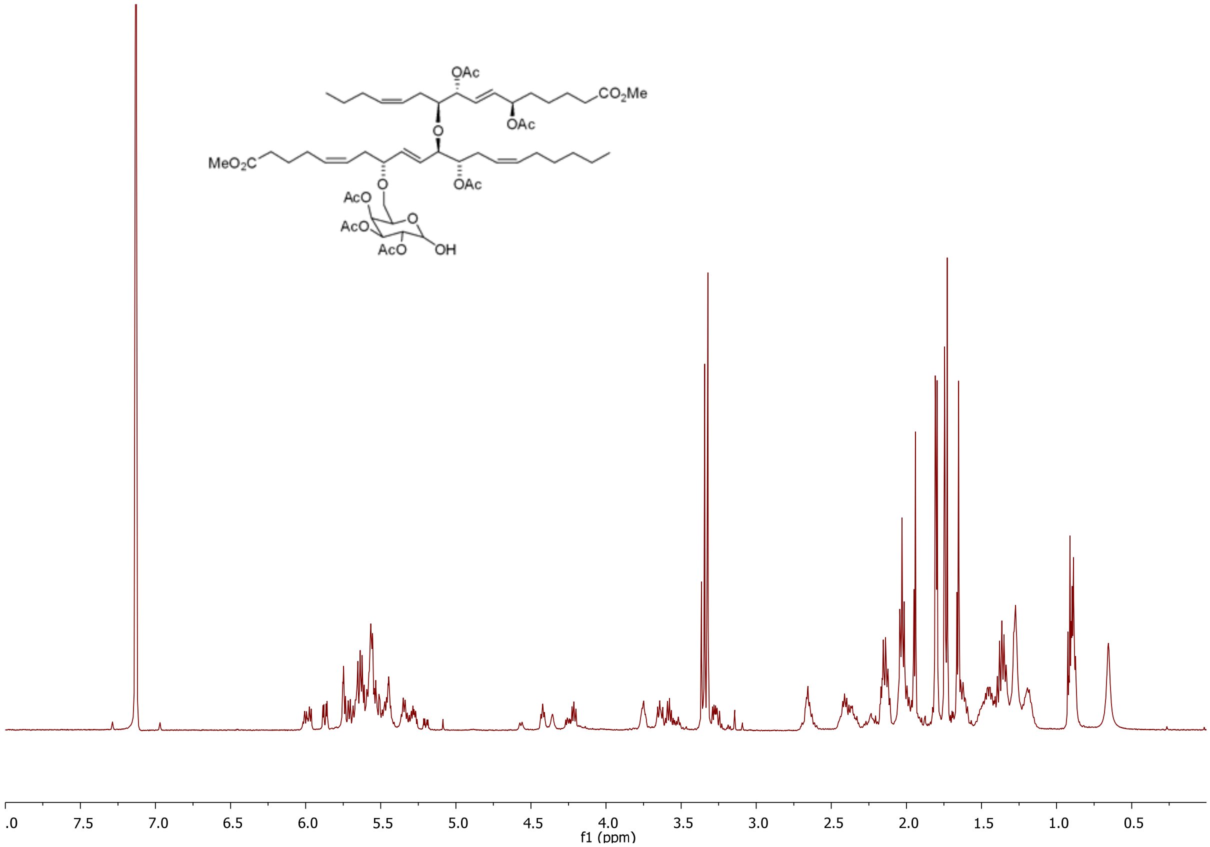
OAc
CO2Me
OAc
O
MeO2C
OAc
O
AcO
AcO
O
AcO
OH
.0
7.5
7.0
6.5
6.0
5.5
5.0
4.5
4.0
3.5
3.0
2.5
2.0
1.5
1.0
0.5
f1 (ppm)

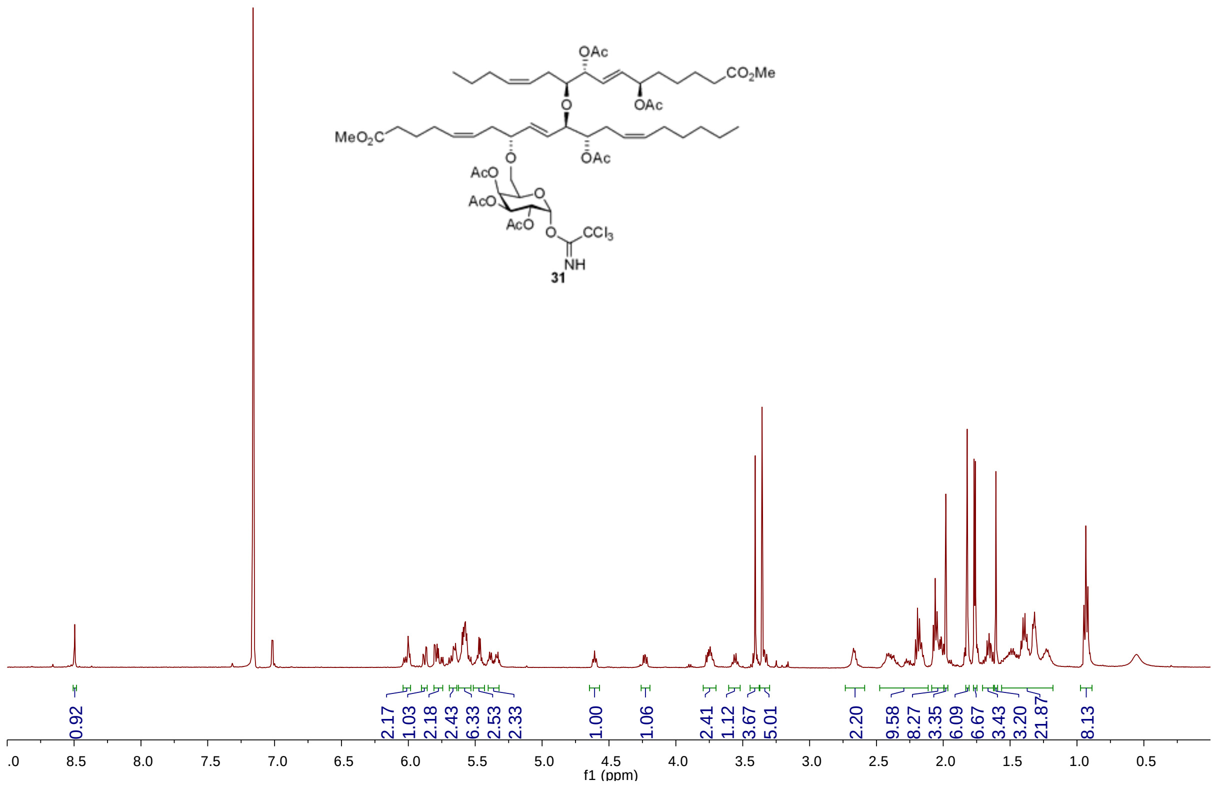
OAc
CO2Me
OAc
MeO2C
OAc
AcO
AcO
AcO
CCl3
NH
31
0.92
2.17 1.03 2.18 2.43 6.33 2.53 2.33
1.00
1.06
2.41 1.12 3.67 5.01
2.20
9.58 8.27 3.35 6.09 6.67 3.43 3.20 21.87
8.13
f1 (ppm)

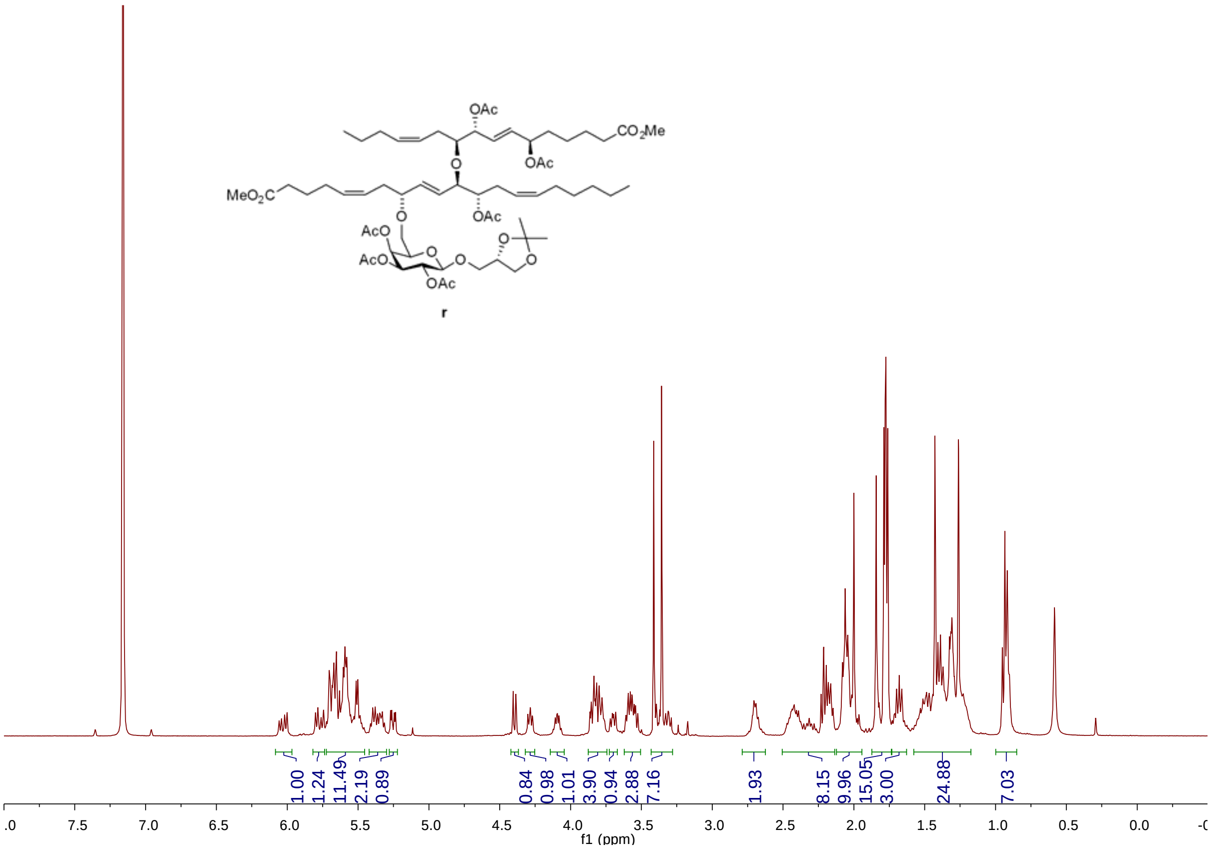
OAc
CO2Me
OAc
MeO2C
OAc
AcO
AcO
OAc
r
1.00 1.24 11.49 2.19 0.89
0.84 0.98 1.01 3.90 0.94 2.88 7.16
1.93
8.15 9.96 15.05 3.00
24.88
7.03
f1 (ppm)

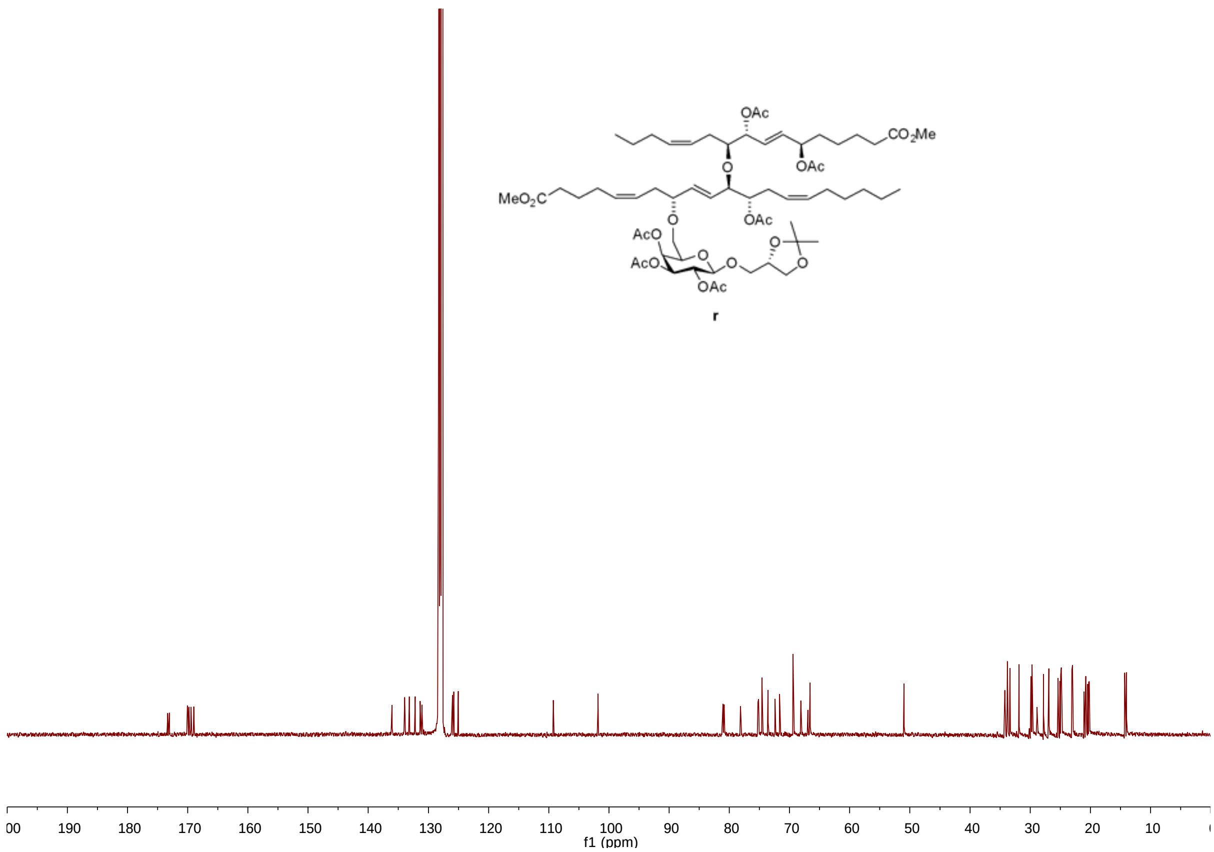
OAc
CO2Me
OAc
O
MeO2C
O
OAc
AcO
O
AcO
O
O
O
O
OAc
00
190
180
170
160
150
140
130
120
110
100
90
80
70
60
50
40
30
20
10
f1 (ppm)

r

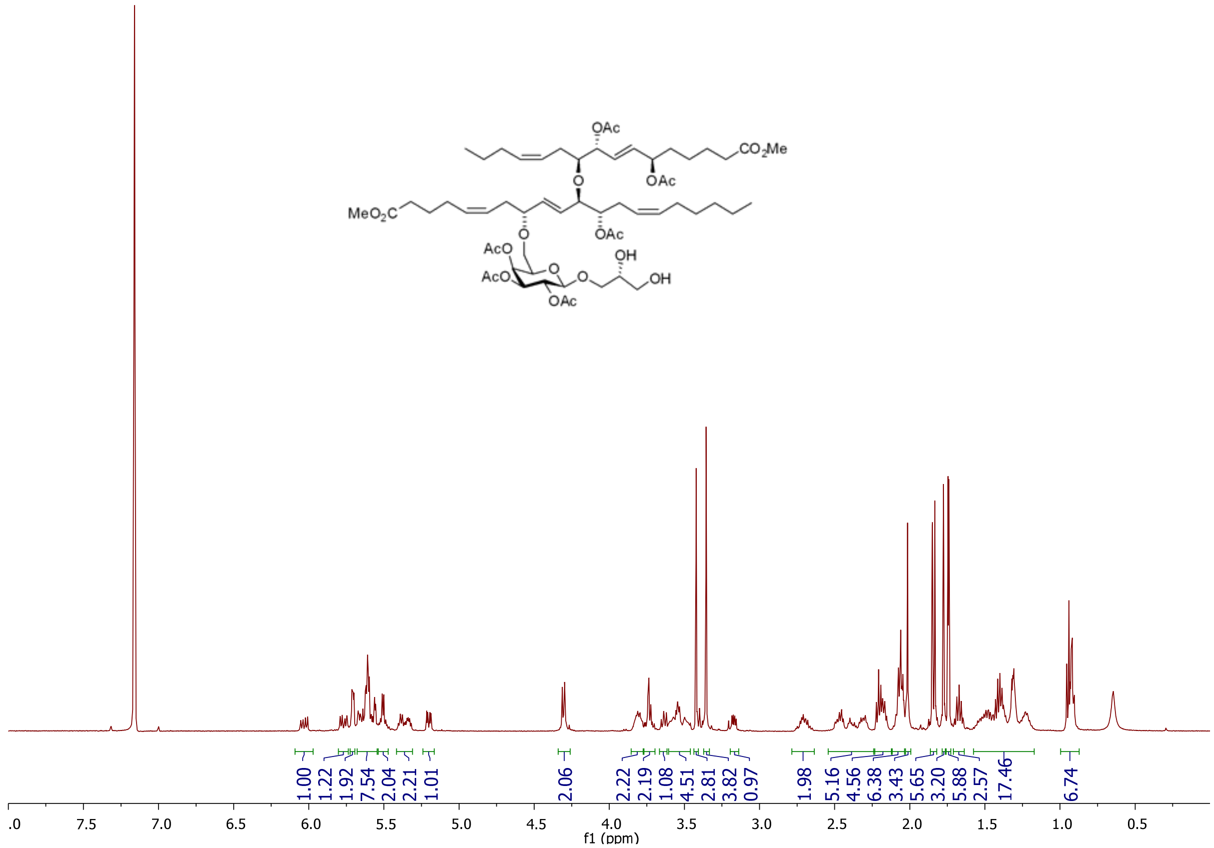
OAc
CO2Me
OAc
O
MeO2C
O
OAc
AcO
OH
O
AcO
O
O
OH
OAc
1.00
1.22
1.92
7.54
2.04
2.21
1.01
2.06
2.22
2.19
1.08
4.51
2.81
3.82
0.97
1.98
5.16
4.56
6.38
3.43
5.65
3.20
5.88
2.57
17.46
6.74
.0
7.5
7.0
6.5
6.0
5.5
5.0
4.5
4.0
3.5
3.0
2.5
2.0
1.5
1.0
0.5
f1 (ppm)

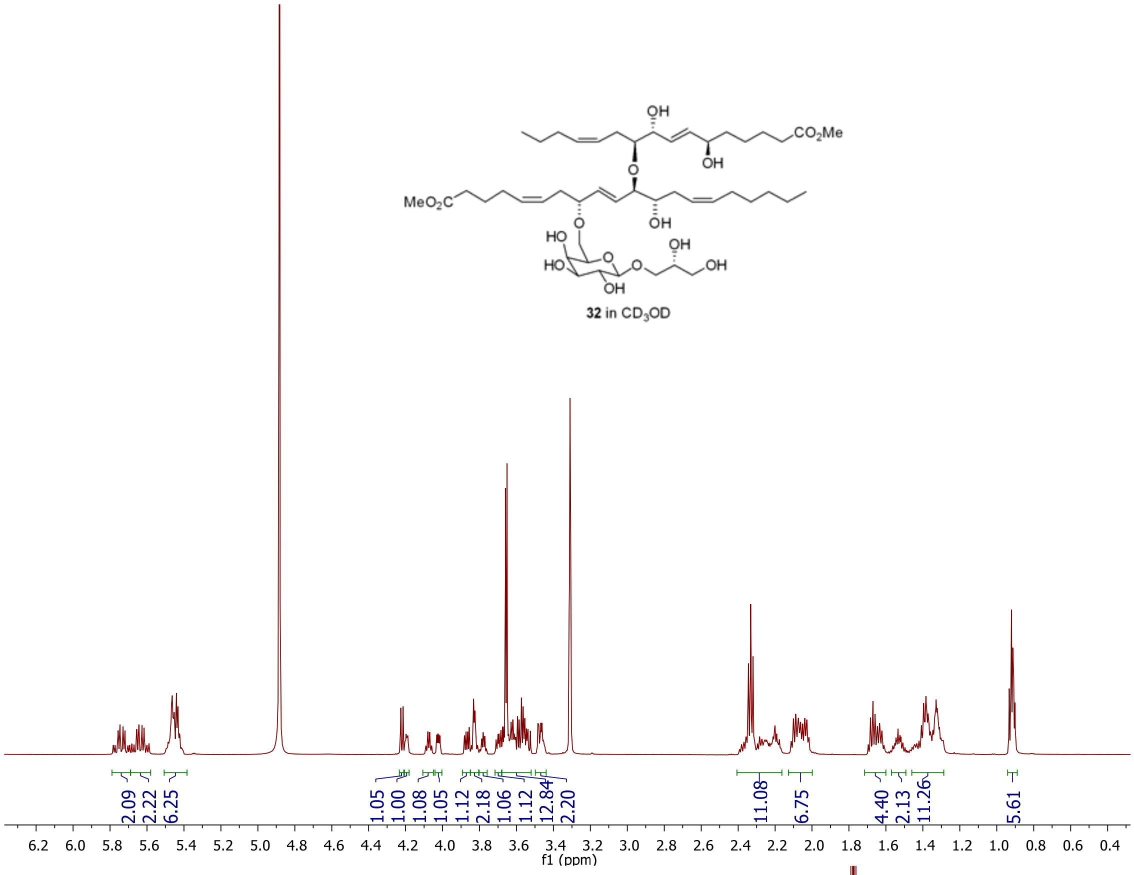

OH
CO2Me
OH
O
MeO2C
OH
HO
OH
HO
O
O
OH
OH
32 in CD3OD
2.09
2.22
6.25
1.05
1.00
1.08
1.05
1.12
2.18
1.06
1.12
12.84
2.20
11.08
6.75
4.40
2.13
11.26
5.61
f1 (ppm)

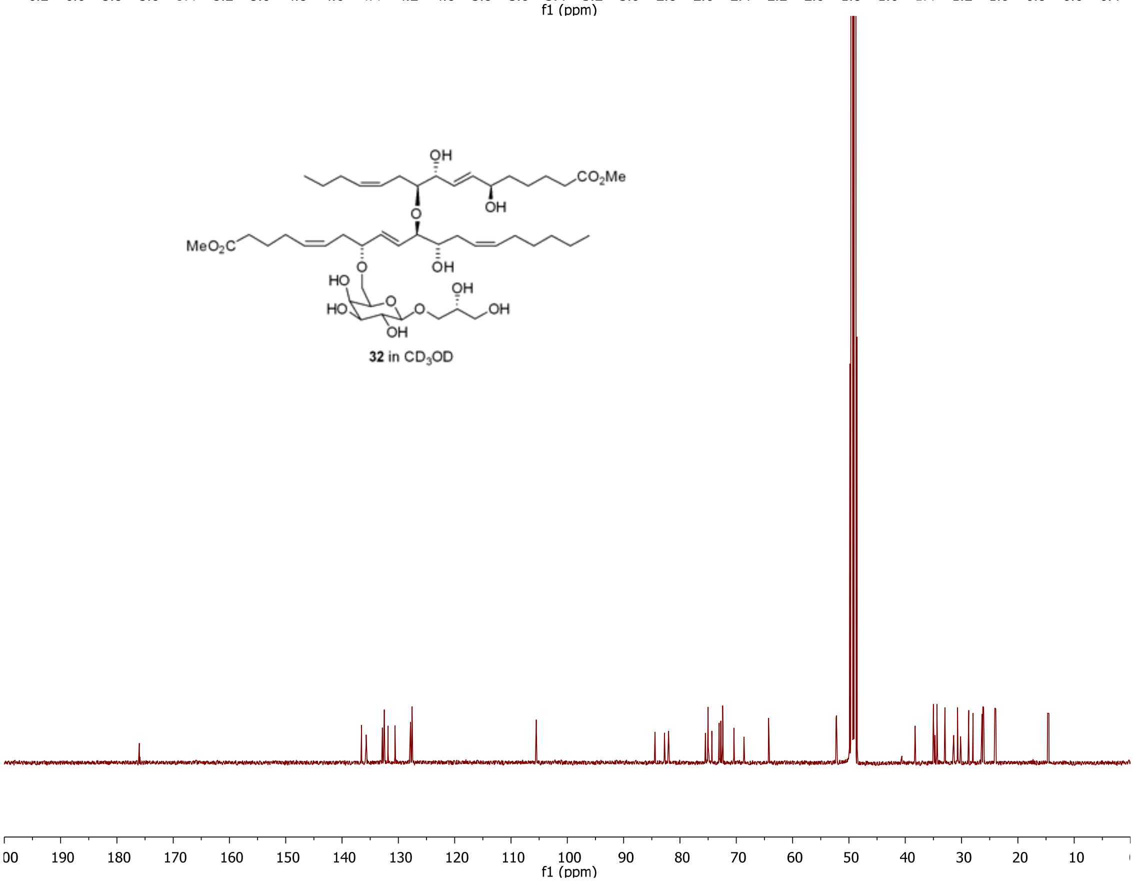

OH
CO2Me
OH
O
MeO2C
OH
HO
OH
HO
O
O
OH
OH
32 in CD3OD
f1 (ppm)

gradCOSY90 NMR Spectrum of compound **32** at 600 MHz in CD_3OD

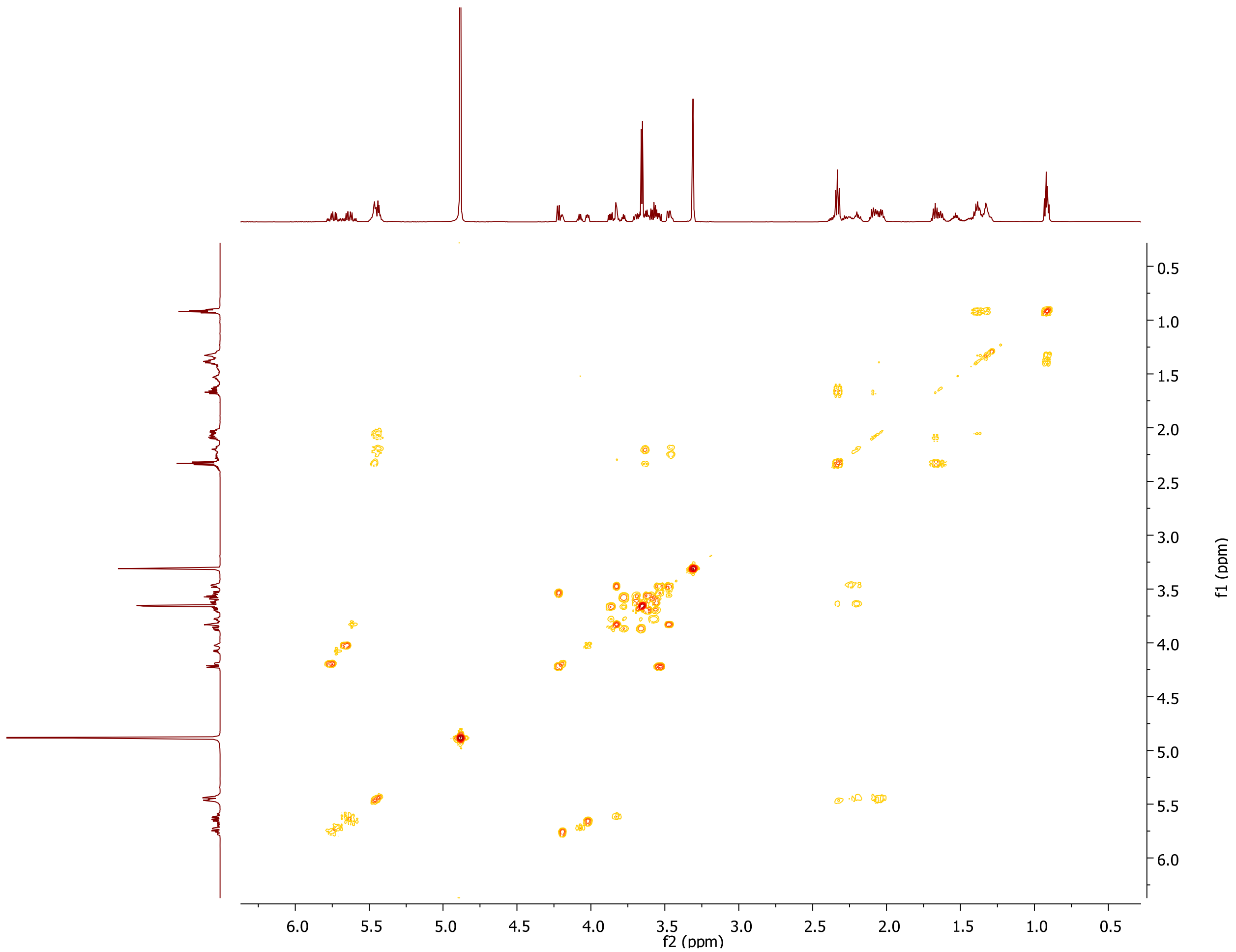

gradHSQC NMR Spectrum of compound **32** at 600 MHz in CD_3OD

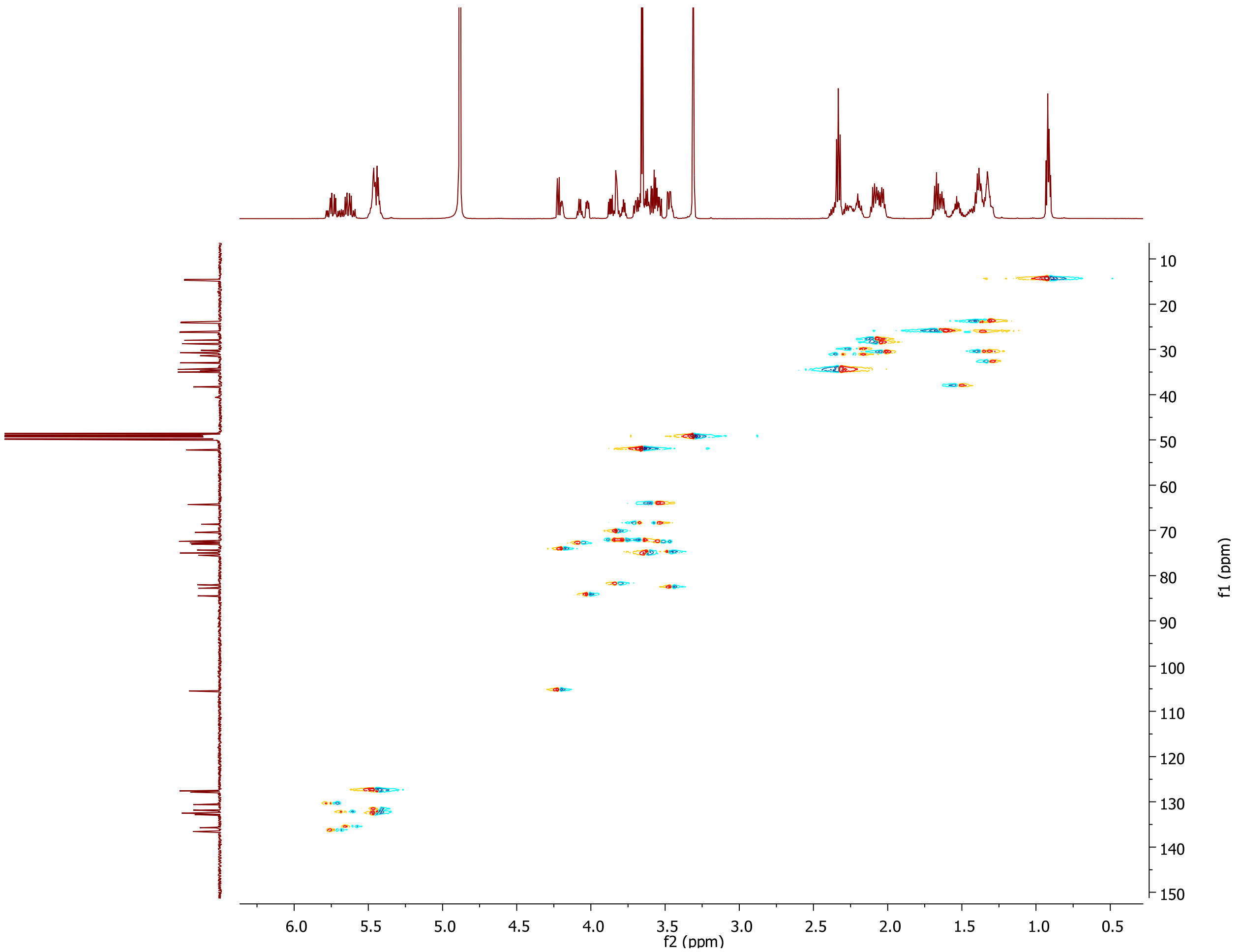

gradHMBC NMR Spectrum of compound **32** at 600 MHz in CD_3OD

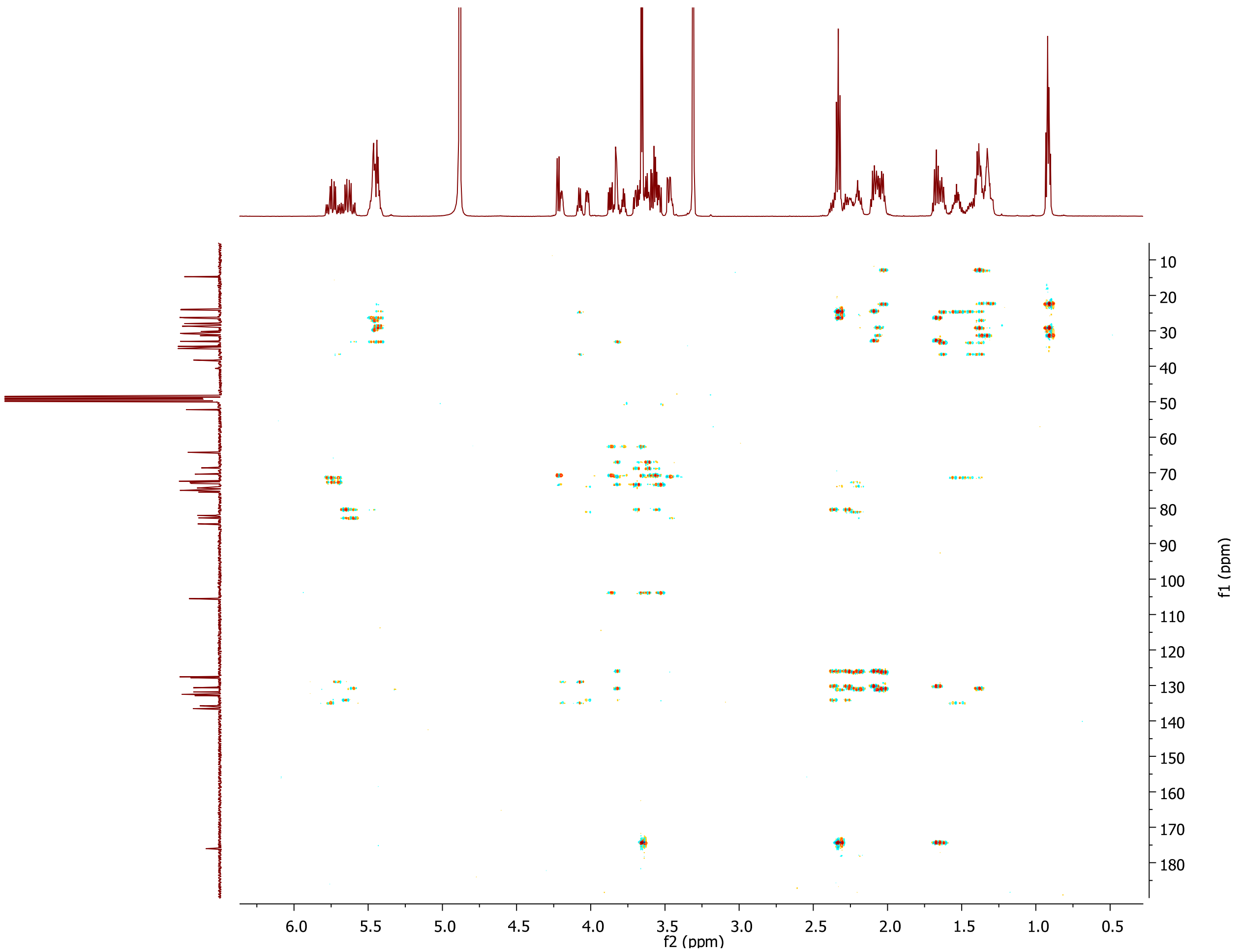

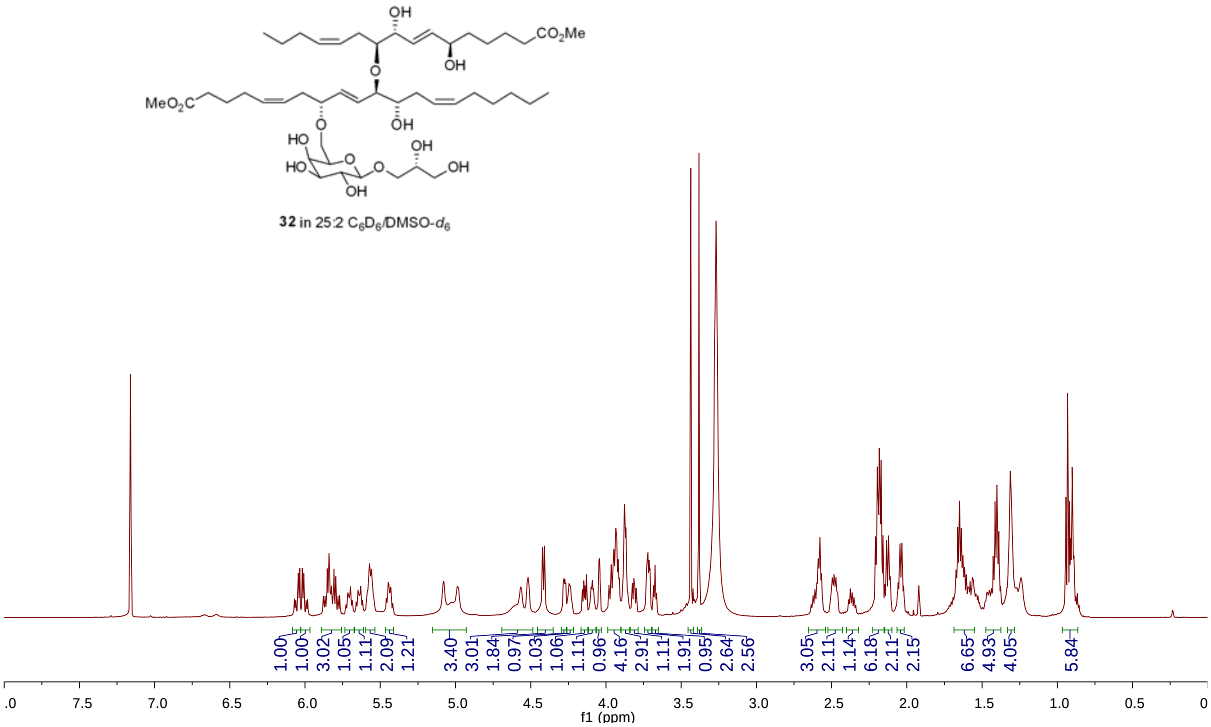

OH
CO_2Me
OH
O
MeO_2C
OH
HO
O
HO
O
O
OH
OH
OH
32 in 25:2 C_6D_6/DMSO-d_6
1.00
1.00
3.02
1.05
1.11
2.09
1.21
3.40
3.01
1.84
0.97
1.03
1.06
1.11
0.96
4.16
2.91
1.11
1.91
0.95
2.64
2.56
3.05
2.11
1.14
6.18
2.11
2.15
6.65
4.93
4.05
5.84
7.5
7.0
6.5
6.0
5.5
5.0
4.5
4.0
3.5
3.0
2.5
2.0
1.5
1.0
0.5
f1 (ppm)

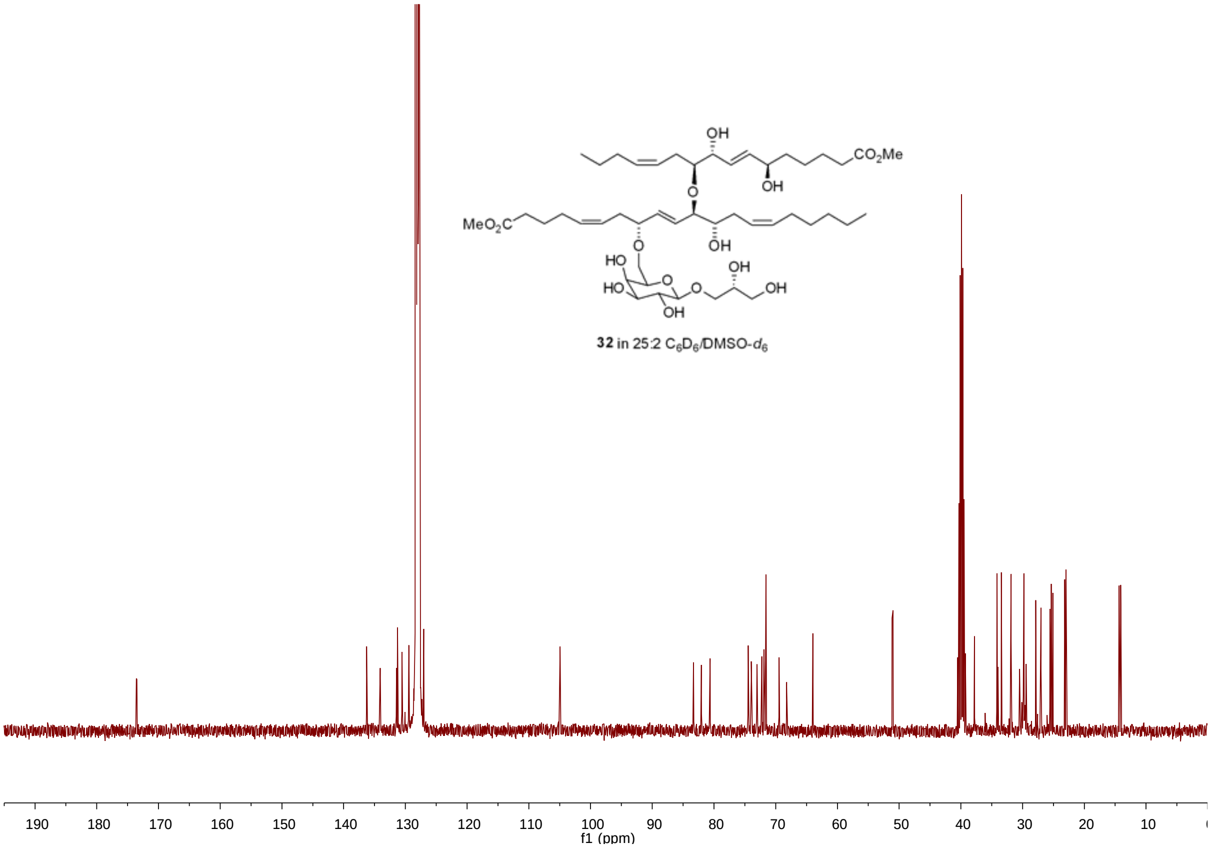

OH
CO_2Me
OH
O
MeO_2C
OH
HO
O
HO
O
O
OH
OH
OH
32 in 25:2 C_6D_6/DMSO-d_6
190
180
170
160
150
140
130
120
110
100
90
80
70
60
50
40
30
20
10
f1 (ppm)

gradCOSY90 NMR Spectrum of compound **32** at 600 MHz in 25:2 C_6D_6/DMSO-d_6

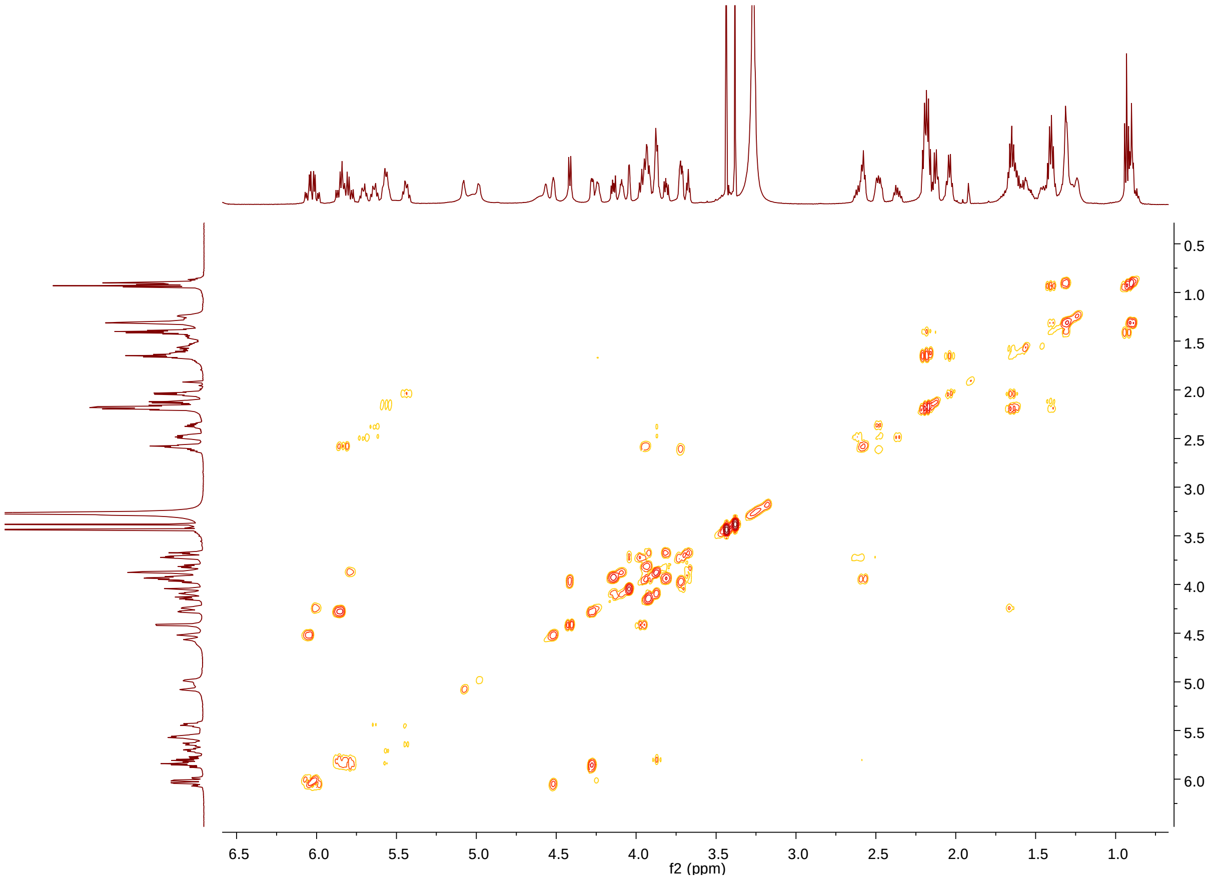

gradHSQC NMR Spectrum of compound **32** at 600 MHz in 25:2 C_6D_6/DMSO-d_6

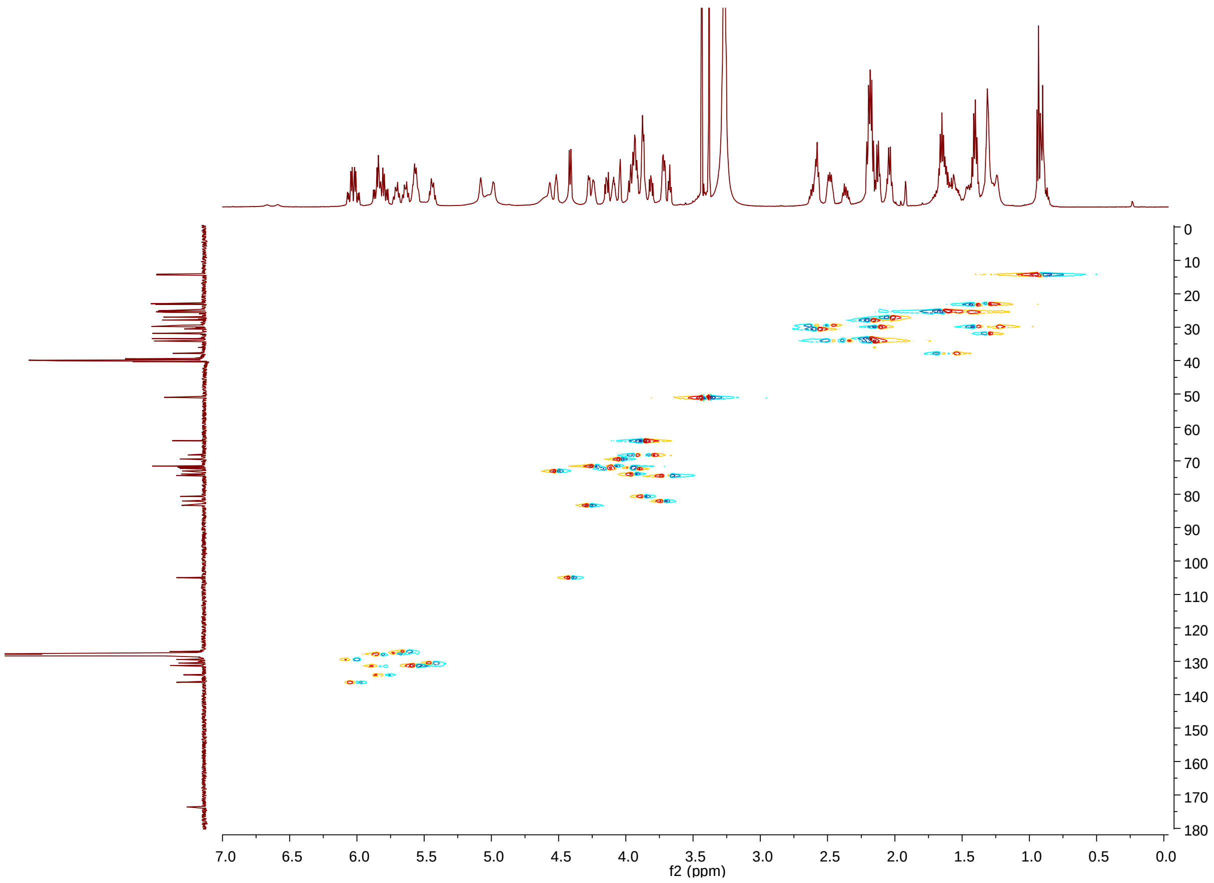

gradHMBC NMR Spectrum of compound **32** at 600 MHz in 25:2 C_6D_6/DMSO-d_6

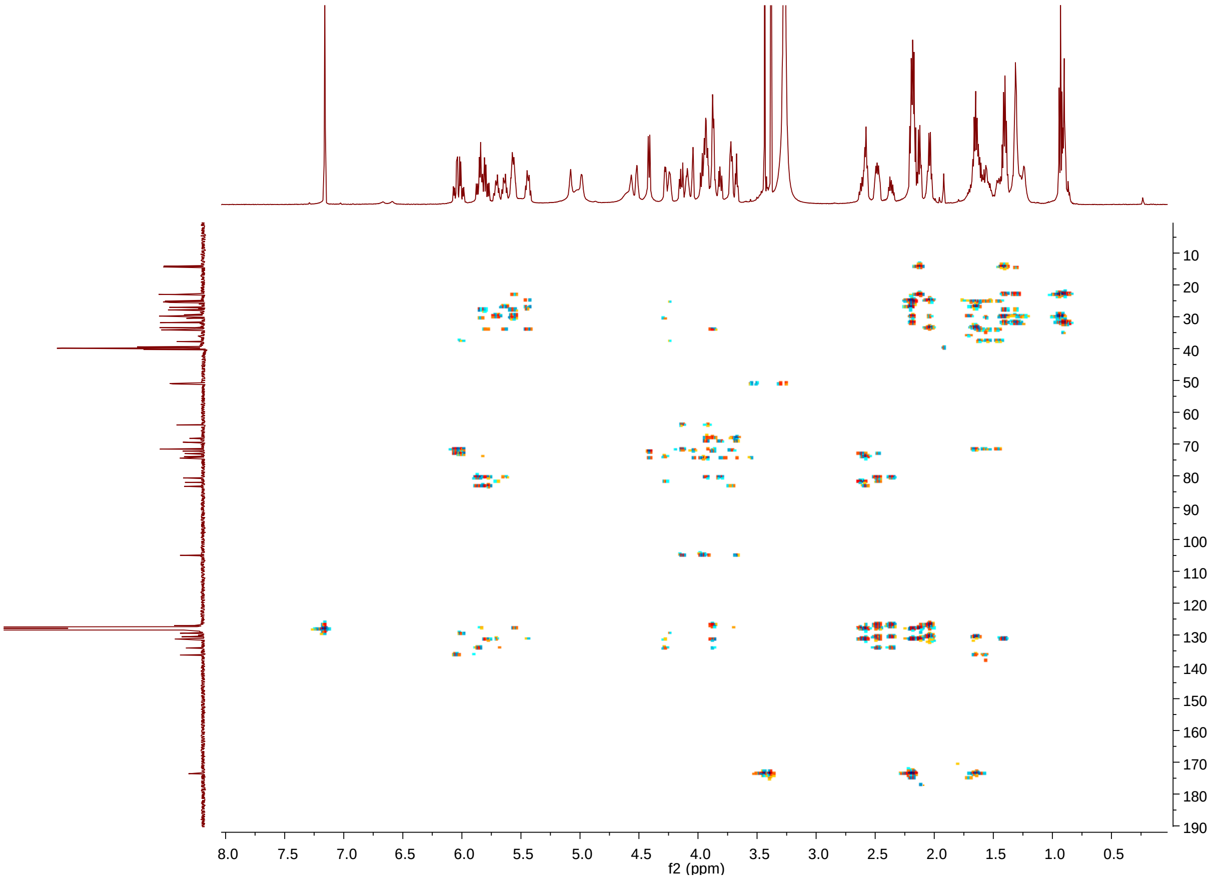

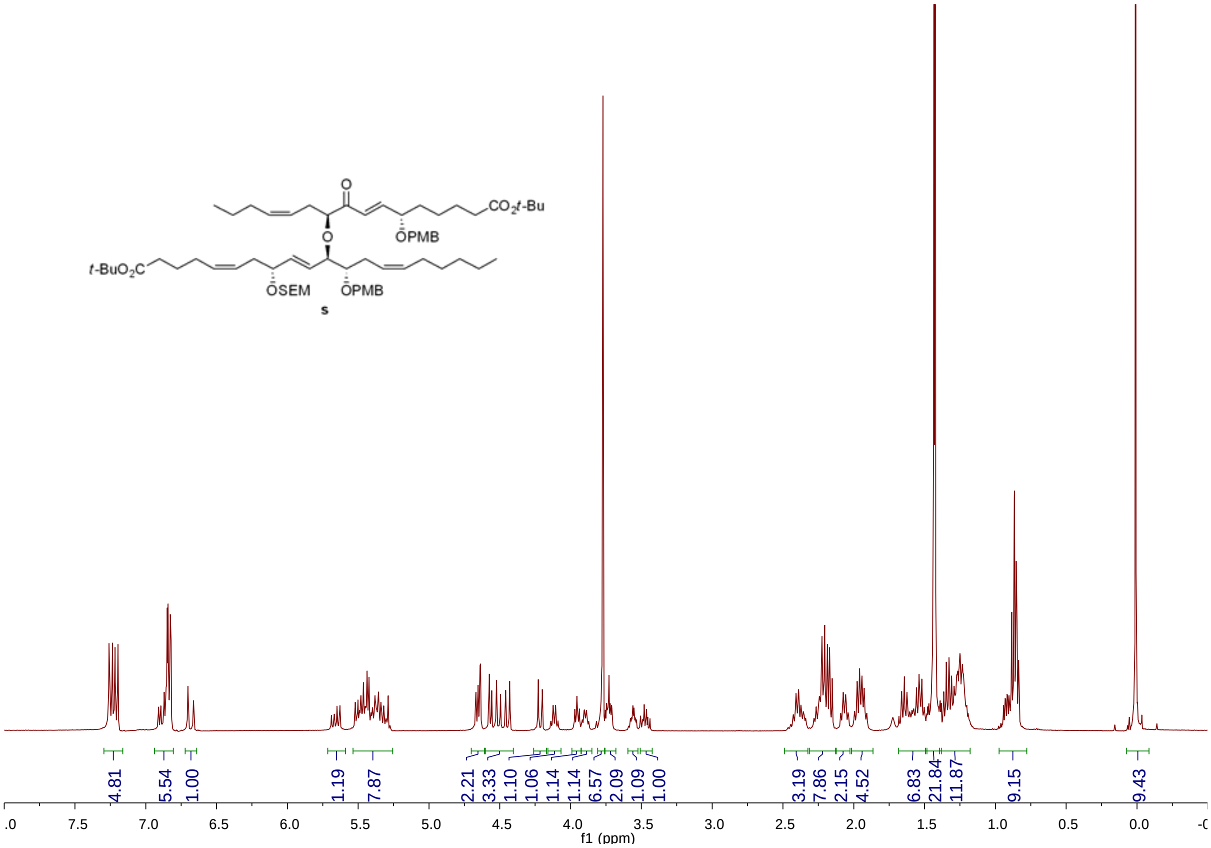
CO2t-Bu
OPMB
t-BuO2C
OSEM
OPMB
s
4.81
5.54
1.00
1.19
7.87
2.21
3.33
1.10
1.06
1.14
1.14
6.57
2.09
1.09
1.00
3.19
7.86
2.15
4.52
6.83
21.84
11.87
9.15
9.43
f1 (ppm)

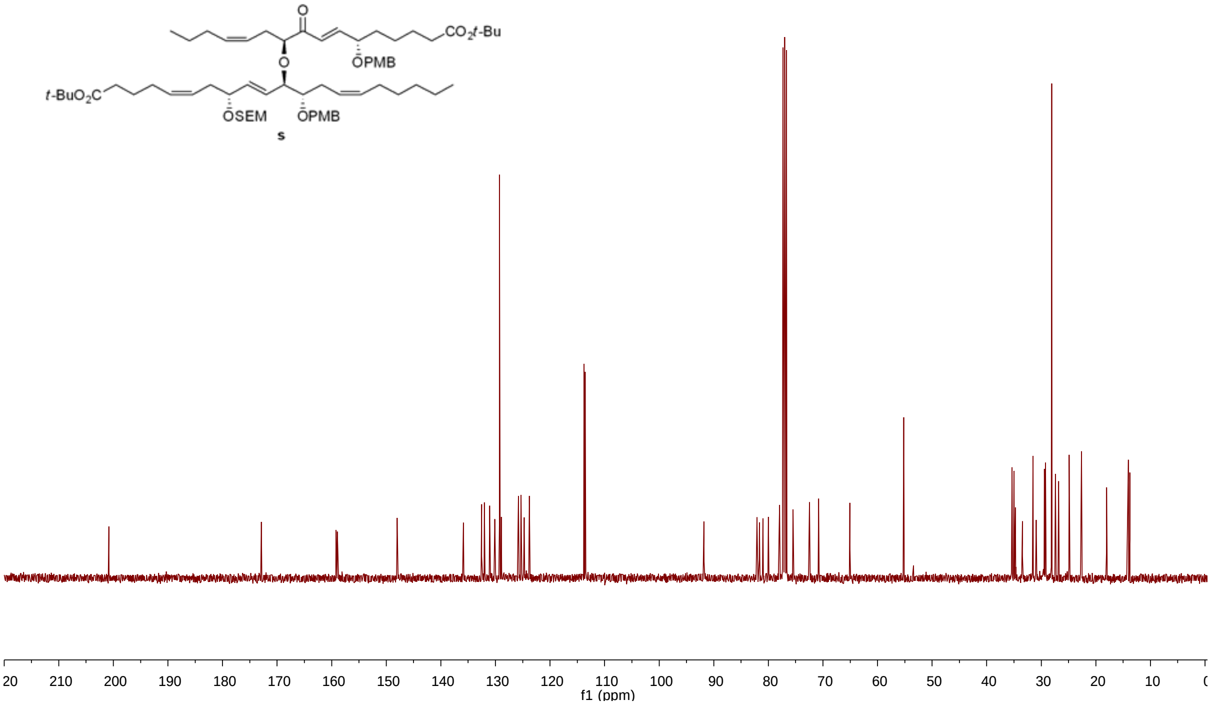
CO2t-Bu
OPMB
t-BuO2C
OSEM
OPMB
s
f1 (ppm)

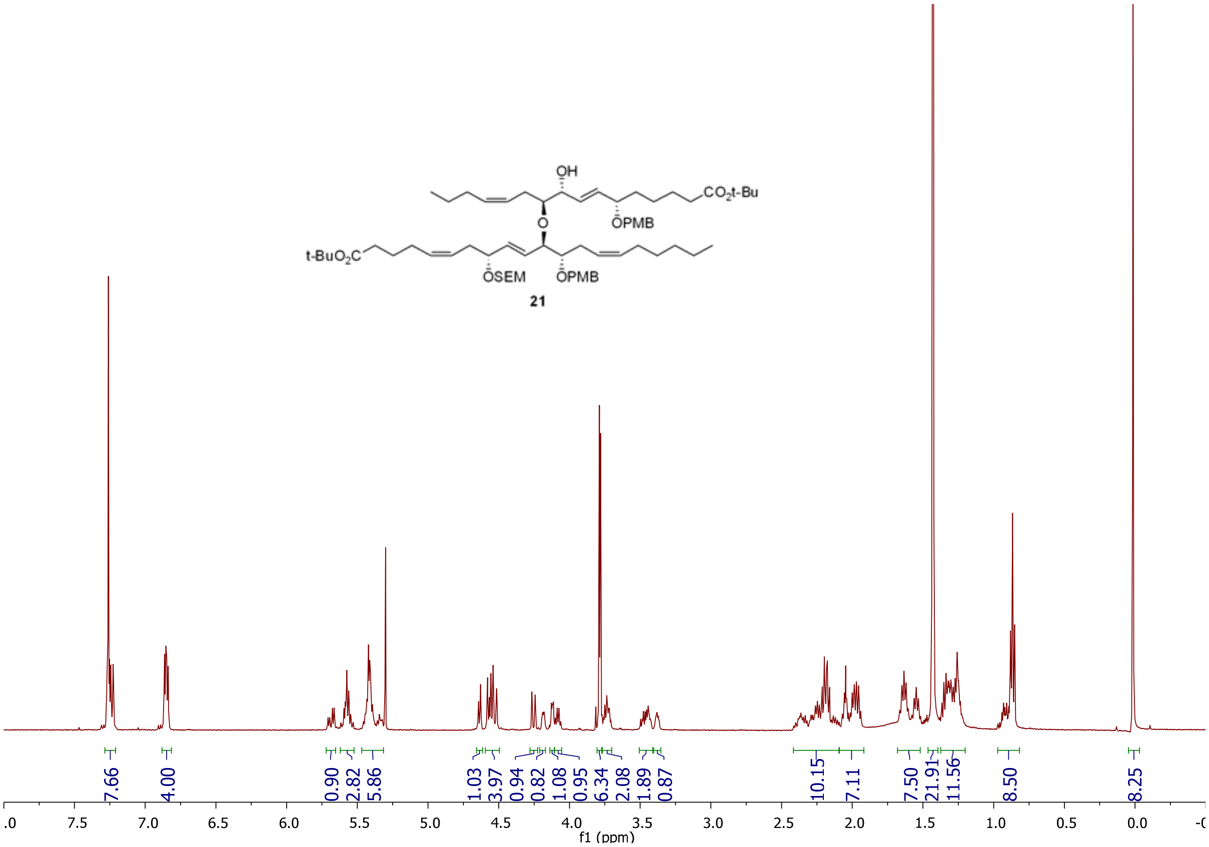
OH
CO2t-Bu
O
OPMB
t-BuO2C
OSEM
OPMB
21
7.66
4.00
0.90
2.82
5.86
1.03
3.97
0.94
0.82
1.08
0.95
6.34
2.08
1.89
0.87
10.15
7.11
7.50
21.91
11.56
8.50
8.25
f1 (ppm)

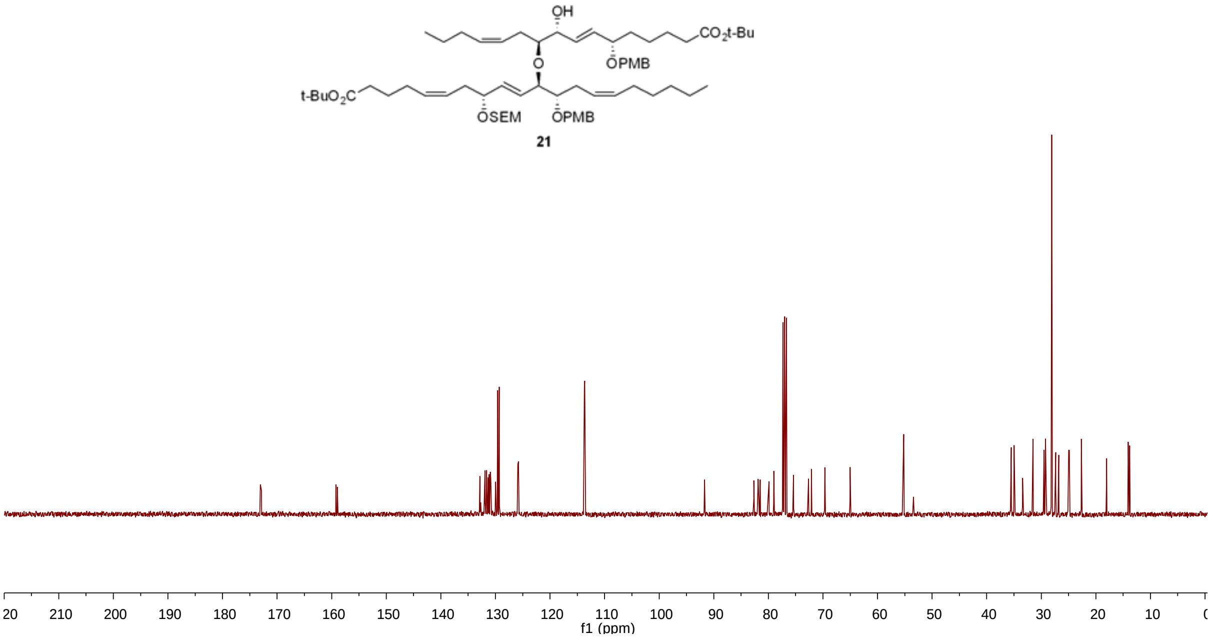
OH
CO2t-Bu
O
OPMB
t-BuO2C
OSEM
OPMB
21
f1 (ppm)

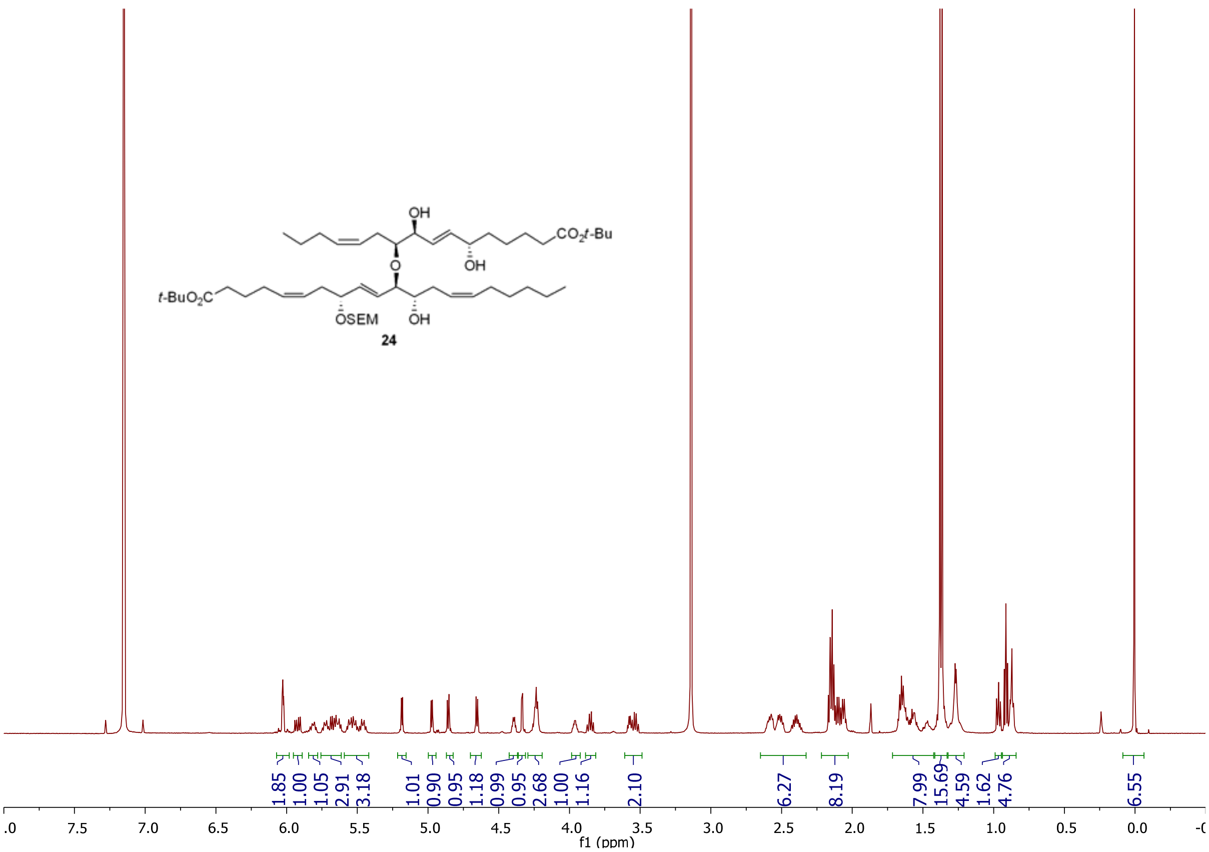

OH
CO2t-Bu
OH
O
t-BuO2C
OSEM
OH
24
f1 (ppm)

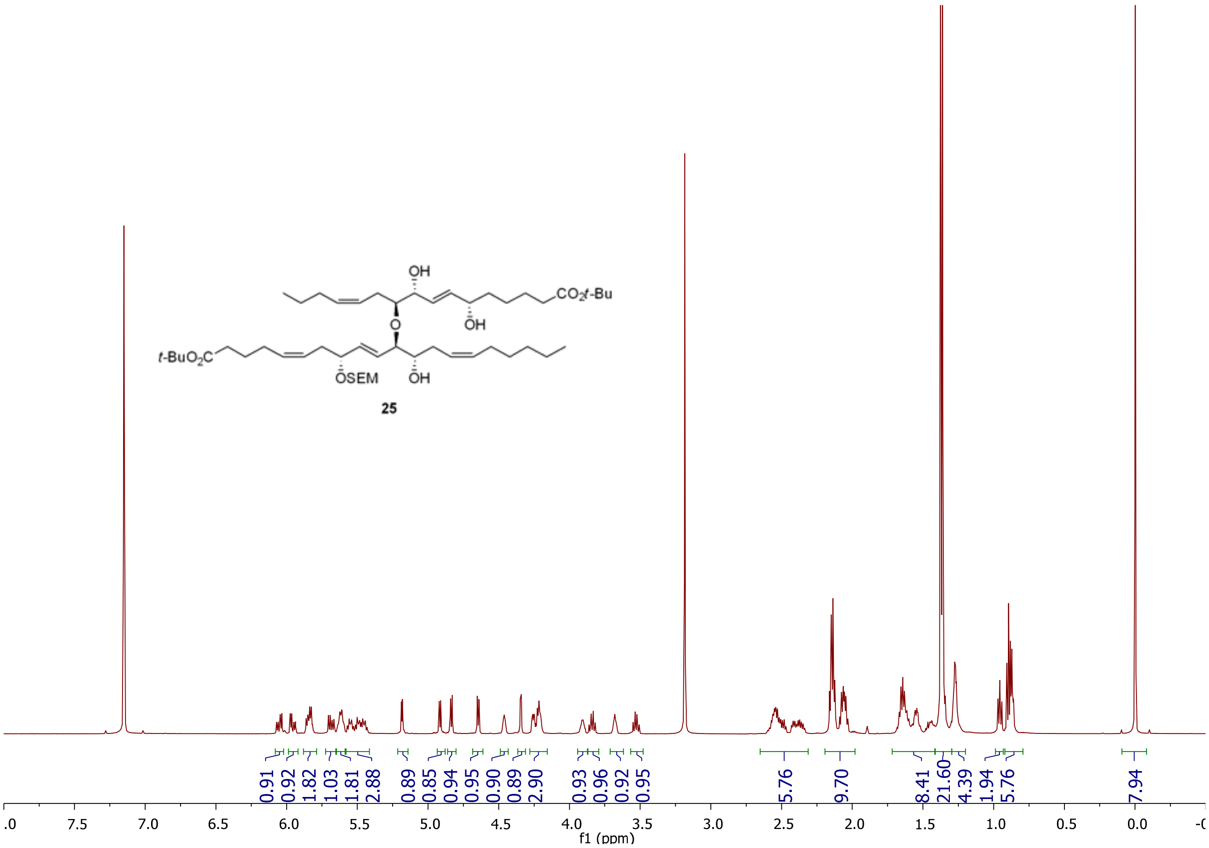

OH
CO2t-Bu
OH
O
t-BuO2C
OSEM
OH
25
f1 (ppm)

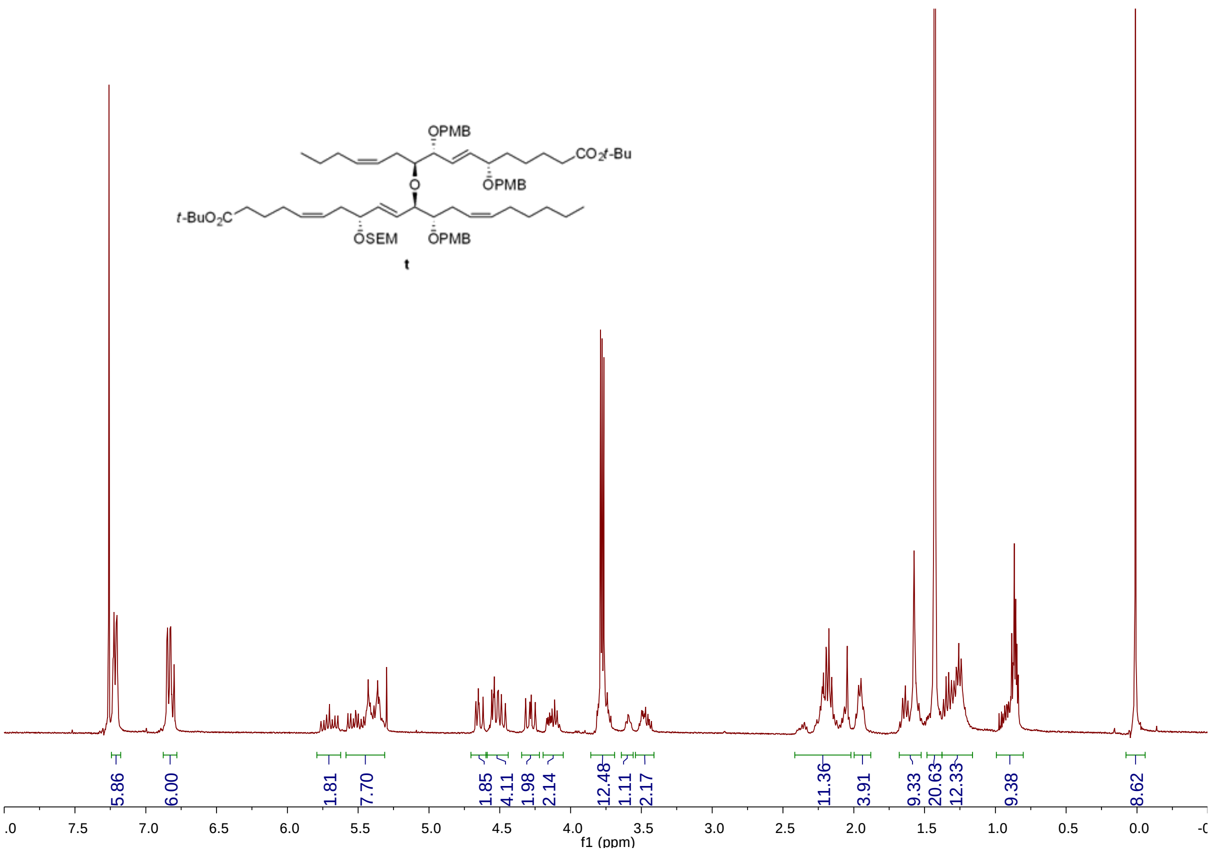
OPMB
CO2t-Bu
OPMB
O
t-BuO2C
OSEM
OPMB
t
5.86
6.00
1.81
7.70
1.85
4.11
1.98
2.14
12.48
1.11
2.17
11.36
3.91
9.33
20.63
12.33
9.38
8.62
f1 (ppm)

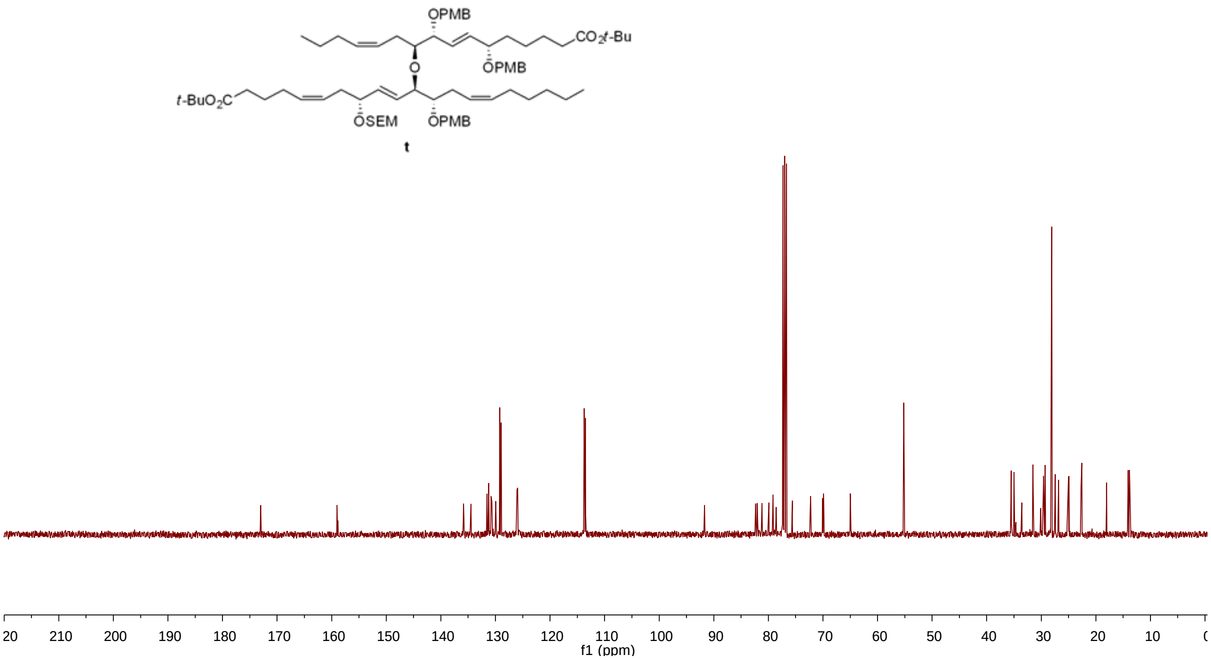
OPMB
CO2t-Bu
OPMB
O
t-BuO2C
OSEM
OPMB
t
f1 (ppm)

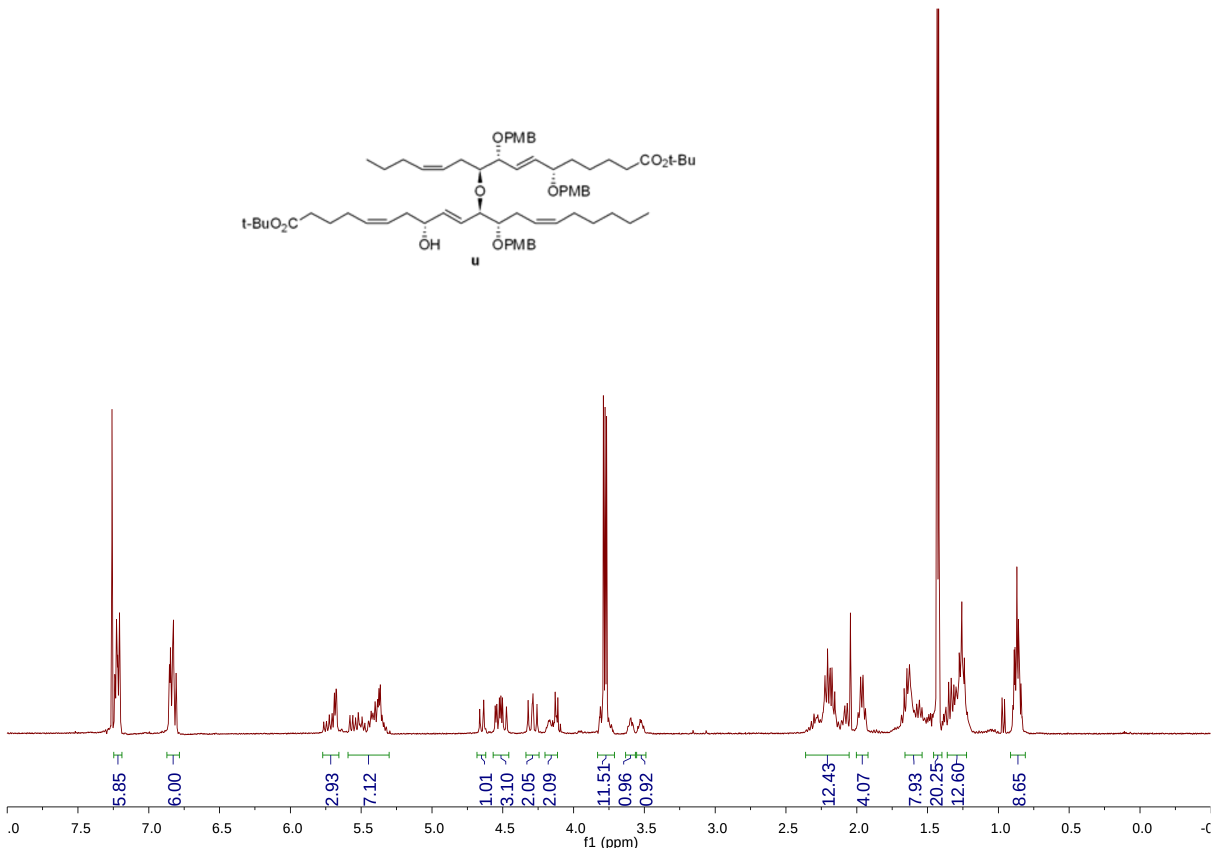
OPMB
CO2t-Bu
O
OPMB
t-BuO2C
OH
OPMB
u
5.85
6.00
2.93
7.12
1.01
3.10
2.05
2.09
11.51
0.96
0.92
12.43
4.07
7.93
20.25
12.60
8.65
f1 (ppm)

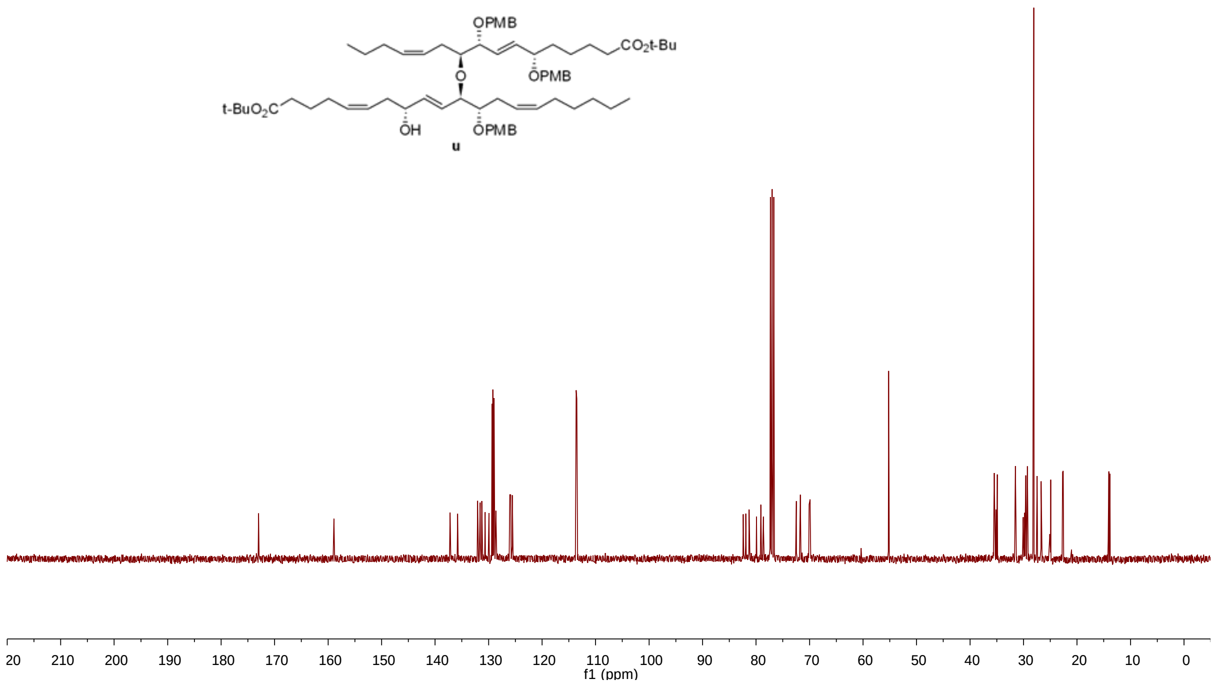
OPMB
CO2t-Bu
O
OPMB
t-BuO2C
OH
OPMB
u
f1 (ppm)

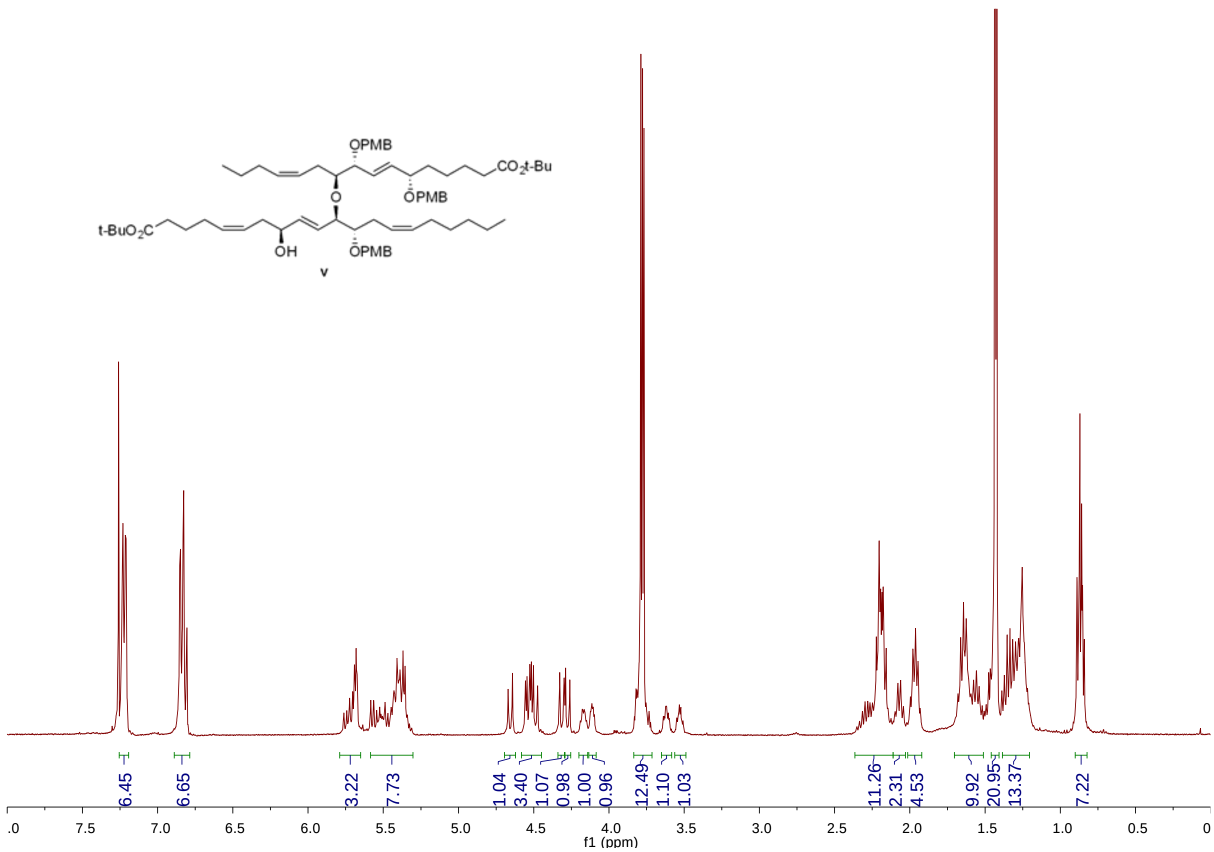
OPMB
CO2t-Bu
O
OPMB
t-BuO2C
OH
OPMB
v
6.45
6.65
3.22
7.73
1.04
3.40
1.07
0.98
1.00
0.96
12.49
1.10
1.03
11.26
2.31
4.53
9.92
20.95
13.37
7.22
f1 (ppm)

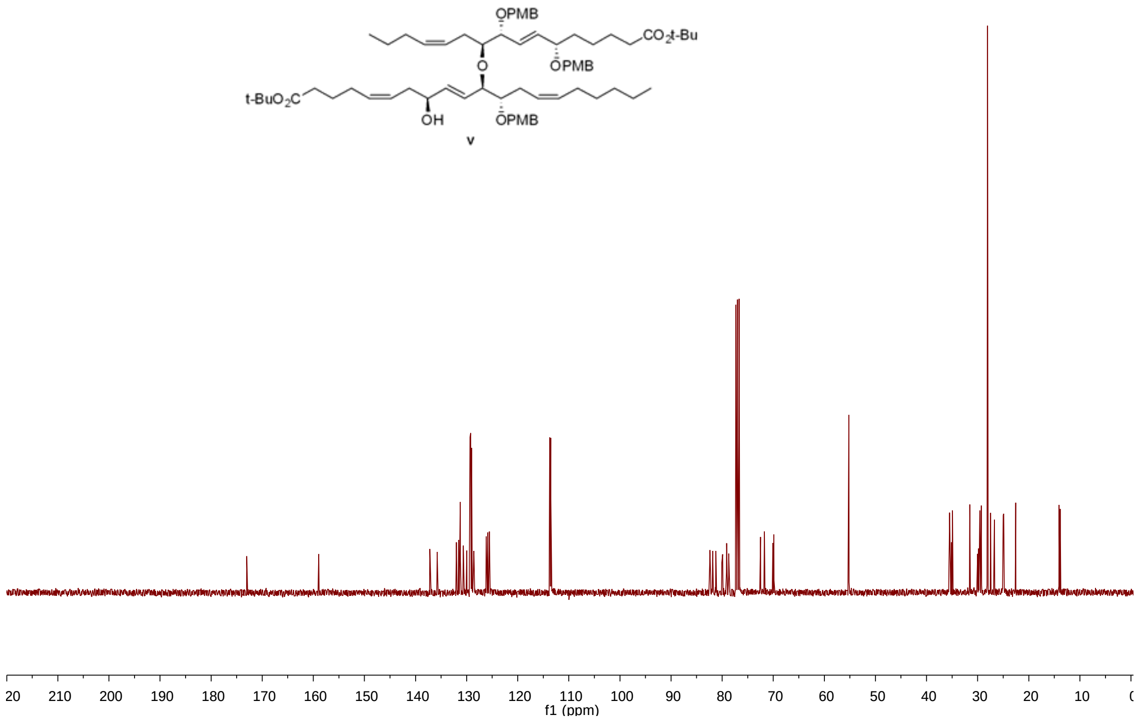
OPMB
CO2t-Bu
O
OPMB
t-BuO2C
OH
OPMB
v
f1 (ppm)

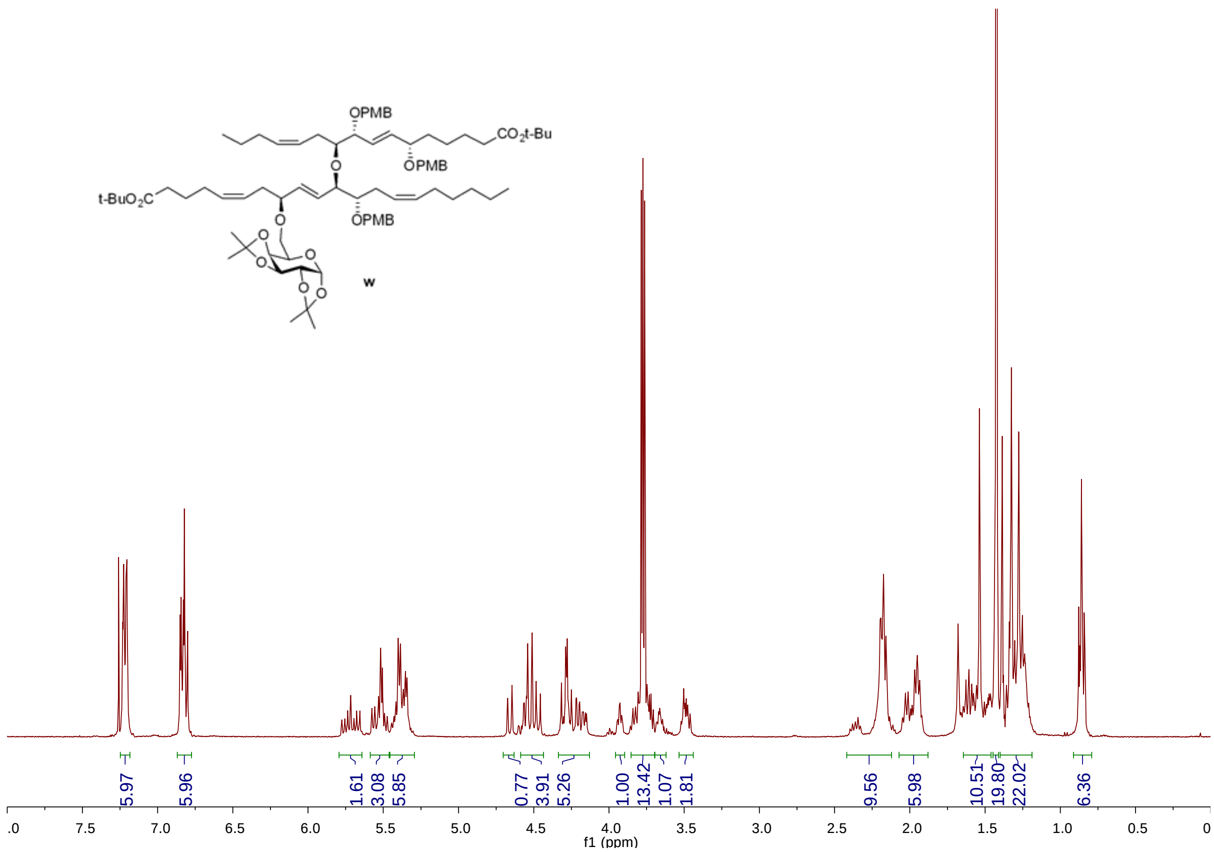
OPMB
CO2t-Bu
OPMB
t-BuO2C
OPMB
w
5.97
5.96
1.61
3.08
5.85
0.77
3.91
5.26
1.00
13.42
1.07
1.81
9.56
5.98
10.51
19.80
22.02
6.36
f1 (ppm)

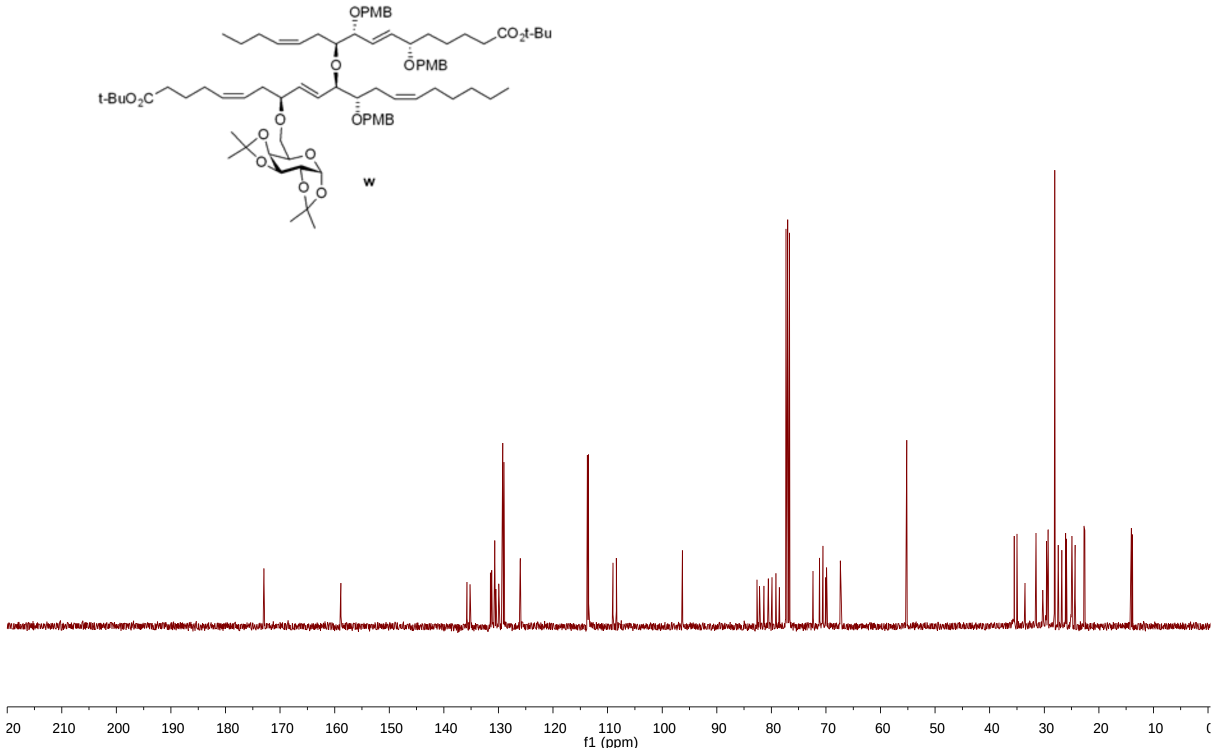
OPMB
CO2t-Bu
OPMB
t-BuO2C
OPMB
w
f1 (ppm)

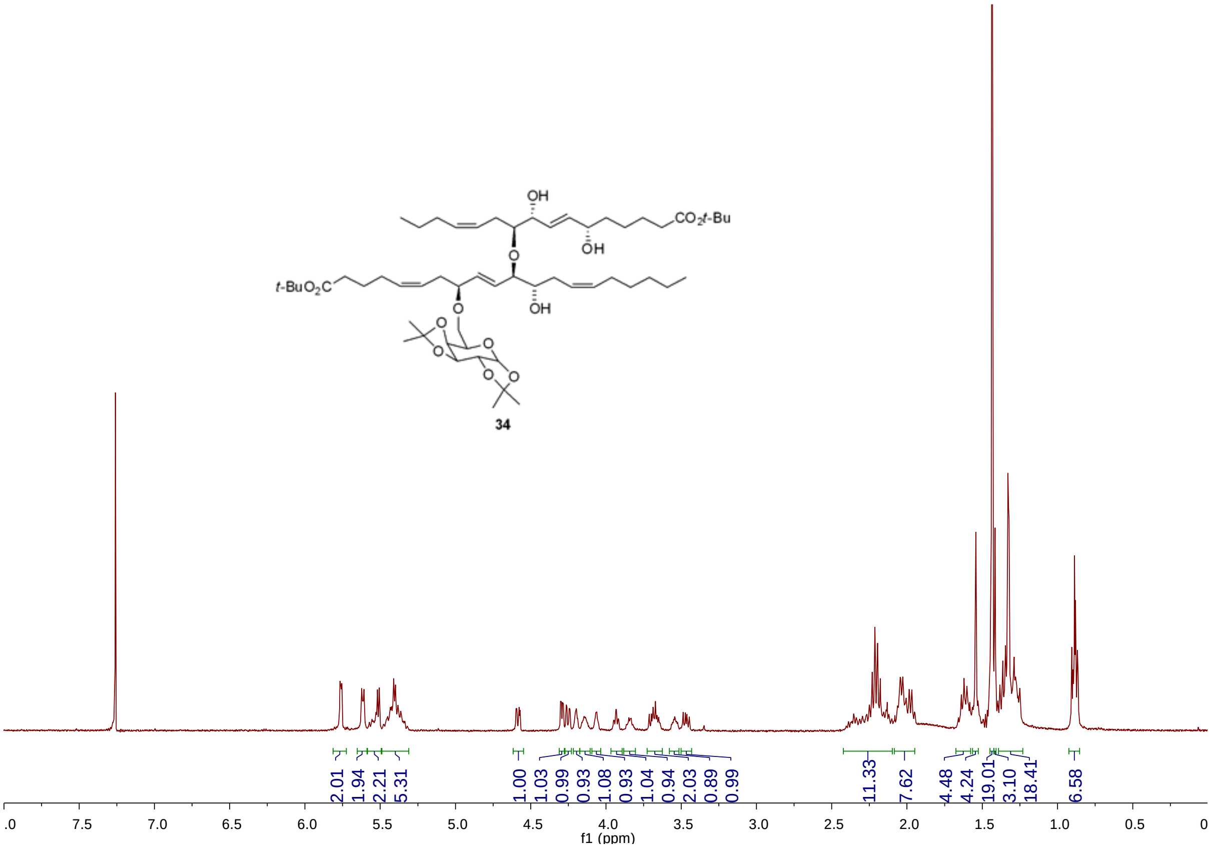
34
2.01
1.94
2.21
5.31
1.00
1.03
0.99
0.93
1.08
0.93
1.04
0.94
2.03
0.89
0.99
11.33
7.62
4.48
4.24
19.01
3.10
18.41
6.58
f1 (ppm)

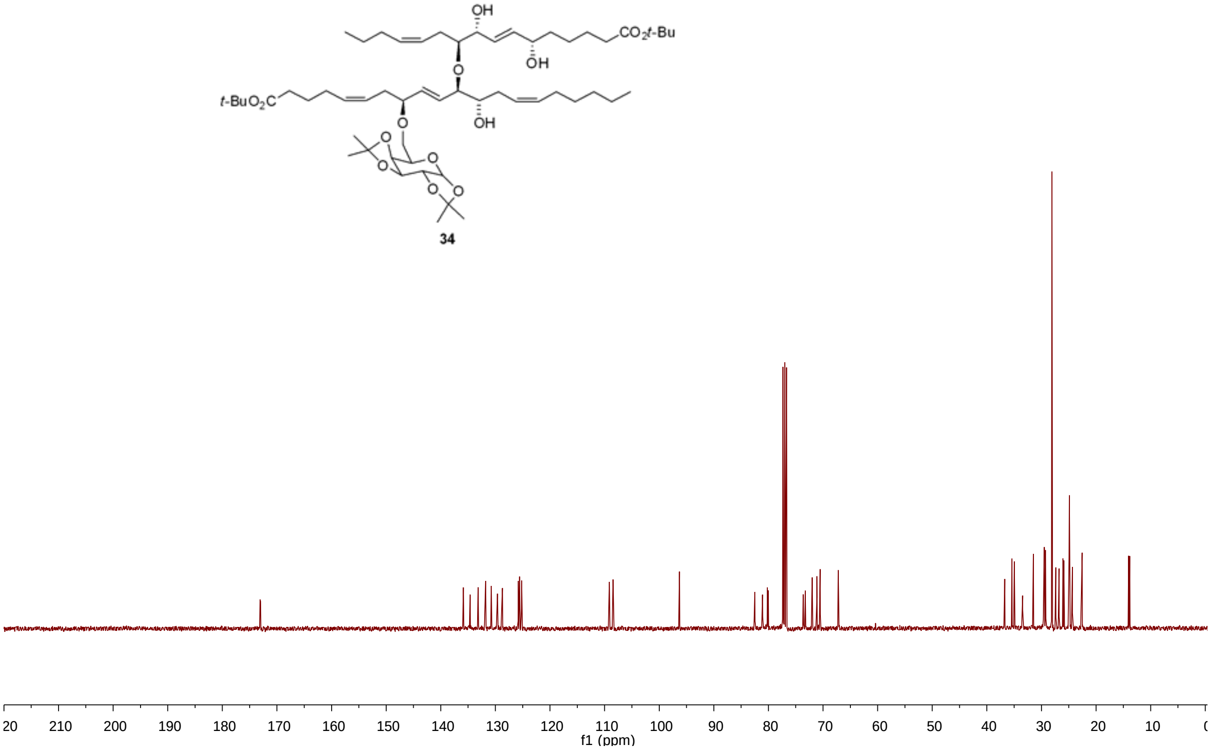
34
f1 (ppm)

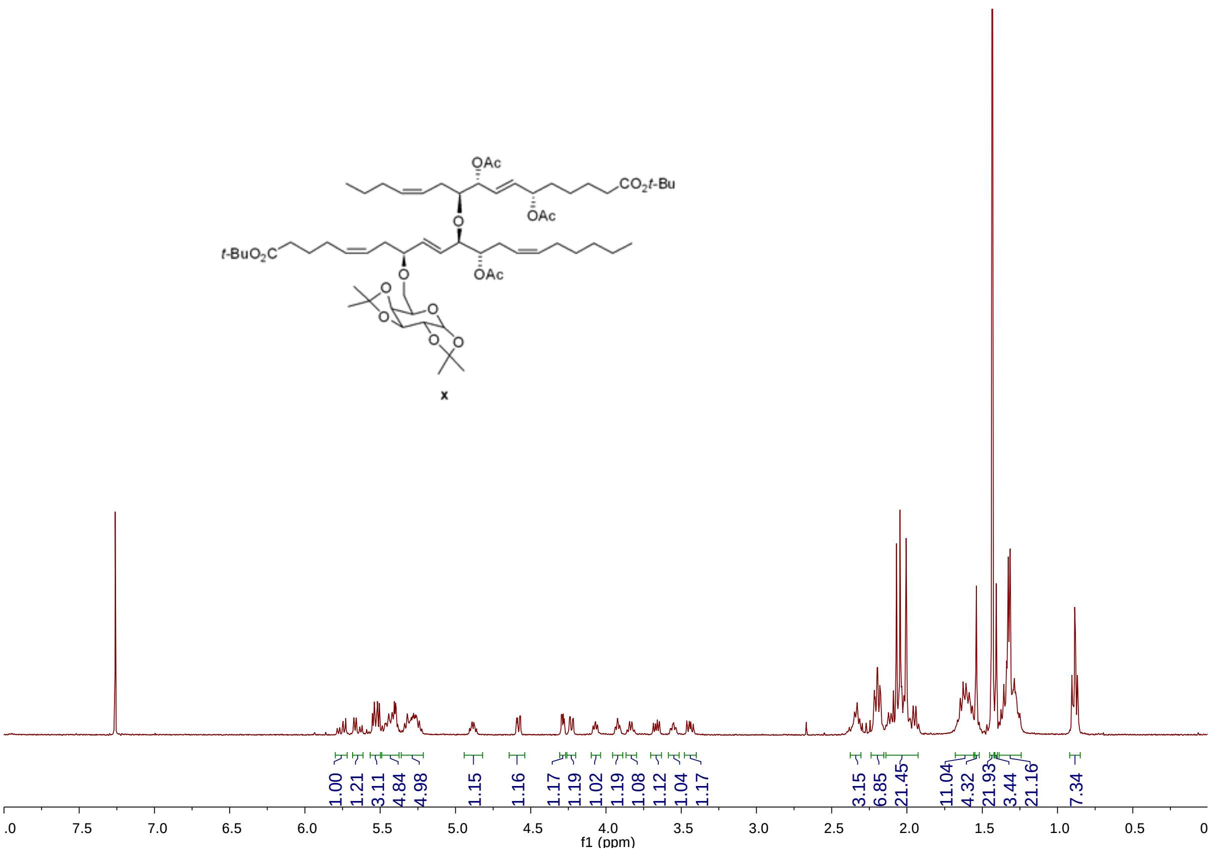

OAc
CO2t-Bu
OAc
O
t-BuO2C
OAc
O
x
1.00
1.21
3.11
4.84
4.98
1.15
1.16
1.17
1.19
1.02
1.19
1.08
1.12
1.04
1.17
3.15
6.85
21.45
11.04
4.32
21.93
3.44
21.16
7.34
f1 (ppm)

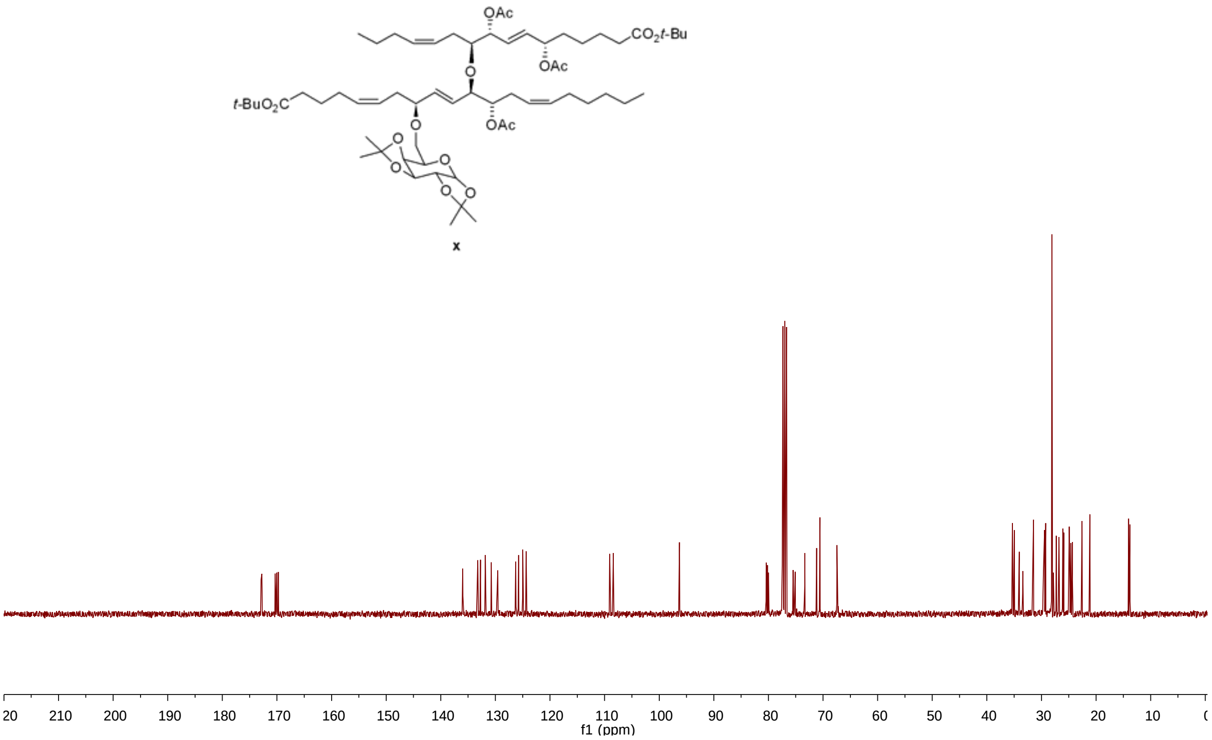

OAc
CO2t-Bu
OAc
O
t-BuO2C
OAc
O
x
f1 (ppm)

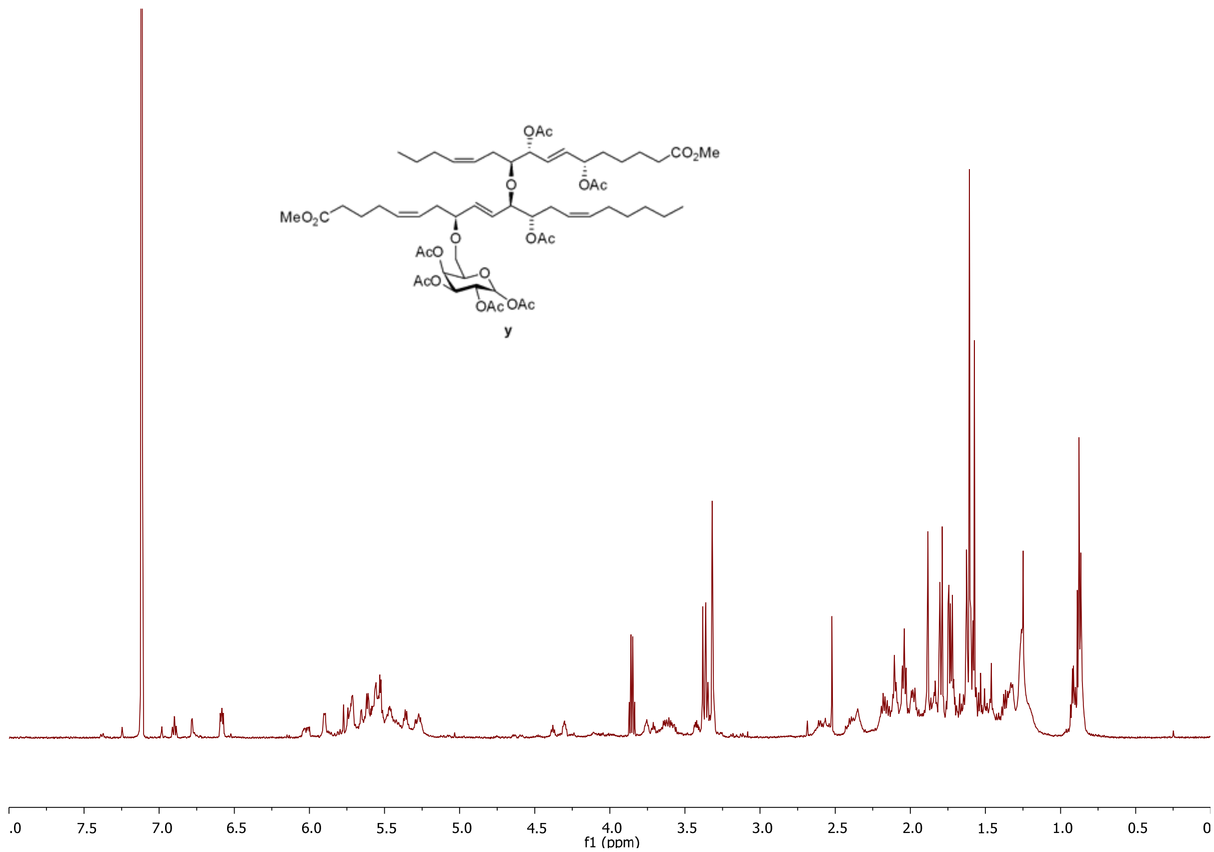

OAc
CO2Me
OAc
O
MeO2C
O
OAc
AcO
AcO
O
OAc OAc
y
.0
7.5
7.0
6.5
6.0
5.5
5.0
4.5
4.0
3.5
3.0
2.5
2.0
1.5
1.0
0.5
0
f1 (ppm)

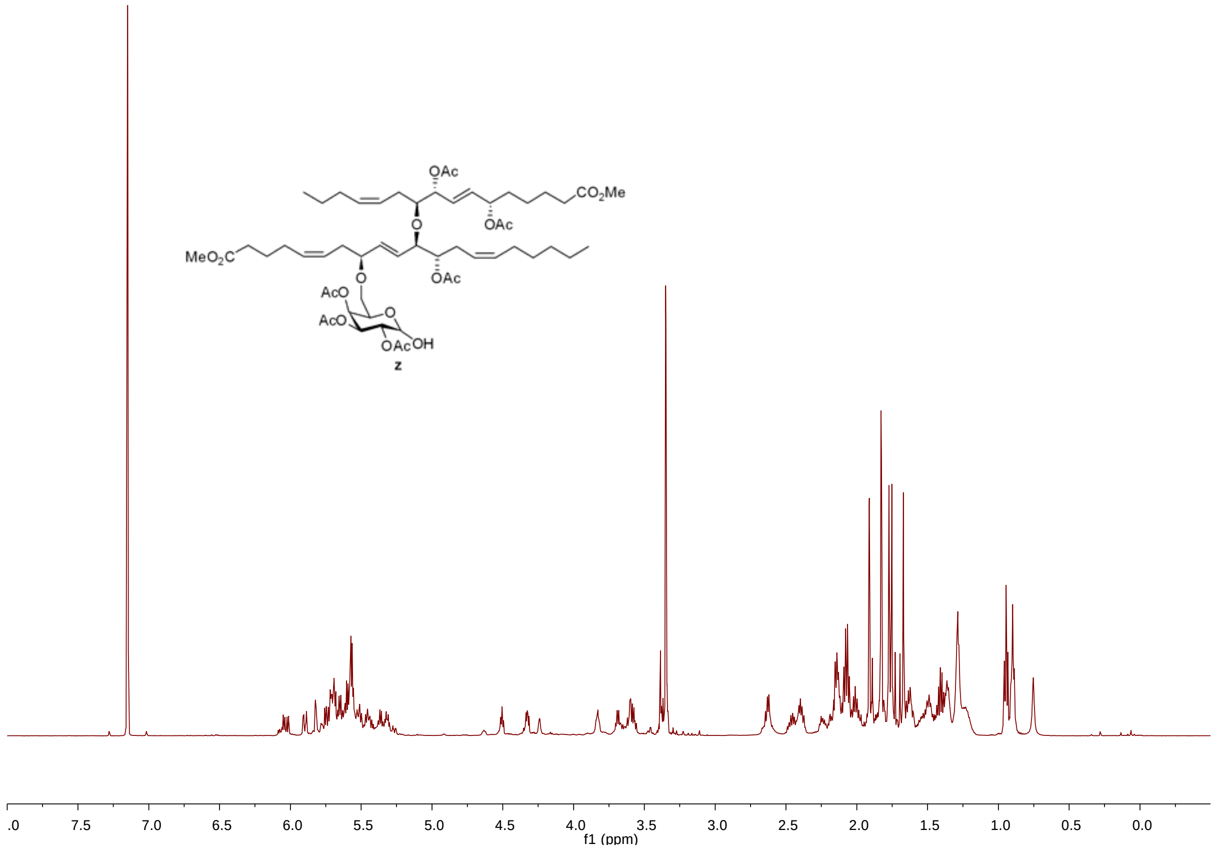

OAc
CO2Me
OAc
O
MeO2C
O
OAc
AcO
AcO
O
OAc OH
z
.0
7.5
7.0
6.5
6.0
5.5
5.0
4.5
4.0
3.5
3.0
2.5
2.0
1.5
1.0
0.5
0.0
f1 (ppm)

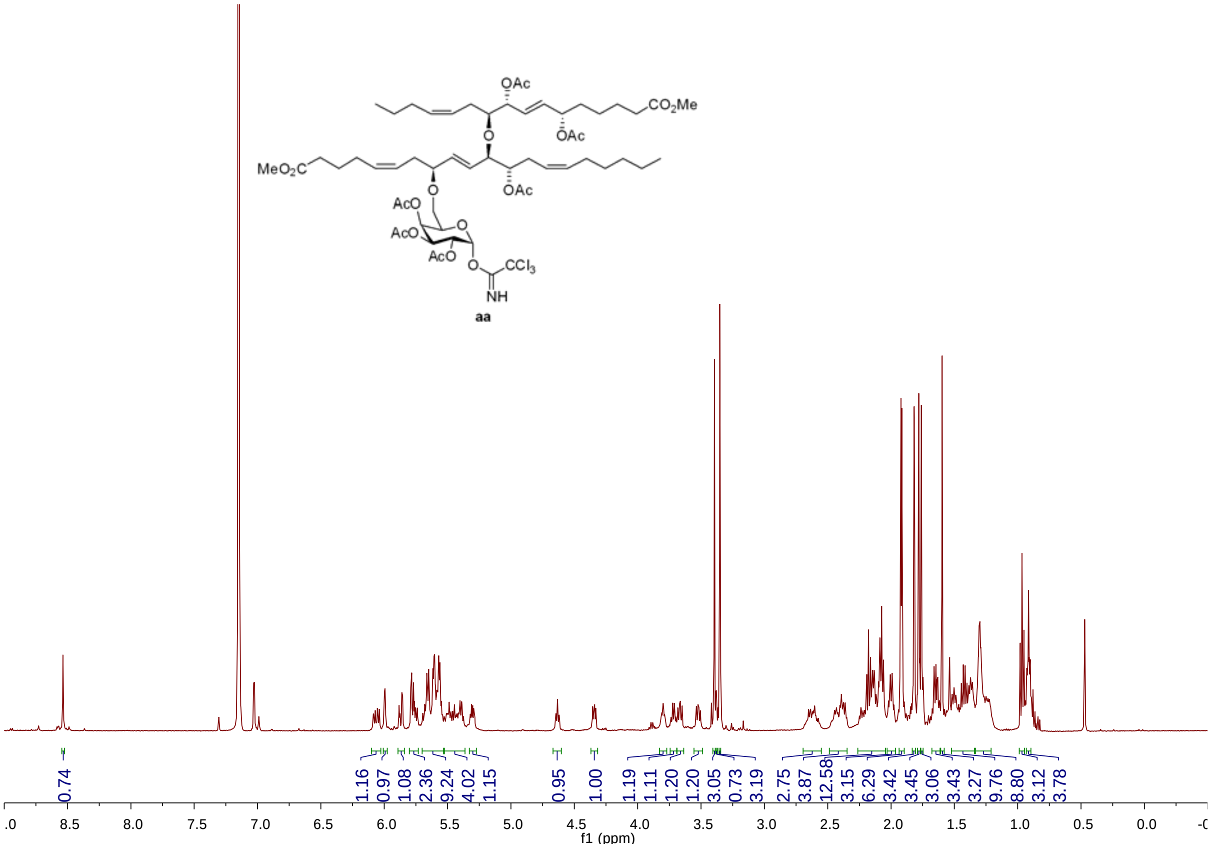
aa
f1 (ppm)

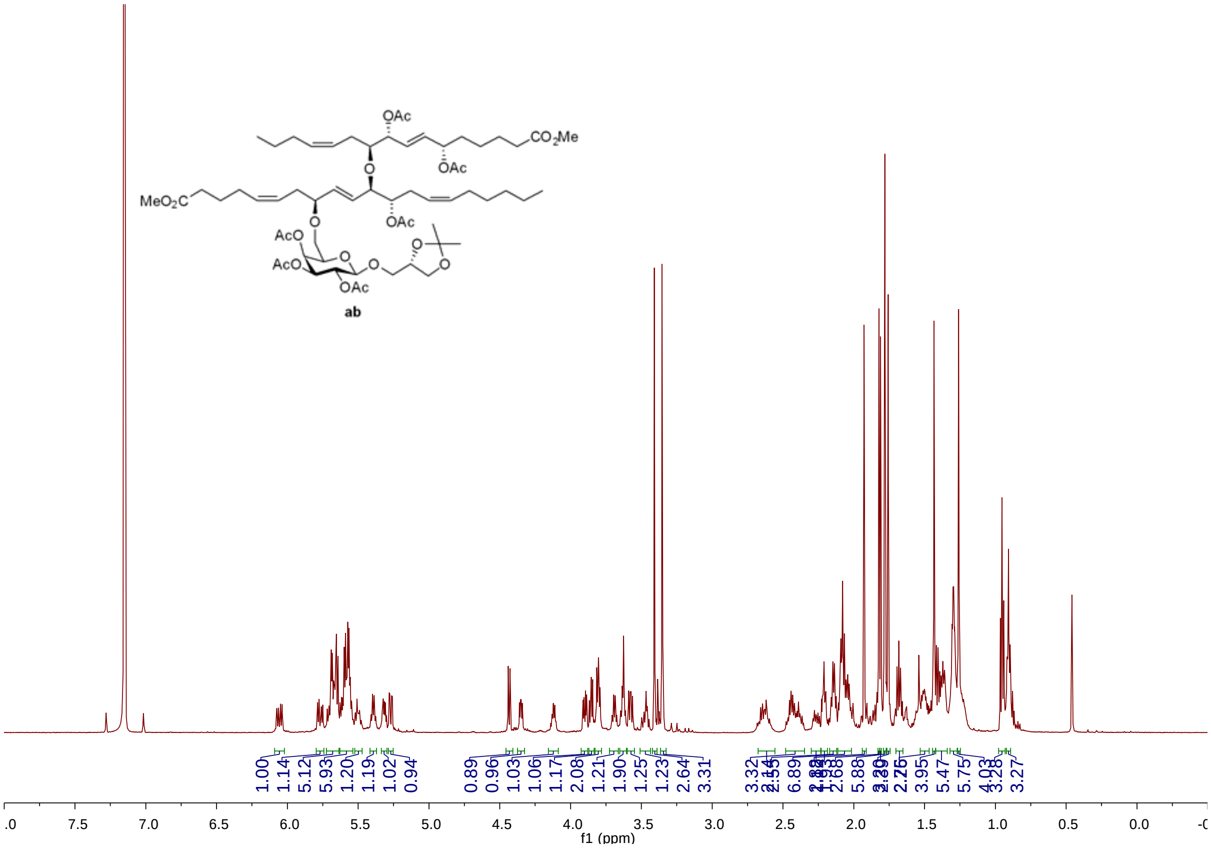
ab
f1 (ppm)

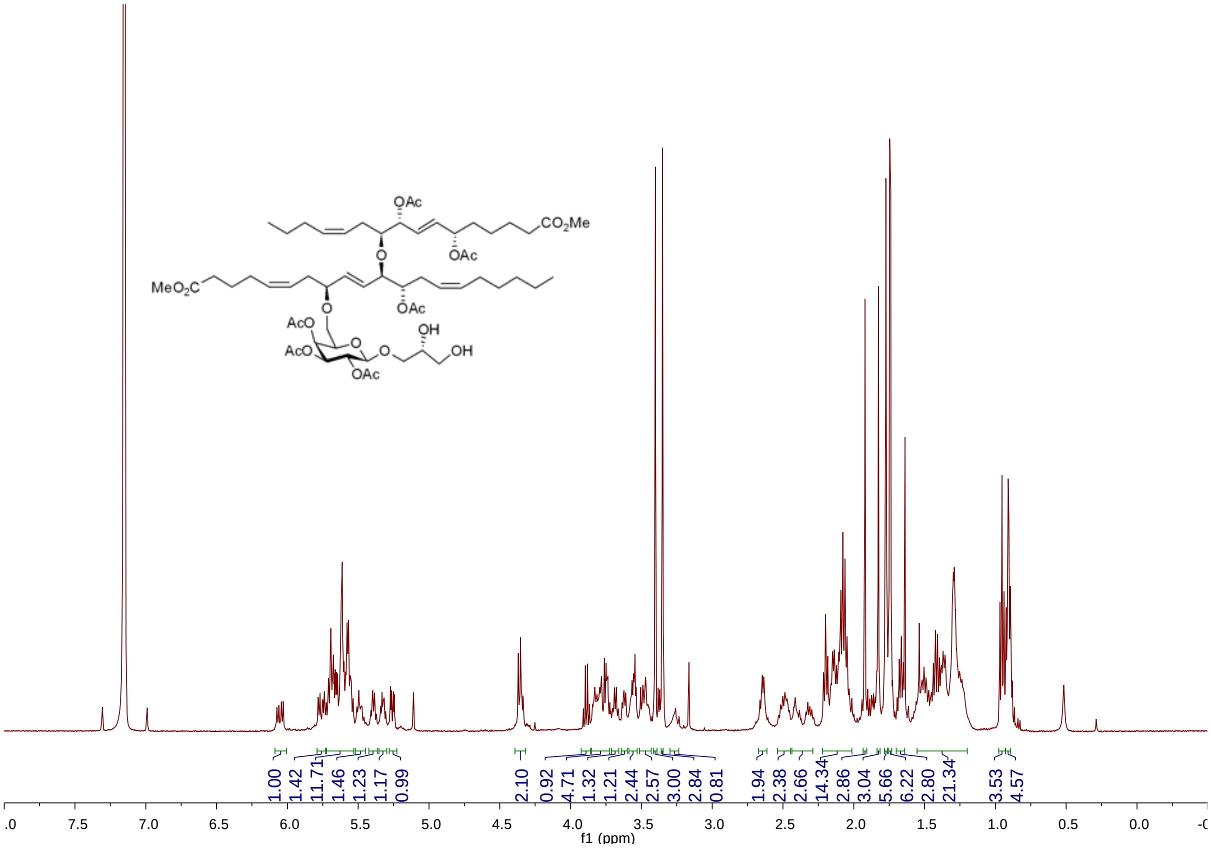
f1 (ppm)

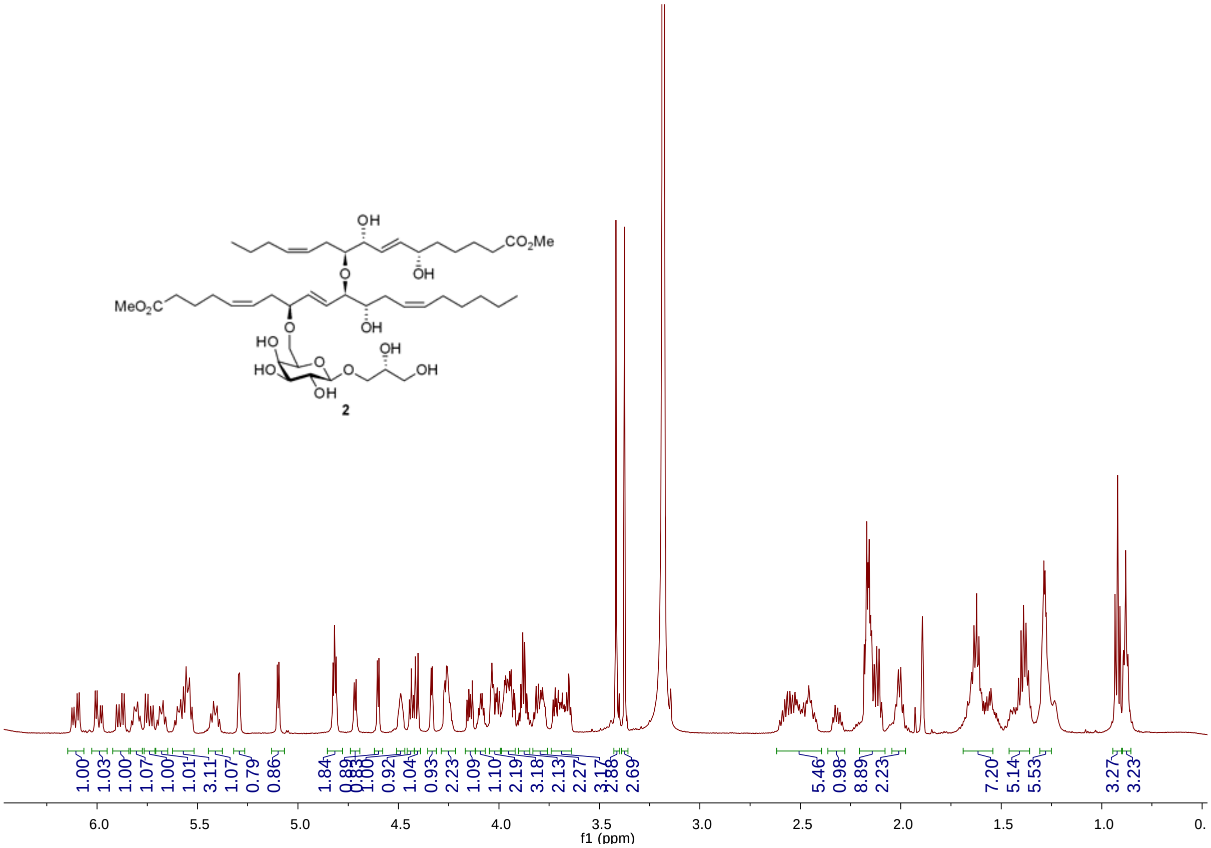
2
f1 (ppm)

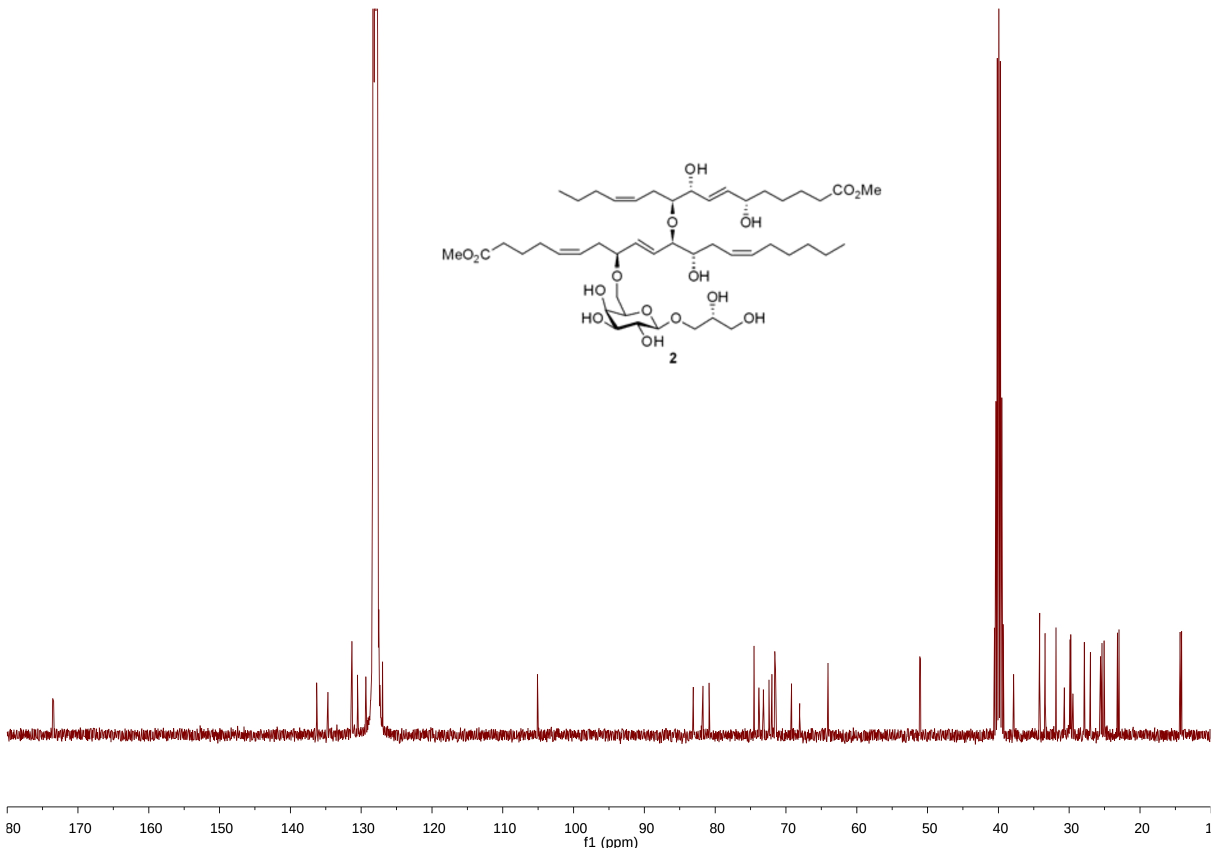

OH
CO2Me
O
OH
MeO2C
OH
O
HO
HO
O
O
OH
OH
OH
2
80
170
160
150
140
130
120
110
100
90
80
70
60
50
40
30
20
1
f1 (ppm)

gradCOSY60 NMR Spectrum of Nigricanoside A Dimethyl Ester **2** at 600 MHz in 25:2 C_6D_6/DMSO-d_6

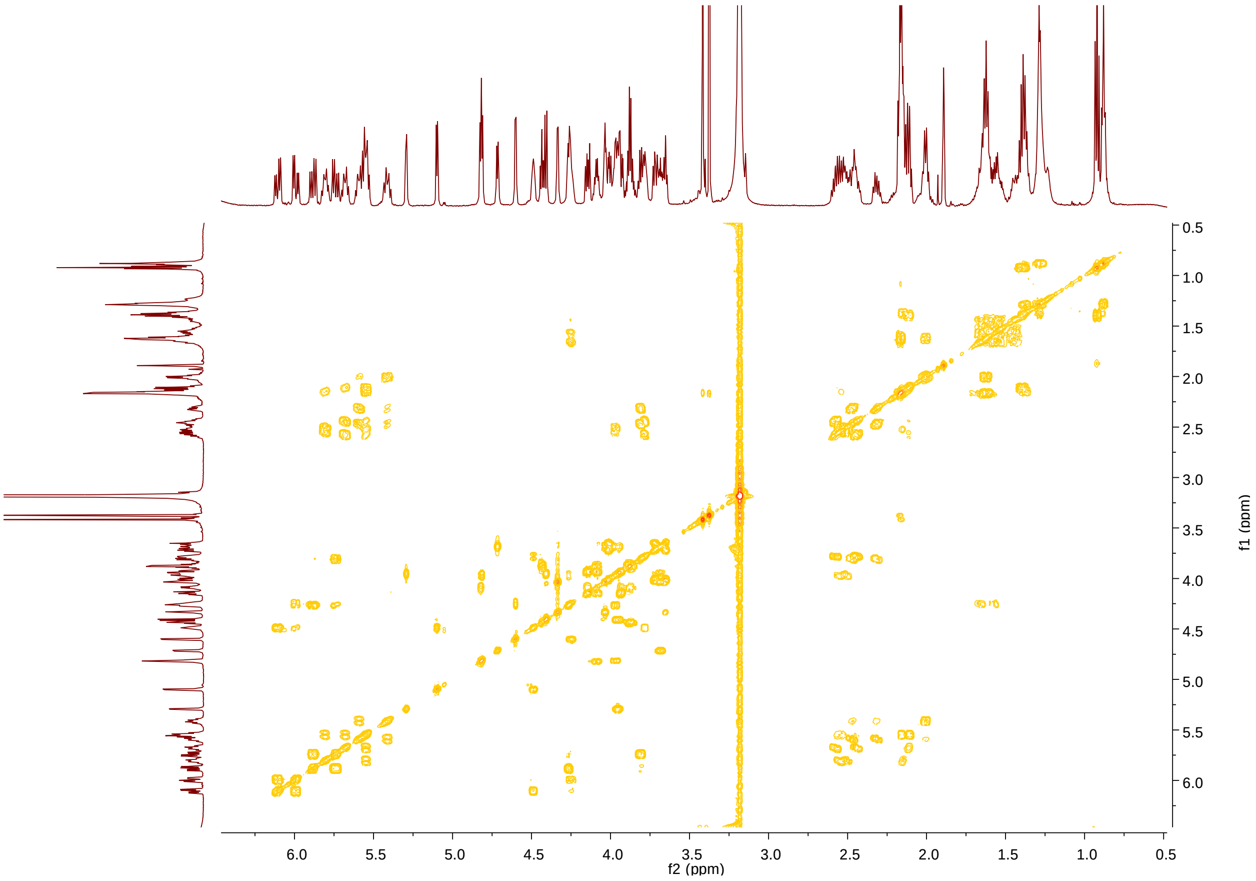

gradHSQC NMR Spectrum of Nigricanoside A Dimethyl Ester **2** at 600 MHz in 25:2 C_6D_6/DMSO-d_6

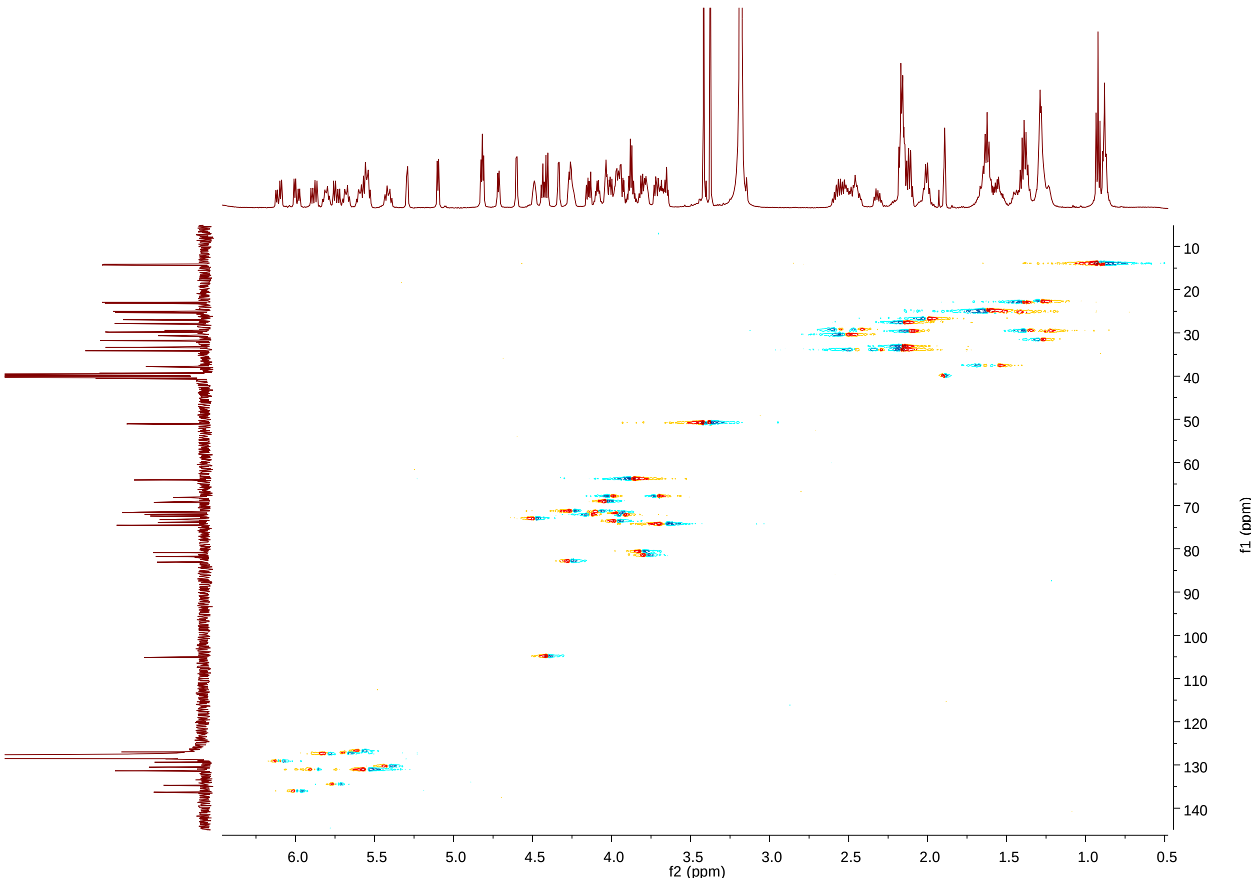

gradHMBC NMR Spectrum of Nigricanoside A Dimethyl Ester **2** at 600 MHz in 25:2 C_6D_6/DMSO-d_6

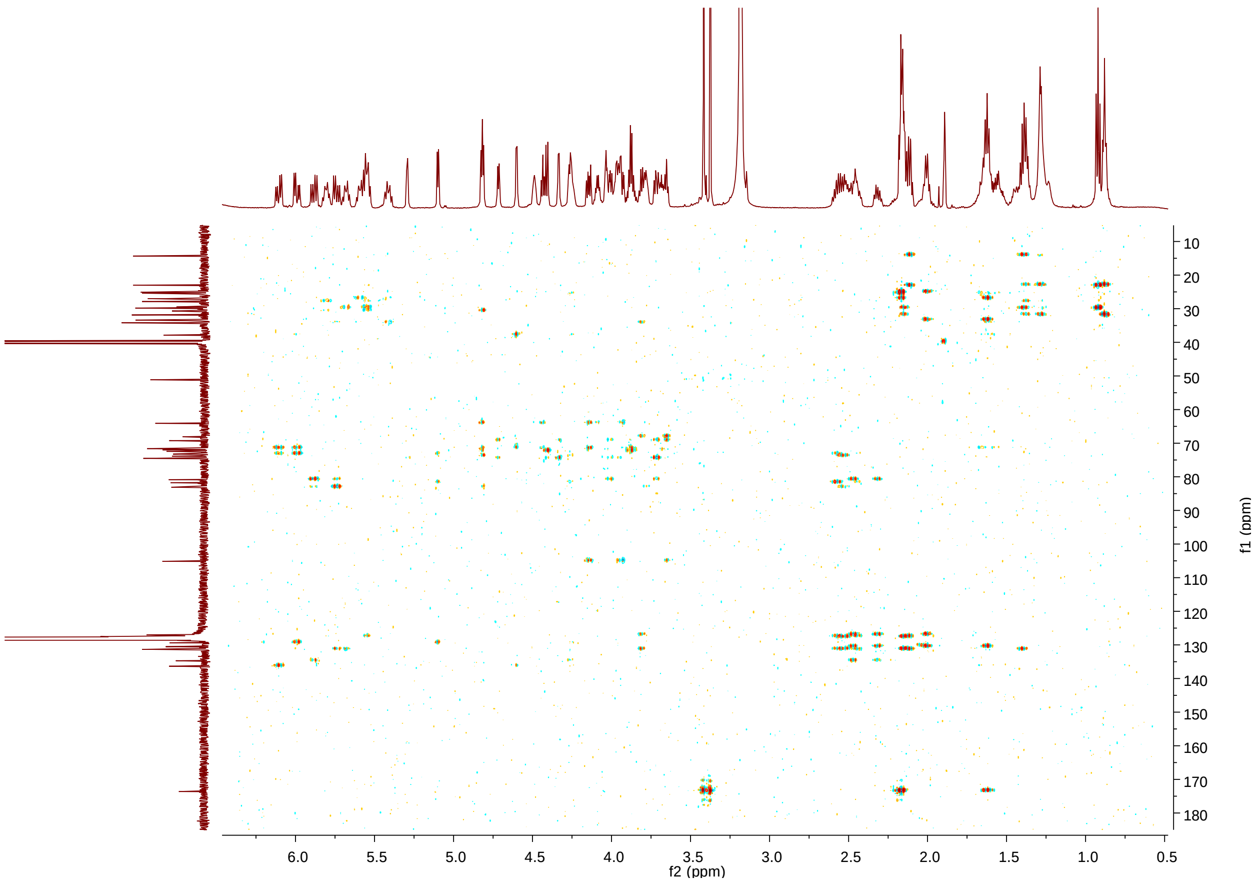

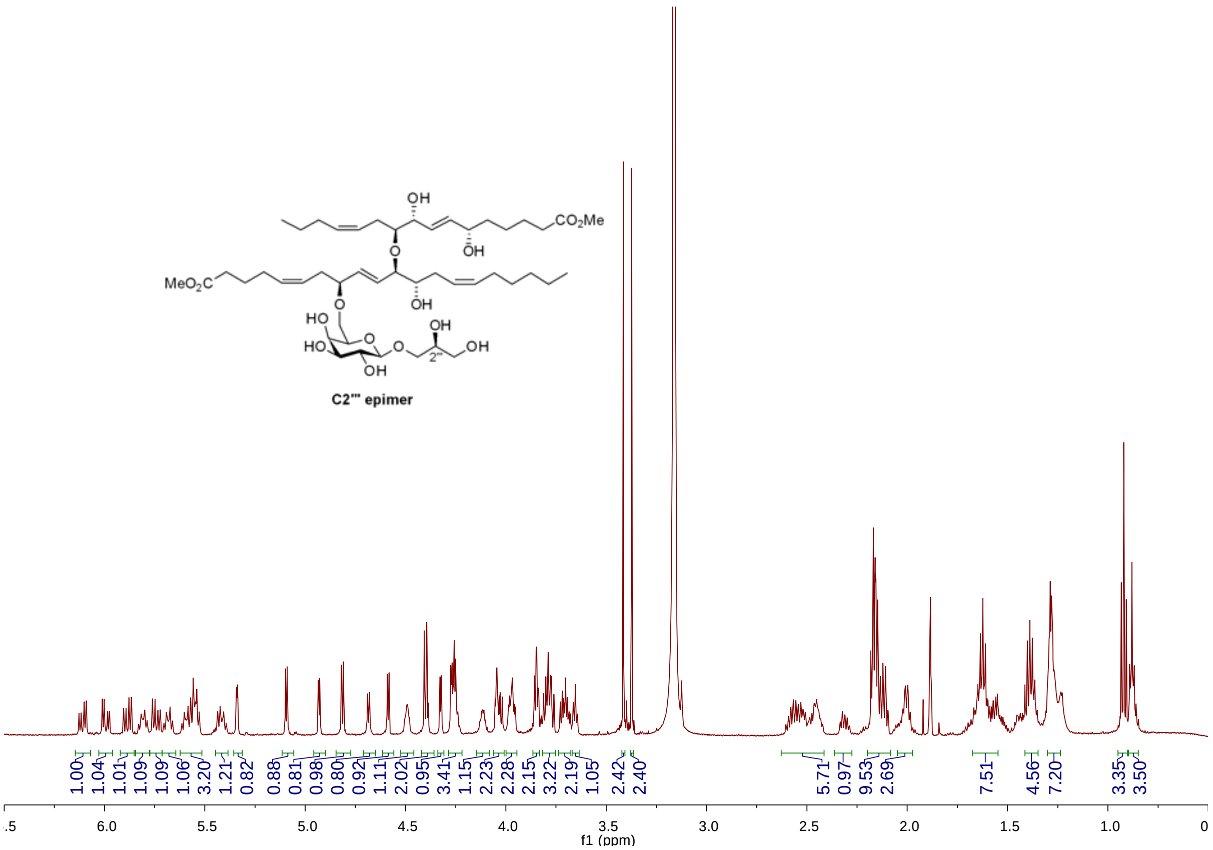
C2''' epimer
f1 (ppm)

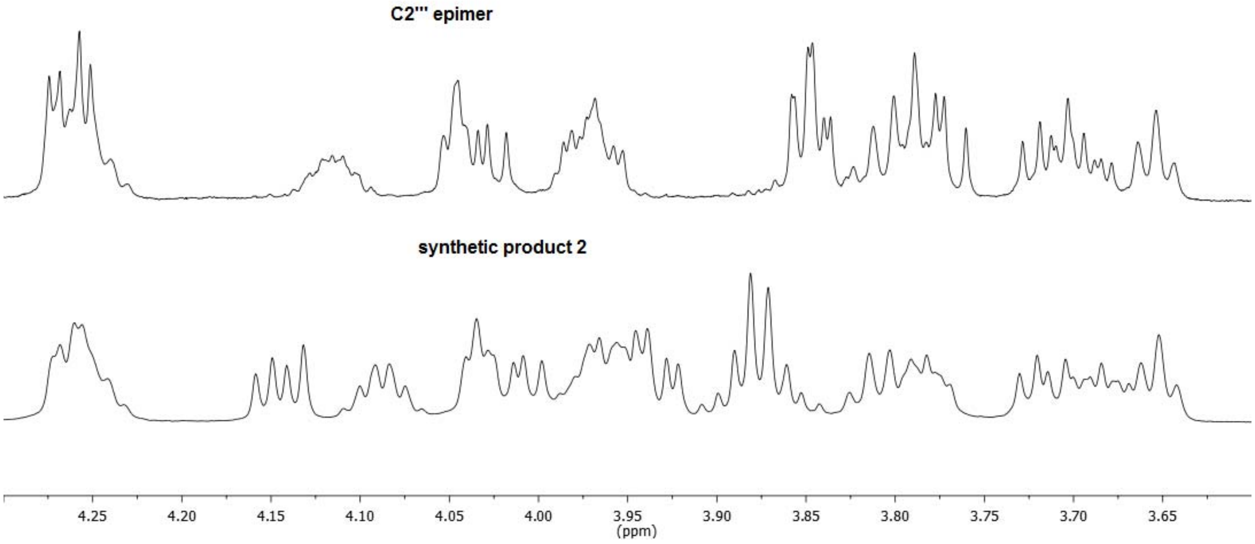
C2''' epimer
synthetic product 2
(ppm)

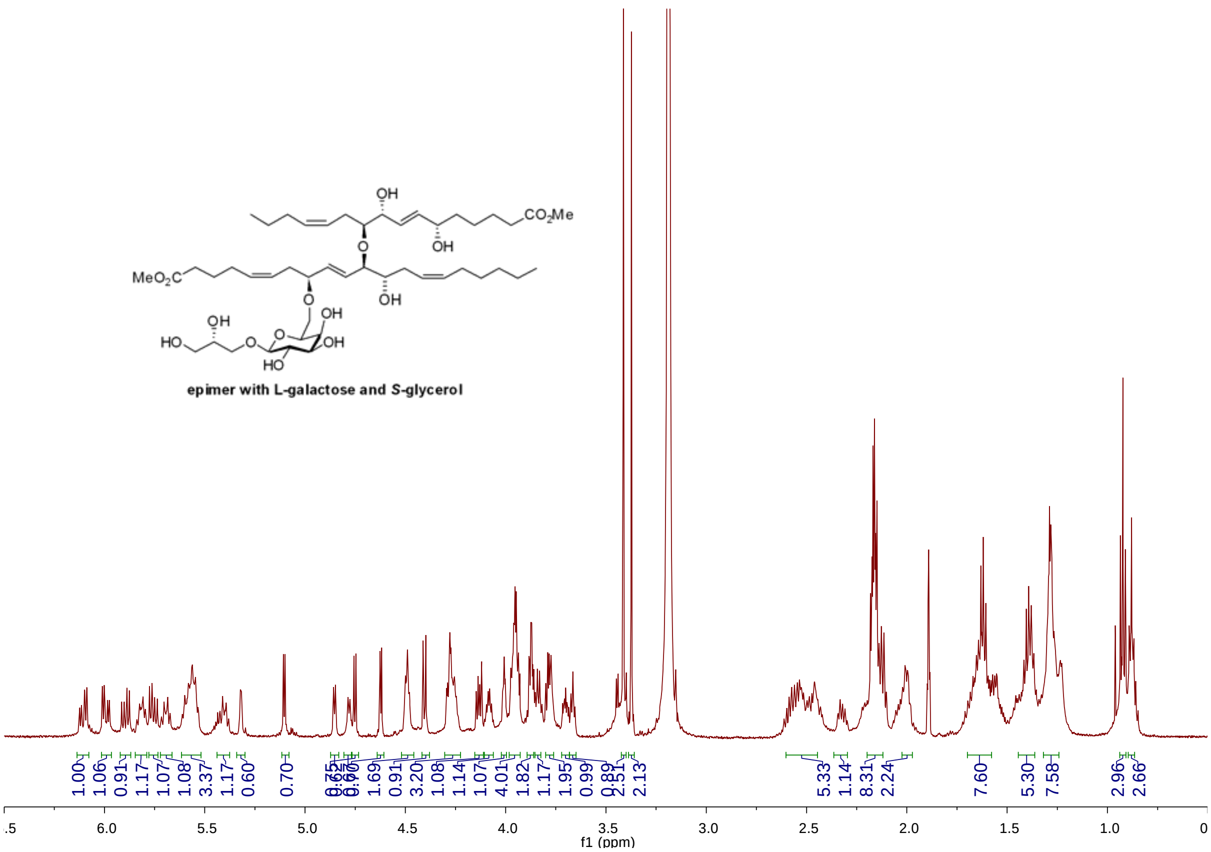
epimer with L-galactose and S-glycerol
f1 (ppm)

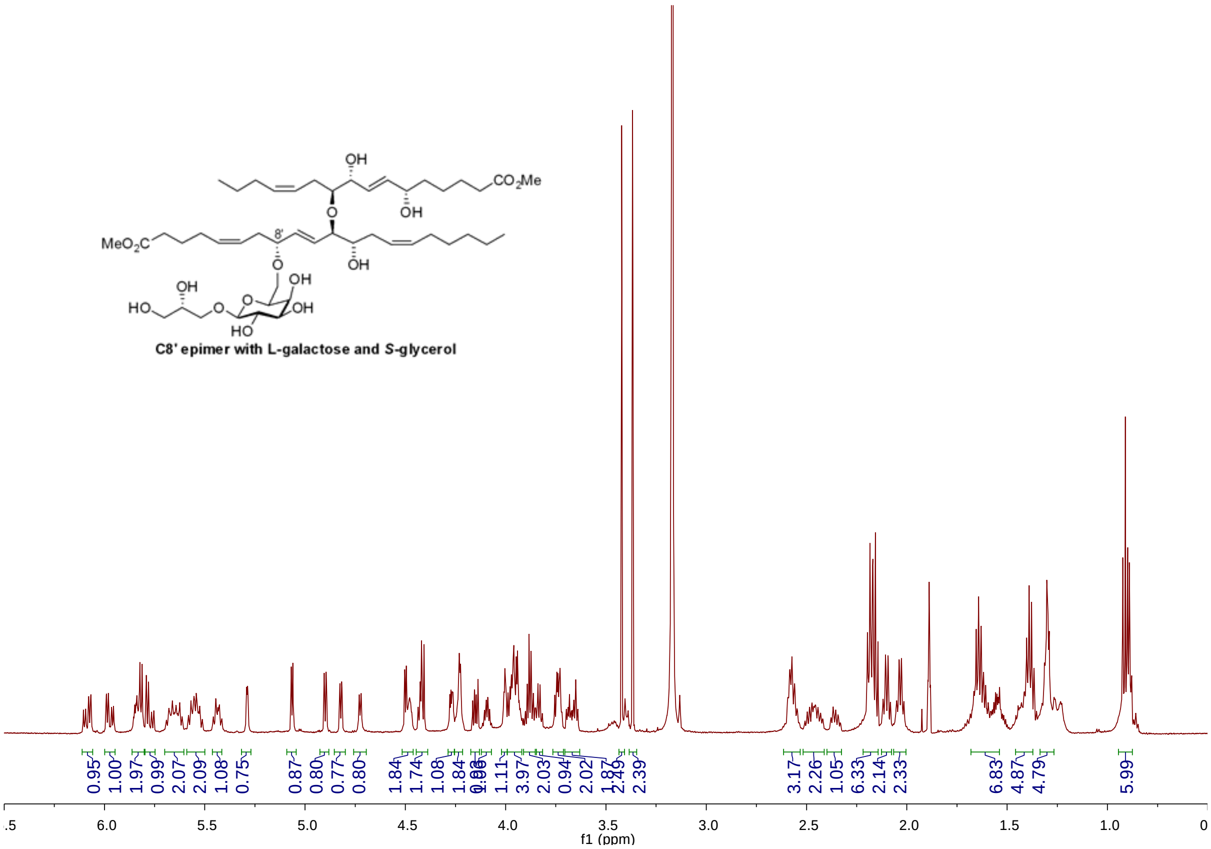
C8' epimer with L-galactose and S-glycerol
f1 (ppm)
